# Glioblastoma-instructed microglia transit to heterogeneous phenotypic states with phagocytic and dendritic cell-like features in patient tumors and patient-derived orthotopic xenografts

**DOI:** 10.1101/2023.03.05.531162

**Published:** 2023-03-06

**Authors:** Yahaya A. Yabo, Pilar M. Moreno-Sanchez, Yolanda Pires-Afonso, Tony Kaoma, Dimitrios Kyriakis, Kamil Grzyb, Suresh K. Poovathingal, Aurélie Poli, Andrea Scafidi, Arnaud Muller, Reka Toth, Anaïs Oudin, Barbara Klink, Guy Berchem, Christophe Berthold, Frank Hertel, Michel Mittelbronn, Dieter H. Heiland, Alexander Skupin, Petr V. Nazarov, Simone P. Niclou, Alessandro Michelucci, Anna Golebiewska

**Affiliations:** 1 NORLUX Neuro-Oncology Laboratory, Department of Cancer Research, Luxembourg Institute of Health (LIH), L-1526 Luxembourg, Luxembourg; 2 Faculty of Science, Technology and Medicine (FSTM), University of Luxembourg, L-4367 Belvaux, Luxembourg; 3 Neuro-Immunology Group, Department of Cancer Research, Luxembourg Institute of Health, L-1526 Luxembourg, Luxembourg; 4 Multiomics Data Science, Department of Cancer Research, Luxembourg Institute of Health, L-1445 Strassen, Luxembourg; 5 Luxembourg Centre for Systems Biomedicine (LCSB), University of Luxembourg, L-4362 Esch-sur-Alzette, Luxembourg; 6 Single Cell Analytics & Microfluidics Core, Vlaams Instituut voor Biotechnologie-KU Leuven, 3000 Leuven, Belgium; 7 National Center of Genetics, Laboratoire National de Santé, L-3555 Dudelange, Luxembourg; 8 Department of Cancer Research, Luxembourg Institute of Health, L-1526 Luxembourg, Luxembourg; 9 German Cancer Consortium (DKTK), 01307 Dresden, Germany, Core Unit for Molecular Tumor Diagnostics (CMTD), National Center for Tumor Diseases (NCT), 01307 Dresden, Germany; German Cancer Research Center (DKFZ), 69120 Heidelberg, Germany; 10 Institute for Clinical Genetics, Faculty of Medicine Carl Gustav Carus, Technische Universität Dresden, 01307 Dresden, Germany; 11 Centre Hospitalier Luxembourg, 1210 Luxembourg, Luxembourg; 12 Luxembourg Center of Neuropathology (LCNP), Luxembourg; 13 National Center of Pathology (NCP), Laboratoire National de Santé, L-3555 Dudelange, Luxembourg; 14 Department of Life Sciences and Medicine (DLSM), University of Luxembourg, L-4362 Esch-sur-Alzette, Luxembourg; 15 Microenvironment and Immunology Research Laboratory, Medical Center - University of Freiburg, Freiburg, Germany; 16 Department of Neurosurgery, Medical Center - University of Freiburg, Freiburg, Germany; 17 Department of Neurological Surgery, Lou and Jean Malnati Brain Tumor Institute, Robert H. Lurie Comprehensive Cancer Center, Feinberg School of Medicine, Northwestern University, Chicago, Illinois.; 18 Department of Physics and Material Science, University Luxembourg, L-4367 Belvaux, Luxembourg; 19 Department of Neuroscience, University of California San Diego, La Jolla, CA 92093, USA

**Keywords:** Glioblastoma, Tumor microenvironment, Myeloid cells, Microglia, Patient-derived orthotopic xenografts, Single-cell RNA-sequencing

## Abstract

**Background:**

Glioblastoma (GBM) evades the immune system by creating an immune-suppressive tumor microenvironment (TME), where GBM-associated myeloid cells are geared towards tumor-supportive roles. However, it is unclear whether recruited myeloid cells are phenotypically and functionally identical. Here, we aim to understand the TME heterogeneity in GBM patients recapitulated in patient-derived orthotopic xenografts (PDOXs) and systematically characterize myeloid cell type identities at the molecular and functional level.

**Methods:**

We applied single-cell RNA-sequencing and spatial transcriptomics, multicolor flow cytometry, immunohistochemistry and functional assays to examine the heterogeneity of the TME in GBM. Various GBM PDOXs representing different tumor phenotypes were analyzed and compared to the patient tumors, normal brain and mouse GL261 glioma model.

**Results:**

PDOX models recapitulate the major components of the TME detected in human GBM, where tumor cells reciprocally interact with host cells to create a GBM-specific TME. We detect the most prominent transcriptomic adaptations in myeloid cells, which are largely of microglial origin. We reveal intra-tumoral heterogeneity of microglia and identify diverse phenotypic states across distinct GBM landscapes and tumor niches. GBM-educated microglia acquire dendritic cell-like features, displaying increased migration and phagocytosis. We further find novel microglial states expressing astrocytic and endothelial markers. Lastly, we show that temozolomide (TMZ) treatment leads to transcriptomic plasticity of both GBM tumor cells and adjacent TME components.

**Conclusions:**

Our data provide insight into the phenotypic adaptation of the heterogeneous TME instructed by GBM. We uncover that GBM-educated microglia are represented by various concomitant states, both in patients and recapitulated in PDOXs, displaying different pro- or anti-tumoral properties that are modulated by anti-neoplastic treatments, such as TMZ.

## INTRODUCTION

Tumor microenvironment (TME) components, encompassing immune and non-immune non-malignant cells, have been recognized as key players in tumor initiation, progression and treatment resistance of literally all aggressive cancers^1^. Glioblastomas (GBMs), the most aggressive and incurable primary brain tumor, form a very dynamic ecosystem, where heterogeneous tumor cells reciprocally interact with various cells of the TME^2^. The brain TME includes endothelial cells, pericytes, astrocytes, neurons, oligodendrocytes and immune cells. Although GBMs are known as ‘cold tumors’ with very little lymphocytic infiltration^3^, the GBM TME contains up to 40% of myeloid cells, which, as a whole, are referred to as tumor-associated macrophages (TAMs) and are known to create a supportive environment facilitating tumor proliferation, survival and migration^4,5^. TAMs are mainly constituted by brain resident parenchymal microglia (Mg), peripheral monocytes (Mo) and perivascular macrophages, known as border-associated macrophages (BAMs)^6–8^. The proportions and functions of TAMs of different origins are not yet clearly defined due to the striking phenotypical and functional adaptation in the TME, lack of stable identifying markers and adequate preclinical models. Recent studies have shown that TAMs in GBM are different from classical pro-inflammatory activated (immune-permissive) M1 or alternatively activated (immune-suppressive) M2 reactive profiles^9,10^. Notably, it has been proposed that TAMs acquire different gene expression programs depending on the GBM subtype and upon GBM recurrence^11–13^. However, it is not yet well understood to what extent TAMs acquire diverse functional phenotypes *in vivo* depending on their origin, specific tumor niches, or along tumor development and progression^14^. Therefore, a better understanding of the functional TAM heterogeneity will pave the way for new therapeutic strategies targeting the myeloid compartment.

To achieve this critical aim, as the commonly used syngeneic and genetically engineered mouse models suffer from their limited resemblance to the human disease, reliable patient-derived brain tumor models are needed. In this context, while GBM patient-derived organoids preserve certain TME components during initial days only and *ex vivo* co-culture protocols are still immature^15^, patient-derived xenografts allow for propagation of primary patient tumors in less selective conditions than in *in vitro* cultures^16^. Additionally, as subcutaneous xenografts do not recapitulate the natural TME, patient-derived orthotopic xenografts (PDOXs) implanted in the brain are certainly more adequate for modeling gliomas. However, although showing an excellent recapitulation of GBM tumor features, PDOXs are characterized by an immunocompromised environment and the replacement of the human TME with mouse counterparts. It is therefore important to assess to what extent PDOXs can mimic major TME features observed in GBM patient tumors.

We have previously reported that PDOX models recapitulate the genetic, epigenetic and transcriptomic features of human tumors^17^. We have also shown that mouse cells interact with human GBM cells in the brain. In particular, endothelial cells forming blood vessels adapt their morphology and molecular features in analogy to the aberrant vasculature observed in patients^18^. We have further shown that mouse endothelial cells respond to anti-angiogenic treatment, as observed in GBM patients, leading to normalized blood vessels and treatment escape mechanisms towards more invasive tumors^19^. Here, we further inferred the heterogeneity of the TME compartment in PDOXs across genetically and phenotypically diverse GBM landscapes as well as upon temozolomide (TMZ) treatment. Focusing on TAMs, we distinguish their origin across GBM landscapes and identify distinct molecular programs. We show that Mg are a key component of the TME in GBM and transit towards heterogeneous phenotypic states in tumors of diverse genetic backgrounds. By interrogating single-cell RNA sequencing (scRNA-seq) and spatial transcriptomic profiles of GBM patient tumors, we confirm that the identified Mg states in PDOXs are abundant also in patients and localize variably across spatial TME niches. Notably, high proportions of these Mg-TAMs present dendritic cell-like features, including enhanced phagocytic and antigen-presenting cell characteristics. Lastly, we show that TMZ modulates the molecular features of the tumor and TME components, leading to a differential network between various components of the GBM ecosystem.

## RESULTS

### Single-cell RNA-sequencing analyses identify major TME components in GBM PDOXs

**Figure 1.**
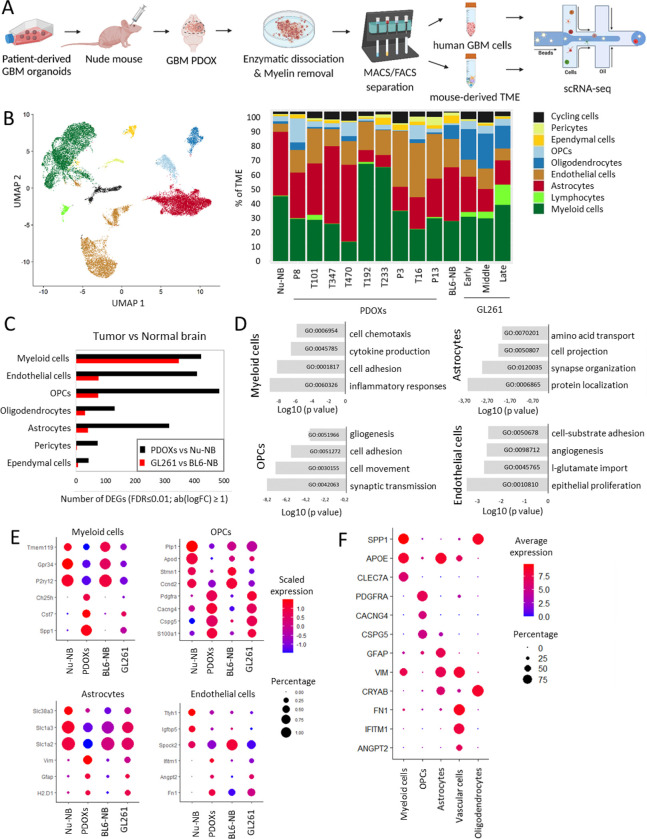
Transcriptomic adaptation of GBM-educated TME subpopulations in PDOXs *in vivo*. **(A)** Schematic of the experimental workflow showing tumor implantation, processing and scRNA-seq for GBM PDOXs. See PDOX characteristics in Fig. S1 and Table S1; **(B)** Left: UMAP projection of scRNA-seq data showing the overall gene expression profile of TME cell types detected. scRNA-seq data combined the biological groups: nude mouse normal brain (Nu-NB), PDOXs (9 models), C57BL6/N mouse normal brain (BL6-NB), GL261 tumor (3 collection time points: early, middle, late). Right: Proportions of TME cell types across different tumors and normal brains; Cell types are color-coded; OPCs: oligodendrocyte progenitor cells. **(C)** Differentially expressed genes (DEGs) between TME of PDOX and GL261 versus corresponding normal brains in identified cell types (FDR ≤0.01, |log_2_FC| ≥1, Wilcoxon rank sum test with Benjamini-Hochberg correction); **(D)** Top gene ontology terms characterizing DEGs in PDOX versus Nu-NB. **(E)** Gene expression levels of exemplary DEGs for distinct cell types in four biological groups: nude mouse brain (Nu-NB), PDOXs (9 models combined), C57BL6/N mouse normal brain (BL6-NB), GL261 tumor (3 time points combined); **(F)** Expression levels of exemplary markers in distinct cell types detected in human GBM tumors.

To assess TME composition in GBM PDOXs, we performed scRNA-seq on mouse-derived cells. PDOXs were derived by intracranial implantation of GBM organoids to nude mice, which have the least immunocompromised background compared to NOD/SCID and NSG strains. We selected nine genetically and phenotypically diverse models, which were derived from treatment-naïve and recurrent IDH wild-type GBMs (Fig 1A, Table S1–2, Fig S1A)^17^. Four models represented longitudinal tumors derived from GBM patients prior and after standard-of-care treatment, which included radiotherapy and TMZ (LIH0347: T347/T470, LIH0192: T192/T233)^17^. All tumors were micro-dissected, following the histopathological features of each model, to ensure minimal contamination of healthy brain. Mouse-derived cells of the TME were purified and processed by Drop-seq. In total we obtained 15,366 cells from nine PDOXs. The data were combined with Pires-Afonso et al. dataset^20^ of TME of the GL261 syngeneic orthotopic GBM mouse model derived in C57BL6/N (BL6/N) wild-type mice (3 time points, 2,492 cells in total). Normal brain controls were included for both mouse strains (1,692 cells/nude brain, 1,972 cells/BL6/N brain). Unsupervised clustering and uniform manifold approximation and projection (UMAP) analysis based on 21,522 cells and 24,067 genes in total, revealed nine major cellular clusters present in all samples (Fig 1B). Cell clusters were identified based on the expression of cell type-specific markers (Fig S1B) and included well-known components of normal brain and GBM TME such as astrocytes, endothelial and ependymal cells, pericytes, oligodendrocytes and oligodendrocyte progenitor cells (OPCs) as well as immune cells (Fig 1B). All major cellular subpopulations were present in PDOXs, GL261 and normal brain controls (Fig 1B). Similarly to patients, myeloid cells constituted the major immune component in PDOX and GL261 models. As expected, T lymphocytes were largely depleted and few functional B and NK cells were detected in PDOXs, (Fig S1C), while the majority of infiltrated lymphocytes in the GL261 TME were T and NK cells, with increased proportions upon tumor development (3.3–13.5%). GL261 displayed also higher proportions of oligodendrocytes (15–23%) compared to PDOXs (0.15–3.3%). In accordance with our previous report^18^, PDOXs with stronger angiogenic features (P13, T16, P3) had higher proportions of endothelial cells than more invasive PDOXs (Fig 1B), where no correlation between histopathological features and abundance of myeloid cells was observed. The TME composition exhibited a patient-specific trend, e.g., longitudinal models (LIH0347: T347/T470, LIH0192: T192/T233) showed similar cellular proportions, where PDOXs derived from LIH0192 patient showed a high percentage of myeloid cells, whereas PDOXs of LIH0347 patient were particularly abundant in astrocytes. This suggests a potential influence of the genetic background of tumor cells on the TME composition, as has been suggested in human GBMs^13^. Still, PDOXs with high myeloid content did not show an increased abundance of mesenchymal-like GBM tumor cells (Fig S1D). Lastly, we did not observe major differences between PDOXs derived from treatment-naïve and recurrent GBMs. In summary, mouse-derived TME in PDOXs is composed of cellular types relevant to human GBM^4^.

### TME subpopulations in PDOXs show transcriptional adaptation towards GBM-specific phenotypic states

To investigate the transcriptomic changes of the murine TME in PDOXs, we compared its subpopulations with the corresponding cells in the naïve nude mouse brain. We detected pronounced transcriptomic differences across all the populations of the TME that were mostly stronger than the changes observed in GL261 tumors when compared to normal BL6/N brain (Fig 1C, Fig S2A), thus indicating an effective crosstalk between human GBM tumor cells and mouse-derived TME. The transcriptomic adaptation was most pronounced in myeloid, endothelial cells, astrocytes, and OPCs (Table S3). Myeloid cells in PDOXs displayed transcriptomic programs linked to cell migration, inflammation and cytokine production (Fig 1D). Furthermore, key “homeostatic” Mg genes including *P2ry12*, *Tmem119* and *Gpr34*^21,22^ were downregulated (Fig 1E). In parallel, myeloid cells overexpressed GBM-specific TAM markers such as *Spp1* (Osteopontin), *Fn1*, *Cst7* and *Ch25h*. We confirmed myeloid cell adaptation across the PDOX and GL261 models by qPCR of FACS-sorted CD11b^+^ cells (Fig. S2B).

Interestingly, other cell types within the PDOX TME also activated biological processes linked to phenotypic states in GBM. For example, OPCs activated programs of tissue inflammation and regeneration (e.g., *Pdgfra, Cspg4, Cspg5, Cacng4* Fig. 1D–E, Fig S2C), astrocytes expressed genes linked to metabolic processes and cellular shape, suggesting ongoing reactive gliosis (e.g., upregulated *Gfap* and *Vim*, downregulated *Slc1a2* and *Slc1a3,* Fig 1D, Fig S2D) and, in agreement with our previous study^18^, endothelial cells displayed an activated and proliferative phenotype associated with angiogenesis. We detected similar profiles in corresponding TME subpopulations of human GBM tumors (n = 3589 cells from 4 IDHwt GBMs^23^, Fig 1F).

Altogether, these results point toward GBM-specific transcriptomic adaptations of myeloid cells and the main TME components in PDOXs.

### GBM-educated myeloid cells in PDOXs are largely of microglial origin

**Figure 2.**
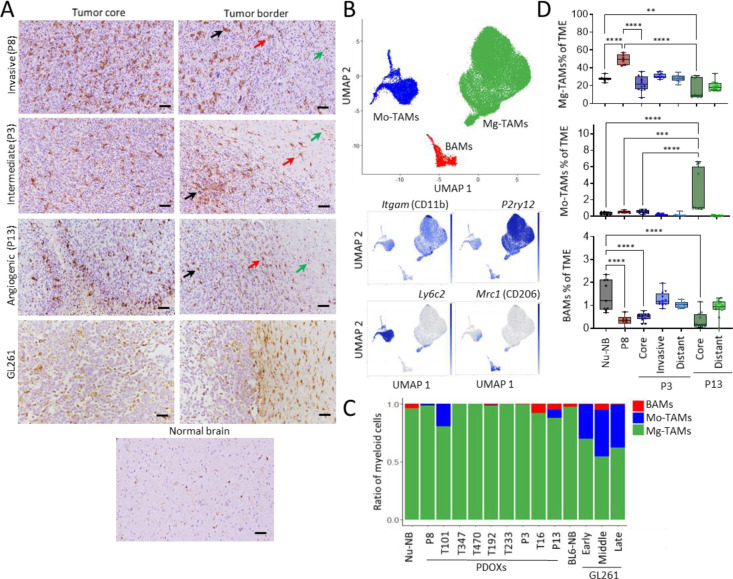
Ontogeny of GBM-educated myeloid cells. **(A)** Representative Iba1 staining in PDOXs representing invasive (P8), intermediate (P3) and angiogenic (P13) tumor growth, normal nude brain and GL261 tumor. Tumor core and tumor border zones are highlighted. Arrows indicate examples of ramified (green), hyper-ramified (red) and amoeboid (black) Mg. Scale bar: 50 μm. **(B)** UMAP projection of reference-based mapping of myeloid cells from TME of GBM PDOXs and GL261 tumors. Three myeloid cell types were identified: Microglia-derived tumor-associated macrophages (Mg-TAMs), blood monocyte-derived TAMs (Mo-TAMs) and border-associated macrophages (BAMs). Inserts show expression of marker genes: pan-myeloid: *Itgam* (CD11b), Mg: *P2ry12*, Mo: *Ly6c2*, BAMs: *Mrc1* (CD206). The color gradient represents expression levels. **(C)** Proportions of myeloid cell subpopulations in nude mouse normal brain (Nu-NB), PDOXs (9 models), C57BL6/N mouse normal brain (BL6-NB), GL261 tumor (3 collection time points: early, middle, late). **(D)** Box plots showing flow cytometry quantification of CD45^+^CD11b^+^Ly6G^−^Ly6C^−^CD206^−^ Mg-TAMs, CD45^+^CD11b^+^Ly6G^−^Ly6C^+^CD206^−^ Mo-TAMs and CD45^+^CD11b^+^Ly6G^−^Ly6C^−^CD206^+^ BAMs in Nu-NB and PDOX TME across different tumor phenotypes and brain regions (n=6–15 from at least three different mouse brains each, one-way ANOVA, ***p<0.001, **p<0.01, *p<0.05). See gating in Fig S3D.

Abundance of myeloid cells was further confirmed by Iba1^+^ staining in PDOXs (Fig 2A). While normal brain of nude mouse showed ‘surveilling’ ramified Mg, GBM tumors in PDOXs displayed TAMs with different morphologies. The cellular tumor was in general occupied by TAMs showing amoeboid or hyper-ramified morphology, matching Mg-derived TAMs (Mg-TAMs). Tumors displaying less invasive growth showed a gradient of TAM phenotypes at the invasive front, from ramified towards hyper-ramified and amoeboid Mg-TAMs. Myeloid cells with macrophagocytic morphology were especially present in areas of pseudopalisading necrosis (P13 PDOX). Invasive tumors showed more uniform, diffuse infiltration and activation of myeloid cells towards amoeboid states at the border with normal brain structures. This contrasted with the much sharper delineated GL261 tumor, showing a pronounced accumulation of Mg-like TAMs at the tumor border and putative Mo-derived TAMs (Mo-TAMs) in the tumor center (Fig 2A).

Due to pronounced heterogeneity within the TME, we further examined the ontogeny of the myeloid cells. We combined our dataset with previously published scRNA-seq data of myeloid cells in GL261 tumors, which assigned ontogeny of TAMs to Mg, Mo and BAMs based on transcriptomic profiles^7,8,20^ (Fig 2B–C, Fig S3A–C). Referencing PDOX myeloid cells to the Ochocka et al.^7^ dataset confirmed a high abundance of Mg-TAMs (81–100%) and a low proportion of Mo-TAMs (0–19%) and BAMs (0–8%). P13 PDOX with pronounced angiogenic features showed a higher proportion of Mo-TAMs and BAMs (7% and 5% respectively), although Mo-TAMs were also present in invasive T101 PDOX. In contrast, GL261 tumors contained significantly more Mo-TAMs (Fig 2C). Flow cytometry confirmed high proportions of Mg-TAMs in PDOX TME compared to Mo/BAMs-TAMs (Fig 2D, Fig S3D–E). While BAM proportions remained similar, we confirmed higher proportions of Mo-TAMs in the tumor core of P13 PDOXs. It was accompanied by increased levels of neutrophils and lymphocytes suggesting that peripheral Mo infiltration is limited to the angiogenic/necrotic tumor regions (Fig S3F) where the blood-brain barrier is disrupted.

Of note, we revealed major differences across GL261 datasets, with Ochocka et al.^7^ and Pires-Afonso et al.^20^ datasets containing 26–32% Mo-TAMs, whereas Pombo Antunes et al.^8^ dataset carrying more than 78% Mo-TAMs, according to the different adopted cell isolation strategies (Fig S3C). In fact, within the first two studies, myeloid cells have been isolated at early stages of tumor development from a larger part of the tumor-containing hemisphere, whereas in Pombo Antunes et al., cells have been extracted at the late stage and specifically from the tumor center. This highlights that Mg/Mo proportions may depend on the sampling approach, suggesting diverse spatial locations in the tumor and adjacent brain regions.

### Mg-TAMs display heterogeneous transcriptional programs

**Figure 3.**
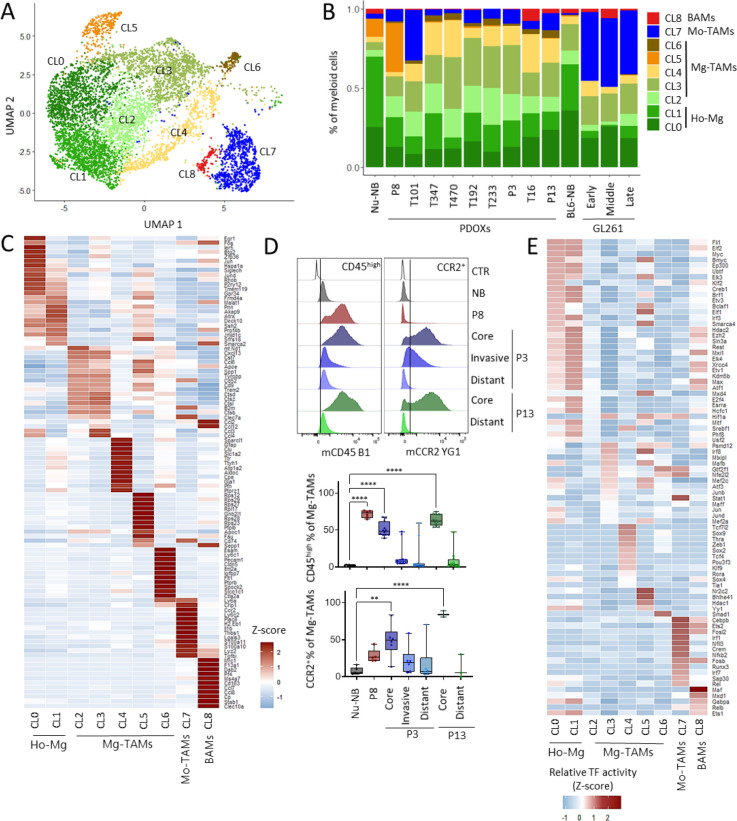
GBM-driven activation of homeostatic Mg towards heterogeneous Mg-TAMs. **(A)** UMAP plot showing clusters of myeloid cells in PDOXs, GL261 and normal brain controls revealing nine distinct clusters (CL). CL0–6 represent Mg-TAMs, CL7 Mo-TAMs, CL8 BAMs. **(B)** Proportions of cells assigned to nine clusters of myeloid cells in nude mouse normal brain (Nu-NB), PDOXs (9 models), C57BL6/N mouse normal brain (BL6-NB), GL261 tumor (3 collection time points: early, middle late). CL0–1: homeostatic Mg (Ho-Mg), CL2–6: Mg-TAMs, CL7: Mo-TAMs, CL8: BAMs. **(C)** Discriminative marker genes for each myeloid state (row z-scores of the expression levels) **(D)** Representative flow cytometry graphs of CD45^+^CD11b^+^Ly6G^−^Ly6C^−^CD206^−^ Mg-TAMs showing increased levels of CD45 and CCR2 in tumor core of three PDOX models. Bottom: quantification of CD45^high^ and CCR2^+^ cells across different tumor phenotypes and brain regions (n=3–6 from at least three different mouse brains each, one-way ANOVA, ***p<0.001, **p<0.01, *p<0.05). **(E)** Relative transcription factor (TF) activity of regulons identified by SCENIC in myeloid clusters.

We next took advantage of our unique dataset to interrogate the phenotypic heterogeneity of Mg in normal brain and tumors of different histopathological features. Reference-free analysis of our in-house data stratified Mg into seven phenotypic clusters (CL0–6), whereas CL7 and CL8 displayed transcriptional profiles of Mo-TAMs and BAMs, respectively (Fig 3A–C, Table S4). Homeostatic Mg (Ho-Mg), highly enriched in normal brain, grouped into two clusters (CL0–1), with CL1 showing lower expression levels of homeostatic genes (e.g. *P2ry12, Tmem119, Gpr34,* Fig 3C). Importantly, this was not the result of Mg activation via enzymatic digestion, since the markers of enzymatically-activated Mg (e.g., *Erg1*, *Fos*^24^) were expressed rather by a subset of Ho-Mg in CL0. Five phenotypic states were observed to be enriched in Mg-TAMs (CL2–6) (Fig 3A–C, Table S4). These included classical pro-tumorigenic Mg-TAMs represented at the highest levels in CL2 and CL3, high for e.g. *Spp1, Cst7, Cxcl13 and Apoe* (Fig 3C). Among these two groups, CL3 presented higher cytokine expression levels (*Ccl3, Ccl4*), suggesting stronger secretory properties and education by GBM when compared with CL2. As expected, these Mg-TAMs showed higher *Ptprc* (CD45) expression and lower levels of homeostatic Mg genes (Fig S4A). This is reminiscent of the decrease of homeostatic genes in reactive Mg, known to occur in GBM TME, but also under inflammatory and neurodegenerative conditions^25–28^. Mg transition towards CD45^high^ and Ccr2^+^ TAM states in the tumor core was further detected by flow cytometry (Fig 3D). While Mg in distant brain areas (P3, P13 PDOXs) resembled normal brain characteristics, the invasive niche (P3 PDOX) showed partial activation of Mg towards Mg-TAMs. Of note, CD45^high^ Mo-TAMs also increased CCR2 levels in the tumor core (Fig S4B).

Additional subpopulations included Mg-TAMs displaying astrocytic features (CL4, e.g. *Sparcl1, Gfap*), high transcriptional activity (CL5, e.g. *Rps12*) and expression of endothelial cell markers (CL6, e.g., *Pecam1, Cldn5*). Interestingly, CL6 cells were enriched for the expression of receptors known to play a role in the reciprocal crosstalk with tumor cells (e.g., *Flt1, Kdr*), whereas CL5 cells expressed periostin (*Postn*), previously shown to be secreted by GBM cells to promote the recruitment of immune-suppressed TAMs favoring malignant growth^29,30^. We excluded the contamination by other TME subpopulations within these clusters as these cells expressed myeloid cell markers, including *Hexb*, *Csf1r*, *Itgam* (CD11b) and *Ptprc* (Fig S4A) and showed low score for potential doublets (Fig S4C). The trajectory analysis also revealed a general transition from Ho-Mg to Mg-TAMs, without profound differences between Mg-TAM states (Fig S4D). Cell cycle genes were low across different clusters (e.g., *Mki67*, *Cd34* Fig S4A) and no cell cycle component was identified by a standard application of consensus-independent component analysis (Fig S4E). Importantly, all PDOXs showed pronounced transitions towards heterogeneous Mg-TAM states, although with variable proportions (Fig 3B).

Despite the differences in proportions, the transcriptomic profiles of Mg-TAMs, Mo-TAMs and BAMs were generally similar between PDOXs and GL261 tumors, except for several activation genes, e.g. higher levels of *Spp1*, *Apoe, Cxcl13* in PDOX Mg-TAMs and higher *H2.Eb1, Cxcl2* and *Arg1* in Mo-TAMs in GL261 (Fig S5A–B, Table S5). Overall, taking into account the proportions of myeloid entities and their activation states, our data suggest that the dominant population may acquire stronger immune response and immunosuppressive features.

We next sought to reveal regulators of these Mg states by conducting SCENIC analyses^31^ (Fig 3E). We identified a high number of regulons for Ho-Mg states including Hdac2 and Ezh2. While Mg-TAM CL2 state did not show specific enrichments of transcription factor activity, other Mg-TAMs displayed unique regulons. For example, pro-tumorigenic Mg-TAMs (CL3) were regulated by Hif1a, Stat1, Nfe2l2 and Mafb, suggesting a role of hypoxia in Mg state transitions. Astrocytic-like Mg-TAMs (CL4) showed high activity for Thra, Sox9 and Sox2, known to regulate astrocytic states. Transcriptionally active Mg-TAMs (CL5) scored high for Hdac1, Mitf and Elf1 activity and endothelial-like Mg-TAMs (CL6) showed enrichment for Foxp1. These regulatory networks were distinct from regulons active in Mo-TAMs and BAMs. These data further highlight the factual differences between transcriptomic states of Mg and show the impact of TME niches in shaping Mg heterogeneity in GBM.

### Mg-TAMs display immunologically active states with increased capacity for chemotaxis, phagocytosis and dendritic cell-like features

**Figure 4.**
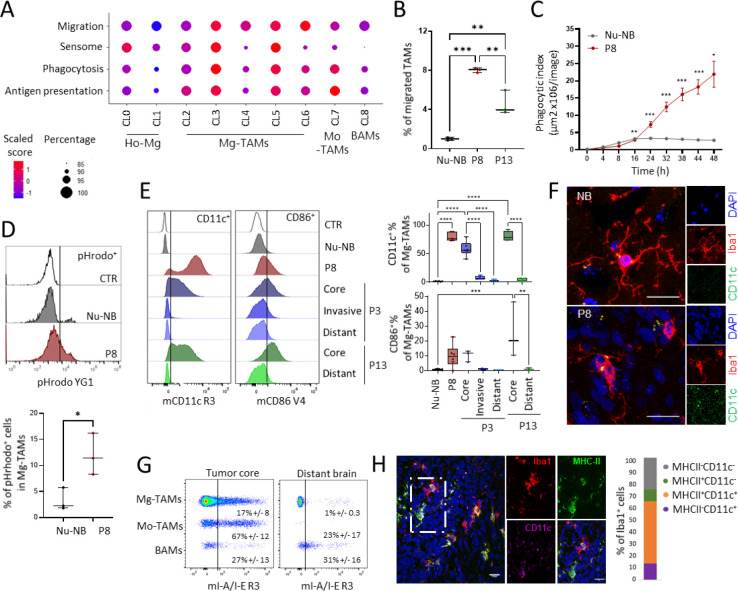
Functional properties of GBM-educated microglia. **(A)** Signature score of genes associated with migration, sensome, phagocytosis and antigen presentation per myeloid cluster: CL0–1 homeostatic Mg (Ho-Mg), CL2–6: Mg-TAMs, CL7: Mo-TAMs, CL8: BAMs. **(B)** *Ex vivo* assessment of migratory capacity in CD11b^+^ cells isolated from Nu-NB and PDOXs (P8, P13) **(**mean ± SEM, ** p < 0.01, *** p < 0.001, one-way ANOVA). **(C)** *Ex vivo* assessment of phagocytic capacity in CD11b^+^ cells isolated from Nu-NB and PDOX P8 (n=3–4, mean +/− SEM, two-way ANOVA followed by Tukey’s multiple comparisons, *p<0.05, ** p < 0.01, *** p < 0.001). (**D**) Phagocytic uptake measured by flow cytometry in CD45^+^CD11b^+^Ly6G^−^Ly6C^−^CD206^−^ Mg-TAMs (n=3, *p<0.05, two-tailed Student’s t-test) **(E)** Representative flow cytometry graphs of CD45^+^CD11b^+^Ly6G^−^Ly6C^−^CD206^−^ Mg-TAMs showing levels of CD11c and CD86 in nude mouse normal brain (Nu-NB) and three PDOX models. Unstained control is shown for each population (CTR). Right: quantification of CD11c^+^ and CD86^+^ cells in Mo-TAMs and BAMs across different tumor phenotypes and brain regions (n=3–6 from at least three different mouse brains each, one-way ANOVA, ***p<0.001, **p<0.01, *p<0.05). **(F)** Representative immunofluorescence pictures depicting CD11c staining of Iba1 cells in P8 PDOX tumor core. Scale bar: 20 μm. **(G)** Representative flow cytometry graphs showing activation of MHC-II (I-A/I-E epitope) in Mg-TAMs and Mo-TAMs in tumor core versus distant brain. Examples shown for P13 PDOX. **(H)** Representative immunofluorescence pictures depicting MHC-II and CD11c co-expression of Iba1 cells in P8 PDOX tumor core. Scale bar: 20 μm.

We next interrogated the functional properties of Mg-TAM subpopulations. Gene ontology analysis of genes activated in myeloid cells in PDOXs uncovered enrichment of terms associated with cell chemotaxis, cell adhesion and migration, and tumor-associated extracellular matrix proteins (Fig 1D). Enrichment analysis of gene signatures involved in migration, sensome, phagocytosis and antigen presentation (Table S6) revealed global enrichment in Mg-TAMs (CL2–6, Fig 4A, Fig S6A). A migration score based on genes associated with monocyte, glial and neutrophil cell migration (e.g. *Fn1, Cxcl13, Ccl3*) inferred increased migratory capacity during transition from Ho-Mg towards different phenotypic states of Mg-TAMs and in Mo-TAMs (Fig 4A, Fig S6A). We verified increased migratory ability of those cells seeding CD11b^+^ cells freshly isolated from PDOXs (P8, P13) or naïve brains in Boyden chambers (Fig 4B). Mg sensome genes (Table S6) showed a higher score in CL0, CL3 and CL5 Mg-TAMs compared to other Mg and non-Mg-TAM subsets (Fig 4A). Importantly, in CL3 and CL5, the sensome signature was driven by genes, such as *Cd74*, *Cd52, Cxcl16* and *Clec7a*, and not homeostatic Mg genes, which were highest in CL0 Ho-Mg (Fig S6A). CL3 and CL5 Mg-TAMs also showed increased expression of genes related to phagocytosis (e.g. *Trem2, Tyrobp, Axl)* and antigen presentation (*Itgax* (CD11c), *Igf1, CD86*) (Fig. 4A, Fig S6A), at similar levels as Mo-TAMs, but not BAMs. These signatures showed strong correlation with each other (Fig S6B). Indeed, Mg-TAMs isolated from P8 PDOXs, mostly represented by Mg-TAMs (Fig 2B), showed increased phagocytic abilities compared to CD11b+ cells (largely Ho-Mg) isolated from normal brains (Fig 4C–D). We also confirmed prominent CD11c and CD86 activation in Mg-TAMs, mainly in the tumor core of three PDOXs confirming antigen presentation cell (APC)-like features (Fig 4E). Increased expression levels of CD11c were also detected by immunohistochemistry in amoeboid Iba1^+^ cells in the cellular tumor, but not in ramified Mg in normal brain (Fig. 4F). CD11c and CD86 were also expressed by Mo-TAMs and BAMs, consistent with gene expression profiles (Fig S6C). We further confirmed activation of MHC-II expression (*H2-Eb1, H2-Ab1* and corresponding epitope I-A/I-E, Fig 4G–H, Fig S6A) in subsets of Mg-TAMs and Mo-TAMs when compared with normal brains. Subsets of Mg-TAMs and Mo-TAMs expressed also checkpoint inhibitors, such as *Cd274* (PD-L1), *Havcr2* (TIM-3) and *Pdcd1* (PD-1, Fig S6A,D), known to inhibit the phagocytic capacity of macrophages. Mg-TAMs also showed increased levels of *Sirpa* and *CD47*, maintaining the ‘‘do-not-eat-me’’ signal. Interestingly, astrocytic-like (CL4) and endothelial-like (CL6) Mg-TAMs showed less prominent phagocytic and antigen presentation scores compared to other Mg-TAMs (CL3, CL5). The functionality and localization of these subpopulations will need further investigation. Lastly, we did not detect increased expression of genes typically associated with macrophage immune suppression marker genes (e.g. *Arg1*; *Retnla*, *Il10*) in Mg-TAMs (Fig S4F).

Overall, these results suggest that specific subpopulations of Mg-TAMs display phagocytic and dendritic cell-like programs under tumorigenic conditions.

### Heterogeneous Mg-TAMs represent central components of GBM patient tumors

**Figure 5.**
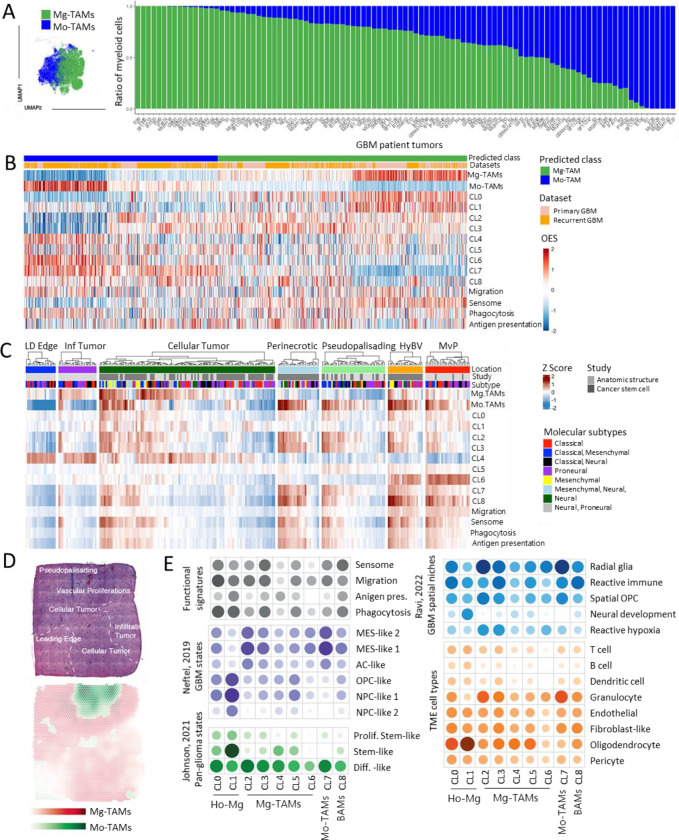
Transcriptomic states of myeloid cells in human GBM. **(A)** UMAP projection of myeloid subsets from GBM patient tumors. Cells are color coded for Mg-TAMs and Mo-TAMs ontogeny based on established gene signatures. Proportions of Mg-TAMs and Mo-TAMs are shown in individual GBM patient tumors. (**B**) Convergence of Mg and Mo to TAMs in primary and recurrent GBM patient tumors. Heatmap shows overexpression scores (OES) of signatures in scRNA-seq primary and recurrent GBMs from^8^ **(C)** Heatmap showing OES of signatures in different tumor locations from the IVY GAP bulk RNA-seq data. (LD edge: leading edge; Inf tumor: infiltrative tumor; HyBV: hyperplastic blood vessels in cellular tumor; MvP: microvascular proliferation). (**D**) Surface plots of spatial localization of myeloid cell signatures in GBM patient tumors. (**E**) Spatially weighted correlation analysis of the enrichment scores in myeloid transcriptomic states (CL0–8) linked to functional myeloid signatures, GBM tumor states, spatial TME niches and TME cell components.

To investigate the relevance of our findings in PDOXs, we next probed the composition of myeloid cells in publicly available scRNA-seq datasets of *IDH* wildtype GBM patient tumors by extracting the myeloid compartment from the GBMap, a curated scRNA-seq database of 240 patient tumors^32^, and by own analysis based on published datasets^8,33–35^. Due to the lack of normal brain Mg and blood Mo references, we applied robust gene signatures to assign TAM ontogeny (Fig 5A, Fig S7A–B, Table S6). Mg-TAMs constituted the main myeloid cell subset across the majority of GBMs. As reported previously^8^, recurrent GBMs generally showed increased proportions of Mo-TAMs, albeit with high interpatient differences. BAM signatures were rather weak and were not clearly segregated within a specific cluster (Fig S7A). The analysis of Mg-TAM gene signatures in human cells confirmed the presence of heterogeneous Mg subsets in human GBMs (Fig 5B). CL2, CL3 and CL5 signatures were robustly expressed by human Mg-TAMs (Fig 5B, Fig S7C). Although CL4 and CL6 signals appeared in general weaker than classical pro-tumorigenic signatures, subsets of Mg-TAMs showed high score for astrocytic-like (CL4) and endothelial-like (CL6) features (Fig S7C). We further confirmed adaptation of Mo to Mo-TAMs, which showed decreased levels of Mo markers concomitant with low expression levels of Mg genes. Such bilateral convergence of Mg and Mo was further confirmed in bulk RNA-seq profiles of Mg-TAMs and Mo-TAMs isolated from GBM patient tumors based on CD49d expression^36^ (Fig S7D). Similarly to mouse myeloid cells, both Mg-TAMs and Mo-TAMs showed activation of migration, phagocytosis and antigen presentation features (Fig 5B) and only a small fraction of Mg-TAMs and Mo-TAMs displayed cell cycle signatures (Fig S7A). We further confirmed the relevance of our findings in PDOXs by mapping mouse myeloid cells to the GBmap^32^ datasets corroborating the presence of Mg and Mo and their convergence towards tumor-specific programs (Fig. S7E). Altogether, these data show the relevance of distinct Mg substates and the robustness of their signatures identified in PDOXs in GBM patient tumors.

### Mg-TAMs in GBM patients are spatially distributed across different tumor niches

To investigate the spatial distribution of GBM-associated myeloid states, we assessed myeloid signatures in the bulk RNA-seq of GBM tumor niches (Ivy Glioblastoma Atlas Project dataset^37^, Fig. 5C, Fig. S8A). In general, Mg signatures were evident in infiltrative and cellular tumor, hyperplastic blood vessels (HyBV) and microvascular proliferation (MvP) zones, although CL2 and CL3 Mg-TAMs also scored high in perinecrotic and pseudopalisading areas. Astrocytic-like CL4 and endothelial-like CL6 showed high signals in leading edge/infiltrating/cellular tumor and HyBV/MvP niches, respectively. Importantly, these signatures are likely biased by the bulk RNA-seq signal including high proportions of reactive astrocytes and endothelial cells in distinct niches. Mo-TAM (CL7) and BAM (CL8) signatures were particularly evident in niches displaying blood-brain barrier leakage, including HyBV, MvP, perinecrotic and pseudopalisading areas, consistent with our findings in PDOXs and GL261. Migration, sensome, phagocytosis and antigen presentation signatures were expressed in the cellular tumor, perinecrotic zone, hyperplastic blood vessels and microvascular proliferation zones, and were particularly high in the tumors with high abundance of Mg and Mo. These functional signatures were less pronounced in the leading edge and infiltrative zone, confirming education of Mg in close proximity to GBM cells. Interestingly, these signatures were relatively lower in pseudopalisading tumor zones regardless high abundance of Mo-TAMs, suggesting a potential role of severe hypoxia in inhibition of myeloid cell functions in this niche. The high score for Mg/Mo signatures was particularly evident for tumors with mesenchymal components, confirming previous studies^12^.

We further confirmed differential distribution of myeloid states in spatially resolved transcriptomic profiles of GBM patient tumors^38^. Again, Mg-TAMs were highly abundant in the infiltrating and cellular tumor, whereas Mo-TAMs were enriched in the pseudopalisading and vascular proliferation areas (Fig 5D, Fig S8B–C). Spatially weighted correlation analysis and spatial gene set enrichment analysis confirmed the co-localization of Mg-TAM CL3 and CL5 with signatures of phagocytosis and antigen presentation (Fig 5E). Interestingly, Mg-TAMs and Mo-TAMs co-localized with MES-like and AC-like GBM states, corresponding to the “Differentiated-like pan-glioma state”. Ho-Mg states were more abundant in close proximity to OPC-like and NPC-like GBM states, equivalent to the “Stem-like pan-glioma states”. This was corroborated with the distribution across spatial GBM niches. All myeloid cells were present in different GBM niches with high tumor content (Radial glia, Reactive immune, Spatial OPC niches). Ho-Mg (CL1) were particularly abundant in ‘Neural development’ niche at the tumor edge, whereas CL2 and CL3 Mg-TAMs were associated with ‘Reactive hypoxia’ niches. Analysis of bulk RNA-seq CGGA patient tumor profiles ^39^ revealed shorter survival of patients with high score for both Mg-TAM CL4, Mo-TAMs and BAMs, but not Ho-Mg in classical GBMs (Fig S8D). No difference in survival was observed for proneural and mesenchymal tumors. Taken together, these data confirm the transcriptomic heterogeneity of Mg-TAMs in GBM patient tumors associated with different functional features across specific tumor niches.

### TMZ treatment leads to transcriptomic adaptation of GBM cells and adjacent TME

**Figure 6.**
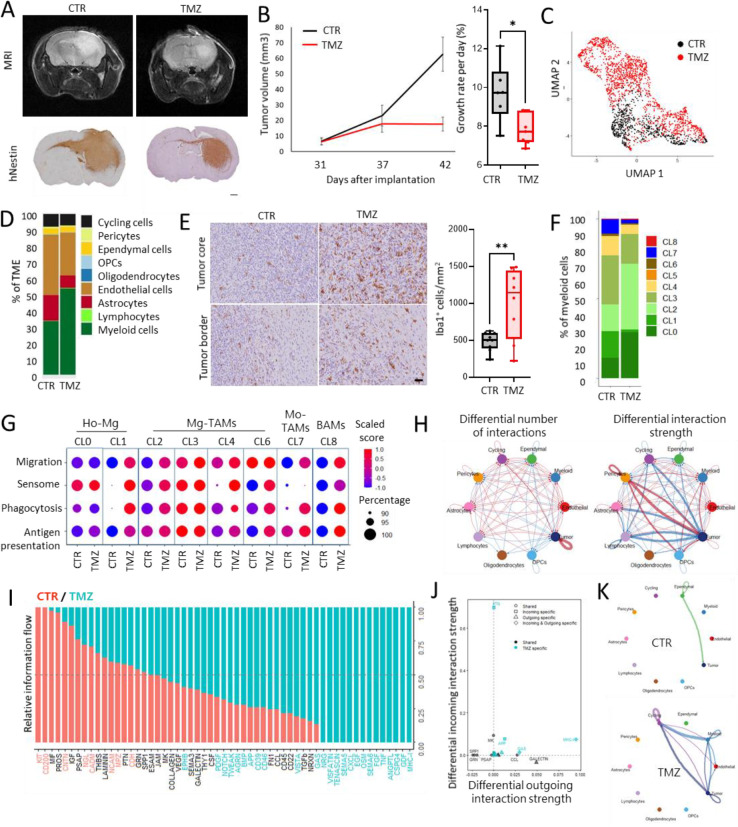
Transcriptomic adaptation of GBM tumor cells and TME upon TMZ treatment. **(A)** Representative MRI images and human-specific Nestin staining showing tumor growth in control P3 PDOXs (CTR) and TMZ-treated mice (TMZ). Scale bar = 100μm. **(B)** MRI-based assessment of tumor progression over time. Tumor growth rate was calculated during the entire study (day 42 vs day 31, n = 6–7, *p<0.05, two-tailed Student’s t-test). **(C)** UMAP projection showing the overall gene expression relationship between TMZ-treated and CTR GBM tumor cells; **(D)** Proportions of cell types in TME in CTR and TMZ-treated P3 PDOX; **(E)** Examples of Iba1 staining and quantification in CTR and TMZ-treated P3 PDOXs. Tumor cores are highlighted. Scale bar: 50 μm. (**p<0.01, two-tailed Student’s t-test) **(F)** Proportions of cells assigned to nine clusters of myeloid cells in CTR and TMZ-treated P3 PDOX; Clusters 0–1: homeostatic Mg (Ho-Mg), Clusters 2–6: Mg-TAMs, Cluster 7: Mo-TAMs, Cluster 8: BAMs. (**G**) Functional signature score per myeloid cluster in CTR and TMZ conditions. (H) CellChat-based differential number of interactions and interaction strength of the inferred cell–cell communication networks between cell types in CTR and TMZ PDOXs. Red or blue colored edges represent increased or decreased signaling in TMZ-treated tumors. (**I**) Relative information flow from cell–cell interaction analysis. Receptor-ligand pathways with blue text are significantly enriched in TMZ-treated cells, and pathways with red text are significantly enriched in CTR cells. (**J**) Signaling changes of myeloid cells in TMZ vs CTR conditions. (**K**) Interactions related to the Gas6 pathway in CTR and TMZ conditions.

Lastly, we aimed to assess the adaptation of tumor cells and TME upon treatment. For this, we administered TMZ to P3 PDOXs representing *MGMT* promoter-methylated GBM (Fig S9A). Tumor growth was validated by MRI and mice were treated 5 times a week at the clinically relevant TMZ dose for 10 days (total 8 doses received). Tumors were resected shortly after the last TMZ dose. Prolonged TMZ treatment led to decreased tumor growth (Fig 6A–B). ScRNA-seq analysis of isolated tumor cells revealed transcriptomic changes linked to survival mechanisms such as regulation of p53-associated signal transduction, apoptosis, cell death and cellular component organization (Fig 6C, Fig S9B–C), suggesting activation of resistance mechanisms in surviving tumor cells. Assessment of GBM cellular subtypes revealed an increased proportion of MES-like states in line with observations made in GBM patients^11,12^. Corresponding scRNA-seq analysis of the TME revealed changes in the proportions of cell populations (Fig 6D). We observed an increased ratio of myeloid cells upon TMZ and a relative decrease of ECs and astrocytes. Indeed, TMZ-treated tumors contained more Iba1^+^ myeloid cells in the tumor core (Fig 6E). The analysis of DEGs between TMZ-treated and control tumors revealed transcriptomic changes in myeloid cells and ECs, but not in astrocytes (Table S7, Fig S9D). Upon treatment, myeloid cells enhanced the expression levels of genes associated with inflammatory responses, such as migration, chemotaxis, and gliogenesis (e.g., *Cxcl13, Cx3Cr1, Csf1r*). Adaptation was visible at the level of regulation of translation (e.g. *Rps15*, *Rpl32*, *Rpl23*), endocytosis (e.g. *Apoe, Lrp1*), cholesterol homeostasis (e.g. *Abca1, Abcg2*) and actin cytoskeleton (e.g*. Fscn1, Coro1a*). This correlated with decreased levels of TAM markers promoting tumor growth, such as *Igfbp7* and *Gng5*. Although the heterogeneity of the myeloid compartment shifted to a higher ratio of CL2 Mg-TAMs (Fig 6F), the activation of functional signatures was observed in the majority of Mg-TAM clusters, Mo-TAMs and BAMs (Fig 6G). In parallel, ECs deregulated genes associated with cell development and death, extracellular space, regulation of chemokine and cytokine production and acetylcholine receptor activity (e.g. up: *Ly6a/c1/e, H2.D1, Timp3, Cxcl12; down: Ctsd, Ctss*) as well as regulation of actin cytoskeleton and protein localization (Fig S9D). These changes led to significant adaptation of the molecular crosstalk within the GBM ecosystem (Fig 6H–I). Ligand-receptor interactions were particularly affected in myeloid cells, which showed increased outgoing interactions after TMZ treatment, specifically those associated with MHC-I, Gas6 (Growth arrest-specific 6), and App (Amyloid-beta precursor protein) pathways (Fig 6J–K, Fig S9F–G). Interestingly Gas6 produced by myeloid and tumor cells was predicted to interact with the receptor Mertk in ECs and cycling cells. Gas6 of myeloid cells was predicted to interact with Tyro3 in tumor cells, while Gas6 produced by tumor cells may interact with Axl in myeloid cells.

Taken together, we showed that induction of cell death resistance mechanism in tumor cells upon TMZ treatment is associated with transcriptomic changes in TME components, including inflammatory responses of Mg-TAMs.

## DISCUSSION

Various cellular components within the TME are critical for the establishment of an immunosuppressive environment that facilitates GBM growth, progression, and treatment resistance. Using unbiased scRNA-seq analysis, we surveyed the TME of nine GBM PDOXs and compared it with the TME of the GL261 mouse glioma model and human GBM tumors. We show that GBM PDOXs present diverse cell types in the TME similar to those reported in human GBM^12,23,33^. We provide evidence that human tumor cells instruct the TME subpopulations in PDOX models towards GBM-associated phenotypic states, thus unlocking their relevance as preclinical models to investigating the modulation of the GBM ecosystem upon treatment and testing novel therapies against tumor and TME cells. We further uncover diverse cellular and molecular specificities of the GBM-associated myeloid compartment. Specifically, we found that resident Mg represent the main myeloid cell population in PDOXs and human GBMs, while peripheral-derived Mo mostly infiltrate the brain at sites of the blood-brain barrier disruption. Mg exhibit molecular plasticity toward diverse GBM-associated states reflecting intratumoral heterogeneity. Notably, we detected reactive dendritic cell-like gene expression programs in a large subset of GBM-educated Mg. These cells show high activation of the Mg sensome followed by increased phagocytic and antigen presentation capacity. We show that Mg states are differentially distributed across spatial GBM niches, where they co-localize with varying TME components and GBM phenotypic states.

Studies of the differences between TAMs of different origin have been confounded by a lack of specific markers to separately purify these cell types within GBMs. By applying robust transcriptomic gene signatures, we found that Mg-TAMs represent an important fraction of myeloid cells in PDOXs, syngeneic models and GBM patients, confirming reports in mouse chimeras^40^ and human GBMs^3,8,41^. Discrepancies in the field may arise from varying sampling strategies and marker selection. As Mg-TAMs downregulate classical homeostatic genes while activating macrophage markers, Mg/Mo discrimination based solely on the expression of selected homeostatic and/or macrophage markers may lead to biased misidentification of Mg-TAM subpopulations. In both human GBMs and preclinical models, Mg-TAMs are abundant in the cellular tumor and invasive niche, whereas Mo-TAMs are confined mostly to perinecrotic, and hypoxic areas with the leaky blood-brain barrier. It is in accordance with the reports showing diffuse presence of Mg in gliomas in contrast to brain metastases, where Mg appears to be often confined to the tumor border areas^3^. Mo are particularly abundant in the GL261 syngeneic model, which does not recapitulate well the cellular tumor and infiltrative tumor zone. It remains to be seen whether Mo-TAMs are active players or a bypass product of the blood-brain barrier leakage. Although we confirm a higher proportion of Mo-TAMs in a subgroup of recurrent human GBMs^8,11^, we highlight high inter-patient differences and potential sampling bias linked to GBM niches and tissue isolation strategy, as observed in preclinical models. Analysis of the myeloid compartment in longitudinal PDOXs derived from matched primary and recurrent patient tumors did not reveal inherent changes in the TME composition. We rather detected patient-specific profiles that were retained in recurrent models. This is consistent with studies conducted in genetically-engineered models of gliomas, where Mg/Mo ratios are model and not treatment dependent^42^. A larger cohort will be needed to further interrogate the correlation between TME composition and GBM molecular features. Interestingly, we observed decreased survival of patients with high Mg-TAM and Mo-TAM signatures within classical GBMs, but not mesenchymal and proneural GBMs. We previously showed that the TAM signature associated with Mo-TAMs correlate with decreased survival in gliomas in general^20^. This is consistent with the co-localization to hypoxic and perinecrotic niches, two factors associated with poorer survival.

Despite their distinct developmental origin and intrinsic transcriptional networks, myeloid cells are known to share signatures of tumor education, although specific functions of Mo- and Mg-TAMs have also been suggested^37,43^. The high abundance of Mg in our PDOXs allowed us to further discriminate distinct phenotypic states of Mg-TAMs, which is more challenging in the syngeneic models^7,20^. We show that Mg-TAM activation occurs in PDOXs generated in nude mice, which show strongly reduced and hypo-responsive T cells^44^, suggesting that the myeloid-T cell crosstalk is not a prerequisite for TAM activation. Since nude mice still possess B and NK cells as their main lymphocytic subpopulations, it remains to be seen whether the loss of T cells is compensated by other available lymphocytes. Of note, in GBM patients naïve T cells are sequestrated in the bone marrow, which contributes to a very low abundance of T cells in the tumor^45^. As a dominant myeloid population, Mg underwent pronounced activation in PDOXs, whereas Mo dominated GL261 tumors, where they acquired highly immunosuppressive states. These observations are in line with results obtained during GL261 progression, where early stages of tumor development are dominated by CD11c^+^ Mg invading the tumor, followed by recruitment of CD11c^+^ Mo-derived dendritic cells^6^.

We show that Mg-TAM subpopulations range from homeostatic to GBM-educated states. We have identified two states corresponding to Ho-Mg (CL0&1) and five Mg-TAM states (CL2–6). Importantly, these states were detected across PDOXs and patient GBMs with different genetic backgrounds, highlighting pan-GBM significance and intra-tumoral heterogeneity. Ho-Mg states are present within several GBM tumor niches representing cellular tumor and invasive edge, suggesting ongoing education in the close proximity by the tumor cells. Mg-TAM states share classical GBM education, including decreased levels of homeostatic genes, co-expression of pro- and anti-inflammatory molecules and increased levels of markers classically associated with pro-tumoral macrophages, such as high levels of CD45 and CCR2. Importantly, Mg states display also discrete transcriptomic features and gene regulatory networks. CL3 and CL5 show particularly high features of Mg sensome, but also phagocytosis and antigen presentation at similar levels to Mo-TAMs, while CL2 appears as a transitory state from Ho-Mg to Mg-TAMs. CL5 Mg-TAMs display in addition high transcriptomic activity, previously reported by Ochocka et al.^7^ Importantly, increased sensome activity is driven by a limited set of specific genes (*Cd74*, *Clec7a, Cxcl16*), while key homeostatic genes responsible for general sensing changes in brain are downregulated, confirming reduced capacity to sense changes in the TME caused by GBM. While all Mg states are detected across several spatial GBM niches, CL2 and CL3 states are particularly abundant in ‘Reactive hypoxia’ co-localizing with MES-like GBM cells, whereas ‘Neural development’ niche is predominantly enriched with CL1 Ho-Mg in close proximity to oligodendrocytes and OPC/NPC-like GBM states. We functionally confirmed that phagocytosis is enhanced in the cellular tumor compared to adjacent normal brain. Our data is in line with previous reports showing the phagocytic activity of GBM-associated Mg^46,47^. Although the functional implications of phagocytic Mg in GBM are still elusive, emerging data suggest both a pro- and anti-tumoral effect. For instance, phagocytic Mg were shown to populate necrotic tumor zones and aid in the clearance of debris to enhance GBM cell invasion^47^. Increased phagocytosis was reported to enhance antigen cross-presentation towards more efficient T cell priming upon combined TMZ and anti-CD47 treatment^48^.

Dendritic-like Mg have been also reported in disease-associated microglia (DAM) of Alzheimer’s disease^49–51^, amyotrophic lateral sclerosis and multiple sclerosis^52^. Mg-TAMs and DAM appear to activate similar programs including decreased homeostatic genes, classical activation markers (e.g. *Spp1*, *Il1b*), the phagocytic (e.g. *Apoe, Trem2*, *Tyrobp*) and APC (e.g. *Itgax* and *Igf1*) signatures. This suggests that phagocytic and APC-specific transcriptional programs are associated with Mg detecting damage within the CNS^28^ as well as recognizing and clearing pathogenic factors, such as neoplastic cells in GBM and β-amyloid aggregates in AD, but not along acute inflammatory processes, where Mg rapidly restore and maintain the homeostatic neuronal network^25^. Mg and brain resident macrophages are also known to act as competent APCs during CNS infections, potentially involved in activation of infiltrating T cells^53^. Intriguingly, we identified additional clusters within Mg-TAMs expressing astrocytic (CL4) and endothelial (CL6) markers. These phenotypic states, despite high score for migration, showed lower levels of sensome, phagocytosis and antigen presentation, suggesting other functions. As bulk RNA-seq and spatial transcriptomics do not present sufficient resolution to deconvolute these signals from bona fide reactive astrocytes and endothelium, their localization in the tumor needs further investigations in the future. The expression of astrocytic genes such as *Gfap* and *Serpina3n* has been previously reported in Mg in mouse models of injury and autoimmune encephalomyelitis where it is speculated that they might suppress pro-inflammatory pathways^54^. GFAP has also been detected in a subset of circulating Mo in brain tumor patients^55^.

Lastly, we demonstrate the utility of PDOX models in understanding adaptation of the GBM ecosystem upon treatment. Although several studies suggested increased abundance of TAMs at recurrence^8,11^, a direct analysis upon treatment is not possible in human tumors. By applying TMZ to a *MGMT*-methylated GBM PDOX model, we identified both tumor cell and TME remodeling. Increased apoptotic signaling and pronounced MES-like GBM state was observed together with increased Mg migration toward the tumor. Mg-TAMs in treated tumors displayed re-polarization towards more pro-inflammatory states with increased phagocytosis and antigen presentation scores. These changes led to a altered molecular crosstalk with tumor cells, e.g. through activation of the Gas6 signaling across several cell types. Although further investigation of the key axes in such complex crosstalk is needed, we speculate that Gas6 expressed by apoptotic cells activated efferocytosis, a well-known function of Mg in the brain, which allows the clearance of damaged apoptotic cells and prevents inflammation^56^. Further studies with a larger cohort of treated PDOXs will be needed to fully understand the adaptation of GBM cells with different molecular backgrounds and their adjacent TME. As direct effects of the treatment on Mg have also been reported^57^, further studies are needed to dissect molecular events upon treatment leading to changes in the cellular network.

Research efforts aimed at deciphering the functional heterogeneity of TAMs may contribute to the development of novel immune therapeutic approaches in GBM patients. It remains to be investigated how TAMs could be reprogrammed against GBM cells^58^. TAMs showed enhanced phagocytic ability following CD47 blockade^46,59,60^, an effect that was more pronounced in combination with TMZ^48^. These results suggest that the phagocytic capacity of Mg-TAMs appears to be pervasive and may require fine-tuning in the context of therapeutic reprogramming. Further functional characterization is needed to harness their anti-tumor potential, for instance by addressing their capacity to recruit lymphocytes and their phagocytic ability against tumor cells.

Taken together, we show key adaptation of brain resident cells to TME niches in GBM. We unravel heterogeneous Mg states, which reside in different spatial niches. In-depth characterization of specific signatures of the TME and their adaptation upon standard-of-care treatment will pave the way towards rational design of targeted treatment strategies. The use of PDOX avatars holds promise for the functional assessment of the plastic GBM ecosystem upon treatment and for testing novel therapeutics, including modalities targeting GBM-educated myeloid cells.

## MATERIALS AND METHODS

### Clinical GBM samples and PDOXs

We collected GBM tissues from the Centre Hospitalier de Luxembourg (CHL, Neurosurgical Department) or the Haukeland University Hospital (Bergen, Norway) from patients who had given their informed consent, and with approval from the local ethics committees (National Committee for Ethics in Research Luxembourg and local ethics committee Haukeland University Hospital in Bergen). PDOX derivation was previously described^17,18,61^ (Table S1). For this study, tumor organoids were implanted (6 per brain) into the right frontal cortex of nude mice (athymic nude mice, Charles River Laboratories, France). Animals were sacrificed at the endpoint defined in the scoresheet. Mice were deeply anaesthetized with a mixture of ketamine (100 mg/kg) and xylazine (10 mg/kg) and transcardially perfused with ice-cold PBS.

For TMZ treatment, P3 PDOXs were evaluated daily. Tumor growth was monitored by MRI (3T MRI system, MR Solutions). At day 30, mice were randomized into 2 groups: from day 33 control PDOXs were administered with NaCl 0.9% + 10% DMSO, treatment group received 40mg/kg TMZ in NaCl 0.9% + 10% DMSO corresponding to 120 mg/m^2^ in human. Treatment was administered by oral gavage 5x per week with a total of 8 doses, where the last treatment dose was given shortly before the endpoint (Figure S9A). Tumor growth was followed by MRI at day 37 and 42 and quantified as described in^61^. Statistical difference was assessed with two-tailed Student’s t-test. Experiments were performed in accordance with the regulations of the European Directive on animal experimentation (2010/63/EU) and were approved by the Animal Welfare Structure of the Luxembourg Institute of Health and by the Luxembourg Ministries of Agriculture and of Health (LRNO-2014–01, LUPA2019/93 and LRNO-2016–01).

### scRNA-seq in PDOXs

We extracted tumor tissues from mouse brains and dissociated with the MACS Neural Dissociation kit (Miltenyi Biotec) according to the manufacturer’s instructions. Single cells were purified with the Myelin Removal Beads II kit (MACS Miltenyi Biotec) as described before^61^. To separate human GBM cells from mouse TME, PDOX-derived cells were FACS-sorted (P8, nude control brain)^62^ or MACS-purified (remaining PDOXs) with Mouse Cell Depletion kit (Miltenyi Biotec) ^61^. Except for tissue dissociation, all steps were handled on ice. Mouse-derived TME was processed via Drop-seq. See Supplementary material for human tumor cells.

Drop-seq and data preprocessing were performed as previously described^17,20^. Briefly, scRNA-seq analysis was performed in R (v4.1.1) with the Seurat package (v4.0.5) ^63^. Human and mouse cells were separated by mapping the scRNA-seq reads to human g38 and mouse mm10 reference genomes. The distributions of UMI counts and features expressed allowed for clear cell separation. Mouse TME samples were merged with published DROP-seq dataset of GL261 TME^20^. QC thresholds were empirically applied per sample, only genes expressed in at least 5 cells, cells expressing at least 200 features and cells with 30% or less mitochondrial reads were selected. Potential doublets were predicted and removed using DoubletFinder (v2.0.3)^64^ Counts were normalized using Seurat-based ‘NormalizeData’ function, batch correction was done by Harmony (v0.1.0) ^65^. Clustering was done on harmony embeddings using the default parameters of the Seurat package. Dimensionality reduction was done using the Uniform Manifold Approximation and Projection (UMAP) of the Seurat package. Differential expression analysis was done using Wilcoxon rank sum test, false discovery rate (FDR) was calculated using the Benjamini-Hochberg method. Cell clusters were identified based on expression of known marker genes and differentially expressed genes (DEGs) were determined by the ‘FindAllMarkers’ function. The myeloid cluster was extracted using the ‘Subset’ function. Gene ontology analysis was performed by METASCAPE (https://metascape.org/). Single cell trajectory inference analyses were done with the R package Monocle v2 and v3 using default parameters^66^. Z-score of genes was calculated by subtracting the mean of expression from the raw expression of each gene and normalization by the corresponding standard deviation. Gene expression was displayed as heatmap of z-scores. Single-cell gene set signature scores were calculated using the Seurat ‘AddModuleScore’^67^. Identification of master transcriptional regulators was done using normalized counts from the subsetted myeloid cells. Gene regulatory network inference was done according to the standard SCENIC workflow ^31^. To deconvolute cellular signals, we applied a consensus-independent component analysis (ICA) method, previously developed in ^17,62,68^. Typically, 20–30 components are enough for single-cell data, however here we investigated ICA with up to 256 components. Each deconvolution was performed 20 times to acquire stability. The method is available in the Bioconductor package *consICA*.

### Reference Based Mapping

Myeloid cells were identified and extracted from publicly available GL261 scRNA-seq datasets by expression of key myeloid cell markers (“*Itgam*”,”*P2ry12*”,”*Csf1r*”,”*Tgfbi*”, “*Ptprc*”, “*Hexb*”,”*Mrc1*”, “*Ly6c2*”) ^7,8,20^. Dendritic cells (DC) were excluded by identifying clusters expressing key DC markers reported in Pombo et al.. The Ochocka et al. dataset was used as the reference. Other datasets were projected onto the reference UMAP structure. The reference principal component analysis (PCA) space was computed using 2000 most variable genes, and the first 30 PCs were used to calculate the UMAP model. Next, we determined the common features of the reference and each of the query datasets by Seurat’s ‘FindTransferAnchors’ function with reduction method ‘pcaproject’ and parameter ‘dims = 1:50’. Lastly, we called ‘MapQuery()’ to transfer cell type labels and project the query data onto the UMAP structure of the reference.

### Cell-to-cell communications analyses

Cell-to-cell communications were inferred using CellChat(v1.6.0)^69^. Human genes in the GBM tumor cells from P3CTR and P3TMZ PDOXs were converted to mouse homologs using ‘convert_human_to_mouse_symbols’ function in nichenetr package (v1.1.1)^70^ and merged with the TME matrix from the corresponding PDOX. Crosstalk inference analyses was done using the ‘CellChatDB.mouse’ database on each of the conditions before they were merged for comparison. Comparison of the 2 conditions was done as indicated in the CellChat tutorial on comparison of “multiple datasets with slightly different cell type compositions”.

### Human GBM scRNA-seq analyses

Analysis of TME in human GBM was performed taking advantage of using the Darmanis et al., dataset ^23^. Analysis of myeloid cells was performed using publicly available 10XGenomics GBM datasets^8,33–35^. Data were obtained as preprocessed gene expression matrices (DEMs) from a total of 36 IDH wild type tumors including newly diagnosed (n = 27) and recurrent (n = 9) GBMs. Each dataset was analyzed separately to extract myeloid cells. Myeloid cells were identified and extracted as follows: (1) For Friedrich’s dataset, cells were identified according to author’s annotation; (2) For Wang’s, Johnson’s and Pombo-Antunes’ datasets, we used an approach that combines overexpression (OE) and clustering analysis. First, genes with zero count in all cells were filtered out and ‘NormalizeData’ function was applied to LogNormalize each cell with a scale factor of 10,000. UMAP was used to visualize the cells and clusters on 2 dimensions. Cluster identity was determined according to over-representation of a cell-type within the cluster as called by OE analysis. Myeloid cells were extracted for further analysis. Overall, 51,302 myeloid cells were extracted and united into one Seurat object. Genes with zero counts in all cells were removed, and cells were log-normalized with a scale of 10,000. Harmony (v0.1.0) package was used to remove variation due to batch effects. All PCs were used and *theta* was set to 1. Harmony embedding was used for clustering analysis.

A subset of myeloid cells including Mono, TAM-BDM and TAM-MG from the GBmap Seurat object’s ‘annotation_level_3’ were scored using our Human Mo and Human Mg gene lists. Myeloid cells were assigned to either Mo.TAMs or Mg.TAMs base on the highest single-cell gene set signature scores between Mo.TAMs or Mg.TAMs gene sets for each cell.

Heatmap showing overexpression scores (OES) of cell type markers, functional signatures as well as cluster markers in Pombo Dataset was generated using pHeatmap (v1.0.12) [Kolde R (2019). OES were calculated using Seurat’s ‘addModuleScore’ function. Cells (columns) were ordered according hierarchical clustering analysis based on OES of Mo.TAM and Mg.TAM gene lists. To generate the heatmap of OES of signatures in the IVY-GAP dataset, OES of the gene lists were calculated using the normalized z-score as done in Jerby-Arnon et al.^71^ OES table was then split according to the tumor location and heatmaps of OES were generated for each tumor location using ComplexHeatmap (v2.12.1) ^72^.

Where applicable, mouse gene lists were converted to human homologs automatically in R using capital letter (*toupper*) or the ‘getLDS’ function in biomaRt package^73^ (Durinck et al., 2009). Only genes with human homologs available in “http://www.informatics.jax.org/downloads/reports/HOM_MouseHumanSequence.rpt“.

### Spatial transcriptomics

Spatial transcriptomic profiles of GBM patient tumors were obtained as described in^38^. For spatial data analysis, we acquired the spatially resolved RNA-seq datasets using the SPATAData package (https://github.com/theMILOlab/SPATAData). For annotation of the scRNA-seq dataset to spatial transcriptomic data, we humanized the genes using the mice2human database (http://www.informatics.jax.org/downloads/reports/HOM_MouseHumanSequence.rpt) and rejected all genes that failed to map to the human transcriptome. Spatially correlation analysis was performed by either a spatial Lag model or a Canonical Correlation Analysis (CCA) using the ‘runSpatialRegression’ function from the SPATAwrappers package. Cell type deconvolution of each spot was performed by Robust Cell Type Decomposition (RCTD) a well-validated toolbox. The deconvolution was performed by the SPATAwrapper (https://github.com/heilandd-/SPATAwrappers) package using the function runRCTD. Visualization of surface plots or correlation analysis was performed by the SPATA2 toolbox.

### Reference Mapping to GBMap Dataset

To map the scRNA-seq data set to the human GBMap reference dataset, we humanized the genes using the mice2human database (http://www.informatics.jax.org/downloads/reports/HOM_MouseHumanSequence.rpt) and rejected all genes that failed to map to the human transcriptome. Next, we used an optimized version of azimuth (‘modified_azimuth.R’) to map the query scRNA-seq dataset to the GBMap ^32^ as described recently. The mapping results were visualized in the ref.umap.

### Immunohistochemistry

The regular histological analysis of PDOX models (H&E, human Nestin/Vimentin, mouse CD31) has been performed as described previously^17,18^. Antibodies are listed in Table S2. Iba1 staining was performed on coronal 4 – 8 μm sections from paraffin-embedded brains. Sections were incubated 30 min at 95°C in retrieval solution (Dako). Primary antibodies were incubated overnight at 4°C or 3 hours at room temperature, followed by 30 min incubation with secondary antibodies. Signal was developed with the Envision+ System/HRP Kit in 5–20 min (K4007, Agilent/Dako). Iba1^+^ cells were quantified based on ImageJ plugin ^74^. For immunofluorescence, brains were perfused and post-fixed with 4% paraformaldehyde/sucrose for 48 hours. 4 – 12 μm coronal sections were permeabilised with PBS with 1.5% Triton X-100, blocked with 5% BSA and incubated with the primary antibodies. Secondary antibodies were incubated for 2h. Alternatively, an Opal 3-Plex Manual Detection Kit (Akoya Biosciences) was used following manufacturer guidelines. Cell nuclei were counterstained with Hoechst (1 mg/ml; Sigma). Sections were mounted on glass slides cover slipped using Fluoromount^™^ Aqueous Mounting Medium (Sigma). Images were obtained using a Nikon Ni-E or Zeiss LSM880 confocal microscopy. Z-stacks were done with 0.5μm steps in Z direction, with a XY resolution of 1.024 × 1.024 pixels.

### Multicolor flow cytometry

Animals were perfused with ice-cold PBS. PDOX brains were dissected into separate zones when specified: tumor core (cellular tumor, including pseudopalisading/hypoxic zone if present), invasive zone (corpus callosum and top left hemisphere, P3) and distant zone (left hemisphere, bottom hemisphere, P3 & P13). P8 PDOX was not dissected, due to its very invasive nature. PDOX tumors and control mouse brains were dissociated with MACS Neural Tissue Dissociation Kit (P) (Miltenyi) following the manufacturers’ instructions. Single cells were resuspended in ice-cold HBSS, 2% FBS, 10mM HEPES buffer (100 μl/test) flow buffer. Fc receptors were blocked with CD16/CD32 antibody for 30 min at 4°C. Cells were incubated with the appropriate pre-conjugated antibodies for 30 min at 4°C in the dark (Table S2). Non-viable cells were stained with Hoechst (0.1μg/ml, Sigma). Data acquisition was performed at 4°C on a FACS Aria^™^ SORP cytometer (BD Biosciences) fitted with a 632 nm (30 mW) red laser, a 355 (60 mW) UV laser, a 405 nm (50 mW) violet laser and a 488 nm (100 mW). Data were analyzed with FlowJo software (version 10.8.1).

### CD11b^+^ myeloid cell isolation and functional assays

Mice were perfused with ice-cold PBS. Tumor tissue was dissected from mouse brains and dissociated with the MACS Neural Dissociation kit (Miltenyi Biotec). Myeloid cells were enriched using CD11b^+^ beads (MACS Miltenyi Biotec). *Ex vivo* migratory abilities were assessed using 8 μm pore size Boyden chambers (ThinCert cell culture inserts, Greiner), fitting into 24-well plate. 100,000 cells were seeded in the upper chambers in DMEM-F12 medium. Upon 48 hours, cells were fixed in 4% PFA for 15 minutes and stained with DAPI for 15 minutes. Migratory cells were quantified by counting the number of cells on the lower side of the membrane under light microscope with a 20x magnifying objective (5 fields/membrane). Experiments were conducted in 3 biological replicates (each with 2 technical replicates). The data was normalized according to the proliferation index and is represented as percentage of cells that migrated relative to the initial number of cells.

*Ex vivo* phagocytic abilities were measured using the pHrodo Red E.coli bioparticles (Essen Bioscience, MI USA). 100,000 CD11b^+^ freshly isolated cells were plated into 96 well-plates in 100 μl and left for 2h to adhere. pHrodo Red E.coli bioparticles were added at 10 μg/ml and the plates were transferred into the Incucyte ZOOM (Essen Bioscience, MI USA) platform. 4 images/well from at least 3 technical replicates were taken every hour for a duration of 48h. Red fluorescence signal was quantified applying a mask and the parameter red object area was extracted for data analysis and visualization. For flow cytometry, Percoll-purified freshly isolated myeloid cells were incubated with 1 ug of pHrodo^™^ Red E. coli BioParticles^™^ Conjugate for Phagocytosis (Invitrogen) at 37°C for 1h followed by multicolor cell membrane marker staining at 4°C. Data acquisition was performed on NovoCyte Quanteon Flow Cytometer (Agilent) fitted with a 405nm Violet laser, a 488 nm blue laser, a 561 nm yellow/green laser and a 637 nm red laser.

### Statistical analyses

Statistical analysis details for each experiment are reported in the figure legends. In box plots, the box limits indicate the 25th and 75th percentiles and center lines show the medians; whiskers represent the minimal and maximal observed values. All data points are represented by dots.

## Supplementary Material

Supplement 1

## Data Availability

PDOX models are available via PDCMfinder (www.cancermodels.org) and are part of the EuroPDX consortium collection (www.europdx.eu). The scRNA-seq data from the PDOX models have been deposited in the Gene Expression Omnibus repository (www.ncbi.nlm.nih.gov/geo/) with the accession number GSE226468 (mouse TME) and GSE128195 (human tumor cells).

## KEY POINTS

- GBM-educated tumor microenvironment is faithfully recapitulated and modulated in PDOX models
- Microglia represent an essential myeloid cell population in the GBM microenvironment
- GBM-educated microglia acquire heterogeneous transcriptomic states across distinct tumor niches
- GBM-educated microglia subsets display phagocytic and dendritic cell-like gene expression programs, which are modulated upon TMZ treatment

## IMPORTANCE OF THE STUDY

This manuscript addresses tumor-immune interactions in GBM, focusing on the molecular changes of the myeloid compartment. We find that myeloid cells, the most abundant immune cell population in brain tumors, undergo the most prominent transcriptional adaptation in the TME. Resident microglia represent the main myeloid cell population in the cellular tumor, while peripheral-derived myeloid cells appear to infiltrate the brain at sites of blood-brain barrier disruption. We identify reactive dendritic cell-like gene expression programs associated with enhanced phagocytic and antigen-presentation features in GBM-educated microglia subsets that might be harnessed for novel immunotherapeutic approaches. Overall, PDOX models faithfully recapitulate the major components of the GBM-educated TME and allow assessment of phenotypic changes in the GBM ecosystem upon treatment.
