# Supplementary material for: Glioblastoma-instructed microglia transition to heterogeneous phenotypic states with phagocytic and dendritic cell-like features in patient tumors and patient-derived orthotopic xenografts
Source: bioRxiv. 2023 Dec 12:2023.03.05.531162. Originally published 2023 Mar 6. Preprint. [Version 2] doi: 10.1101/2023.03.05.531162 (PMC10028830; doi:10.1101/2023.03.05.531162)
Supplement: Supplement 2 [file media-2.pdf]

Table S1. Characteristic of PDOX models applied in the study.

Model	Patient information							PDOX model characteristics						Molecular features in PDOXs			
PDOX model	Patient code	Age at collection	Sex	Tumor localization	Surgery	Treatment received prior PDOX	Histological diagnosis	Implantation	PDOX generation reached	PDOX survival time (days)	Proliferation index (%Ki67 +/- SD)	Detection by MRI	Ploidy tumor cells	Copy number variations (CNV)	Glioma-specific gene mutations	Brain tumor classifier	MGMT promoter methylation
P3	BER0003	64	Male	NA	NA	NA	GBM Grade IV	organoids	>G10	42 +/- 5.2	54.7 +/- 3.7	detectable, quantifiable	2.1N	+[Chr7, 19p, 20q], -[1p36-1p34.1, 1q21.1-q44, -5p15.33-31, Chr9, Chr10, 11p15-14, 20p] --CDKN2A/B	PTEN (chr10:g.87957923del, ENSP00000361021.3:p.Asp236ThrfsTer20)<br>TP53 (chr17:g.7675085C>T, ENSP00000269305.4:p.Cys176Tyr)	GBM, IDHwt, RTK II	methylated
P8	BER0008	64	Female	NA	NA	NA	GBM Grade IV	organoids	>G10	52.5 +/- 3	38.3 +/- 10.8	detectable, quantifiable	2.2N	++EGFR, +[5p15.3-p12, 5q31.1-q35.3, Chr7, 8q24], -[6q21-q27, Chr10, 13q13.3-q34, 15q21.2-q23, 18q21.2-q22.4], --CDKN2A/B	EGFR (chr7:g.55154128G>A, ENSP00000275493.2:p.Ala289Thr)<br>MET (chr7:g.116771573G>T, ENSP00000317272.6:p.Ala954Ser)<br>PTEN (chr10:g.87961047_87961050del, ENSP00000361021.3:p.Thr319Ter);<br>ATRX (chrX:g.77663459C>G, ENSP00000362441.4:p.Ser1348Thr)<br>ATRX (chrX:g.77683638T>A, ENSP00000362441.4:p.Ser540Cys)	GBM, IDHwt, RTK I/II	methylated
P13	BER0013	NA	Female	NA	NA	NA	GBM Grade IV	organoids	>G10	35 +/- 2.5	42.9 +/- 10.8	detectable, non quantifiable	2.3N	+(Chr7, Chr19, Chr20), -(1p21.1-p31.2, 6q16.3-q21, Chr10, Chr13, 17q11-12), --CDKN2A/B	NF1 (chr17:g.31221849G>A, -)- likely not pathogenic	GBM, IDHwt, RTK II	methylated
T16	LIH0016	52	Female	Fronto-parietal right	recurrence	TMZ	GBM Grade IV	organoids	>G10	58.5 +/- 5 (>G7)	18.9 +/- 5.8	detectable, quantifiable	(2.1N) + 3.4N	++[EGFR, MDM4], +7q, - [6q, Chr10, 13q11-q31.1], --CDKN2A/B		GBM, IDHwt, RTK II	unmethylated
T101	LIH0101	60	Male	NA	NA	NA	GBM Grade IV	organoids	G7	106 +/- 5.5	13.4 +/- 4.8	detectable, non quantifiable	(2N) + 4.6N	++[EGFR, MDM2] +[Chr1, Chr7, Chr9, Chr13, Chr16, Chr17, Chr19] -[3q, Chr4, Chr6, Chr10, Chr11, 14q11, Chr15], --CDKN2A/B,	No Glioma-specific mutations	GBM, IDHwt, RTK II/I	methylated
T158	LIH0158	70	Female	Frontal right	primary	no prior treatment	GBM Grade IV	organoids	G5	68 +/-1.6	35.2+/-3	detectable, quantifiable	2.3N	[++MDM2], +[Chr 7, Chr19, Chr20], -[Chr10, Chr15q], --CDKN2A/B	CDK6 (chr7:g.92615172T>A, ENSP00000265734.4:p.Ser317Cys)<br>NF1(chr17:g.31163302G>A, ENSP00000491431.1:p.Gly31Glu)- likely not pathogenic	GBM, IDHwt, RTK II	unmethylated
T185	LIH0185	76	Female	Occipital right	NA	no prior treatment	GBM Grade IV	organoids	G6	89 +/- 10	19.4 +/- 6.9	detectable, non quantifiable	2.2N	++EGFR, +[Chr7, Chr20], -[Chr 10q, Chr22q] --CDKN2A/B	EGFR (chr7:g.55165350G>T, ENSP00000275493.2:p.Gly598Val)<br>MET (chr7:g.116695757A>T, ENSP00000413857.1:p.Met1?)<br>NF1 (chr17:g.31350201A>G, ENSP00000348498.3:p.Glu2426Gly)<br>STAG2 (chrX:g.124090748A>G, ENSP00000218089.9:p.Thr1151Ala)	GBM, IDHwt, RTK I/II	methylated
T186	LIH0186	34	Male	NA	NA	no prior treatment	Anaplastic Oligodendroglioma Grade III	fresh organoids/tissue	G9	159 +/- 43	33+/-4.8	detectable, non quantifiable	2.1-2.4N	complex genome ++[MYCN, CDK4]	IDH1 (chr2:g.208248388C>T, ENSP00000260985.2:p.Arg132His)<br>PDGFRA (chr4:g.54285926A>T, ENSP00000257290.5:p.Asp842Val)<br>MET (chr7:g.116771936C>T, ENSP00000317272.6:p.Thr1010Ile)<br>RET (chr10:g.43100551C>A, ENSP00000344798.4:p.Leu56Met)<br>TP53 (chr17:g.7673803G>A, ENSP00000269305.4:p.Arg273Cys)<br>ATRX (chrX:g.77652140C>A, ENSP00000362441.4:p.Glu1511Ter)	Glioma, IDHm, high grade astrocytoma	methylated
T188	LIH0188	68	Male	Temporal right	primary	no prior treatment	GBM Grade IV	organoids	G6	63+/-3,94	37.1+/- 2.9	detectable, quantifiable	2.3N	[++EGFR], +[Chr7, Chr 19, Chr 20], -[1p36.23, 6p21.32, 6q27, Chr 10, 11q24.2], --CDKN2A/B	MDM4 (chr1:g.204549329A>C, ENSP00000356150.3:p.Lys374Gln)<br>PTCH1 (chr9:g.95459693C>T, ENSP00000332353.6:p.Val932Ile)	GBM, IDHwt, RTK II/I	unmethylated
T192	LIH0192	42	Female	Frontal left	primary	no prior treatment	GBM Grade IV	organoids	G6	71+/-17,8	26.5 +/-8	detectable, non quantifiable	2.4N	++[EGFR, 2q34], +[1q21.2-24.2, Chr7, Chr19, Chr20], - [9p24.3 - 13.3, Chr10],	EGFR (chr7:g.55154017C>T, ENSP00000275493.2:p.Arg252Cys)	GBM, IDHwt, RTK II	unmethylated
T226	LIH0226	61	Male	Supratentoriell	NA	NA	GBM Grade IV	organoids	G5	127+/-4,9	NA	detectable, non quantifiable	NA	++[EGFR], +[Chr 5p, Chr7, Chr 20p], -[1p21.1-p31.2, 6p22.3, 6q16.3-q21, Chr 10, 14q, 16p12.2, 19q, 22q], , --CDKN2A/B	PIK3R1 (chr5:g.68296465A>C, ENSP00000513328.1:p.Gln235His)<br>EGFR (chr7:g.55173057G>A, ENSP00000275493.2:p.Gly665Asp)<br>PTEN (chr10:g.87933154G>A, ENSP00000361021.3:p.Gly132Asp)	GBM, IDHwt, RTK I	methylated
T233	LIH0192	42	Female	Frontal left	recurrence 1	radiotherapy + TMZ	GBM Grade IV	organoids	G5	148+/-7,83	11,3	no detectable	2.3N	++[EGFR, 2q34], +[1q21.2-24.2, Chr7, Chr19, Chr20], - [9p24.3 - 13.3, Chr10, Chr 14q]		GBM, IDHwt, RTK II/I	methylated (v. wide range)
T238	LIH0238	42	Male	NA	NA	no prior treatment	GBM Grade IV	organoids	G3	100+/-8,77	22.1	detectable, non quantifiable	(2.1N) + 3.7N	complex genome, +[Chr7, Chr19, Chr20] -[4q35.2, 6q, Chr10, 13q, 17q], --CDKN2A/B	MDM4 (chr1:g.204542937C>G, ENSP00000356147.3:p.Thr107Arg)<br>PTCH1 (chr9:g.95447309T>C, ENSP00000332353.6:p.Tyr1316Cys)<br>PTEN (chr10:g.87961095C>T, ENSP00000361021.3:p.Arg335Ter)<br>TP53 (chr17:g.7675157G>A, ENSP00000269305.4:p.Pro152Leu)	GBM, IDHwt, RTK II	methylated
T239	LIH0239	80	Male	Parieto-occipital	NA	no prior treatment	GBM Grade IV	organoids	G3	97,5+/-2,34	4.5+/-4.9	NA	2.3N	+[Chr7, Chr19], -[1p36.32-p34.3, Chr10], --CDKN2A/B	MYCN (chr2:g.15942237C>T, ENSP00000281043.3:p.Thr58Met)<br>PIK3R1 (chr5:g.68280986del, ENSP00000513333.1:p.Asn299MetfsTer19)<br>PTEN (chr10:g.87957955C>T, ENSP00000361021.3:p.Pro246Leu)<br>RB1 (chr13:g.48381415G>A, ENSP00000267163.4:p.Arg556Gln)	GBM, IDHwt, RTK I/II	methylated
T251	LIH0192	43	Female	Frontal left	recurrence 2	radiotherapy +TMZ	GBM Grade IV	organoids	G7	65,2+/-1,5	36.1 +/- 7.7	detectable, non quantifiable	2.3N	++[EGFR, 2q34], +[1q21.2-24.2, Chr7, Chr19, Chr20], - [9p24.3 - 13.3, Chr10],		GBM, IDHwt, RTK II	unmethylated
T281	LIH0281	51	Female	Parietal Left	primary	no prior treatment	GBM Grade IV	organoids	G4	126+/-6,06	5.3 +/-0.41	detectable, quantifiable	2.0N + 3.5N	complex genome, +Chr7, - (partial Chr1, 3p, Chr6, Chr9, Chr10, Chr13, Chr14, Chr16, partial Chr17)--CDKN2A/B	PDGFRA (chr4:g.54274864G>C, ENSP00000257290.5:p.Trp559Cys)<br>PTEN (chr10:g.87933012G>A, -)- likely not pathogenic	GBM, IDHwt, RTK I/II	methylated
T304	LIH0304	53	Male	Frontal left	primary	no prior treatment	GBM Grade IV	organoids	G7	89,03+/-4,49	6-janv	detectable, quantifiable	2.2N + 3.2N +4.4N	++[EGFR, CDK4, MDM2], +[Chr7, Chr20], -(partial Chr1, Chr10, partial Chr12)	PTEN (chr10:g.87933166G>A, ENSP00000361021.3:p.Cys136Tyr)	GBM, IDHwt, RTK I/II	unmethylated
T331	LIH0331	84	Male	Fronto-temporo-parietal left	primary	no prior treatment	GBM Grade IV	organoids	G4	116 +/- 8,13	33.2 +/- 2.73	detectable, quantifiable	2.0N	+[Chr 1, Chr 7, Chr 20], -[9p, Chr 10, Chr 22], --[CDKN2A/B, PTEN]	MDM4 (chr1:g.204549329A>C, ENSP00000356150.3:p.Lys374Gln)<br>EGFR (chr7:g.55143387G>A, ENSP00000275493.2:p.Arg108Lys)<br>EGFR (chr7:g.55181309_55181317dup, ENSP00000275493.2:p.Ala767_Val769dup)<br>RET (chr10:g.43124887C>T, ENSP00000344798.4:p.Arg982Cys)	GBM, IDHwt,RTK I/II	methylated
T341	LIH0337	75	Female	Temporal right	primary	no prior treatment	GBM Grade IV	organoids	G4	47,25+/-0,4	37.7 +/- 6.1	NA	2.1N + 3.65N	++[PDGFRA, CDK6], - [3q25.3-q26.2, Chr10], --CDKN2A/B	TP53 (chr17:g.7673806C>T, ENSP00000269305.4:p.Val272Met)	GBM, IDHwt, RTK I	methylated
T347	LIH0347	41	Male	Frontal left	primary	no prior treatment	GBM Grade IV	organoids	G4	125,56+/-5,6	42.1+/-1.9	detectable, non quantifiable	2.2N	++EGFR, +[Chr7, 19p, Chr20], - [1p36.3-p34.3, 1q32.3-q44, Chr10, 13q12.11-31.1, 14q11.2-q21.3], --CDKN2A/B	MET (chr7:g.116700208A>G, ENSP00000317272.6:p.Asn375Ser)<br>PTEN (chr10:g.87961003del, ENSP00000361021.3:p.Cys304SerfsTer3)	GBM, IDHwt, RTK I/II	unmethylated
T356	LIH0281	52	Female	Parietal Left, para-sagittal	recurrence 1	radiotherapy +TMZ	GBM Grade IV	organoids	G5	203,6+/-19,74	28.17 +/- 4.49	detectable, quantifiable	2.1N	complex genome, +Chr7, -(partial Chr1, partial Chr2, partial Chr3, Chr4, Chr6, Chr10, Chr14, Ch17, Chr18)	PIK3CA (chr3:g.179234297A>G, ENSP00000263967.3:p.His1047Arg)<br>PDGFRA (chr4:g.54274864G>C, ENSP00000257290.5:p.Trp559Cys)<br>NF1 (chr17:g.31327521_31327537del, ENSP00000348498.3:p.Val1743AspfsTer12)	GBM, IDHwt, RTK II	unmethylated
T361	LIH0361	69	Female	Temporal left, para-brainstem	NA	no prior treatment	GBM Grade IV	organoids	G5	79+/-0	46.3+/-1.6	detectable, quantifiable	2.3N	++(EGFR, MDM4) + [Chr7, 19p, 20p13-p11.1, 20q13.1-q13.3], -[3q13.33-q21.1, 6p25.3-p21.32, 10q23.2-q26.3], --CDKN2A/B	EGFR (chr7:g.55143387G>A, ENSP00000275493.2:p.Arg108Lys)	GBM, IDHwt, RTK I/II old II	methylated
T363	LIH0363	84	Female	Occipital lobe right	primary	no prior treatment	GBM Grade IV	organoids	G3	140+/-0	6.36	no detectable	2.1N	complex genome ++EGFR,-[Chr 1, 9p, Chr10, 15q], --CDKN2A/B	MET (chr7:g.116771869C>T, ENSP00000317272.6:p.Arg988Cys)<br>RET (chr10:g.43128249A>G, ENSP00000347942.3:p.Met1109Val)	GBM, IDHwt, RTK I/II	methylated
T367	LIH0367	69	Male	Temporal left	primary	no prior treatment	GBM Grade IV	organoids	G3	168+/-0	29.7+/-3.1	detectable, quantifiable	2.3N	++[EGFR, MDM4], +[Chr7, Chr19], - Chr10	PDGFRA (chr4:g.54267424G>A, ENSP00000257290.5:p.Val299Ile)<br>MET (chr7:g.116700208A>G, ENSP00000317272.6:p.Asn375Ser)<br>CDKN2A (chr9:g.21971209C>T, -)<br>PTEN (chr10:g.87957873C>T, ENSP00000361021.3:p.Gln219Ter)	GBM, IDHwt, RTK I/II	methylated
T384	LIH0384	50	Female	Temporo-Occipital left	primary	no prior treatment	GBM Grade IV	organoids	G4	202+/-6,88	NA	no detectable	NA	++[EGFR, 7p15.2-p11.2, 7q31.31-32.2], -[4p, 9q, 10q, partial Chr 12, partial Chr 15], --[FGFR1\TACC1, MYBL1]	TP53 (chr17:g.7674257A>T, ENSP00000269305.4:p.Tyr236Asn)	GBM, IDHwt, RTK II (no match)	methylated
T386	LIH0386	51	Male	Frontal left	primary	no prior treatment	GBM Grade IV	organoids	G4	104,1+/-6,83	28.9+/-0.72	detectable, quantifiable	2.1N	+[Chr7, 19p], - [1p36.3-p34.3, Chr10, 11q12.3-q13.2, 11q24.1-q13.2, 14q11.2-q24.1, 17q11.2-q21.2, 18p11.32-p11.22, 22p], --[CDKN2A/B, PTEN]	PIK3R1 (chr5:g.68293801_68293803del, ENSP00000323512.8:p.Asp164_Arg165delinsGlu)<br>NF1(chr17:g.31221932A>G, ENSP00000412921.4:p.Tyr575Cys) likely not pathogenic	GBM, IDHwt, RTK I	unmethylated
T394	LIH0394	45	Female	Fronto-basal right	recurrence 1	radiotherapy	GBM Grade IV	fresh organoids/tissue	G5	68,56+/-0,9	67.8+/-8.3	detectable, quantifiable	2.1-2.3N + 3.5N	complex genome ++[PDGFRA,FGFR3/TACC3, KIT, KDR, MET, Chr 4, 7q31.31], --CDKN2A/B	IDH1 (chr2:g.208248388C>T, ENSP00000260985.2:p.Arg132His)<br>MET (chr7:g.116700208A>G, ENSP00000317272.6:p.Asn375Ser)<br>TP53 (chr17:g.7670697_7670707del, ENSP00000269305.4:p.Glu336AspfsTer7)<br>ATRX (chrX:g.77681626_77681628del, ENSP00000362441.4:p.Asp1212del)<br>ATRX (chrX:g.77682604_77682607del, ENSP00000362441.4:p.Glu886LeufsTer18)	Glioma, IDHm, high grade astrocytoma	methylated
T407	LIH0394	45	Female	Fronto-basal right	recurrence 1	radiotherapy	GBM Grade IV	fresh organoids/tissue	G6	64+/-4,27	49.5+/-6.8	detectable, quantifiable	2.1N + 3.9N	complex genome ++[PDGFRA,FGFR3/TACC3, KIT, KDR, MET, Chr 4, 7q31.31], --CDKN2A/B	IDH1 (chr2:g.208248388C>T, ENSP00000260985.2:p.Arg132His)<br>MET (chr7:g.116700208A>G, ENSP00000317272.6:p.Asn375Ser)<br>TP53 (chr17:g.7670697_7670707del, ENSP00000269305.4:p.Glu336AspfsTer7)<br>ATRX (chrX:g.77681626_77681628del, ENSP00000362441.4:p.Asp1212del)<br>ATRX (chrX:g.77682604_77682607del, ENSP00000362441.4:p.Glu886LeufsTer18	Glioma, IDHm, high grade astrocytoma	methylated
T434	LIH0304	54	Male	Frontal left	recurrence 2	radiotherapy + TMZ	GBM Grade IV	organoids	G6	43,20+/-0	40.4 +/- 11.43	detectable, quantifiable	2.0N + 2.3N +3.8N	++[EGFR, CDK4, MDM2], +[7p,7q36.1-q36.2, Chr20], -[1p36.23-p36.21, 6q26-q27, Chr10, 12q15, 14q13-qter]	EGFR (chr7:g.55154023T>A, ENSP00000275493.2:p.Phe254Ile)<br>PTEN (chr10:g.87933166G>A, ENSP00000361021.3:p.Cys136Tyr)	GBM, IDHwt, RTK II	unmethylated
T470	LIH0347	42	Male	Frontal left	recurrence 1	radiotherapy + TMZ	GBM Grade IV	organoids	G4	78,33+/-15,27	17.98 +/- 0.88	detectable, quantifiable	2.2N	++EGFR, +[Chr7, 19p, Chr20], - [1p36.3-p34.3, 1p31.1-p11.21, 1q32.3-q44, Chr10, 13q12.11-31.1, 14q11.2-q21.3], --CDKN2A/B	MET (chr7:g.116700208A>G, ENSP00000317272.6:p.Asn375Ser)<br>PTEN (chr10:g.87961003del, ENSP00000361021.3:p.Cys304SerfsTer3)	GBM, IDHwt, RTK II	unmethylated
T476	LIH0476	75	Male	Fronto-parietal left	primary	no prior treatment	GBM Grade IV	organoids	G4	66+/-0	44.3+/-3.8	detectable, non quantifiable	2.3N	++[EGFR, MDM4], +[Chr 7], - [9p24.3-p21.3, Chr 10], --[CDKN2A/B, PTEN]	EGFR (chr7:g.55165350G>T, ENSP00000275493.2:p.Gly598Val)<br>EGFR (chr7:g.55200333G>C, ENSP00000275493.2:p.Asp956His)<br>MET (chr7:g.116771936C>T, ENSP00000317272.6:p.Thr1010Ile)	GBM, IDHwt, RTK I	unmethylated
T515	LIH0515	46	Male	Frontal-right	primary	no prior treatment	Anaplastic Oligodendroglioma Grade III	fresh organoids/tissue	G5	393,79+/-55,56	NA	detectable, quantifiable	NA	NA	NA	NA	NA
T591	LIH0384	52	Female	Temporo-Occipital right	recurrence 1	radiotherapy +TMZ + Avastin + Irinotecan	GBM Grade IV	organoids	G3	110+/-16	22.08+/-12.72	detectable, quantifiable	2.2N + 3.5N	++[EGFR, 7p15.2-p11.2, 7q31.31-32.2], -[1p21.1, 3q26.1, 8p, 9p24.3, Chr 10, 11q24.2, 13q21.33-q31.1, 15q]	No Glioma-specific mutations	GBM, IDHwt, RTK II/I	unmethylated
T744	LIH0744	69	Male	Bilateral involvement of the hippocampus	primary	no prior treatment	GBM Grade IV	organoids	G3	100+/-0	28.9+/-23.5	detectable, quantifiable	2.2N	+Chr 7, -[6q, 9p, Chr10, Chr 11, 14q, 22q], --CDKN2A/B	NF1 (chr17:g.31338092C>T, ENSP00000348498.3:p.Arg2237Ter)	GBM, IDHwt, RTK I/II	unmethylated
T756	LIH0556	46	Male	Multifocal left	recurrence 3	radiotherapy	GBM Grade IV	fresh organoids/tissue	G5	77,93+/-1,42	12.09 +/-1.62	detectable, quantifiable	2.2N + 2.9N + 3.9N	+ [4q12, 10p], -[4p15.32-q35.2, 6q27, 10q, partial Chr 16, 22q], --CDKN2A/B	IDH1 (chr2:g.208248388C>T, ENSP00000260985.2:p.Arg132His)<br>PTCH1 (chr9:g.95469004G>A, ENSP00000332353.6:p.Thr666Met)<br>TP53 (chr17:g.7675085C>T, ENSP00000269305.4:p.Cys176Tyr)	Glioma, IDHm, high grade astrocytoma	methylated
T772	LIH0615	54	Female	Fronto-temporal left	recurrence 1	radiotherapy + TMZ	GBM Grade IV	organoids	G4	107+/-0	32.1+/-1.2	detectable, non quantifiable	2.1N	++EGFR, +[7p, Chr19], -[1p36.3, 9p, Chr 10, 15q] --CDKN2A/B	EGFR (chr7:g.55165350G>T, ENSP00000275493.2:p.Gly598Val)<br>RET (chr10:g.43124887C>T, ENSP00000344798.4:p.Arg982Cys)<br>PTEN (chr10:g.87961115del, ENSP00000361021.3:p.Phe341LeufsTer3)	GBM, IDHwt, RTK I	methylated
T784	LIH0784	56	Male	Frontal left	primary	no prior treatment	GBM Grade IV	organoids	G4	138+/-2	43.5+/-3.3	detectable, non quantifiable	2.1N	+[Chr 7], -[1p36.3-22.1, 3p12.1, 10q], --CDKN2A/B	MET (chr7:g.116695757A>T, ENSP00000413857.1:p.Met1?)<br>RET (chr10:g.43102379C>A, ENSP00000491505.1:p.Leu107Ile)<br>RB1 (chr13:g.48381295G>A, ENSP00000267163.4:p.Trp516Ter)<br>STAG2 (chrX:g.124094127dup, ENSP00000218089.9:p.Thr1230AsnfsTer21)	GBM, IDHwt, RTK I	methylated
T797	LIH0797	55	Female	Left occipital	primary	no prior treatment	GBM Grade IV	organoids	G3	119+/-34,5	9.65 +/-0.40	detectable, quantifiable	2.2N + 3.9N	complex genome ++[CDK4, MDM2], +[Chr 7, Chr 20], -[Chr 10, 13q, 15q, 22q]	NA	GBM, IDHwt, RTK I	methylated
T831	LIH0831 (n	51	Female	Bifrontal	primary (multifo	no prior treatment	GBM Grade IV	organoids	G3	70+/-0	3.46 +/- 2.33	NA	NA	++EGFR, +[Chr 7], -[3p12.1, 6p24.2, Chr 10, 13q, 16p12.2], --CDKN2A/B	PIK3CA (chr3:g.179199048C>G, ENSP00000263967.3:p.Gln75Glu)	GBM, IDHwt, RTK II/I	methylated
T832	LIH0831 (n	51	Female	Bifrontal	primary (multifo	no prior treatment	GBM Grade IV	organoids	G3	138,5+/-14,50	0.25 +/- 0.04	no detectable	2.1N	NA	NA	NA	NA
T841	LIH0841	38	Male	Right temporal	primary	no prior treatment	GBM Grade IV	organoids	G3	49,2+/-1,09	NA	NA	NA	complex genome ++[MYCN, CDK4], +Chr 7, -[2p24.2, 6q12, Chr 10, 13q31.2, 14q12, 15q15.3]	PIK3CA (chr3:g.179199156_179199161del, ENSP00000263967.3:p.Lys111_Ile112del)<br>MET (chr7:g.116695757A>T, ENSP00000413857.1:p.Met1?)<br>CDKN2A (chr9:g.21970917C>T, ENSP00000307101.5:p.Ala148Thr)	Glioblastoma, Idh Wildtype, With Primitive Neuronal Component (No match)	unmethylated
T861	LIH0841	38	Male	Right temporal	recurrence 1	radiotherapy + TMZ	GBM Grade IV	organoids	G2	157,2+/-3.88	NA	detectable, non quantifiable	NA	NA	NA	NA	NA
T899	LIH0609	75	Female	Right Central	recurrence 1	no prior treatment	GBM Grade IV	organoids	G2	70,75 + /- 2,63	NA	NA	NA	complex genome ++[PDGFRA, CDK4], -[Chr10, 14q]	PIK3R1 (chr5:g.68296465A>C, ENSP00000513328.1:p.Gln235His)<br>PTCH1 (chr9:g.95468828G>A, ENSP00000332353.6:p.Pro725Ser)<br>CDK4 (chr12:g.57749225G>A, ENSP00000257904.5:p.Ser259Leu)<br>TP53 (chr17:g.7674188C>A, ENSP00000269305.4:p.Asp259Tyr)	Diffuse Pediatric Type High Grade Glioma, H3 Wildtype And Idh<br>Wildtype, RTK I (No match)	methylated
T905	LIH0609	76	Female	Right Central	recurrence2	no prior treatment	GBM Grade IV	organoids	G3	91+/-1,73	NA	detectable, non quantifiable	NA	NA	NA	NA	NA
T1053	LIH1053	62	Male	Right intraaxial	primary	no prior treatment	GBM Grade IV	organoids	G2	98.75+/-0.5	NA	NA	NA	NA	NA	NA	NA
T1070	LIH0784	60	Male	Left frontal	recurrence 1	radiotherapy + TMZ	GBM Grade IV	organoids	G1	254.25 +/- 36.18	NA	detectable, quantifiable	NA	NA	NA	NA	NA

Table S2. List of antibodies used in the study.
*Flow cytometry test 10^6 cells/100µl

Antibody	Supplier	Catalog number	Concentration used
hCD90 PE-Cy7	BD	561558	5 µl/test*
hCD90 BV605	BD	562685	5 µl/test*
CD31 Dy590 (PE-TR)	Immunotools	21270317.5	10µl/test*
CD45 PE-Cy7	BD	557748	5µl/test*
CD16/32	eBioscience	14-0161-85	1 µl/test*
mCD45 FITC	eBioscience	11-0454-82	1 µl/test*
mCD11b Percp-Cy5.5	BD	550993	5 µl/test*
mLy6C PB	Biolegend	128014	0.5 µl/test*
mLY6G BV785	Biolegend	127645	2.5 µl/test*
mCCR2 PE	R&D	FAB5538P	5 µl/test*
iso IgG PE	R&D	IC108P	5 µl/test*
mCD206 APC	Biolegend	141708	2.5 µl/test*
mCD11c APC-Cy7	Biolegend	117323	5 µl/test*
mCD86 BV605	Biolegend	105037	5 µl/test*
iso IgG2a BV606	Biolegend	400540	5 µl/test*
m I-A/I-E APC-Cy7	Biolegend	107628	1 µl/test*
mCD45 BV605	Biolegend	11045182	1 µl/test*
LIVE/DEAD™ Fixable Near-IR	Invitrogen	L34975	0.1 µl/test*
Nestin	Abcam	AB6320	IHC:1/500
Vimentin	Thermo Fisher Scientific	Mab3400	IHC:1/200
CD31	Cell Signaling	77699	IHC:1/100
Iba1	Biocare Medical	CP 290A	IHC:1/1000
Iba1	Wako	019-19741	IHC:1/1000
CD11c	Abcam	ab11029	IHC:1/100
GFAP	Dako	Z0334	IHC:1/1000
MHC-II	Abcam	ab25333	IHC:1/100
Ki-67	ThermoScientific	14-5698-82	IHC: 1/100
Pdgfra	Biolegend	135905	IHC:1/100
Anti-Rat IgG Alexa Fluor 555	Invitrogen	A21434	IHC:1/1000
Anti-Rabbit IgG Alexa Fluor 555	Thermo Fisher Scientific	A-11039	IHC:1/500
Anti-Mouse IgG Alexa Fluor 647	Thermo Fisher Scientific	A-11037	IHC:1/500
Anti-Rat IgG Alexa Fluor 488	Thermo Fisher Scientific	A-21244	IHC:1/500
Opal 520 Flurophore	Akoya Biosciences	NEL810001KT	IHC:1/100
Opal 570 Flurophore	Akoya Biosciences	NEL810001KT	IHC:1/100

Table S3. Characteristics of cell clusters identified in DROP-seq data in mouse-derived TME. Marker genes for each cluster were defined as differentially expressed genes between clusters at threshold $\log_2$FC>=0.5

Genes are listed from the highest to lowest $\log_2$FC

Cluster number	Astrocytes	Myeloid	Endothelial	OPCs	Oligodendrocytes	Cycling	Ependymal	Pericytes	Lymphocytes
	Slc1a2	Ctss	Ly6c1	Cacng4	Plp1	Hist1h4c	Ttr	Ccdc153	Ccl5
	Aqp4	Hexb	Cxcl12	Pdgfra	Ermn	Top2a	Enpp2	Tmem212	Nkg7
	Gja1	Lyz2	Cldn5	Olig1	Mbp	Gm14892	1500015O10Rik	Rarres2	Ms4a4b
	Aldoc	C1qb	Itm2a	Rtn1	Mag	Cenpf	Prlr	Rsph1	Trac
	Slc1a3	Cx3cr1	Igfbp7	Cntn1	Ptgds	Gm12331	Calml4	Enkur	Gzmb
	Bcan	Cd74	Rgs5	Gng3	Cldn11	Prc1	Igf2	Fam183b	Trbc2
	Ttyh1	Tyrobp	Bsg	Lhfpl3	Mobp	Ezh2	Kl	Ak7	Cd3g
	Slc4a4	C1qa	Flt1	3110035E14Rik	Mal	Smc4	Kcnj13	Mns1	Trbc1
	Clu	P2ry12	Abcb1a	Cspg5	Cnp	Gm17909	Cab39l	Dynlrb2	Gimap4
	Plpp3	Spp1	Slco1a4	Ccnd1	Stmn4	RP24.232I9.2	Stk39	Spef2	Klrd1
	Atp1a2	C1qc	Spock2	Pcsk1n	Apod	Gm12152	Igfbp2	Cfap44	Cd3d
	Scg3	Ctsd	Adgrf5	Nap1l5	Tspan2	Smc2	Trpm3	Ccdc146	Gimap3
	Acsl3	Csf1r	Ly6a	Meg3	Aplp1	Gm12504	Folr1	Wdr66	Ptpn22
	Pla2g7	Laptm5	Ctla2a	Tnr	Car2	Rps19.ps4	Cdkn1c	Rsph4a	P2ry10
	Htra1	H2.Ab1	Car4	Ncald	Trf	Gm5853	Chchd10	Calml4	Ptprcap
	Cldn10	Cxcl13	Pltp	S100a1	Qdpr	Cenpe	Sostdc1	Cfap126	Cytip
	Tspan7	Fcer1g	Ptprb	Cdo1	Gm21984	Ube2c	Clic6	Cfap45	Sept1
	Id4	Mpeg1	Egfl7	Gap43	Sept4	Gm12165	Rbp1	Iqcg	Lck
	Acsl6	Siglech	Palmd	Fabp7	Pex5l	Gm18014	Otx2	Fam216b	H2.Q7
	Ntsr2	H2.Aa	Ramp2	Olig2	Mog	Gm14537	Pcp4	4933434E20Rik	Thy1
	Gpr37l1	Trem2	Gng11	Scg5	Opalin	Tpx2	Tmem72	Spa17	Mndal
	Sparcl1	Ccl4	Adgrl4	Vcan	Tmem88b	Gm13121	Ppp1r1b	Gm45396	AW112010
	Dclk1	Clec7a	Kitl	Sulf2	Ugt8a	Gm4321	F5	Ccdc39	Nr4a2
	Mt3	Itgam	Esam	Epn2	Il33	Nusap1	Fam81a	Meig1	Stk17b
	Atp1b2	Aif1	Ablim1	Matn4	Ttll7	Gm17511	Ahcyl2	Ccdc113	S100a4
	Gpm6a	Il1b	Slc39a10	Anks1b	Ptprd	RP23.390K13.4	Cul4b	Gm973	Gimap6
	Cpe	Gpr34	Arhgap29	Bex2	Gjc3	Gm44010	Vat1l	Lrrig1	Ptprc
	Sox2	Ctsb	Tm4sf1	Pllp	Anln	Birc5	Car12	Ppp1r36	Vps37b
	Id2	Hpgds	Col4a2	Stmn3	Epb41l3	Gm11223	Hemk1	Wdr78	Plac8
	Luzp2	Ccl3	Pcp4l1	Gria4	Ccp110	2810417H13Rik	Sulf1	1110017D15Rik	Il2rg
	S1pr1	Lgmn	Sdpr	Kcnip3	Gnai1	Kif15	Ociad2	Mia	Ifi203
	Ptprz1	Rnase4	Slco1c1	Scrg1	Tubb4a	Gm8790	Spint2	Dnah12	Hcst
	Ndrg2	Lpl	Pecam1	Il1rap	Aspa	Gm44122	Msx1	Spag17	S100a10
	Mfge8	Fyb	Uaca	Gjc3	Spock3	Hmmr	Rsph1	1700016K19Rik	Rps18
	Nrxn1	Tgfbr1	Nostrin	Gria3	Tppp	RP23.144N15.3	Fhad1	Foxj1	Rps15a
	Tmem47	Ctsz	Abcg2	Bcas1	Map7	Ncapg	Slc4a10	Cd24a	Rpl34
	Gria2	Grn	Col4a1	Tuba1a	Slc24a2	Gm7860	Kcne2	Tppp3	H2.K1
	Mt2	Selplg	Ifitm3	Serpine2	Lpar1	Bub1	Htr2c	Sntn	Rps24.ps2
	Mt1	Ccl6	9430020K01Rik	Cspg4	Cntn2	Kif23	Atp2b3	1700012B09Rik	Cd52
	Lsamp	Plek	Hspb1	Phlda1	Pllp	Nuf2	Col9a3	Tmem107	H2.Q6
	Ntrk2	Fcgr3	Tsc22d1	Dbi	Mapt	Gm5070	Emb	Riiad1	Gm11478
	Ednrb	Ly86	Slc9a3r2	S100a16	Bcas1	Mki67	Drc7	Ccdc180	AU020206
	Prnp	Rgs2	Pglyrp1	Ednrb	Sox2ot	Rps12.ps19	Cox6b2	Cfap43	Gm6030
	Ckb	Apbb1ip	Cp	Tmem100	Edil3	Tubb3	Fam161a	Drc1	Rac2
	Gpm6b	Cd68	Itih5	Gria2	Gjb1	Gm28344	Lrrig1	Iqca	Rps3
	Ptn	Rgs1	Kdr	Nnat	Desi1	Gm3788	Mlf1	Bphl	Srgn
	Pou3f3	Cd52	Nes	Ptprz1	Gpr37	Gm8513	Krt18	Cep126	Ms4a6b
	Prdx6	Mafb	Sptbn1	Nav1	Ndrg1	Rpl21.ps5	Rdh5	Mlf1	Gm15427
	Cspg5	Rgs10	Slc2a1	Syt11	Olig1	Gm2522	Plppr4	Fhad1	Rps2
	Gstm1	Slc15a3	Rflnb	Phactr3	Dusp26	RP23.408J7.2	Col8a1	Cfap54	Rplp1
	Cxcl14	Olfml3	Ccdc141	Nrxn2	Map1a	Gm2178	Tbc1d9	Odf3b	Tmsb10
	Ldhb	Atf3	Myo1b	Map2	Grb14	Nasp	Car14	Dnali1	Rps24
	Fabp7	Npc2	Tpm1	Gpm6b	Enpp2	Rpl26.ps2	Atp1b1	Cfap161	Gm9794
	Syt11	Zfp36	Ece1	Rpl13	Scd2	Rpl34.ps2	Tmem237	1500015O10Rik	Rps15a.ps5
	Timp3	Man2b1	Utrn	Myo5a	Sez6l2	Acta1	Gpx8	Elof1	Wdr89
	Fam213a	Cd14	Tspan13	Lbh	Plekhb1	Gm11810	Sh3d19	Dnah6	Rpl39
	Syne1	Slc11a1	Slc16a1	Ttc3	Hapln2	Gm11517	Ctnnal1	Ccdc173	Rps14
	Asrgl1	Cst7	Ets1	Ahi1	Elavl3	Rpl10l	Arsg	Lrrc23	Gm10275
	Glul	Irf8	Myl12a	Tspan3	Ank3	Rps12.ps24	Acad8	Ascc1	Rps13.ps2
	Scd2	Pld4	Klf2	Asrgl1	Trim59	Gm7351	Slc22a17	Ak9	B2m
	Luc7l3	Golm1	Esyt2	Fip1l1	Ppp1r14a	Gm2225	Pcp4l1	Hydin	Shisa5
	Tspan3	Creg1	Igf1r	Hip1	Pls1	Tubb5	Fxyd1	Cfap100	Rps26
	Hnrnpa2b1	Cyth4	Tmsb10	Basp1	Slc44a1	Gm13215	Gsta4	Dnaic1	Rpl32
	Kif1b	Unc93b1	Tpm4	Rev3l	Slc48a1	Gm7558	Acaa2	Dnah5	Rpsa.ps10
	Apc	Ptprc	BC028528	Arl2bp	Efnb3	Tmpo	Sfrp1	Nme5	Samd9l
	Ccdc88a	Maf	Gm10080	S100b	Cmtm5	Gm5370	Gm17018	Ttc21a	Ets1
	Glud1	Ctsa	Ptprg	Ccnd2	Kcna1	Gm15896	Mrps6	Hdc	Rps24.ps3
	Serpine2	Ctsc	Epas1	Scd2	Fa2h	Gm11472	Mcee	Ccdc81	Gm9843
	Zbtb20	Itgb5	Akap12	Ppfibp1	Mapk8ip1	Gm15159	Atp5g1	Efcab1	Gm15500
	Spag9	Cyba	Ppfibp1	Slc25a4	Nfasc	Gm9625	Rabgap1l	Nme9	Ifngr1
	Id3	Plxdc2	Crip2	Hmgcs1	Aatk	Rpl17.ps4	Phactr2	Elmod1	Rps15
	Macf1	Mef2c	Vwa1	Nrxn1	Sept8	Rpl27.ps2	Tubb4b	Akap14	Rplp0
	Sept7	Cd53	Myl12b	Tmem176b	Lgi3	Gm14277	Car2	Rsph10b	Rpl22
	Dst	Ptgs1	Arl4a	Tpm1	Tmeff2	Rpl13.ps5	Mdh1	Rgs22	Rpl4
	Vim	Cd84	Golim4	Psip1	Cpox	Gm6543	Bphl	Gm28729	Rpl35a
	Malat1	Cybb	Ly6e	Ramp1	Tppp3	Lmnb1	Map1lc3a	Efcab2	Rps20
	mt.Rnr1	Lair1	App	Zeb1	Dnajb2	Incenp	Ift74	3300002A11Rik	Rps5
	Qk	Ccr5	Itgb1	Pcdh17	Dixdc1	Nucks1	Slc16a2	Ttc25	H2.D1
	Pnisr	Hexa	Sparc	Atxn7l3b	Josd2	Gm14450	Prdx5	1700007K13Rik	Gm5963
	Fermt2	Fcgr1	Id3	Lsamp	Nkx6.2	Gm14480	Gm10039	Armc3	Slfn2
	Arglu1	Cst3	Calm1	Pcdh10	Shtn1	Rrm1	Tbcel	Cfap69	Rps21
	Son	Junb	Sept4	Pebp1	Arsg	H2afv	Maob	1700024G13Rik	Eef1b2
	Arhgap5	Cd83	Atox1	Prdx2	Sox10	Rps3a3	Mtch1	Lrrc51	Rplp2
	Rac1	Kctd12	Crip1	Ndufa4	Sv2a	Gm12857	Hist1h2bc	1700088E04Rik	Lcp1
	Rtn3	B2m	Id1	Klhl5	Cryab	Rad21	1110004E09Rik	Tctex1d4	Rps19
	Tsc22d4	Ncf1	Cald1	Kifap3	Pcdh9	Gm29228	Thyn1	Syt10	Rps3a1
	Tcf4	Lpcat2	Fxyd5	Luzp2	Anks1b	Gm11560	Elovl7	Efcab10	Gm5805
	Ywhae	Smap2	Luzp1	Atp1b1	Plcl1	Fth.ps2	Tgfb2	Armc4	Rpl37rt
	Serinc1	Apoe	Col4a3bp	Alcam	Tmod2	Gm7618	Gas6	Capsl	Rpl3.ps1
	Slc25a4	Cd37	Slc6a6	Hsd17b12	Slc12a2	Gm6136	Ifi27	Lrrc48	Rps16.ps2
	Hnrnpdl	Nfkbiz	Cd2ap	Ifi27	Agpat4	Bex3	1110032A03Rik	Cdhr3	Gm6472
	Ktn1	Ms4a6c	Fnbp1l	Cd9	Gpm6b	Gm12482	Hopx	Lrrc6	Tpt1.ps3
	Gm42418	Lat2	Myl6	Ubb	Syt11	Rps19.ps6	Ltc4s	Ccdc30	Gm5905
	Eif5	Lcp1	Fermt2	Eif1b	Gsn	Gm13815	Cd59a	Dnah3	Rpl9.ps6
		Samsn1	Cd34	Atpif1	Fez1	Gm13340	Ndufa11	Tekt1	S100a11
		Arsb	H2.D1	Hsp90aa1	Dnm3	Gm7308	Ppfibp2	Maats1	Rps9
		Gns	Bnip2	Ptn	Rtn4	Gm15773	Thumpd3	Nek5	Rpl3
		Sgk1	Cyb5r3	Nme1	Csrp1	Hmgb2	Ndufv3	Cfap46	Cnn2
		Egr1	Sept11	Chpt1	App	Hnrnpa2b1	Cox20	Lca5	Gm14513
		Pycard	Srgn	Xist	Trim2	Atad2	Usmg5	Crygn	Rps15a.ps7
		Gusb	Sh3bgrl	Ankrd11	Ywhag	Rbmx	Ldhb	Pifo	Gm6204
		Cotl1	Rpl38.ps2	Cadm2	Elovl7	Selenoh	Cox7b	Nnat	Gm8995
		Btg2	Ctnna1	Ncam1	Otud7b	Gm27046	Ndufa1	Chchd10	Rpl14.ps1
		Abhd12	Hes1	Ckb	Pak1	Gm2000	Cox6c	Ift88	Rps11
		Ifi27l2a	Dynll1	Lmo4	Fnbp1	Ddx39	Uqcr10	Fam81a	Ahnak
		Lyn	Gm4617	Tubb5	Shisa4	Vim	Anapc13	Fhl1	Rps10.ps1
		Jun	Shfm1	Fubp1	Secisbp2l	Hnrnpd	Glrx3	Dnal1	Rpl18.ps1
		Basp1	Fn1	Smarcc1	Kif1b	Ybx1	Cetn2	Nuf2	Rps10
		Ctsh	Anp32a	Hnrnpr	Gatm	Rps15.ps2	Atp11c	Chek2	Btg1
		AU020206	Aplp2	Hspd1	Tulp4	Gm16379	Idh3a	Ccdc34	Gm16580
		Pmepa1	Selenop	Bzw1	Chn2	Gm4204	Atp6v1a	Slc38a1	Pabpc1
		Sirpa	Iqgap1	Tsc22d4	Kif21a	RP23.114G13.7	Etfa	2610301B20Rik	Gm5835
		Syngr1	Nedd4	Srrm2	Spg20	Gm5778	Gm12669	Nudt4	Psmb8
		Psap	Nrp1	Ppp2r2a	Cdc37l1	Psip1	Cgnl1	Hspa4l	Rpl8
		Serinc3	Srpr	Nedd4	Sirt2	Dnmt1	Pebp1	Prelp	Rpsa
		Ier5	Atp5j	Anp32e	Bin1	Hspd1.ps3	Vamp8	Rassf9	Crip1
		Rhob	Kif5b	Top2b	Inpp5f	Gm13498	Dynlt3	Fam161a	Arhgap30
		Ctsl	Ubb	Ostf1	Sccpdh	Pcna.ps2	Ezr	Gm5617	Rps26.ps1
		Arhgdib	Gnai2	Ccdc88a	Psat1	Ssrp1	Atp5h	Wdr60	Gm4604
		Bin1	Chchd2	Tecr	Gstp.ps	Calm2	Gm16418	Ift74	Lgals1
		Lgals3bp	Pomp	Agpat5	Sept7	Gm10167	Arl6ip1	Aebp1	Rpl26
		H2.K1	Slc3a2	Pnn	Arpc1a	Gm12222	2010107E04Rik	Cetn2	Gm4149
		Ivns1abp	Gm6472	Eid1	Osbpl1a	Anp32e	Uqcr11	Ift81	Rack1
		Efhd2	Gm9800	Hsbp1	Neat1	Dek	Aldh2	1110032A03Rik	Rpl30
		Bmp2k	Gm14586	Ybx1	Rnf13	Ccdc34	Hint2	Dbi	Ifi27l2a
		Arpc1b	Gm11966	Rab2a	Cers2	Gm11488	Hcfc1r1	Cep290	Sp100
		Cd9	1810037I17Rik	Ndufa5	Erbin	Gm4332	Cox8a	Hsp90aa1	Gm10288
		Scamp2	Laptm4a	Tial1	Fam171b	Anp32b.ps1	Ndufa4	Nphp1	Rpl41
		Timp2	Nfkbia	Dhx36	Dbi	Rn7s6	Gm16399	Rpgrip1l	Hspa8
		Rin2	Msn	Dnaja1	Pip4k2a	Ilf3	Rnf7	Strbp	Rps11.ps2
		Rrbp1	Ndufa7	Ywhab	Gm11223	Gm22567	Atp5o	Nin	Gm14303
		Gm14303	Psma7	Cox6a1	Evi2a	Pa2g4	Atp5d	Tubb4b	S100a6
		Fam49b	Tpt1.ps3	Hnrnpk	Cdc42bpa	Anapc11	Etfb	6330403K07Rik	Fxyd5
		Epb41l2	H3f3b	Thoc7	Degs1	Snrpd1	Ap1s2	Hsph1	Gm4332
		Cfh	Ptma	Nap1l1	Nap1l5	Ranbp1	Gm10250	Gm14964	Rps11.ps1
		Mef2a	Gm10250	Dynll2	Map7d1	Ybx1.ps2	Ndufb10	Fam149a	Sh3bgrl3
		Mertk	Gm4149	Cox6c	Tuba1a	Gm6159	Arl3	Pcp4l1	Gas5
		Rnaset2a	Spop	Cacybp	Efcab14	Gm9294	Cox4i1	Rdh10	Rpl39.ps
		H2.D1	Sh3glb1	Wbp5	Tsc22d4	Dnajc9	Atp5g3	Zcchc18	Rpl23
		Erp29	Cox6a1	Hnrnpc	Pla2g16	Gm13461	Cisd1	Gm12346	Eef1a1
		Gpx1	Rpl41	Rpl13a	Gltp	Gm11942	Pgrmc1	Hipk1	Rpl38.ps2
		Marcks	Anxa5	Ran	Ppp2r3a	Hdac2	Acadsb	Nek1	Pdcd4
		Tmsb4x	Snx3	Pcm1	Gstp1	Gm8186	Slc31a1	1110004E09Rik	Jak1
		Actb	Cdc42	Rrp1	Taldo1	Gm13092	Slc12a2	Ccdc191	Rps10.ps2
		Itm2b	Rpl37rt	Arl6ip1	Sec11c	Gm6394	Polr2m	Tsga10	Gm9385
		Rplp1	Cbx1	Zmynd11	Rnf7	Gm11633	Ndufb2	Gm12222	Rpl10a.ps1
		Pabpc1	Slk	Hnrnpab	Kif1a	Snrpe	Ndufc1	Ccdc181	Psmb9
		Fth1	Timp3	Cox6b1	Epn2	Ppm1g	Dstn	Ildr2	Rpl37
		Sparc	Cox6c	Rab10	Mbnl2	Snrpd2	Ccdc34	Tm4sf1	Cntrl
		Trf	Tpm3.rs7	Sec11c	Ncam1	Hist1h1e	Atp5j	Ipo11	Junb
		Rps24	Dnajc8	Acat1	Thra	Hnrnpr	Gm14539	Wwc1	Npm1
		Skil	Rps3a1	Ndufc2	Ank2	Hnrnpab	Ccng1	Hspa2	H2.T23
		Zeb2	Rps19	Hmgn1	Dpysl2	Gm12020	Ndufa13	Cep83	Gm8692
		Psmb8	Uqcrb	Ranbp1	Gnao1	Ywhae	Smim14	Syne1	Gm8730
		Ywhah	Rps27l	Cwc22	Kcnj10	Hint1	Mrpl58	Gsta4	Elf1
		Ifngr1	Rpl31.ps8	Ppib	Qk	Gm12346	Acot13	Synm	Rpl36a.ps2
		Srgap2	Atp5h	Cct6a	Clasp2	Hnrnpdl	Aldoa	Eea1	Mbnl1
		Mtdh	Srp14	Set	Myo6	Gm12696	Slc25a39	Mlc1	Rpl14
		Gm9843	Tceb2	1810037I17Rik	Hsp90aa1	Myh10	Ghitm	Tmem47	Arhgdib
		Mcl1	Akr1a1	Atp5o	Cldnd1	Rack1	Ndufb6	Mycbp	Rps8
		Ly6e	Cox7a2	Fkbp3	Hspa8	Usp1	Hprt	Fam213a	Gm15421
		Hsp90b1	Rock2	Tceb2	S100a16	Cbx5	Phyh	Odf2l	Gm43712
		Gm8995	Ptms	Rsl1d1	Atp8a1	Hn1	Ubl5	Irak1bp1	Pnrc1
		Capza2	Chmp2a	Dnm1l	Fth1	Myef2	Suclg1	Dync2h1	Ccnd2
		Mbnl1	Minos1	Snrpd3	Tmcc3	Rheb	Tnfaip8	Enah	Pfn1

		Rpl32	Txn1	Cox7a2	Glul	Rps5	Atp6v1e1	Gm17018	Gm15148
		Snx5	Dstn	Gsk3b	Atp1b3	S100a6	Sh3bgrl	Tex9	2410006H16Rik
		Rps9	Itsn2	Psma4	Phyhipl	Hnrnph3	Ndufs5	Gas6	Zfas1
		Cyfip1	Ndufc1	Akap9	Fam168a	Atp5e	Vcam1	Nudc	Actb
		Tpm3	Nap1l4	Eprs	Adipor2	Matr3	Uqcrh.ps2	Cfap36	Gm10073
		Nfe2l2	Atp5j2	Cetn3	Dock10	Tcf12	Tceb2	Chchd6	Mir703
		Akap13		Ndufb11	Vmp1	Gm3531	Sdhc	Ezr	Ncl
		Calr		Mif	Eid1	Supt16	Ndufa12	Pnmal2	Eef2
		Clta		Senp6	Daam1	Parp1	Atp5j2	Tuba1a	Cd47
		Fos		Tm9sf3	Gng11	Srsf4	Pla2g16	BC005624	Tmsb4x
		Arpc2		Cfdp1	Limch1	Tubb2a	Ndufa8	Tctex1d2	Rps25.ps1
		Nfkbia		Sfr1	Hepacam	Gm11889	Atp6v1d	Phtf1	Gltscr2
		Rpl39			Clic4	Rbbp7	Dnase2a	Sox9	Serbp1
		Clic1			Cfl2	Ckap5	RP23.269H21.1	Ramp1	Rpl22l1
		Klf6			Selenok	Trim24	Ndufb11	Dnajb2	Akap13
		Srgn			Reep5	Smc1a	Bola3	Plekhb1	Gm12254
		Sat1			Fkbp1a	Nop58	Park7	Nudc.ps1	Rpl36al
		Anxa3			Zeb2	Cct6a	Atp5b	Trim37	Myh9
		Celf2			Ptpn11	Lgals1	Romo1	Cfap20	Sub1
		Picalm			Zdhhc20	Eny2	Cox7a2	Etfa	Rpl22.ps1
		Rps26			Slu7	Psma7	Ndufab1	Srek1ip1	Cdkn1b
		Gatm			Mid1ip1	Ola1	Uqcrh	Bola1	Eif3h
		Rpl26			Gm12346	Gm9769	Tomm7	Gstm1	Rpl37a
		Hspa5			Ckb	Rpl30	Uqcrb	Lztfl1	Ablim1
		Btg1			Hist1h2bc	Bud31	Sod1	S100b	Actr3
		Ier2			Slc38a2	Anp32b	Timm17a	Mt2	Trim30a
		Lamp2			Pik3r1	Hnrnpm	Nedd4	Kcnq1ot1	Tpm3.rs7
		Lamp1			Dst	Ilf2	Uqcrq	Hirip3	Rapgef6
		Ssr4			Nudt4	Calm3	Ggh	Pltp	Gm6863
		Gdi2			Rnf130	Gm4366	Cacybp	Cluap1	Rpl13
		Jund			Hsp90ab1	Wdr89	Ndufb7	Naaa	Cox17
		Tln1			Rap1a	RP24.389J11.1	Gm5436	Spats2l	Psme2b
		Wasf2			Lamp1	Pdcd5.ps	Emc2	Mpdz	Gm10076
		Sh3bgrl3			Ppp2r2a	Vbp1	Srek1ip1	Arl13b	Gm14586
		Actr2			Lap3	Gadd45gip1	Mrps21	Ntrk2	Iqgap1
		Ubc			Gm6977	Srsf7	Wbp5	Gm5904	Rpl31.ps8
		Tpm3.rs7			Serinc1	Gm6368	Churc1	Trp53bp2	Rpl6
		Ccnl1			Sgk1	Rac1	RP23.123D6.12	Cald1	Eif3e
		Rplp2			Wnk1	Gm8566	Grsf1	Meg3	Rgs1
		Ifnar2			Dync1li2	Sox2	Dbi	Slf1	Arpc1b
		Rplp0			Cd47	Hdgf	Atp5f1	Mt3	Arpc5
		Rpl37			Sypl	Gm10320	Cox5b	Dnajc3	Naca
		Gm10275			Fis1	Gm5805	Cfap36	Mettl7a1	Rpl18
		Gm5905			Atxn7l3b	Polr2f	Atox1	Ift20	Psme1
		Lrp1			Zfp706	Dpy30	Smdt1	Pdia4	Rps4x
		Cd63.ps			Tmem30a	Syncrip	Arl4a	Dmd	Rpl36
		Gm10288			Psip1	Kpnb1	Pdcd6	Psph	Gm7027
		Cd63			Pafah1b1	Map1b	Oxct1	Arl3	Arpc2
		Msn			Slc25a4	Serbp1	Gm6265	Cspp1	Lrrfip1
		Ppt1			Ifi27	Rps16.ps2	Atp6ap2	Mt1	Eif3f
		Gm16580			Hsbp1	Gm8292	Ndufa3	Bex3	Neat1
		Cstb			Pea15a	Gm6542	Atp1a1	Anxa5	Sfpq
		Rps19			Dynll2	Txn.ps1	Timp2	Itm2c	Tpm3
		Gm4149			Rtn3	Khdrbs1	Ndufa6	Mgst1	Skil
		Rpl41			Mat2a	Whsc1	Fam213a	Usp2	Cd53
		Gm10073			Tmem258	Baz1b	Cox6b1	Kif21a	Gm14681
		Rps20			Specc1	Fkbp3	Hibadh	Cep162	Gm8276
		Pfn1			Reep3	Hnrnpu	Mrpl42	Cntrl	Hsp90ab1
		Rps10			1810037I17Rik	Gm4617	Gde1	Ahi1	Sept11
		Coro1b			Sqstm1	Calu	Calm3	Gm2830	Ddx5
		Rpl37rt			Ktn1	Psmd14	Ndufs6	Anxa2	Eef1g
		Rpl6			Tecr	Eif3k	Atp5a1	Tusc3	Rps6.ps4
		Rps25.ps1			Tmbim6	Hpf1	Atpif1	Hmgn5	Fkbp3
		Arpc5			Ociad1	Srsf10	BC002163	Hist1h2bc	Anp32b
		Ptp4a2			S100a1	Skp1a	Cyc1	Hmgn1	H2afy
		Psme1			Rab2a	2310036O22Rik	Atp5e	Lmo4	Eif3a
		Rpl10a.ps1			Nap1l4	Brd8	Acadm	Fam204a	Rps7
		Capzb			Hsd17b12	Sept7	Oaz1	Zbtb20	Eif5a
		Psme2b			Ptn	Tuba1a	Syap1	Tceal3	Capzb
		Rps13.ps2				Cops6	Nudc	Aig1	Ddx21
		Rps24.ps3				Cct5	Slc25a5	Cnn3	Gm12183
		Rps15a				Suz12	Pkm	2810013P06Rik	Rgs2
		Akr1a1				Taf15	Scp2	Stox2	Sf3b1
		Sppl2a				Dhx9	Ndufv2	Fundc1	Ptbp3
		Rpl35a				Sept11	Tuba1b	Ptges3	Kmt2a
		Rps8				Naa38	Hsp90aa1	Timp2	Prrc2c
		Gm9794				Cct3	Cox14	Cntln	Tln1
		Cltc				Nap1l1	Zfp106	Nap1l5	Klf6
		Oxct1				Nfib	Srp14	Crip2	Eef1d
		Rps3a1				Csnk2b	Cox7c	Gm1673	Mier1
		Gm15427				Snrpd3	Myeov2	Prdx6	Jund
		Atpif1				Cct2	Acat1	Fam184a	Clk1
		Rps15				Ncl	Nenf	Fxyd1	BC005537
		Grb2				Mrps24	Sod2	Wls	Clic1
		Rpl13				Sub1	Ndufb5	Gm4617	Pfdn5
		Rpl37a				Kif2a	Ndufa5	Gm9800	Srrm2
		Zfand5				Psmd8	Eno1	Cpd	Tpr
		Rps10.ps1				Zfr	Ndufa2	Atp1a1	Myl12b
		Os9				Gm8276	Mpc2	Cacybp	Txn1
		Arhgap5				Srsf2	Cdv3	Ccng1	Cotl1
		Prpf38b				H2afy	Trim37	Clu	Rsbn1l
		Tmem50a				Rps6.ps4	Slc25a3	Atpif1	Itgb1
		Mycbp2				Gm7809	Gm12350	Glod4	Msn
		Rpl36a.ps2				Gm14681	Akr1a1	Prdx4	Btg2
		Wnk1				Tcea1.ps1	Uqcrc2	Pink1	Rock1
		Rpl31.ps8				Fabp7	Mdh2	Cystm1	Rsrp1
						Smc3	Gm15920	Pebp1	Oaz1
						Pdap1	Lrpap1	Acadm	Itsn2
						Rpl8	Swi5	Cpe	Xrn2
						Gm5835	Cox6a1	Cetn3	Tra2a
						Sf3b6	Ndufa7	Oxct1	Dek
						Gm5963	Slc25a4	Spop	Hnrnpd
						Gm6863	Cep290	Creld2	Atp5d
						Papola	Acadl	Gm12350	Ywhaz
						Rps7	Hspa4	Txn1	Myl12a
						Rpl14.ps1	Dynll2	Paip2	Mycbp2
						Rps4x	Uqcrc1	Csrp2	Syf2
						Dynll1	Atp6v1f	Akap9	Gm12338
						Gm9385	Chchd2	Cfdp1	Hnrnpab
						Nudc	Pdha1	Smim11	Psma2
						Btf3	Atp5k	Clcn4	Thrap3
						Rps20	Atp6v1g1	Gng5	Kmt2e
						Atp5g3	Atp5c1	Stip1	Ptma
						Eif1ax	Nedd8	Phpt1	
						Ppp1cb	Anp32e	Tomm7	
						Ran	Tmem256	Pla2g16	
						Gm13436	Rpl41	Chchd2	
						Pou3f3	Gm11966	Hsp90ab1	
						Snx3	Usp25	Calm1	
						Hp1bp3	Acbd5	Bag1	
						Rtf1	Mif	Nol7	
						Xrn2	Lamp2	Rnh1	
						Romo1	Glrx5	Laptm4a	
						Gm13835	Tmem59	Cxcl13	
						Gm6563	Paics	Shfm1	
						Ddx1	Gnas	Gm42418	
						Ndufb6	Gm12338		
						Ptprz1	mt.Nd5		
						Zeb1	Eif4g2		
						Fez1	mt.Nd2		
						Bclaf1	Tax1bp1		
						Gm16580			
						Cbx1			
						Tuba1b			
						Cyp51			
						Pcbp1			
						Spcs2.ps			
						Txn1			
						Rps11			
						Rpsa.ps10			
						Hnrnph1			
						Lmo4			
						Zcrb1			
						Bzw1			
						Gm9892			
						Tmem258			
						Arl6ip1			
						Cct4			
						Cct8			
						Yy1			
						Eif2s2			
						Sox4			
						Srpk2			
						Set			
						Eif3i			
						Gspt1			
						Tceb1			
						Calm1			
						Rps3			
						Zfp207			
						Pcm1			
						Hnrnpc			
						Txnl1			
						Pcbp2			
						Tmsb4x			
						Psmd7			
						Suclg1			
						Nap1l4			
						Cox5a			
						Gm6204			
						Srp72			
						Stag2			
						Pdia6			
						Dnajc2			
						Gm43712			
						Tmsb10			
						Rpl14			
						Nol7			
						U2surp			
						Psmc1			
						Map2			
						Bola2			
						Banf1			
						H3f3a			
						Rps27l			
						Hspd1			
						Minos1			
						Nme1			
						Cacybp			
						Ptma			
						Npm1			

Table S4. Lists of differentially expressed genes between PDOXs and nude brain per each major cell type. Differentially expressed genes were defined at threshold: FDR <=0.01 and |log$_2$FC|>=1

Genes are listed from the highest to lowest |log2FC|

Cell type	Astrocytes		Endothelial cells		Ependymal cells		Myeloid cells		Oligodendrocytes		OPCs		Pericytes	
Fold change	upregulated	downregulated	upregulated	downregulated	upregulated	downregulated	upregulated	downregulated	upregulated	downregulated	upregulated	downregulated	upregulated	downregulated
**Number of genes**	186	129	304	105	21	22	287	136	7	124	286	199	2	71
	Gm42418	Sfrs18	Gm42418	Plp1	Tmsb4x	Gm26924	Spp1	Sfrs18	Olig1	Igfbp5	Pdgfra	Plp1	Gm42418	Wdr96
	Cryab	Ppap2b	Rpl14.ps1	Gm26924	Gm42418	Sez6	Gm42418	Snord49b	Pdgfra	Mal	Gm42418	Apod	Gm12346	Gm26924
	Vim	Gm26924	Adgrf5	Gpr116	Lyz2	Vps8	Cxcl13	Sepp1	Gria2	Plp1	Lhfpl3	Npy		1700009P17Rik
	Tmsb4x	Gm26339	Selenop	Sfrs18	Tyrobp	Rpl35	Clec7a	Gm26924	Cspg5	Phldb2	Pnisr	Sepp1		1700026D08Rik
	Pnisr	Snord42a	Gm6977	Ttyh1	Hexb	Vimp	Gm6977	4632428N05Rik	Cacng4	Cd200	Gap43	Igfbp5		Ptplad1
	Plpp3	Xist	Gm5905	Sepp1	C1qa	D3Bwg0562e	Gm5905	Epb4.1l2	mt.Cytb	4922501C03Rik	Cspg5	Slc4a4		Stxbp3a
	Rack1	Gm3764	Gm9794	Fabp7	C1qb	Gm6625	Gm14303	Gm26339	Actb	Slc4a4	3110035E14Rik	Clca1		A330021E22Rik
	Gm6977	2810055G20Rik	Rpl37rt	Igfbp5	B2m	Calml4	Gm9794	Entpd1		Ptn	Rpl14.ps1	Gja1		Wdr52
	Gm9625	Slc38a3	Gm11478	mt.Tc	Ctss	BC023829	Rpl14.ps1	Abca9		Sash1	Gm5905	Ptn		Ccdc135
	Rpl13.ps5	D4Wsu53e	Gm4617	Fut9	C1qc	1110001A16Rik	Ccl4	D4Wsu53e		Clca1	S100a1	Nr2f2		Ccdc19
	Rps3a3	Sepp1	Gm15427	D4Wsu53e	Ctsd	0610011F06Rik	Rpl37rt	P2ry12		Mxi1	Pllp	Ccdc80		2610015P09Rik
	Gm14892	Snord49b	Gm9385	Ppap2b	Cd63.ps	Ppia	Lpl	Gpr34		Ccdc157	Slc25a5	Vtn		Ccdc104
	Gm7558	Ptplad1	Rps16.ps2	Slc4a4	Ctsb	Ccdc41	Ttr	Il7r		Tlr3	Gng3	Gm26924		Spag6
	Fth.ps2	Vegfa	Gm14303	Eltd1	Rpl14.ps1	1110059M19Rik	H2.Ab1	Ddx26b		Sepp1	Scg5	Frzb		Rps17
	Rpl14.ps1	Sc4mol	Gm5805	Dcn	Gm9794	Rpl29	Gm15427	P2ry13		Ttyh1	Ramp1	Ttyh1		Sfrs18
	Rps5	Slco1c1	Rpl32	Gm26339	Wdr89	4922501C03Rik	Fth1	Csmd3		Lifr	Rps5	Apoe		Ifltd1
	Gm12346	Adrbk2	Gm4149	Rps6	Rack1	Gm9493	Gm11478	Gm17087		Frzb	Gm9385	Hmgcs2		2510003E04Rik
	Gm12857	2610017I09Rik	Gm10080	Snord49b	Lamp1	Cox7b	Cd63.ps	Ccdc55		Wdr60	Olig2	Timp3		Gm872
	Gm13215	Slc1a2	Gm5963	Hnrnpa3	Sparc	Stk39	H2.Aa	Maf		Timp3	Rpl3.ps1	Ncam1		Ppap2b
	Gm6136	Vimp	Pnisr	Ptn	Cst3	Selt	Rack1	Gpr56		Trp53bp2	Gm12346	Inhba		2010015L04Rik
	Rps16.ps2	Zfml	Rpl3.ps1	Slc7a11	Actb	Enpp2	Cd74	Hnrnpa3		Mybpc1	Rpl37rt	Sash1		A030009H04Rik
	Gm13498	Tmem66	Adgrl4	Vimp		4930546H06Rik	Ccl3	Tmem66		Ralgps2	Gm9794	Iqub		Pax3
	Gm22567	Syne1	Rpl38.ps2	Pcdhga9			Gm16580	Ppap2b		Sfrs18	Gm11478	Col5a2		Vps28
	Gm12183	St6galnac5	Tpt1.ps3	Atp1a2			Gm11942	Zfhx3		Aspa	Tnr	Tspan15		D2Wsu81e
	Gm9385	CT030684.1	Gm9843	Abca9			Gm5963	Jund		Slc7a11	Gm11560	Stmn1		Gm6625
	Gm5805	Nim1	Rack1	Hes5			Gm4149	CT030684.1		Gja1	Olig1	Lpar1		Ccdc41
	Rn7s6	Fut9	Rps24.ps3	Slc1a3			Rps24.ps3	Zfml		Plekha4	Clu	Igf1		Selk
	Id1	Cwc22	Gm14586	Cyp2j9			Selenop	Nrip1		Rgag4	Rack1	Wls		Lsmd1
	Fth1	Gnao1	Gm6204	Timp3			Cst7	Slco2b1		Gas7	Gm15427	Sparc		6720401G13Rik
	Gm12222	Pcdhga9	Gm11966	Fam213a			Rps16.ps2	Tmem119		Ahctf1	Rsrp1	Col23a1		B630005N14Rik
	Gm5370	2610203C20Rik	Rflnb	mt.Tp			Gm9385	Vimp		Inhba	Bcas1	Slc7a11		AK129341
	Gm11560	Trim9	Gm11560	Nell2			Gm5805	Hpgds		Fmo1	Rps16.ps2	Fam213a		Gm26339
	Gm12165	S1pr1	Rps13.ps2	Scd2			Rpl3.ps1	Lrrc58		Gtpbp2	Gm4617	Sfrs18		Sept15
	Gm13340	Macf1	Rpl36a.ps2	Sash1			Rps13.ps2	Cx3cr1		Fbln2	Tmem100	Gm26339		Ankrd32
	Rpl10l	5830428H23Rik	Gm11942	Ptgds			Pnisr	Il6ra		Gm872	Stmn3	Hey2		Dpcd
	Gm11942	Ttyh1	Fn1	Vegfa			Apoe	Cep110		Cldn5	Gm12338	Phldb2		CT030684.1
	Rpl17.ps4	Ank2	Gm16580	Gm17087			Fn1	Gm13476		Mdk	Calm1	Cd59a		Gm12942
	Gm4332	4930546H06Rik	Wdr89	Mllt4			Ccl6	Mertk		Cyp4v3	Nap1l5	Gdpd4		Gm5918
	Gm11810	Ctage5	Gm10073	2810055G20Rik			Gm6204	Wapal		Pla2g16	Bex3	Fmo1		Pet112
	Rps15.ps2	Dclk1	Gm4332	Slc1a2			Gm8995	Naalad2		Scd1	Id2	Pla2g16		Sc4mol
	Gm14450	Malat1	Rsrp1	Colec12			Rpl36a.ps2	RP24.312B12.1		Wls	Pcdh15	Mfap3l		2410018M08Rik
	Gm12696	Clmn	Rps26	Wapal			Cd52	Rps23		Pbxip1	Rpsa.ps10	Atp1a2		I7Rn6
	Gm6368	Acss1	Rplp0	Mdk			H2.K1	Snord42a		Fam213a	Ranbp1	Mybpc1		BC023829
	Wdr89	Itih3	Slc38a5	Gm14964			Kctd12	Rpl5		Igf1	C1ql1	Mal		Ccdc37
	Gm2225	Vwa1	Gm9800	Mboat2			Epb41l2	Ppp1r9a		Dab2	Cd81	Col27a1		Epb4.1l4a
	Gm5963	Pitpnc1	Rplp1	Rpl15			Rpl10a.ps1	Smap2		Col5a2	Ostf1	Dnajc1		9330101J02Rik
	Ttr	Fam21	Gm6863	Suv420h1			Rps5	Jhdm1d		Slc43a2	Gria3	Pex11a		Epb4.1l2
	Gm11472	Cep110	Rpl31.ps8	Fbln2			Ch25h	Rps29		Lysmd3	Igfbp2	Abca8a		4922501C03Rik
	Rps12.ps19	4930402H24Rik	Gm12338	Rpl13a			Gm4617	Rpl21		Fstl1	Cox8a	Dock7		2700089E24Rik
	Gm17909	Appl2	Rpsa.ps10	Atp13a5			Gm14586	2810055G20Rik		Frg1	Dynll1	Fbln2		1700094D03Rik
	Rac1	Tril	Rps10.ps1	Rpl5			Gm4332	Tanc2		Hey2	Nxph1	Rnd3		Bai3
	Gm12152	Epb4.1l2	Gm10288	Sept15			Rsrp1	Son		Ncam1	Selenok	Filip1l		Sepn1
	Cd63.ps	Mycbp2	Gm6265	4933426M11Rik			Rpsa.ps10	Slc16a6		Atp8a1	Alcam	Golgb1		Lphn3
	Gm5835	Atp1a2	Scgb3a1	Gja1			Ctsd	D15Ertd621e		Acss1	Gm4149	Thrsp		2310044G17Rik
	Gm9843	Zfp949	Fth1	Gpm6b			Lilrb4a	RP24.288C12.6		Cd82	Actb	2610203C20Rik		Rpl24
	Cald1	Acsl6	Ctla2a	Kcnj16			Ifi27l2a	Arid4a		Glul	Rps26	Gas7		Nim1
	Id3	Gja1	Rps5	Kmt2a			Rps26	A430104N18Rik		Mfsd6	Gm14586	Glul		2410066E13Rik
	Hspd1.ps3	Timp3	Gm10275	Tmem66			Rps25.ps1	Chd9		Selk	Gm6863	Sdc4		Gm10311
	Gm2178	Son	Apold1	Spag9			Tpt1.ps3	Numb		Lgi1	Tpt1.ps3	Cry2		Gm13225
	Gm9794	Ccdc104	Gm12183	Ptms			Mt1	Srgap2		Gramd1b	Emid1	Scd1		5430417L22Rik
	Map1b	Btbd17	Gm7809	Gprasp1			Gm6030	Elmo1		Npy	Ndufa4	Hsd17b11		Gm17388
	Gm44010	Ddhd1	Afdn	Morf4l1			Calr.ps	Pwwp2a		Sco1	Igfbp3	Vimp		Gpr125
	Rps19.ps6	Suv420h1	Gm15500	Rpl10			Lyz2	Ctage5		Slc25a13	Car8	Mllt4		Grlf1
	Gm5778	Ppia	Gm5835	Zbtb20			Gm10288	Col27a1		Adam23	B2m	Gucy1b3		Leprel2
	Gm4204	Slc1a3	Rps25.ps1	Hspa8			Wdr89	Ogfrl1		Tgfbr1	Rps13.ps2	Fam3c		Trp53rk
	Selenop	Ttc14	Gm9892	Htra1			Gm10073	Mycbp2		Fgd4	Gm14303	Gpr56		Zbed6
	Gm15159	Slc7a10	Calr.ps	S1pr3			H2.D1	Scamp2		Tmem66	Gm6977	Abca5		Fam101a
	RP23.390K13.4	Nrcam	Rpl18.ps1	Rpl23a			Gm15500	Lpar6		Nr2f2	Pcsk1n	Edil3		Fam115a
	Gm11478	Pdcd4	Rpl10a.ps1	Kcnj10			Rpl31.ps8	Marcks		BC016423	2900011O08Rik	Huwe1		9830147E19Rik
	Cxxc5	Pla2g7	Gm8894	Ccdc80			Rpl18.ps1	Erbb2ip		Zmat1	Gm5805	Ift57		Rom1
	Bex3	Fam115a	Gm8292	Rps23			Apoc1	Rbm5		Gm26339	Ndufb6	Aldh2		Zfp191
	Gm16580	Gyk	Rps6.ps4	Ncam2			Gm5835	Tmem100		Aim1	Cd63.ps	Ahcyl2		
	Gm15896	Csgalnact1	Selenom	Gnb2l1			AU020206	Rps6		Dock7	Ndufa8	Efnb2		
	Gm27046	Nrxn1	Ly6c1	Inhba			Gm9843	Macf1		Tiam1	Rpl22l1	Grik3		
	Gm29228	Hlf	Eif1.ps1	Cwc22			Gm8276	Ythdc1		4931406C07Rik	Banf1	Mgst1		
	Cnot2	Rorb	Gm12254	Prrc2c			Rplp1	Ssh2		Vtn	Ddah1	Hmgn2		
	Gm9294	D14Abb1e	Cd34	Ncam1			Tyrobp	Qk		Osbpl11	Ttr	Gm12688		
	Gm11517	Flrt1	Rps15a.ps5	Zfml			Il1b	Hmgb1		Phf17	Gpm6a	Sparcl1		
	Rpl34.ps2	Frmd4a	Rps11	Gpx4			Ccrl2	Med12l		Hspa8	Scrg1	Fut9		
	Gm9521	Fjx1	Ttr	S100b			Kcnq1ot1	Arl4c		Cpt1a	Wdr89	Mest		
	Gm8513	Acin1	Tmsb4x	Rps17			AC149090.1	Cwc22		5830418K08Rik	Gm12222	Map4k4		
	Gm4321	Arhgap5	Gm3531	Xist			Rpl32	Dusp6		Tpr	S100a16	Ptgds		
	Gm15427	Hnrnpa3	B2m	4932438A13Rik			Rps10.ps1	Sall1		Apoe	Gm12696	Plcb4		
	Gm18014	Mfge8	Ly6e	Ahcyl2			Axl	Dip2b		Tmem245	Tuba1a	Slc27a1		
	Gm12482	C030037D09Rik	Rps9	RP24.288C12.6			Id2	Rps27a		Hadha	Cdo1	Mex3a		
	RP23.408J7.2	Tnik	Rps26.ps1	Ddit3			Vim	Frmd4a		Atp1a2	Tceal3	Grin3a		
	Gm17511	Selk	Selenok	Ppia			Ctsb	Rps28		Nnat	Gm9892	Cyp2d22		
	Gm6543	Ubn2	Gm12346	Rps27			Itgax	P4ha1		Snx24	Gm6204	Rgag4		
	Matr3.ps2	Slc4a4	Cdkn1a	Mga			Vsir	Morf4l1		Dnajc1	Gm5778	Fabp7		
	Gm7308	Tmed5	Rpl8	Ccdc104			Rps20	Olfml3		Plk1s1	Nrxn1	Cd200		
	Gm5514	Cadm2	Selenof	Rps12			Cd300c2	Hspa8		Clca2	Plpp3	Wapal		
	Gm4366	Nwd1	Gm12350	Kdm5b			Gm6863	Prrc2c		Ccdc39	Tmem176b	Syne2		
	Rsrp1	Dio2	Gm15772	Igfbp2			Ccl12	Fau		Stmn1	Gm5835	Ccnd2		
	Id2	P4ha1	Igfbp7	Diap2			Gm11560	Map4k4		Cd55	Calr.ps	D3Bwg0562e		

	Rps3	Acsl3	Pomp	Rpl21			Gm14681	Safb		2700089E24Rik	Cox6b1	Nid1		
	S100a6	Tex9	Gm6472	Apod			Ints6l	Arhgap5		Mfap3l	Mmp15	Suv420h1		
	Gm3531	Myo6	Sparc	Nupr1			mt.Cytb	Acin1		P2ry12	Dynlt1.ps1	Hadha		
	Gm7618	Sparcl1	Slc25a5	Wdr60			mt.Nd1	Ifngr1		Fabp7	Cfap20	Tlr3		
	Gm5905	Mfn1	Gm7027	Tmx4			Cd83	Bin2		Fut9	Ccnd1	Clca2		
	Gm12912	Gm973	Gm6368	Son			Gm10275	Srrm2		Fam3c	Gm5963	Dtx4		
	Gm13815	Slc6a11	Gm14681	Ednrb			Gm9892	Tsc22d4		Trim41	Cfap36	Abcb1a		
	Rps11	Kcnj16	Ppfibp1	Stmn1			Rps15a.ps5	Selplg		Mecp2	Tagln2	Gm6483		
	Gm11488	Ythdc1	Rab1a	Casc4			Gnas	Zfp292		Rbms3	Gm6265	Gpd1		
	Pabpc1	Mdn1	Rpl22.ps1	Asph			Rpl38.ps2	Mtdh		Scd2	H2.D1	Slc1a3		
	Dclk2	Ntrk2	Gm4366	Nr2f2			Flt1	Ccr5		P2rx4	Rpl36a.ps2	Slc13a3		
	Gm12020	Sycp2	Rpl9.ps7				Gm7027	Adap2		E130311K13Rik	Hspd1.ps3	Gpd2		
	Gm15773	Trpm3	Gm8995				Selenos	Abcb4		Lrig1	Grm5	Zbtb20		
	Calm2	Wapal	Gm15421				Ank	Vwa1		Rnd3	Pcp4	Itsn2		
	App	AI464131	Hsp25.ps1				Gm12338	Ivns1abp		Paqr6	Cacng4	Chd9		
	Gm6204	Mmp14	Gm14539				Rps6.ps4	Plcl2		Ltn1	Meg3	Sat1		
	Calr.ps	Pfkp	Plpp3				B2m	Pde3b		Lama4	Dnm3	4922501C03Rik		
	Vcan	Neat1	Gm6563				Srgn	Sox4		Nek9	Rab31	Hnrnpa3		
	Spp1	Nebl	Anp32.ps				Gm12346	Fchsd2		Son	Slc38a3	Ptms		
	Marcks	Chrdl1	Tpm3.rs7				Atf3	Rps27		Slc16a1	Pdcd5.ps	Tspan18		
	Fos	Dtna	Gm12696				Cd63	Malat1		B4galt6	Ubb	Arhgap5		
	Hacd3	Aifm3	Saraf				Atox1	Rock2		Zbtb20	Gm10288	Magt1		
	Gm6863	Utp14b	Ctsd				Ifi202b	Ttc14		Ccdc171	Ppib	Son		
	Gm21399	Peg3	Gm5436				Cstb	Tgfbr1		Nexn	Dpp6	Gbp7		
	Atp5e	9330159F19Rik	Gm5865				Nfkbia	Ubn2		Colgalt2	Oxct1	Nupr1		
	Ctsd	Sfxn5	S100a11				Rps26.ps1	March1		Sparcl1	Serpine2	Nfat5		
	Gm12892	Slc7a11	Cyb5a				Ctsz	Pnn		Calr	Arl2bp	9330159F19Rik		
	Golim4	Prpf4b	RP23.123D6.12				Ms4a6c	Rbm25		4930480K23Rik	Gm3531	Cyp39a1		
	B2m	Slitrk2	Cd63.ps				Gm12254	Rpl35		Kdm2a	Gm11966	Igfbp4		
	Jun	Gprasp1	Rpl39				Rps10.ps2	Ppia			Rtn1	Ccdc104		
	Selenos	Ankrd11	Ybx1.ps2				Npc2	Pmepa1			Gng11	Gm6625		
	Gfap	Cxcl14	Shfm1				Atp5e	Serinc3			Rpl10a.ps1	Snx29		
	Gm11966	Gapdh	AU021092				Ifi44	Rpl10			Arpc2	Tec		
	Sept7	Kcnk1	Gm8276				Csf1	Nav3			Rps26.ps1	Cmtm5		
	F3	Ccdc50	Gm13835				Gm15772	Gnb2l1			Agpat5	Mboat2		
	Hacd2		Gm5644				Tnf	Rpl18a			Ndufs4	Kdm5b		
	Gnas		Gm8730				Mia2	Rpl17			Gm5865	Abca1		
	Cct2		Smim10l1				Gm15421	Ptgs1			Phlda1	RP24.288C12.6		
	Rps20		Gm5778				Rpl9.ps6	Ywhah			Rps24.ps3	M6pr		
	Rpl32		Gimap4				Plpp3	Hmha1			Cox4i1	Nfe2l2		
	Gatm		Gm6733				Rgs1	Basp1			Tmem179	Zfhx3		
	Rpl8		Vim				Calm1	Srrm1			Ahi1	Sort1		
	Sbds		Pdcd5.ps				Rpl39				Fis1	Wnt6		
	Skp1a		Cfap36				Rpl22.ps1				Selenof	Tmem132b		
	Msmo1		Rps3				Gm11966				Eif3i	Frmd4a		
	Snx3		Tspan13				Gm7266				Ly6h	Phf17		
	Ybx1		Rpl18.ps2				Gm12183				Rplp1	Entpd1		
	H3f3b		Nsrp1				Cox4i1				Ndufa7	Agt		
	Eif3k		Rps19.ps6				Ctsl				Gm16580	Cldn5		
	Sox4		Wfdc1				Gm9800				Gm15500	Crip2		
	Hint1		Ifitm3				Cox6a1				Gm4204	Plcd4		
	Calm1		Selenow				Rps15				Omg	Lix1		
	Psma7		mt.Cytb				Dbi				Ednrb	Ctbp2		
	Fez1		Tcea1.ps1				Rps11.ps1				Gria2	Cyp2j6		
	Rheb		Higd2a				Cxcl2				Rpl36al	Baz2b		
	Nucks1		Ier2				Saraf				Tspan13	Socs2		
	Eef2		H2.K1				Rps19.ps6				Gm6368	Atp8a1		
	Cbr1		Gm7266				Cox6b1				Cox7b	Cyp4v3		
	H2.D1		Selenos				Hacd2				Il1rap	Snord49b		
	Tcf12		Gm12481				Wfdc17				Commd6	Stx5a		
	Eef1a1		Gm15920				mt.Nd4				Gm14681	Cep85l		
	Matr3		Cox7a2				Capg				Mpc2	Nell2		
	Rps9		Gpx1				Prdx1				Atp5g3	Fxyd1		
	Anapc11		Rps10.ps2				Pld3				Ndufa1	Parp4		
	Rps14		mt.Nd1				Lgals3bp				Cct7	Zfp191		
	Gtf2i		Cox4i1				Postn				Nsg1	2310022B05Rik		
	Bcan		Actb				Gm6136				Psmb1	Bmp7		
	Rps4x		Igf1r				Gm4366				Cfl1	Hspa8		
	Ubb		Ostf1				Gm8730				Prdx2	Foxo1		
	Hnrnpd		Mia2				Grn				Dbi	Eps15		
	Dync1li2		Fkbp1a				Slc25a5				Sulf2	Cdh8		
	Rps21		Eif3j2				Rplp0				Dtd1	Paqr6		
	Rps15		Rps15.ps2				Gm4604				Cspg4	Slc43a3		
	Tpm3.rs7		Ube2d3				Fcer1g				Pcyt1b	Chd2		
	Rpl4		Arpc2				Cd9				Dscam	Fnbp1l		
	Rplp1		Gm4604				Zfp638				Mlf2	Notch2		
	Dstn		Tonsl				Cox8a				Gm1673	Sipa1l1		
	Sec11c		Ramp2				Rps3				Nop10	Tmtc2		
	Cnn3		Pabpc1				Pabpc1				Uqcrq	Lipg		
	Nasp		Gm14253				Eif1.ps1				Atxn10	Prrg3		
	Ppp1cb		Hint1				Gusb				Ckb	Scd2		
	Vapa		Id3				Cntrl				A730017C20Rik	Megf10		
	Ilf3		Gm9840				Gm6265				Serpina3n	Hivep3		
	Pcbp2		Slc16a4				Ier3				Aplp1	Slc25a13		
	Cct6a		Rps15				Lgals1				Higd2a	Ncoa7		
	Ywhab		Rpl9.ps6				Tmsb10				Cxcl14	Synm		
	Pcbp1		Pglyrp1				Gpx1				Rps9	Leprel2		
	Ctss		Gstp.ps				Csf2ra				Dner	RP23.32A8.1		
	Zhx1		BC002163				Gm43712				Pcbp2	Prss56		
	Ywhae		Tpm4				Hpf1				Olfm1	Fam107a		
	Arpc2		Epb41l2				Gm6368				Cryab	Tiam1		
	Rpl23		Naa38				Rps15.ps2				Kcnd2	Gm4076		
			Gm12020				Rps9				Znhit6	CT030684.1		
			Ndufa11				Anxa5				Gtf2h5	Tmem47		
			Calm1				E230029C05Rik				Ndufc2	Cpne8		
			S100a16				Gm5644				Opcml	Ccdc173		
			Gm6542				Uqcrq				1810037I17Rik	Erbb3		
			AC149090.1				Rpl18.ps2				Uchl3	Vwa1		
			Ssr2				Gm6472				Cdh13	Cgnl1		
			Rpl37				Wapl				Sfr1	Tnfrsf19		

			Gm13436				Rps19				Gm2a	Itga6		
			Gm10039				Nsrp1				Cp	Zfp609		
			Grrp1				Mir703				Tubb2a	mt.Rnr1		
			S100a13				Sh3bgrl3				3632451O06Rik	Alx4		
			Tpm3				Rpl9.ps7				Gjc3	Prrc2c		
			Gm7407				Gm10080				Atp6v1g1			
			Fam204a				Rps11				Lnx1			
			Myl12a				Cotl1				Adam9			
			Gm10031				Gm8606				Pea15a			
			Txn.ps1				Lyzl4				Sfxn1			
			Atp5j				H2.DMa				Pfn1			
			Psma5				Plau				Uqcrc1			
			Ccdc186				Akr1a1				Srp14			
			Gm6136				Gm12020				Cdk4			
			Ifi27l2a				Cybb				Itgb8			
			Rplp2				Psme2b				Deb1			
			Dynlt1.ps1				Ssr4				Megf11			
			Sf3b6				Cd72				Cox6c			
			Zfp638				Selenof				Myo5a			
			Rps11.ps1				Gm15148				Pou3f1			
			Wapl				Zfas1				Tacc2			
			Icam2				Ndufa2				Atp5j			
			Rac1				Gm9294				Gnb4			
			Gm14513				Shfm1				Cadm2			
			Bag1				Il4i1				Atp6v1a			
			Gm43712				Gm13436				Gria4			
			Gm21399				Gm12481				Sept7			
			Tmem252				Erbin				Brinp1			
			Rps15a				Ppp1r15a				Eif5a			
			P4hb				Selenok				S100a13			
			Lgals1				Rpl22l1				Ndufb8			
			Tmsb10				Eif3f				Pgp			
			Pcbp1				Ifitm3				Psmb3			
			Uqcrh.ps2				Rps21				Bcan			
			Gm11964				Zeb2os				Gm10250			
			H2.D1				Gm1966				Ptprn			
			RP23.269H21.1				Gm14513				Krtcap2			
			Ilk				Ctsh				Atp5b			
			Cox6b1				Mt2				Sri			
			Lmo2				Glmp				Cask			
			Rasip1				Gm10076				Tceb2			
			Psme2b				Pdcd5.ps				Mt3			
			Erbin				Pfdn5				Ppp1r14c			
			Zmat2				Cxcl16				Park7			
			Gm9294				Crip1				C4b			
			Gata2				Gm6394				Sox6			
			Oaz1.ps				Cspg4				Pcdh9			
			Anp32b.ps1				Ier2				Uqcrb			
			Atox1				Myeov2				Rgs20			
			Rras				Cd68				Hint1			
			Rhoj				Gm8692				Ugp2			
			Ly6a				RP24.389J11.1				Mdh2			
			Mydgf				Gm4204				6330403K07Rik			
			Rps19				Gm3531				Ndufab1			
			Uqcr11				Gm14539				Mt1			
			Esam				Lgals3				Atp6v0e			
			Rps20				Nfkbiz				Rpl32			
			Gm8129				Gm8292				Pomp			
			Gm8566				Gm11361				Rps3			
			Psmb2				Snhg8				Rpl39			
			Cldn5				Ifi207				Psma7			
			Bvht				Rps14				Isoc1			
			Snhg8				Gm12696				Zcchc24			
			Gm12231				Ybx1.ps2				Bzw1			
			Junb				2010107E04Rik				Cdc42			
			Hpf1				Diaph2				Cox6a1			
			Chchd2				Mydgf				Lrrtm3			
			Ndufa8				Bcl2a1b				Pafah1b1			
			Pea15a				Mpeg1				Phyhipl			
			Alas1				Cox7a2				Ptpre			
			Anxa2				Rpl8				Ran			
			Hspd1.ps3				Spcs2.ps				Gm10260			
			Csnk2b				Cox6c				Cox7a2			
			Suclg1				Gadd45b				Wbp5			
			Gm16399				Hspd1.ps3				Acat1			
			Id1				Atp6v1f				Cd9			
			Ost4				Rab1a				Rps14			
			Eea1				Ccl9				Atpif1			
			Gabarapl1				RP23.123D6.12				Atp5a1			
			Dpm3				Arpc1b				Tmem176a			
			Spcs2.ps				Tspo				mt.Cytb			
			Gng5				Txn1				Phactr3			
			Txndc17				Rpl39.ps				Atp5e			
			Sec61b				Ndufa1				Sdhb			
			Gm6394				Psmb8				Ndufa2			
			Atp5o				Eef2				Rps20			
			Rbx1				Gm15920				Ywhab			
			Snx3				Ifi211				mt.Nd1			
			Tcaf1				Cd14				Gnas			
			Cstb				Hcar2				Tpm1			
			BC028528				H2.T23				Atp5c1			
			Gm2000				Bcl2a1a				Fermt2			
			Gm9392				Mif							
			Mrps5											
			Uri1											
			Gm8692											
			Rpl7.ps7											
			Dynll1											
			Snrpb											
			Gm8606											
			Srgn											
			Lrp8											
			Gm5881											
			Col4a2											
			Anapc11											
			Gm11361											
			RP24.389J11.1											
			Cox8a											
			Myh10											
			Gm12966											

Table S5. Characteristics of myeloid clusters identified in DROP-seq data. Top 4 gene ontology (GO) terms were listed. Marker genes for each cluster were defined as differentially expressed genes between clusters at threshold: FDR <=0.01 and $lo_{g2}FC$>=0.5
Genes are listed from the highest to lowest log_2FC

Cluster number	CL0	CL1	CL2	CL3	CL4	CL5	CL6	CL7	CL8
Ontogeny	Ho-Mg	Ho-Mg	Mg-TAMs	Mg-TAMs	Mg-TAMs astrocytic-like	Mg-TAMs EC-like	Mg-TAMs cycling	Mo	BAMs
Top 4 GO terms	GO:1902075 cellular response to salt	GO:0008380 RNA splicing	GO:0002282 microglial cell activation involved in immune response	GO:0030595 leukocyte chemotaxis	GO:0042063 gliogenesis	GO:0001944 vasculature development	GO:0000278 mitotic cell cycle	GO:0045087 innate immune response	GO:1904646 cellular response to amyloid-beta
	GO:0030099 myeloid cell differentiation	GO:0048024 regulation of mRNA splicing, via spliceosome	GO:0030162 regulation of proteolysis	GO:0002252 immune effector process	GO:0007610 behavior	GO:0008360 regulation of cell shape	GO:0006325 chromatin organization	GO:0035456 response to interferon-beta	GO:0001816 cytokine production
	GO:0002521 leukocyte differentiation	GO:0060009 Sertoli cell development	GO:0055094 response to lipoprotein particle	GO:0006959 humoral immune response	GO:0031175 neuron projection development	GO:0030335 positive regulation of cell migration	GO:0051984 positive regulation of chromosome segregation	GO:0097193 intrinsic apoptotic signaling pathway	GO:0006954 inflammatory response
	GO:1901214 regulation of neuron death	GO:0048872 homeostasis of number of cells	GO:1905954 positive regulation of lipid localization	GO:1901796 regulation of signal transduction by p53 class mediator	GO:0055082 intracellular chemical homeostasis	GO:0045765 regulation of angiogenesis	GO:0006259 DNA metabolic process	GO:0019884 antigen processing and presentation of exogenous antigen	GO:0042742 defense response to bacterium
Marker genes	Jun	Luc7l3	Cst7	Spp1	Sparcl1	Ly6c1	Mki67	Ly6c2	Mrc1
	Egr1	Malat1	Spp1	Cst7	Clu	Cldn5	Top2a	Plac8	Dab2
	P2ry12	Prpf4b	Ccl6	Ccl4	Slc1a2	Itm2a	Cenpf	H2.Eb1	Pf4
	Tmem119	Luc7l2	Cxcl13	Cxcl13	Ptn	Igfbp7	Cenpe	Lyz2	F13a1
	Fos	Rbm5	Lpl	Apoc1	Mt2	Abcb1a	Prc1	Il1b	Ms4a7
	Gpr34	Pnn	Apoe	Tyrobp	Atp1a2	Ptprb	Smc4	Ms4a4c	Wfdc17
	Ier5	Arid4a	Cd63	Ccl3	Ttyh1	Bsg	Hmmr	Ifitm3	Cp
	Jund	Golgb1	Tyrobp	Cd52	Plpp3	Adgrf5	Smc2	Lgals3	Wwp1
	Rhob	Cx3cr1	Cd63.ps	Cd63	Aldoc	Spock2	Kif20b	Tgfbi	Maf
	Btg2	Ythdc1	Ctsd	Ch25h	Ttr	Flt1	2810417H13Rik	Ly6a	Stab1
	Zfp36	Smap2	Ctsb	Ccl6	Cpe	Ctla2a	Incenp	S100a11	Gas6
	Selplg	Akap9	Creg1	Cd9	Scg3	Slco1a4	Tpx2	Crip1	Fcgr2b
	Cx3cr1	Atrx	Cd9	Ctsd	Ptprz1	Slco1c1	Birc5	H2.Ab1	Selenop
	Junb	Acin1	mt.Nd1	Rpl11	Gja1	Pltp	Ezh2	Srgn	Lyz2
	Siglech	Qk	Ctsz	Ctsz	Mt1	Cxcl12	Knl1	Fxyd5	Igf1
	Cst3	Arglu1	mt.Cytb	Lpl	Aqp4	Sptbn1	Kif15	AW112010	Ehd4
	Srgap2	Ivns1abp	Mpeg1	Ctsb	Slc1a3	Slc2a1	Nusap1	Mndal	Pla2g7
	Abca9	Ncor1	Npc2	Ccl12	Slc4a4	Ly6a	Cdca8	Isg15	Cybb
	Il6ra	Prrc2c	Trem2	Grn	Cald1	Gng11	Ckap2l	H2.Aa	Ms4a6b
	P2ry13	Jmjd1c	Grn	Ctsl	Fxyd1	Pcp4l1	Ube2c	Msrb1	Snx6
	Olfml3	Rdx	Ctsl	Rpl37	Fabp7	Sdpr	Kif23	Gbp2	Ifitm3
	Ptgs1	Pnisr	Rpl32	Cd74	Gpm6b	Esam	Aspm	Ifi204	Ifi202b
	Ubc	Rsrc2	mt.Nd5	Cd68	Mt3	Ablim1	Hmgb2	Cdkn1a	Lgals1
	Tgfbr1	Phf14		Lgals3bp	Dbi	Slc39a10	Diaph3	Pim1	Apoe
	Pde3b			C1qa	Ednrb	Egfl7	Pbk	S100a6	
	Ccr5			Rpl31	Htra1	Tm4sf1	Tuba1b	Slfn2	
	Hpgds			Rpl13	Acsl3	Car4	Cdk1	Vim	
	Ivns1abp			Syngr1	Gpr37l1	Tsc22d1	Cenpa	Cebpb	
	Elmo1			Gm11942	Cspg5	Adgrl4	Ccnb2	Iqgap1	
	Golm1			Gm10260	Ntrk2	Kitl	Tubb5	Cd14	
	Mertk			Rpl19	Prnp	Id3	Mis18bp1	Samhd1	
	Zfhx3			Rps19	Atp1b2	Ramp2	Spc25	Ifi211	
	Rnase4			Cd63.ps	Ndrg2	Pecam1	Clspn	Slfn5	
	Arhgap5			H2.D1	Gstm1	9430020K01Rik	Kif11	Tmsb10	
	Mef2c			Rps24	Nrxn1	Mal	Lig1	Ifi27l2a	
	Maf			Apoe	Syt11	Col4a1	Tyms	Ms4a6c	
	Scamp2			Rpl13a	Bcan	Palmd	Tacc3	Prdx5	
	Mef2a			C1qc	Asrgl1	Golim4	Dek	Ms4a7	
	Csf1r			Rps7	Gria2	Ccdc141	Anln	Txn1	
	Marcks			Gm14303	Dclk1	Itih5	Ndc80	Ms4a6b	
	Serinc3			Rpl37a	Luzp2	Edn1	Cep55	Calm1	
	Lgmn			Rps15a	Scd2	Slc16a1	Nuf2	Lilrb4a	
	Srrm2			Ank	Lsamp	Epas1	Tmpo	Cstb	
	Itgam			Hexa	S100b	Nes	Ncapg	Cd74	
	Son			Rpl39	Timp3	Utrn	Gm11223	Gm6977	
	Malat1			Eef1a1	Prdx6	Ppfibp1	Atad2	Clec7a	
	Hexb			Pld3	Tmem47	Selenom	Stmn1	Cybb	
	Rbm39			Fcer1g	S1pr1	Fam13c	Cks1b	Lgals1	
				Mpeg1	Id3	Ptprg	Hirip3	Fth1	
				Ctsh	Ntsr2	Id1	Sgol2a	Psmb8	
				Ndufa1	Tspan7	Sgms1	Ccl12	Ifi207	
				B2m	Fermt2	Ptn	Nucks1	Msr1	
				Rpl18a	Pla2g7	Fn1	Anp32b	Nampt	
					Fam213a	Timp3	Bub1	Sp140	
					Mfge8	Slc6a6	Cbx5	Tpt1.ps3	
					Tuba1a	Arhgap29	Selenoh	Ccr1	
					Gpm6a	Cd59a	Pttg1	Bcl2a1b	
					Nedd4	Fermt2	Arl6ip1	Rpl34	
					Sox2	Kdr	Hist1h1e	Pla2g7	
					S100a6	Ets1	Pcna	Bst2	
					Ckb	Crip2	Usp1	Ifi202b	
					Tpm1	Foxq1	Tuba1c	Ifrd1	
					Vim	App	Ranbp1	Emp3	
					Zbtb20	Vwa1	Anp32e	Wfdc17	
					S100a1	Abcg2	Cxcl13	Cxcl16	
					Sdc4	Cp	H2afz	Ifi30	
					Gm42418	Maoa	Gm4617	Lst1	
					Kif1b	Dynll1	Whsc1	Tagln2	
					mt.Rnr1	Aplp2	Rangap1	Stat1	
					Myo6	Ece1	Hnrnpab	Fcgr2b	
					Tspan3	Col4a2	Ran	Shfm1	
					Sept7	Ifitm3	Ckap5	H2.K1	
					Ldhb	Apod	Rad21	Psme2b	
					Slc25a4	Fnbp1l	Ccdc34	Rps2	
					Cbx5	Alas1	Tagln2	Anxa5	
					Agpat5	Iqgap1	Gm8203	Hspa8	
					Psip1	Ifitm2	Hjurp	Psmb9	
					Nfia	Osbpl1a	Lgals1	H2.DMa	
					Add3	Tceb2	Nasp	Ms4a6d	
					Ccdc88a	Actn4	Dnajc9	Sh3bgrl3	
					Sptbn1	Ctnnb1	Anp32b.ps1	Btg1	
					Cfap36	Tpm1	Fam111a	Tspo	
					Serpine2	Prnp	Hpf1	Sh3bgrl	
					Glud1	Grap	Tubb4b	Mcl1	
					Skp1a	Igf1r	Pcna.ps2	H2.T23	
					Gm12346	Wbp5	H2afv	Rps18	
						Tmsb10	Ifi27l2a	Ccrl2	
						Tspan13	Smchd1	Taldo1	
						Hip1	Smc1a	Cd52	
						Col4a3bp	Hmgn1	Rpl35a	
						Afdn	Txn1	Trim30a	
						Luzp1	Lmnb1	Tpm4	
						Sparcl1	Gm4204	Cyba	
						Snx3	Cpd	Pfn1	
						Mt2	Smc6	Ldha	
						Rpl36a.ps2	Gm9800	Prdx6	
						Anxa2	Cdkn1a	H2.DMb1	
						S100a13	Adamts1	Gm9794	
						Myl12b	Ncl	Alox5ap	
						Nedd4	Psip1	B2m	
						Tmem256	Tpr	Ptprc	
						Rps6.ps4	Nop58	Rps27l	
						BC002163	Snrpd1	Pkm	
						Calm1	Larp7	Rps15a	
						Igf1	Xpo1	Gm15427	

						Myl12a	Aimp1	Rps24.ps3	
						Atox1	Uchl5	Rps13.ps2	
						Cuta	Hn1	Rps3	
						Dynlt3	Smarcc1	Psme2	
						Rpl38.ps2	Ssrp1	Rps16.ps2	
						Gm8186	Prdx1	Myl12a	
						Crip1	Banf1	H2.D1	
						Gm7809	Rif1	Psma7	
						Hes1	Brd8	Mif	
						Selenop	Tpm4	Prdx1	
						Gm5436	Ybx1	Rplp0	
						Ahnak	Supt16	Sdcbp	
						Syne1	Ifi30	Actr3	
						Gm8894	Snrpa1	Arpc5	
						Gas6	Pa2g4	Psma2	
						Shfm1	Ptma	Gm9843	
						Gm6265	Fkbp3	Wdr89	
						Ctnna1	Hnrnpu	Cox17	
						Gba	Pbrm1	Sub1	
						Cd34	Cox5a	Rel	
						Ndufb5		Ctsc	
						Ifnar1		Rpl39	
						Cfdp1		Gm6472	
						Glud1		Rps9	
						Lima1		Rpl22	
						Gm4617		H3f3b	
						Atp5j		Lrrfip1	
						Gm3531		Gm5905	
						Capns1		Sec61b	
						Atp5d		Cd47	
						Uqcc2		Gm9385	
						Arhgef12		Gm12254	
						Nampt		Rpl18.ps1	
						Gm4149		Lcp1	
								Chmp4b	
								Rps15a.ps5	
								Rps26	
								Fosb	
								Rps14	
								Gpx1	
								Gm4332	
								Gm5963	
								Arpc3	
								Aldoa	
								Gm15500	
								Rps10.ps2	
								Rpl36al	
								Myl12b	
								Psme1	
								Rpl37rt	
								Gm6030	
								Gm5805	
								Hif1a	
								Gm5835	
								Rps3a1	
								Atox1	
								Capg	
								Slk	
								Rpl38.ps2	
								Ly6e	
								Neat1	
								Shisa5	
								Gm10250	
								Psap	
								Rps5	
								Nfe2l2	
								Gm8995	
								Gm6204	
								Gm8730	
								Rack1	
								Rpl9.ps6	
								Rps10.ps1	
								Nfkbia	
								Rpsa.ps10	
								Rps11	
								Npc2	
								Fcer1g	
								Gm14586	
								Rps20	
								Rps19	
								Litaf	
								Arpc2	
								Gm4149	
								Tpm3.rs7	
								Rpl8	
								Tax1bp1	
								Rps21	
								Atp5h	
								Rpl36a.ps2	
								Gm10275	
								Rpl31.ps8	
								Cox7a2	
								Rpl4	
								Spop	
								Rpl14.ps1	
								Gm4204	
								Rps15	
								Chmp2a	
								Gm11478	

Table S6. Lists of differentially expressed genes between PDOXs,GL261 and their respective normal brain controls (Nu-NB, B6-NB) per each myeloid cell type.
Differentially expressed genes were defined at threshold: FDR <=0.01 and |log2FC|>=1
Genes are listed from the highest to lowest |log2FC|

Cell type	Microglia (Mg)						Peripheral monocyte-derived cells (Mo)						Border associated macrophages (BAMs)					
comparison	PDOX vs GL261		PDOX vs Nu-NB		GL261 vs B6-NB		PDOX vs GL261		PDOX vs Nu-NB		GL261 vs B6-NB		PDOX vs GL261		PDOX vs Nu-NB		GL261 vs B6-NB	
Fold change	upregulated	downregulated	upregulated	downregulated	upregulated	downregulated	upregulated	downregulated	upregulated	downregulated	upregulated	downregulated	upregulated	downregulated	upregulated	downregulated	upregulated	downregulated
Number of genes	31	39	171	68	108	36	47	68	81	45	0	96	7	53	46	67	0	0
	Cxcl13	H2.Eb1	Spp1	Sfrs18	Cd74	Ttr	Cxcl13	H2.Eb1	Spp1	Gm26924		Ttr	Ifi202b	H2.Eb1	Selenop	Sepp1		
	Ttr	Plp1	Gm42418	Snord49b	H2.Ab1	Sparcl1	Ifi202b	Thbs1	Gm6977	Sepp1		Cd209a	Gm11942	Cd14	Gm14303	Rps29		
	Lpl	Rpl34	Cxcl13	Gm26924	H2.Eb1	P2ry12	Sparc	Arg1	Clec7a	Rpl5		Ighm	Mef2c	Rsad2	Gm6977	Sfrs18		
	Spp1	Hspa8	Clec7a	4632428N05Rik	H2.Aa	Gpr34	Ftl1	Hspa8	Cxcl13	Sfrs18		Cd244	Jun	Cdkn1a	Clec7a	Gm26924		
	Ifi202b	Rps2	Cd74	Sepp1	Il1b	Hpgd	Lpl	Rpl34	Gm5905	Rps27a		Nemp2	Cst3	Arg1	Gm5905	D4Wsu53e		
	Cwc22	H2.Q7	H2.Aa	Epb4.1l2	Lyz2	Selplg	Hexb	Cxcl2	Gm42418	Jhdm1d		Tnrc6c	mt.Cytb	Cxcl10	Gm42418	AI607873		
	Fn1	Fosb	Gm6977	Gm26339	Ifi27l2a	Rgs2	Gm11942	Rps2	Gm9794	Gm17087		H2.K2	Malat1	Rpl34	Rpl14.ps1	RP24.288C12.6		
	Ank	Rps18	Gm14303	Entpd1	Ccl12	Tmem119	Cst7	Rps18	Gm14303	Rps23		Gm19815		Ubl3	H2.Aa	Rps12		
	Ccl6	Bst2	Gm5905	Abca9	Clec7a	Tgfbr1	Cwc22	Vegfa	Rpl37rt	AI607873		Myrf		Ly6a	Gm9794	Vimp		
	Gm11942	Cebpb	Ccl4	D4Wsu53e	H2.D1	Mertk	Ctsd	Bst2	Rpl14.ps1	Rpl35		Phtf2		Tgfbi	Gm15427	Cmah		
	Ch25h	Gbp2	Gm9794	Gpr34	Ifitm3	Rnase4	Cx3cr1	Fosl2	Gm15427	Rps29		Pou2f2		Rps2	Gm11478	Gm26339		
	Ftl1	Nr4a1	H2.Ab1	P2ry13	H2.K1	Maf	Siglech	Cebpb	Ctsd	Lrrc58		Zfp329		Rps18	Cd63.ps	Lilrb4		
	Apoe	Gbp7	Ttr	P2ry12	Apoe	Golm1	Ttr	Cd14	Cd63.ps	Rpl19		Ccp110		Ly6c2	Gm4149	Cd163		
	Itgax	Fos	Lpl	Maf	Xist	Csf1r	Ifi44	Ptgs2	Gm11478	Rps12		Pla2g12a		Etf1	Rpl37rt	Rps27a		
	Sparcl1	Atf3	Rpl14.ps1	Jund	Vim	Cx3cr1	Serpine2	Fosb	Rps16.ps2	Rpl3		Spc25		Rpl30	Gm11942	Rpl10		
	Gm10260	Rpl35a	Lyz2	Zfhx3	Gbp2	Dusp6	Cst3	Il1b	Gm4149	Rps6		Tlr7		Iigp1	Gm5963	Rps23		
	Arsb	Ldhb	Rpl37rt	Nrip1	Cd83	Ifngr1	Gm10260	Nr4a2	Rack1	Trim30a		Wiz		Rpl35a	Rps24.ps3	Snord49b		
	Ptn	Ly6a	Fth1	Slco2b1	Ccl4	Scoc	Cd81	H2.Q7	Rps24.ps3	Diap2		Megf9		Il1b	Rplp1	F13a1		
	mt.Co1	Il1a	Gm15427	Tmem119	Nr4a1	P2ry13	mt.Cytb	S100a11	Gm9385	Ppp1r12a		Siglech		Bst2	Gm9385	Crebrf		
	mt.Cytb	Ccl12	Ccl3	Hpgds	H2.Q7	Fcrls	Trem2	AA467197	Gm5963	Arhgap30		Ctnnal1		Hspa8	Tpt1.ps3	Ccnd2		
	Cd34	Cd83	Gm11478	Il6ra	Ly6a	Siglech	Mef2c	Clec4n	Ccl4	Fau		Hspa1b		Nrp2	Wdr89	Med13		
	Axl	Fcrls	Cd63.ps	Cx3cr1	Cd52	Jun	Rnase4	Nr4a3	Trem2	Erbb2ip		Ate1		Ifi209	Rps16.ps2	Myl6		
	Cadm1	H2.Ab1	Rack1	Mertk	Il1a	Rhob	mt.Co1	Rpl29	Tpt1.ps3	Rps27		Stap1		Ccl2	Gm5805	Fau		
	Scd2	Ccl4	Gm16580	Smap2	Ifi204	Cst3	Golm1	Nlrp3	Gm11942	Rpl17		Myo18a		Cebpb	Pnisr	Zc3h7a		
	Xist	Dusp1	Gm11942	Ppp1r9a	Fth1	Ivns1abp	Ccl3	Ifrd1	Lilrb4a	Rpl21		Zfp207		Lgals3	Rpl3.ps1	Stard8		
	Ctsd	C1qb	Gm5963	Arid4a	Bst2	Serpine2	Creg1	Tgfbi	Gm5805	Rab8b		Mef2c		Prdx5	Gm10073	Ptpn6		
	mt.Nd4	Ifrd1	Cst7	Son	Ifrd1	Il6ra	Spp1	Ly6a	Rpl3.ps1	Rpl27		Dmxl1		Aldoa	Gm10288	Ralgps2		
	mt.Nd2	Cd14	Apoe	Tanc2	Rpl32	Elmo1	mt.Nd1	Ccr5	Gm16580	Gnb2l1		Mum1		Lyz2	Rps13.ps2	Abi1		
	Gnas	Ifi204	Selenop	Chd9	Txn1	Pip4k2a	mt.Nd4	Ccr1	Rps13.ps2	Rpl13		Cuedc1		Ckap5	Lilrb4a	Ppia		
	mt.Nd5	Nfkbia	Gm4149	Srgap2	Rpl39	Sirpa	Cadm1	Dab2	Gm6204	Rpl18a		Ankrd49		Pim1	Rsrp1	Kmt2c		
	mt.Nd1	C1qc	Rps24.ps3	Ogfrl1	Stat1	Plxdc2	Jun	Gpnmb	Fth1	Tpt1		Plxdc2		Mndal	Cd52	Tab2		
		Il1b	Gm9385	Scamp2	Nfkbia	Fam105a	Rpl13a	Osm	Rpl36a.ps2	Mrps25		Clock		Fosb	Rack1	Iqgap2		
		Mcl1	Rps16.ps2	Elmo1	Tmsb10	Rbm5	Cd9	Gm9493	Gm4332	Rpl13a		Faap100		Vim	Rpl18.ps1	Cwc22		
		Txnip	Gm5805	Mycbp2	Rps20	Vsir	mt.Nd2	Mndal	Cst7	Sarnp		Slco2b1		Psap	Mt1	Nrp1		
		H2.DMa	Rpl3.ps1	Lpar6	Plp1	Hpgds	Egr1	Rpl3	Gm8995	Rpl10		Tcf3		Srgn	Rpl36a.ps2	Map4k4		
		Ly86	Pnisr	Rbm5	Cstb	Hexb	Ctsl	Crem	Rps10.ps1	Ptp4a2		Znrf2		Ccl7	Ttr	Abca9		
		Ubc	Ccl6	Ssh2	Nfkbiz		Hpgds	Lyz2	Rps26	Rpl7a		Acss2		Txn1	Gm6204	Sbds		
		Rpl3	Fn1	Marcks	Rps2		Gnas	Tnfaip2	Gm10073	Ifi203		Dhx33		Gm16580	Slc25a5	Gm17087		
		Socs3	Rps13.ps2	Qk	Lgals3bp		Abhd12	Nr4a1	Gm14586	Rpl15		Tmem183a		Samd9l	Rps5	Rps6		
			Il1b	Macf1	Slfn2		Mtdh	Cdkn1a	Gm4617	Prrc2c		Sp3		Ccr1	Fth1	Fam111a		
			Gm6204	Arl4c	Ccl3		Apbb1ip	Plaur	Rgs1	Rpl23a		Eif3s6.ps2		Nfe2l2	Gm9843	Emp3		
			Ifi27l2a	Ythdc1	Rps26		Man2b1	Rpl30	Lpl	Ptma		Trp53inp1		Rnasel	Npc2	Aldh2		
			Gm8995	Cwc22	Atf3		Ccl4	Slamf7	Rpl10a.ps1	D4Wsu53e		Plxna4os1		Cox5a	H2.D1	Mcfd2		
			Cd52	Frmd4a	Anp32b		Cd63	Trim25	Rpl18.ps1	Lyz1		Cc2d1b		Psmb9	B2m	Cfh		
			Kctd12	Dusp6	B2m		Rps4x	Tnfaip3	Gm15500	Clec12a		Trerf1		Fth1	Fcer1g	Ctage5		
			H2.K1	Olfml3	Mif		Apoe	Fgl2	Cd63			Ttf1		H2.Ab1	Actb	Hmgb1		
			Rpl36a.ps2	Prrc2c	Rpl9.ps6		Rgs1	Tiparp	mt.Nd1			Pik3cd		Pomp		Sgpl1		
			Epb41l2	Srrm2	Rpl37			Pim1	Gm6030			Gm6564		C1qb		Rabep1		
			Ch25h	Ifngr1	Ncl			Mxd1	Rps25.ps1			1110051M20Rik		Sh3bgrl3		Dusp6		
			Rpl10a.ps1	Ivns1abp	Rpl41			Pde4b	Rpsa.ps10			Igip		H2.K1		Cx3cr1		
			Rps5	Mtdh	Rplp1			Il1rn	mt.Cytb			Rfx5		Calm1		Ppp1r9a		
			Rsrp1	Acin1	Rack1			Ets2	Rps5			Hpgd		Stat1		Tlr7		
			Gm14586	Arhgap5	Gm10288			Nfil3	Gm5835			Gm15207		B2m		Hnrnpl		
			Gm4617	Ccr5	Rpl22			Cytip	Ctsl			Slc12a2				Plekho1		
			Ctsd	Selplg	Rpl14			Tgm2	Rsrp1			Zfp770				Mrc1		
			Gm4332	Malat1	Tspo			Acp5	Rpl31.ps8			Fam149a				Gpr183		
			Ifi202b	Pnn	Srgn			H2.Ab1	Rps15a.ps5			Nfix				Maf		
			Rpsa.ps10	Pde3b	Cd14			Cyp4f18	Cd81			Nupl1				Rpl13a		
			Rps26	Tgfbr1	Rpl31.ps8			Dusp1	Sparc			Slc1a3				Acin1		
			Mt1	Rbm25	Psmb8			Clic4	Gm6863			Poc1b				Mndal		
			H2.D1	Pmepa1	Rps10.ps1			Rps24.ps2	Rplp0			Il16				Zfp36l2		
			Rps25.ps1	Anp32a	Rps8			Tmsb10	H2.K1			Enpp4				Rin2		
			Gm10288	Basp1	Rps3			Klf4	Gm10275			Syne2				Fus		
			Calr.ps	Serinc3	Rps10			Rpl35a	Gnas			Strip1				Hspa8		
			Gm6030	Srrm1	Rps25.ps1			Bcl2a1b	Rps15			Ipo9				Pdia3		
			Cybb	Ywhah	Gm9794			Vim	Apoe			Vps52				D8Ertd738e		
			Wdr89	Jmjd1c	Mcl1			Emb	mt.Nd4			Slc44a2				C1qa		
			Ctsb	Ddx5	Rps19			Lgals3	Rplp1			Dleu2						
			Rpl31.ps8		Wdr89				Ctsb			Rbm25						
			AU020206		Ccrl2				Slc25a5			Gm10064						
			Tyrobp		Cebpb				Gm9843			D17H6S53E						
			Ccrl2		Rps24				Atox1			Rabggta						
			Gm15500		Gm4332				B2m			Cfh						
			Gm9843		Hint1				Npc2			Map4k5						
			Gm5835		Gm14303				Rps3			Rrad						
			Tpt1.ps3		Gm5963				Rpl32			Dopey1						
			Gm10073		Rplp0				Tyrobp			Spata13						
			Rpl18.ps1		Rpl8				Mpeg1			Rpl5						
			Gm8276		Rpl37rt				Wdr89			Tesk1						
			Rpl32		Rps15				Aif1			Fam120b						
			Vsir		Rps26.ps1				Ctsz			Usp32						
			Axl		Prdx1							Prnp						
			Rps20		Rps15a							Bbc3						
			Rplp1		Gbp7							Zfp120						
			Itgax		Rps24.ps3							Gm37494						
			Id2		Tubb5							Ube3b						
			Rps10.ps1		Egr1							Gpm6b						
			mt.Cytb		Fosb							Dhx57						
			mt.Nd1		Gm16580							Wdr60						
			Gm14681		Rps21							Tppp3						
			Gm6863		Gm6977							RP23.394G23.2						
			Ms4a6c		Ifi30							Gm7589						
			Cd83		Rpl23							Sort1						
			Gnas		Bcl2a1b							Hspa1a						
			Gm10275		Eef2							E330020D12Rik						
			Ank		Gm6030							Rufy2						
			Cd63		Rps5													
			Atox1		Npm1													
			Ctsz		Pkm													
			Atf3		Pabpc1													
			B2m		Rpl34													
			Nfkbia		Plek													
			Atp5e		Rpl36a.ps2													
			Srgn		Rps13.ps2													
			Calm1		H2.T23													
			Cox6a1		Gatm													
			Rps26.ps1		Rpl4													
			Cstb		Eef1b2													
			Npc2															
			Dbi															
			Rpl39															
			Cox4i1															
			Ctsl															
			Rgs1															
			Grn															
			Prdx1															
			Rps15															
			Csf2ra															
			Cox6b1															
			mt.Nd4															
			Gusb															
			Lgals3bp															
			Pabpc1															
			Pld3															
			Ppp1r15a															
			Rplp0															
			Tmsb10															
			Rps19															
			Gpx1															
			Cox8a															
			Fcer1g															
			Rps9															
			Cd9															
			Slc25a5															
			Nfkbiz															
			Gm8730															
			Rps3															
			Rps11															
			Akr1a1															
			Cotl1															
			Sh3bgrl3															
			Uqcrq															
			Ier2															
			Gm6472															
			Ndufa2															
			Ssr4															
			Shfm1															
			Ctsh															
			Psme2b															
			Eif3f															
			Rpl22l1															
			Myo5a															
			Rps21															
			Rps14															
			Rpl8															
			2010107E04Rik															
			Gm10076															
			Arpc1b															
			Atp6v1f															
			Cox6c															
			Eef2															
			Pfdn5															
			Cd68															
			Myeov2															
			Mpeg1															
			Itgb2															
			Cox7a2															
			Ndufa1															
			Tmem256															
			Rpl41															
			Atp5j2															

Table S7. List of gene signatures applied for scRNA-seq analysis.

Signature	Human Mg	Human Mo	Enzymatic activation	Homeostatic Mg	Sensome	Sensome w/o homeostatic Mg	Migration	APC	Phagocytosis
Genes	12	13	25	35	140	127	464	106	219
	HEXB	CCR2	Rgs1	Bhlhe41	4632428N05Rik	Abcc3	Abl1	Abcb9	4932438A13Rik
	P2RY12	PLAC8	Hist2h2aa1	Crybb1	Abcc3	Adora3	Acvrl1	Ap3b1	4933434E20Rik
	P2RY13	CLEC12A	Hist1h4i	Csf1r	Adora3	Adrb2	Adam17	Ap3d1	Abca1
	TMEM119	FCN1	Nfkbiz	Ctsd	Adrb2	Bin2	Adam8	Arl8b	Abca7
	GPR34	VCAN	Klf2	Ctsf	AF251705	C3ar1	Adam9	Atg5	Abl1
	CXCR1	LYZ	Junb	Cttnbp2	Bin2	C5ar1	Adamts9	B2m	Abl2
	TGFBR1	LGALS3	Dusp1	Cttnbp2nl	C3ar1	Ccr5	Adgra2	Bag6	Abr
	CST3	CRIP1	Ccl3	Cx3cr1	C5ar1	Ccrl2	Adgrb1	Calr	Adgrb1
	OLFML3	S100A4	Hspa1a	Fcrls	C5ar2	Cd14	Adgrg1	Ccr7	Aif1
	SALL1	S100A6	Hsp90aa1	Golm1	Ccr5	Cd180	Adipor1	Cd1d1	Ano6
	SELPLG	S100A8	Fos	Gpr34	Ccrl2	Cd22	Adora2b	Cd68	Anxa1
	TREM2	S100A9	Hspa1b	Gpr84	Cd101	Cd300a	Agt	Cd74	Anxa3
		CD44	Jun	Hexb	Cd14	Cd33	Aif1	Clec4a1	Appl1
			Jund	Hpgds	Cd180	Cd37	Akirin1	Clec4a2	Appl2
			Nfkbid	Hspa1a	Cd22	Cd48	Akt1	Clec4a3	Arhgap12
			Gem	Lgmn	Cd300a	Cd52	Akt3	Ctsl	Arhgap25
			Ccl4	Lrrc3	Cd33	Cd53	Amot	Ctss	Arl8b
			Ier5	Ltc4s	Cd37	Cd68	Amotl1	Erap1	Atg3
			Txnip	Mafb	Cd48	Cd74	Angpt1	Ext1	Atg5
			Hist1h2bc	Nuak1	Cd52	Cd83	Angpt2	Fcer1g	Atg7
			Zfp36	Olfml3	Cd53	Cd84	Anln	Fcgr1	Axl
			Hist1h1c	P2ry12	Cd68	Cd86	Ano6	Fcgr2b	Bcr
			Egr1	P2ry13	Cd74	Clec4a2	Anxa1	Fcgr3	Becn1
			Atf3	Plxdc2	Cd79b	Clec4a3	Anxa3	Fcgr4	Bin2
			Rhob	Rab3il1	Cd83	Clec5a	Apoe	Fgl2	C2
				Sall1	Cd84	Clec7a	App	Gba	C3
				Scoc	Cd86	Cmklr1	Aqp1	Gm8909	C4b
				Serpine2	Clec4a2	Cmtm6	Arf6	H2.Aa	Calr
				Siglech	Clec4a3	Cmtm7	Arl13b	H2.Ab1	Camk1d
				Slc2a5	Clec4b1	Csf2rb2	Arsb	H2.D1	Ccl2
				Tgfbr1	Clec5a	Csf3r	Atoh8	H2.DMa	Ccr2
				Tmem119	Clec7a	Cxcl16	Atp2b4	H2.DMb1	Ccr7
				Atp8a2	Cmklr1	Cysltr1	Atp5a1	H2.DMb2	Cd300a
				Lag3	Cmtm6	Daglb	Atp5b	H2.Eb1	Cd300lf
				Slco4a1	Cmtm7	Ecscr	Axl	H2.K1	Cd302
					Csf1r	Entpd1	Bcar1	H2.M3	Cd36
					Csf2rb2	Fcer1g	Bcas3	H2.Oa	Cd47
					Csf3r	Fcgr1	Bmpr2	H2.Q10	Cdc42
					Cx3cr1	Fcgr2b	Bsg	H2.Q4	Cdc42se1
					Cxcl16	Fcgr3	Bst1	H2.Q6	Cdc42se2
					Cysltr1	Fcgr4	C1qbp	H2.Q7	Cfp
					Daglb	Gpr160	C3ar1	H2.T22	Clcn3
					Ecscr	Gpr183	C5ar1	H2.T23	Clec7a
					Emr1	Gsdmd	Calr	H2.T24	Cln3
					Entpd1	H2.Oa	Camk1d	H2.T3	Cnn2
					Fcer1g	H2.Ob	Capn7	Hfe	Colec12
					Fcgr1	H2.T23	Card10	Icam1	Coro1a
					Fcgr2b	Havcr2	Ccdc141	Ide	Coro1c
					Fcgr3	Hfe	Ccl12	Ifi30	Csk
					Fcgr4	I830077J02Rik	Ccl17	Ighm	Cyba
					Fcrl1	Icam1	Ccl2	Mfsd6	Dnm2
					Fcrls	Icosl	Ccl24	Mr1	Dock1
					Gpr160	Ifitm6	Ccl25	Nod1	Dock2
					Gpr183	Ifngr1	Ccl3	Nod2	Eif2ak1
					Gpr34	Igsf6	Ccl4	Pdia3	Elmo1
					Gpr56	Il10ra	Ccl5	Pikfyve	Elmo2
					Gpr84	Il21r	Ccl6	Psap	Elmo3
					Gsdmd	Il4ra	Ccl7	Psmb8	Fcer1g
					H2-Oa	Il6ra	Ccl8	Psmb9	Fcgr1
					H2-Ob	Itgam	Ccl9	Psme1	Fcgr2b
					H2-T23	Itgb2	Ccr1	Psme2	Fcgr3
					Havcr2	Itgb5	Ccr2	Ptpn22	Fgr
					Hfe	Kctd12	Ccr7	Pycard	Gas6
					I830077J02Rik	Lair1	Cd200	Rab10	Gata2
					Icam1	Laptm5	Cd200r1	Rab27a	Gsn
					Icam4	Lat2	Cd40	Rab32	Gulp1
					Icosl	Lgals9	Cd63	Rab33a	Hck
					Ifitm6	Lilra5	Cd74	Rab34	Hmgb1
					Ifngr1	Ly86	Cd81	Rab35	Hspa8
					Igsf6	Nckap1l	Cd9	Rab3b	Ighm
					Il10ra	Nfam1	Cdh13	Rab3c	Il15
					Il21r	Ninj1	Cdh5	Rab4a	Il15ra
					Il4ra	Numb	Cdk5	Rab5b	Il1b
					Il6ra	P2ry6	Cdk5r1	Rab6a	Il2rg
					Itgam	Pilra	Ceacam1	Rab8b	Irf8
					Itgb2	Plekho1	Cib1	Relb	Itga2
					Itgb5	Pmepa1	Cklf	Rftn1	Itgal
					Kctd12	Pmp22	Clasp1	Slc11a1	Itgam
					Lag3	Ptafr	Clasp2	Tap1	Itgav
					Lair1	Ptprc	Clec14a	Tap2	Itgb1
					Laptm5	Selplg	Cmklr1	Tapbp	Itgb2
					Lat2	Sirpa	Cntn2	Tapbpl	Itgb3
					Lgals9	Slamf9	Col3a1	Thbs1	Jmjd6
					Lilra5	Slc11a1	Coro1b	Traf6	Lbp
					Liph	Slc16a3	Coro1c	Trem2	Ldlr
					Lpar5	Slc44a2	Creb3	Unc93b1	Lepr
					Ltf	Slc46a1	Csf1	Was	Letmd1
					Ly86	Slc7a7	Csf3r	Wdfy4	Lman2
					Nckap1l	Slc7a8	Csnk2b	Ythdf1	Lrp1
					Nfam1	Slco2b1	Ctnnb1	H2.M5	Lyar
					Ninj1	Stab1	Ctsh	Kdm5d	Lyst
					Numb	Tcirg1	Cul5	Flt3	Masp2
					P2ry12	Tgfbr2	Cx3cl1	Gm11127	Megf10
					P2ry13	Tlr13	Cxadr	H2.Ob	Mertk
					P2ry6	Tlr2	Cxcl1	H2.Eb2	Met
					Pilra	Tlr4	Cxcl10	H2.M2	Mex3b
					Plekho1	Tlr6	Cxcl12	H2.Q1	Mfge8
					Plxdc2	Tlr7	Cxcl13	H2.Q2	Msr1
					Pmepa1	Tmem173	Cxcl2	Treml4	Myd88
					Pmp22	Tmem206	Cxcl9	Ifng	Myh9
					Ptafr	Tmem37	Cxcr4	Clec4b1	Myo18a
					Ptprc	Tnfrsf13b	Cyp1b1	Ctse	Myo1g
					Selplg	Tnfrsf1a	Daam2	H2.M9	Myo7a
					Siglec5	Tnfrsf1b	Dab1	Azgp1	Ncf2
					Siglece	Trem2	Dab2ip	Gm7030	Ncf4
					Siglech	Trpv2	Dcn	Ccl21a	Nckap1l
					Sirpa	Tyrobp	Dicer1		Nod2
					Slamf9	Icam4	Disc1		Nr1h3
					Slc11a1	Cd79b	Dixdc1		P2rx7
					Slc16a3	Siglece	Dnaja4		P2ry6
					Slc2a5	Tlr1	Dnm1l		Pear1
					Slc44a2	C5ar2	Dock1		Pecam1
					Slc46a1	Cd101	Dock5		Pik3ca
					Slc7a7	Liph	Dpp4		Pikfyve
					Slc7a8	Tlr12	Dusp1		Pip5k1c
					Slco2b1	Tnfrsf17	Dusp10		Pla2g5
					Stab1	Upk1b	Edn1		Plcg2
					Tcirg1	Fcrl1	Edn3		Pld4
					Tgfbr1	Tmem8c	Ednra		Pot1b
					Tgfbr2	Clec4b1	Ednrb		Prkcg
					Tlr1	Lpar5	Efhc1		Pros1
					Tlr12	4632428N05Rik	Efna1		Pten
					Tlr13	AF251705	Efnb2		Ptk2
					Tlr2	Emr1	Egf		Ptprc
					Tlr4	Gpr56	Egfr		Ptprj
					Tlr6	Siglec5	Emc10		Ptx3
					Tlr7	Ltf	Emilin1		Pycard
					Tmem119		Emp2		Rab11fip2
					Tmem173		Emx2		Rab14
					Tmem206		Enpp1		Rab20
					Tmem37		Enpp2		Rab27a
					Tmem8c		Epb41l4b		Rab31
					Tnfrsf13b		Epb41l5		Rab34
					Tnfrsf17		Epha2		Rab5a
					Tnfrsf1a		Ephb4		Rab7
					Tnfrsf1b		Ets1		Rab7b
					Trem2		Evl		Rac1
					Trpv2		Fap		Rac2
					Tyrobp		Fbxo45		Rack1
					Upk1b		Fbxw7		Rap1a
							Fcer1g		Rap1gap
							Fcgr3		Rapgef1
							Fgf1		Rara
							Fgf2		Rhobtb1

							Fgf7		Rhobtb2
							Fgfr1		Rhog
							Flt1		Rhoh
							Flt4		Scarb1
							Foxc2		Sh3bp1
							Foxg1		Sirpa
							Foxp1		Slc11a1
							Fstl1		Snx3
							Fut10		Sod1
							Gab2		Spg11
							Gadd45a		Sphk1
							Gata2		Stap1
							Gbf1		Syk
							Gipc1		Syt11
							Gli3		Syt7
							Glipr2		Tgfb1
							Glul		Tgm2
							Gpi1		Thbs1
							Gpld1		Tlr2
							Gpr35		Tlr4
							Gpx1		Tm9sf4
							Grn		Tmem175
							Has2		Tnf
							Hbegf		Trbc2
							Hc		Trem2
							Hdac5		Tub
							Hdac6		Tusc2
							Hdac7		Tyro3
							Hdac9		Tyrobp
							Hif1a		Unc13d
							Hmgb1		Vamp7
							Hmox1		Vav1
							Hspb1		Xkr5
							Hyal1		Xkr6
							Igf1		Xkr8
							Igf2		Fer1l5
							Il1b		Icam5
							Il34		Trbc1
							Iqsec1		Apoa2
							Itga1		Il2rb
							Itga2		Mbl2
							Itga3		Cfb
							Itga9		Siglece
							Itgam		Sirpb1b
							Itgav		Sirpb1c
							Itgb1		Ticam2
							Itgb1bp1		Xkr4
							Itgb2		Gm9733
							Itgb3		Dysf
							Jam3		Gm5150
							Jun		Ighd
							Jup		Igkc
							Kank1		Iglc3
							Kank2		Rab39
							Kdr		Sirpb1a
							Kit		Slamf1
							Klf4		Treml4
							Krit1		F2rl1
							Lamb1		Ifng
							Lbp		Marco
							Lemd3		Prtn3
							Lgals3		Ager
							Lgals8		Apoa1
							Loxl2		Cd209b
							Lpxn		Iglc1
							Lrp6		Iglc2
							Lrp8		Masp1
							Lrrk2		Spon2
							Lyn		Srpx
							Macf1		Tulp1
							Map2k3		Alox15
							Map2k5		Havcr1
							Map3k3		Timd4
							Map4k4		Trdc
							Mapk1		Colec11
							Mapk3		
							Mapre2		
							Mboat7		
							Mcc		
							Mcu		
							Mdk		
							Mecp2		
							Mef2c		
							Met		
							Mia3		
							Mif		
							Mmp14		
							Mmp28		
							Mmrn2		
							Mospd2		
							Mpp1		
							Mtor		
							Mtus1		
							Myh9		
							Myo9b		
							Nanos1		
							Nbl1		
							Nckap1l		
							Ndel1		
							Nf1		
							Nfe2l2		
							Ninj1		
							Nod2		
							Nos3		
							Notch1		
							Nr2e1		
							Nr2f2		
							Nr4a1		
							Nrp1		
							Nup85		
							Nus1		
							P2rx4		
							Pafah1b1		
							Patz1		
							Paxip1		
							Pdcd10		
							Pdcd6		
							Pde4b		
							Pde4d		
							Pdgfb		
							Pdpk1		
							Pecam1		
							Pex13		
							Pex5		
							Pf4		
							Pfn1		
							Pfn2		
							Pik3c2a		
							Pik3cb		
							Pik3cd		
							Pik3cg		
							Pik3r2		
							Pikfyve		
							Pkn1		
							Pkn2		
							Pkn3		
							Pla2g7		
							Plcg1		
							Plcg2		
							Plekhg5		
							Plk2		
							Plpp3		
							Plxnd1		
							Pou3f2		
							Pou3f3		
							Ppard		
							Ppia		
							Ppib		
							Ppm1f		
							Prcp		

							Prex1		
							Prkca		
							Prkce		
							Prkd1		
							Prkd2		
							Prox1		
							Psen1		
							Pten		
							Ptgs2		
							Ptk2		
							Ptk2b		
							Ptp4a3		
							Ptpn11		
							Ptpn23		
							Ptprg		
							Ptprj		
							Ptprm		
							Ptpro		
							Pxn		
							Rab11a		
							Rab13		
							Rac1		
							Rac2		
							Rarres2		
							Reln		
							Rgcc		
							Rhoa		
							Rhob		
							Rhoj		
							Rin2		
							Rnf7		
							Robo1		
							Rock1		
							Rock2		
							Rpl13a		
							Rps19		
							Rras		
							Rreb1		
							Rtn4		
							Sash1		
							Scarb1		
							Sell		
							Sema3a		
							Sema4a		
							Sema5a		
							Serpine1		
							Serpinf1		
							Sh3bp1		
							Sirt1		
							Slc37a4		
							Slit2		
							Smoc2		
							Snai2		
							Socs7		
							Sox9		
							Sp1		
							Sp100		
							Sparc		
							Spp1		
							Spred1		
							Src		
							Srf		
							Srgap2		
							Srpx2		
							Stap1		
							Stard13		
							Stat5a		
							Sun1		
							Sun2		
							Svbp		
							Syk		
							Syne2		
							Synj2bp		
							Tbxa2r		
							Tek		
							Tesk1		
							Tgfb1		
							Tgfb2		
							Tgfbr2		
							Tgfbr3		
							Thbs1		
							Tirap		
							Tnf		
							Tnfaip6		
							Tnr		
							Trem1		
							Trem2		
							Trpv4		
							Tyro3		
							Vash1		
							Vav1		
							Vav3		
							Vegfa		
							Vhl		
							Vil1		
							Wdpcp		
							Wnt5a		
							Zc3h12a		
							Zeb2		
							Zfp580		
							Zmiz1		
							Il23a		
							Mst1		
							Ccl22		
							Xcl1		
							Apoh		
							Cxcl3		
							Gata3		
							Gdf2		
							S100a14		
							Arx		
							Bmper		
							Ccl11		
							Dcx		
							Dll4		
							Fezf2		
							Fgf13		
							Lcn2		
							Ptprr		
							Stc1		
							Thbs4		
							Wnt7a		
							Cdk5r2		
							Bmp4		
							C5ar2		
							Ccr6		
							Dysf		
							Nrg1		
							S100a8		
							Slamf1		
							Slamf8		
							Trem3		
							Vegfc		
							Vstm4		
							Mcoln2		
							Dapk2		
							Ccl1		
							Ifng		
							Tac1		
							Tnfsf11		
							Ager		
							Alox12		
							Apoa1		
							Cxcl5		
							Defb25		
							Fgf18		
							Fgfbp1		
							Foxb1		
							Lgals12		
							Mdga1		
							Slit1		
							Adtrp		
							Ccbe1		
							Drd1		
							Fgf10		
							Lhx6		
							Lrg1		
							Meox2		
							Mmp9		
							Msmp		
							Mstn		
							Ror2		
							S100a9		
							Tacr1		
							Cxcr2		
							Egr3		
							Trim55		
							Nrg3		
							Fermt1		
							Ccl21a		

Table S8. Lists of differentially expressed genes between TMZ treated and control P3 PDOXs per each major cell type analysed.
Differentially expressed genes were defined at threshold: FDR <=0.01 and |log2FC|>=0.5
Genes are listed from the highest to lowest |log_2FC|

Cell origin	mouse TME						human GBM	
Cell type	Astrocytes		Endothelial cells		Myeloid cells		Tumor cells	
Fold change	upregulated	downregulated	upregulated	downregulated	upregulated	downregulated	upregulated	downregulated
Number of genes	2	1	41	47	127	28	90	78
	Scg2	Gm12222	RP23.269H21.1	Ttr	Rnaset2b	Rpl26	GDF15	BCAN
	Aldoc		Rnaset2b	Rpl26	Gm14513	Rpl35a	CDKN1A	ID3
			mt.Tc	Rpl35a	Cxcl13	Igfbp7	SCG2	C1orf61
			Gm7638	Hexb	Gm7638	Ttr	CHI3L1	HES5
			Abcb1a	Apln	Pde3b	Ptn	NEAT1	CSPG5
			Cxcl12	Rpl39.ps	Ptgs1	Gng5	GADD45A	FABP7
			Pcp4l1	Ctsd	Jund	Rps15a.ps5	RPS27L	HMGCS1
			Paqr5	Cwc22	Slco2b1	Gm10076	FTL	CST3
			Timp3	Lrp8	Spp1	Rpl22.ps1	MDM2	HSPA1A
			Slc6a6	Myh10	Zfhx3	Enpp2	DDB2	HSPA1B
			H2.D1	Ctss	Rpsa	Gm12338	GAP43	NCAN
			Hsp25.ps1	Tyrobp	Apoe	Rps15a.ps7	OCIAD2	MSMO1
			Degs2	Ndufs5	P2ry12	Gm5644	NRP2	TTYH1
			Fam32a	Rpl36a.ps2	Tmem119	Gas5	S100A6	ACAT2
			Ly6a	Dok4	Cfl1	Gm10269	BTG1	HEY1
			Ppil4	Hopx	Cnpy2	Cd52	PHLDA1	FDPS
			Ucp2	Hspa5	Cx3cr1	Rpl36a.ps2	SPP1	DBI
			Hspb1	Slc7a1	Rpl9.ps6	Ndufa4	SQSTM1	IDH1
			Ly6c1	Lyz2	Cited2	Rpl37rt	TIMP1	METRN
			Serinc3	Gm7266	Tubgcp5	Gm4332	SLC3A2	LMO4
			Sparcl1	Wwtr1	Mpc1	Rplp2	ZMAT3	PEA15
			Lrg1	Ccny	Fscn1	Rpl23	C6orf141	MT3
			Maoa	Rpl38.ps2	Ltc4s	Gm4149	TUBA1C	EDNRB
			Aldh2	Rps15a.ps5	Gm10263	Rps16.ps2	IGFBP3	SCRG1
			mt.Rnr1	Slc38a2	Mylip	Rps21	PHPT1	NDRG2
			Tspo	Rps11.ps3	Ftl1	Ndufs5	PHLDA3	CYP51A1
			U2af1	Gas5	Aimp1	Rpl32	IDS	CNN3
			Pltp	Gm10076	Dtnbp1	Rps15	GAS5	HIST1H4C
			mt.Rnr2	Gm15148	C1qa		VGF	RPS10
			Cd59a	RP23.288C18.3	Ucp2		DDIT3	YWHAE
			Gm694	Cst3	Pwwp2a		ARL4C	FDFT1
			Mal	Pmepa1	Rhob		MALAT1	FAM181B
			Gm14513	Gm10269	Coro1a		FDXR	ARC
			Ly6e	Rflnb	Pik3cg		RPS19	ATP1A2
			Bsg	Rpl37rt	Zfp90		BAX	IDI1
			Malat1	Nostrin	Rpl13		LMNA	MARCKS
			Ifitm2	Trf	Gpr34		GABPB1-AS1	ASCL1
			Utrn	Calr	Frmd4a		CAMK2D	FOS
			Ubb	Rps11.ps2	Ifngr1		RCAN1	GNAI2
			Gm11560	Gm4149	Scamp2		PMEPA1	SAMD1
			Cldn5	Sparc	Rpl18.ps1		IGFBP5	HES4
				Tmsb4x	Lrp1		LONRF2	SDC3
				Hsp90b1	Sox4		MDK	STMN1
				Gm9794	Selplg		ENC1	IGFBP2
				Tmsb10	Qpct		AEN	LRRC17
				Rpl32	Sltm		PGM2L1	TUBB2B
				Rplp1	Rpl10.ps3		TNFRSF12A	TMSB4X
					Gpsm3		CEBPB	TUBA1B
					Lamtor1		ZFAS1	ITM2C
					Ier3		SRPX	NRARP
					Tmem100		NUPR1	MGST3
					Sdhd		HBEGF	SQLE
					Commd2		YBX3	SLC1A3
					Eif4g2		ANXA2	CHMP4B
					Arl4c		PMAIP1	ID1
					Mknk1		VMP1	GFAP
					Dnm2		ASCC3	TMSB15A
					Kctd12		RBP1	PLPP3
					Scd2		PLK3	CAMTA1
					Fam76b		TRIAP1	GATM
					Ankle2		TMSB10	MDFI
					Scarb2		APLP1	HSPA8
					Ly6e		PLXNB2	HNRNPA2B1
					Gm15536		SLC25A37	QKI
					Rab10		AC010198.2	SLC6A11
					Gm10443		TMEM158	ARL6IP6
					Hpgds		KCNF1	TUBB2A
					Mxi1		SESN2	OLIG1
					Zfand6		AL353138.1	MT1M
					Prdx5		CMBL	APCDD1
					H2.T.ps		CCND1	TSPAN3
					Naa50		CIRBP	FGFBP3
					Tmem86a		FBXO22	AC004540.2
					Slc29a3		S100A16	MT2A
					Abca1		SMIM3	ID2
					Atp6v0a1		JAG1	MARCKSL1
					Ninj1		LGALS3	PAFAH1B3
					Limd2		ARHGEF2	SAPCD2
					Olfml3		PDLIM4	
					Elf1		XPC	
					Prune2		TIGAR	
					Arpc4		BLOC1S2	
					Zfp36l1		TXNIP	
					Timp2		CEBPG	
					Msr1		METTL7B	
					Bin1		PRKCQ-AS1	
					Parvg		QDPR	
					Marcks		SCG5	
					Gm8979		SH3BGRL3	
					Ywhae		PDGFC	
					Phyh			
					Rgs19			
					Sp3			
					Arl6ip4			
					Tuba1b			
					Ndufs7			
					Cd37			
					Comt			
					Brk1			
					Rpl10a			
					Sun2			
					Scoc			
					Rtn4rl1			
					Colgalt1			
					Crebrf			
					Pla2g7			
					H2.D1			
					Abcg1			
					Srgap2			
					Gla			
					Gm8399			
					Ube2d3			
					Lgmn			
					mt.Co1			
					Bmyc			
					Csf1r			
					Gm8606			
					Id2			
					Cndp2			
					Zfp62			
					Lyl1			
					Iah1			
					Dock10			
					Fam49b			
					Kdm7a			
					Psmc4			
					Selenop			
