# Supplementary material for: Glioblastoma-instructed microglia transit to heterogeneous phenotypic states with phagocytic and dendritic cell-like features in patient tumors and patient-derived orthotopic xenografts
Source: bioRxiv. 2023 Mar 6:2023.03.05.531162. Preprint. [Version 1] doi: 10.1101/2023.03.05.531162 (PMC10028830; doi:10.1101/2023.03.05.531162)
Supplement: Supplementary file 1 [file media-1.pdf]

# Glioblastoma-instructed microglia transit to heterogeneous phenotypic states with phagocytic and dendritic cell-like features in patient tumors and patient-derived orthotopic xenografts

Yahaya A. Yabo[1,2], Pilar M. Moreno-Sanchez[1,2], Yolanda Pires-Afonso[2,3], Tony Kaoma[4], Dimitrios Kyriakis[2,5], Kamil Grzyb[5], Suresh K. Poovathingal[5,6], Aurélie Poli[3], Andrea Scafidi[2,3], Arnaud Muller[4], Reka Toth[4], Anaïs Oudin[1], Barbara Klink[7,8,9,10], Guy Berchem[8,11], Christophe Berthold[11], Frank Hertel[5,11], Michel Mittelbronn[2,5,8,12,13,14], Dieter Henrik Heiland[15,16,17,] Alexander Skupin[5,18,19], Petr V. Nazarov[4], Simone P. Niclou[1], Alessandro Michelucci[1,3,5,#], Anna Golebiewska[1,#]

[1]NORLUX Neuro-Oncology Laboratory, Department of Cancer Research, Luxembourg Institute of Health (LIH), L-1526 Luxembourg, Luxembourg; [2]Faculty of Science, Technology and Medicine (FSTM), University of Luxembourg, L-4367 Belvaux, Luxembourg; [3]Neuro-Immunology Group, Department of Cancer Research, Luxembourg Institute of Health, L-1526 Luxembourg, Luxembourg; [4]Multiomics Data Science, Department of Cancer Research, Luxembourg Institute of Health, L-1445 Strassen, Luxembourg; [5]Luxembourg Centre for Systems Biomedicine (LCSB), University of Luxembourg, L-4362 Esch-sur-Alzette, Luxembourg; [6]Single Cell Analytics & Microfluidics Core, Vlaams Instituut voor Biotechnologie-KU Leuven, 3000 Leuven, Belgium; [7]National Center of Genetics, Laboratoire National de Santé, L-3555 Dudelange, Luxembourg; [8]Department of Cancer Research, Luxembourg Institute of Health, L-1526 Luxembourg, Luxembourg; [9]German Cancer Consortium (DKTK), 01307 Dresden, Germany; Core Unit for Molecular Tumor Diagnostics (CMTD), National Center for Tumor Diseases (NCT), 01307 Dresden, Germany; German Cancer Research Center (DKFZ), 69120 Heidelberg, Germany; [10]Institute for Clinical Genetics, Faculty of Medicine Carl Gustav Carus, Technische Universität Dresden, 01307 Dresden, Germany; [11]Centre Hospitalier Luxembourg, 1210 Luxembourg, Luxembourg; [12]Luxembourg Center of Neuropathology (LCNP), Luxembourg; [13]National Center of Pathology (NCP), Laboratoire National de Santé, L-3555 Dudelange, Luxembourg; [14]Department of Life Sciences and Medicine (DLSM), University of Luxembourg, L-4362 Esch-sur-Alzette, Luxembourg; [15]Microenvironment and Immunology Research Laboratory, Medical Center - University of Freiburg, Freiburg, Germany; [16]Department of Neurosurgery, Medical Center - University of Freiburg, Freiburg, Germany; [17]Department of Neurological Surgery, Lou and Jean Malnati Brain Tumor Institute, Robert H. Lurie Comprehensive Cancer Center, Feinberg School of Medicine, Northwestern University, Chicago, Illinois. [18]Department of Physics and Material Science, University Luxembourg, L-4367 Belvaux, Luxembourg; [19]Department of Neuroscience, University of California San Diego, La Jolla, CA 92093, USA

# Equal contribution; Correspondence: Anna.Golebiewska@lih.lu; Alessandro.Michelucci@lih.lu

## SUPPLEMENTARY MATERIAL AND METHODS

### 10XGenomics single-cell RNA-sequencing and analysis

Human tumor cells were processed via Drop-seq (P3, P8, P13, T101, T16) or 10XGenomics (T192, T233, T347, T470). Drop-seq and data preprocessing were performed as previously described[1,2]. For the 10XGenomics, purified single tumor cells were diluted to $10^6$ cells/ ml with RNAse free DPBS + 0.05 % FBS. Diluted cells were filtered using a 50µm filter. The viability of cells was verified using Beckman coulter automatic cell counter and C-Chip – Disposable Haemocytometer (NanoEntek) viability analyzer. Cell concentration was adjusted to target the encapsulation of 3000 cells according to 10XGenomics cell preparation guidelines. Chromium Next GEM Chip with single-cell suspension was loaded to the Chromium Controller (10XGenomics) for generation of encapsulated cells and GEMs. scRNA-seq libraries were prepared using the Chromium Next GEM Single Cell 3' GEM, Library & Gel Bead Kit v3.1 and a Chromium i7 Multiplex kit according to the manufacturer's protocol. Libraries were purified using SPRIselect magnetic beads and analyzed using Agilent 2100 Bioanalyzer. Sequencing was performed using NextSeq 500/550 High Output Kit v2.5 (75 Cycles). scRNA-seq analysis was performed in R (v4.1.1) with the Seurat package (v4.0.5)[3]. Barcode processing and UMI filtering were performed using the Cell Ranger 4.0.0. Average saturation of the sequencing was 30-40% as calculated by Cell Ranger. GBM molecular subtypes (Classical, Proneural and Mesenchymal) and cellular states (MES, AC, NPC and OPC-like) were determined using signatures listed in Wang et al., 2017 and Neftel et al., 2019 respectively. Single-cell signature scores were calculated using scrabble(v1.0.0) used in Neftel et al., 2019[4].

### Gene expression analysis by qPCR

Myelin was removed prior FACS-sorting with the Myelin removal beads II (Miltenyi) and $CD11b^+$ cells were FACS-sorted directly to Trizol® LS (Life Technologies) according to the manufacturer's protocol (n=2-3; from at least 3 animals each). RNA extraction and RT-PCR were conducted as previously described [5]. The sequences of the primers designed with the Primer-BLAST tool are as follows: *Spp1 F: CTGGCTGAATTCTGAGGGACT, R: TTCTGTGGCGCAAGGAGATT; P2ry12 F: GTGCAAGGGGTGGCATCTA, R: TGGAACTTGCAGACTGGCAT; Tmem119 F: TGCAATGTCGCTGTCACTCT, R: AGTTTGTGTTTCCACGGGGT Grp34 F: GGAAAGCTTCAACTCAGTTCCTG, R: TCCATGAGAGGAGCAAAGCC, Rpl27 F: ACATTGACGATGGCACCTC, R: GCTTGGCGATCTTCTTCTTG*

### Survival analysis

For the survival analyses, IDHwt GBMs were selected from the CCGA datasets[6]. Mouse gene symbols were converted from Mouse MGI symbols into human HGNC symbols using BioMart. Scores for the signatures used were computed for each sample using the ssGSEA method from the package GSVA (v.1.38.2)[7] in a purrr tidyverse environment leading to a matrix of enrichment score of each signature (rows) and for each sample (columns). The samples have been stratified into "high-score" or "low-score" by splitting the top 25% highest ssGSEA scores and the top 25% lowest ssGSEA score respectively. The stratified samples were subjected to the Kaplan-Meier analysis in order to estimate the survival with the package *survival* (v3.2-11).

## SUPPLEMENTARY FIGURES

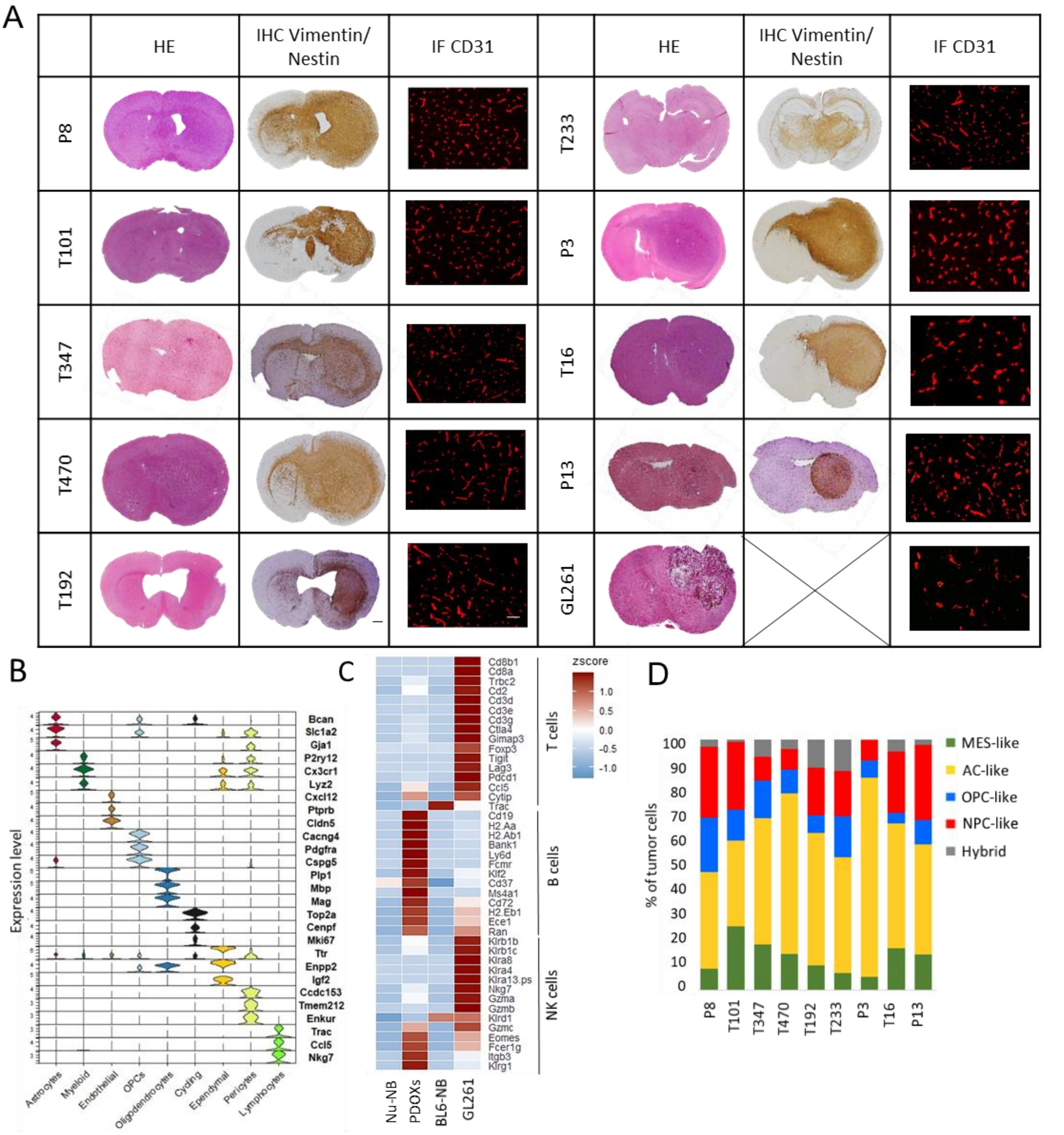


**Fig. S1. Characterization of TME diversity in PDOX and GL261 GBM models. (A)** Hematoxylin and Eosin (H&E), human-specific Vimentin/Nestin and mouse-specific CD31 staining of PDOX cohort and GL261 models. Human-specific Vimentin/Nestin staining highlights varying levels of invasion in PDOX models, while mouse-specific CD31 staining shows aberrant blood vessels in the GBM models. Scale bars represent 1mm (black) and 100µm (white). **(B)** Violin plots showing expression of marker genes specific to distinct cell types identified in the scRNA-seq dataset (the biological group: nude mouse normal brain (Nu-NB), PDOXs (9 models), C57BL6/N mouse normal brain (BL6-NB), GL261 tumor (3 collection time points: early, middle, and late). Three top markers of each population are displayed (Wilcoxon Rank Sum Test). **(C)** Analysis of lymphocyte cell cluster. Heatmap shows the z-score of the average expression of marker genes for T, B and NK cells in lymphocytes detected in each sample group. **(D)** Human tumor cells of PDOX models assigned to four GBM transcriptomic states [4]: mesenchymal-like (MES-like), astrocytic-like (AC-like), oligodendrocyte progenitor cell-like (OPC-like) and neural progenitor cell-like (NPC-like). Cells with shared states were grouped as “Hybrid”.

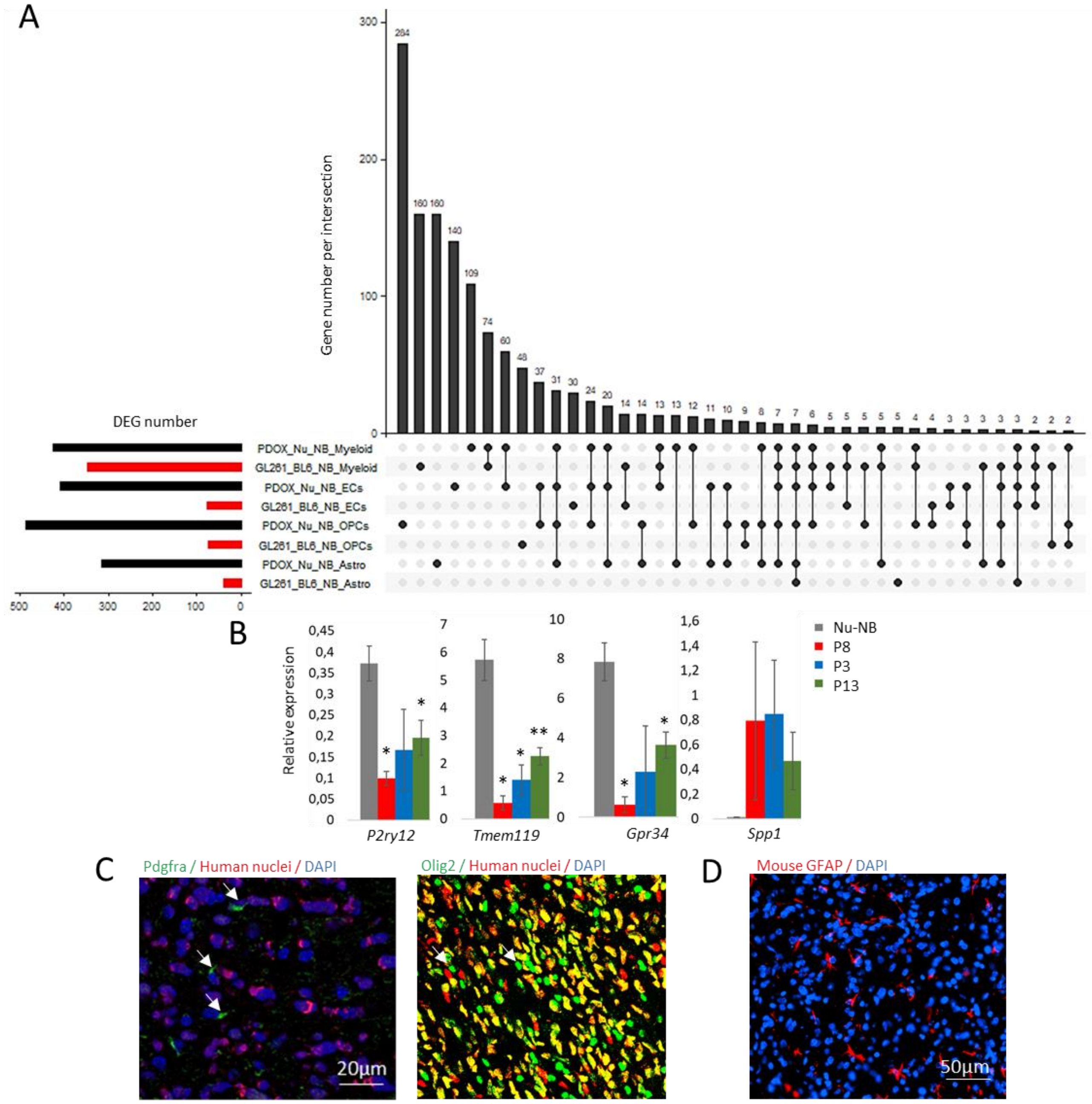


**Fig. S2. Tumor-specific activation of TME cell types. (A)** Upset plot showing differentially expressed genes (DEGs) between TME of PDOX and GL261 versus corresponding normal brains in identified cell types (FDR ≤0.01, $|\log_2 FC| \geq 1$, Wilcoxon rank sum test with Benjamini-Hochberg correction). Bar chart depicts the number of differentially expressed genes shared between the different groups. **(B)** Decreased expression of homeostatic myeloid markers (*P2ry12*, *Tmem119, Gpr34*) and increased levels of activated myeloid marker (*Spp1*) were validated by qPCR in CD11b$^{+}$ myeloid cells isolated as bulk from TME of representative PDOX models (P3, P8, P13) and nude normal brain (Nu-NB); graphs show bulk mean relative expression levels ± SEM (n=2-5, **p<0.01 *p<0.05, two-tailed Student's t-test). *Rpl27* was used as a housekeeping gene. **(C)** Immunofluorescence staining showing Pdgfrα$^{+}$ and Olig2$^{+}$ cells in the tumor core of P8 PDOX. Arrows point Pdgfrα$^{+}$ and Olig2$^{+}$ mouse cells, negative for human nuclei staining. **(D)** Immunofluorescence staining showing mouse GFAP$^{+}$ astrocytic cells in the tumor core of T192 PDOX.

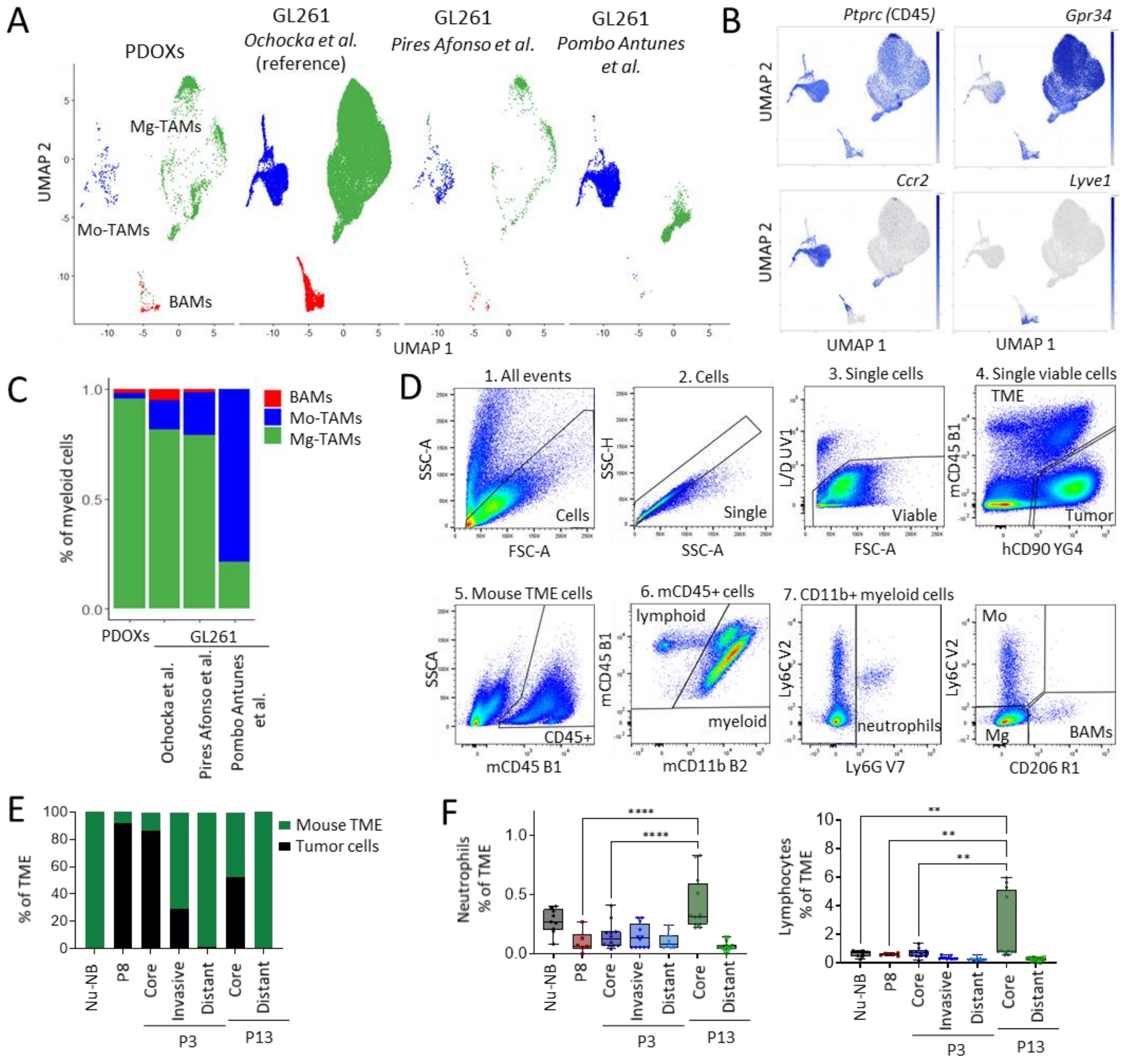


**Figure S3. Myeloid cell heterogeneity and ontogeny in human and mouse-derived GBM TME. (A)** Split UMAP projection of myeloid cells from different datasets. Myeloid cell types are color-coded (BAMs = red, Mg-TAMs = green, Mo-TAMs = blue). **(B)** UMAPS showing expression of marker genes: pan-immune *Ptprc* (CD45), Mg *Gpr34*, monocytes *Ccr2*, BAMs *Lyve*. The color gradient represents expression levels. **(C)** Proportions of myeloid cell types in PDOXs and GL261 for three independent datasets [2,8,9]. Percentage of Mo-TAMs depends on the tumor stage and dissection strategy. **(D)** Gating strategy for flow cytometry. Example is shown for P13 PDOX tumor: (1) Cells were distinguished from debris based on the Forward Scatter (FSC) and Side Scatter (SSC). (2) Cell aggregates were gated out based on their properties displayed on the SSC area (SSC-A) versus height (SSC-H) dot plot. (3) Dead cells were recognized by their strong positivity for the dead cell marker (L/D) (4) Human tumor cells were recognized as CD90 positive with human-specific antibody. TME was of mouse origin. (5) Immune cells were recognized as CD45 positive within mouse-derived TME. (6) Lymphocytes were recognized as CD11b negative population. Negative gating includes also CD11b$^{low}$ NK cells. CD11b positive cells correspond to myeloid cells. (7) CD11b$^{+}$Ly6C$^{low}$Ly6G$^{+}$ cells represent neutrophils. (8) Ly6C and CD206 allows distinguishing Ly6C$^{+}$CD206- Mo-TAMs, Ly6C$^{-}$CD206$^{-}$ Mg-TAMs and Ly6C$^{-}$CD206$^{+}$ BAMs. **(E)** Ratio of human tumor cells over mouse-derived TME across the different tumor phenotypes and zones. The ratio of tumor cells to TME was higher in the tumor core compared to invasive zone and distal brain regions, reflecting a similar tumor core mass in all PDOX phenotypes. **(F)** Box plots show flow cytometry quantification of CD45$^{+}$CD11b$^{+}$Ly6G$^{+}$Ly6C$^{low}$CD206$^{-}$ neutrophils and CD45$^{+}$CD11b$^{-}$ lymphocytes in Nu-NB and PDOX TME across different tumor phenotypes and brain regions (n=6-15 from at least three different mouse brains each, one-way ANOVA, ***p<0.001, **p<0.01, *p<0.05).

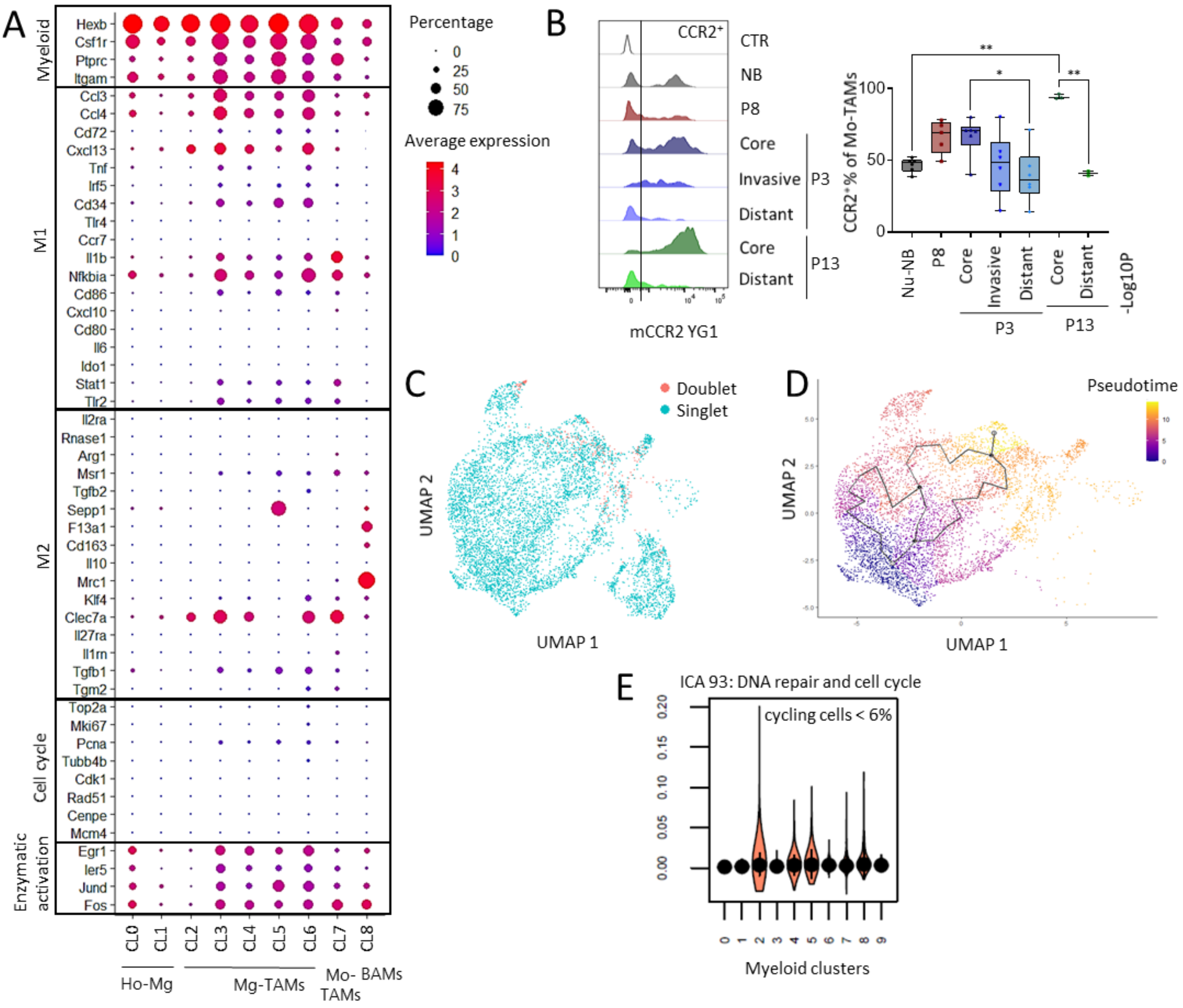


**Figure S4. Analysis of heterogeneous TAMs in PDOXs. (A)** Expression levels of exemplary genes representing myeloid pan-markers, M1/M2 markers, cell cycle and enzymatic activation in Nu-NB, PDOXs (9 models combined), BL6-NB, GL261 tumor (3 time points combined). **(B)** Representative flow cytometry graphs of CD45$^+$CD11b$^+$Ly6G$^-$Ly6C$^+$CD206$^-$ Mo-TAMs showing increased levels of CCR2 in tumor core of three PDOX models. Right: quantification of CCR2$^+$ cells in Mo-TAMs across different tumor phenotypes and brain regions. (n=5-6 from at least three different mouse brains each, one-way ANOVA, ***p<0.001, **p<0.01, *p<0.05). **(C)** Scoring for putative doublets in the myeloid cluster. **(D)** Monocle-based trajectory inference analysis of Mg-TAM clusters showing a trajectory from Ho-Mg to Mg-TAMs. **(E)** Independent component analysis. A standard application with 32 components did not reveal cell cycle component. Only with a high number of components (128), one component was linked to DNA repair and mitotic cell cycle, highlighting less than 6% of potentially cycling cells.

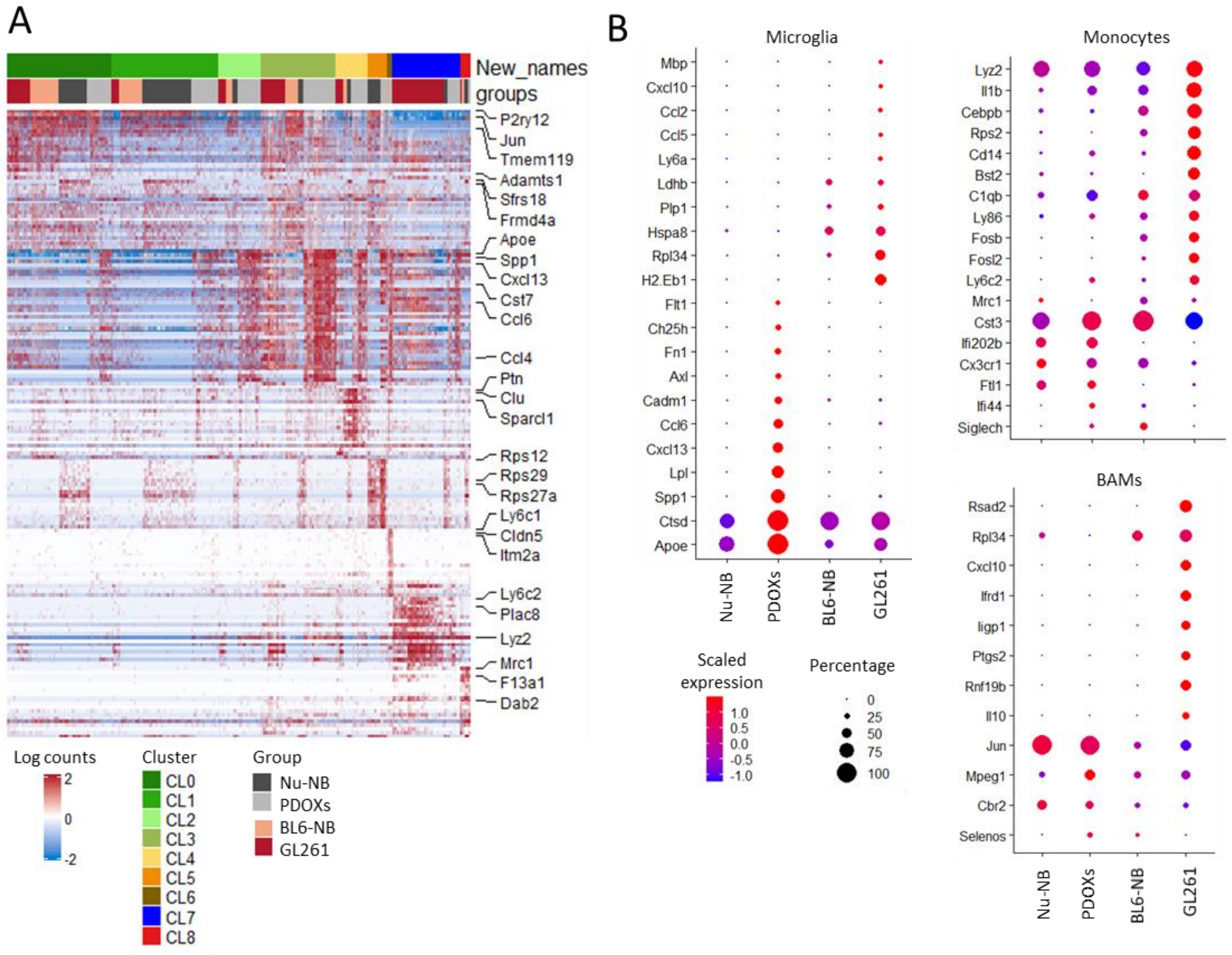


**Figure S5. Comparison of transcriptomic profiles of myeloid cells in PDOXs and GL261 models. (A)** Heatmap representing expression of marker genes of each myeloid transcriptomic state identified. No major differences were observed between myeloid cells of PDOXs and GL261. **(B)** Representative differentially expressed genes between PDOXs versus GL261 for Mg, Mo and BAMs (Wilcoxon rank sum test with Benjamini-Hochberg correction). Expression levels are shown for PDOXs (9 models combined), GL261 (3 time points combined) and their respective normal brain controls (Nu-NB= Nude normal brain; BL6-NB = C57BL6/N normal brain).

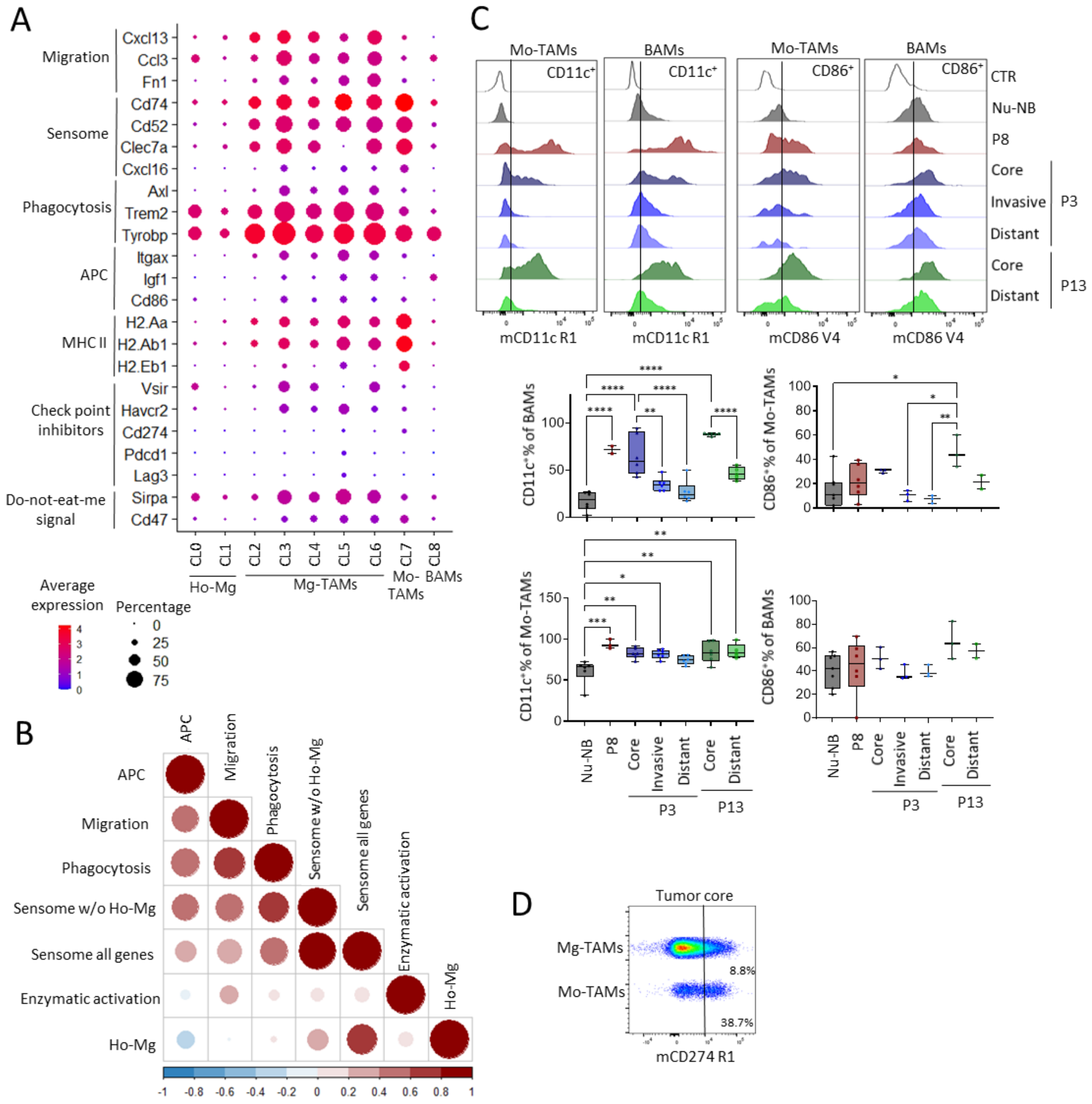

**Figure S6. Analysis of functional markers in TAMs. (A)** Expression levels of representative genes for migration, sensome (GBM specific), phagocytosis, antigen presentation (APC) and checkpoint inhibition. **(B)** Spearman correlation analysis between functional signatures in myeloid cells. **(C)** Left: representative flow cytometry graphs of $CD45^+CD11b^+Ly6G^-Ly6C^+CD206^-$ Mo-TAMs and $CD45^+CD11b^+Ly6G^-Ly6C^-CD206^+$ BAMs showing levels of CD11c and CD86 in nude mouse normal brain (Nu-NB) and three PDOX models. Unstained control is shown for each population (CTR). Bottom: quantification of $CD11c^+$ and $CD86^+$ cells in Mo-TAMs and BAMs across different tumor phenotypes and brain regions (n=3-6 from at least three different mouse brains each, one-way ANOVA, ***p<0.001, **p<0.01, *p<0.05). **(D)** Representative flow cytometry graphs showing expression of PDL-1 in Mg-TAMs and Mo-TAMs in tumor. Examples shown for T304 PDOX.

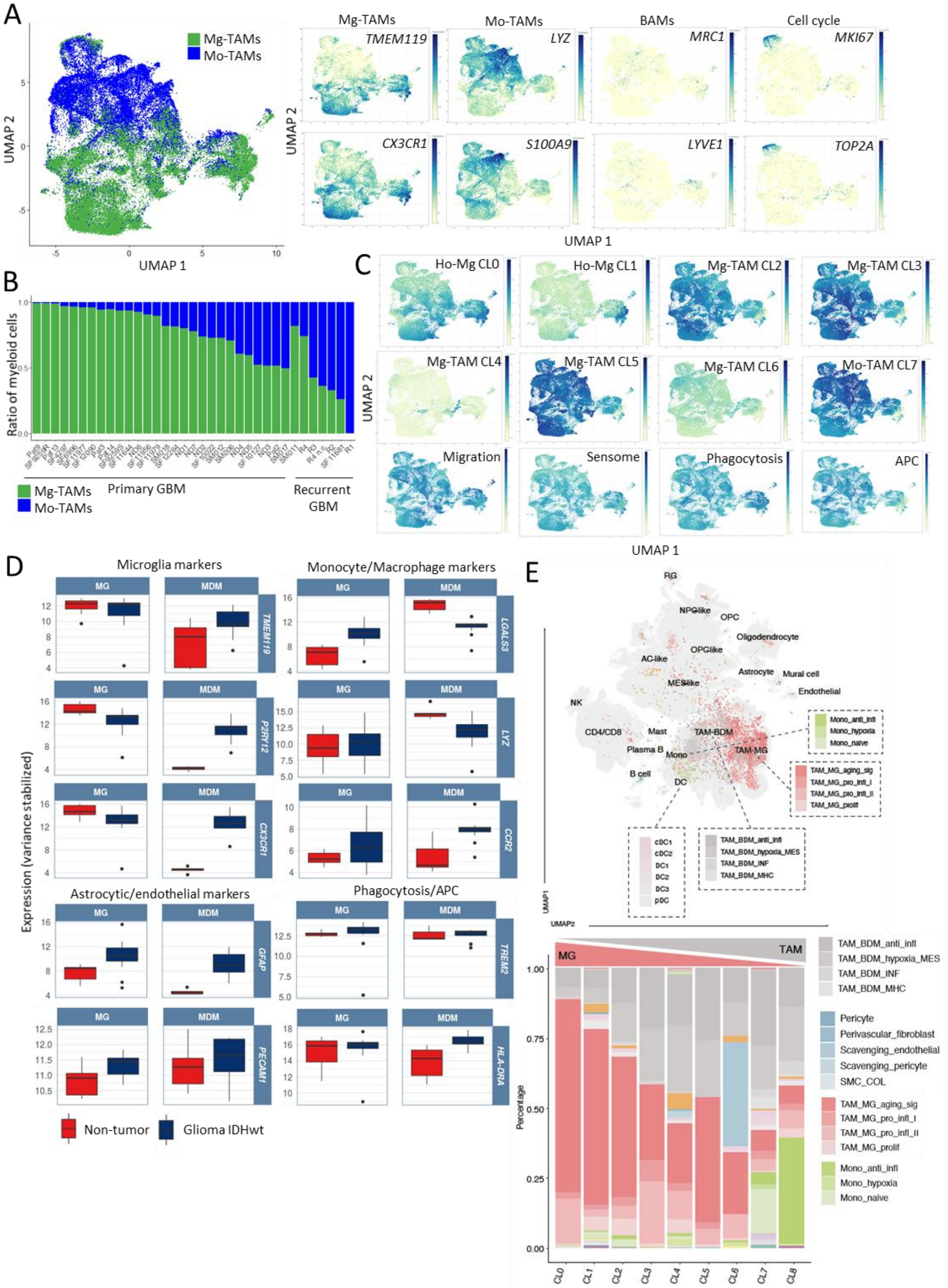


**Figure S7. Analysis of Mg/Mo-TAMs in GBM patient tumors. (A)** UMAP projection of myeloid cells in GBM patient tumors. Cells are color coded for Mg-TAMs and Mo-TAMs ontogeny based on established gene signatures. Inserts show expression levels of marker genes. The color gradient represents expression levels. **(B)** Proportions of Mg-TAMs and Mo-TAMs in individual GBM patient tumors. Recurrent GBMs show higher proportions of Mo-TAMs ($p<0.01$, two-tailed Student's t-test). **(C)** UMAP projection of human GBM myeloid cells displaying expression levels of cluster (CL0-8) and functional signatures identified in PDOX TME. The color gradient represents expression levels. (**D**) Expression of exemplary Mg, Mo, astrocyte and endothelial cell markers showing convergence of Mg and Mo towards heterogeneous TAMs in GBM tumors. Bulk RNA-seq analysis was performed in purified CD49d$^{+}$ (MG) and CD49$^{-}$ (MDM) myeloid cells[10] representing Mg and Mo in normal brain and GBM patient tumors. While Mg decreases homeostatic genes, Mo acquire microglia profiles within GBM tumors. Reversed convergence is observed for monocytic markers. (**E**) Mapping of mouse myeloid cells to the scRNA-seq profiles of human GBMs (GBmap[11]). Mg states (CL0-CL6) predominantly mapped to

previously identified human Mg states, although Mg-TAMs (CL3-5) showed increased proportion of TAMs associated with blood-derived macrophages, highlighting strong convergence of the Mg and Mo towards comparable TAM signatures. The astrocytic-like CL4 state is clearly associated with human myeloid compartment, whereas the endothelial-like CL6 state only partially overlaps with pericytes and endothelial cells. Mo-TAMs (CL7) and BAMs (CL8) showed a stronger association with Mo signatures.

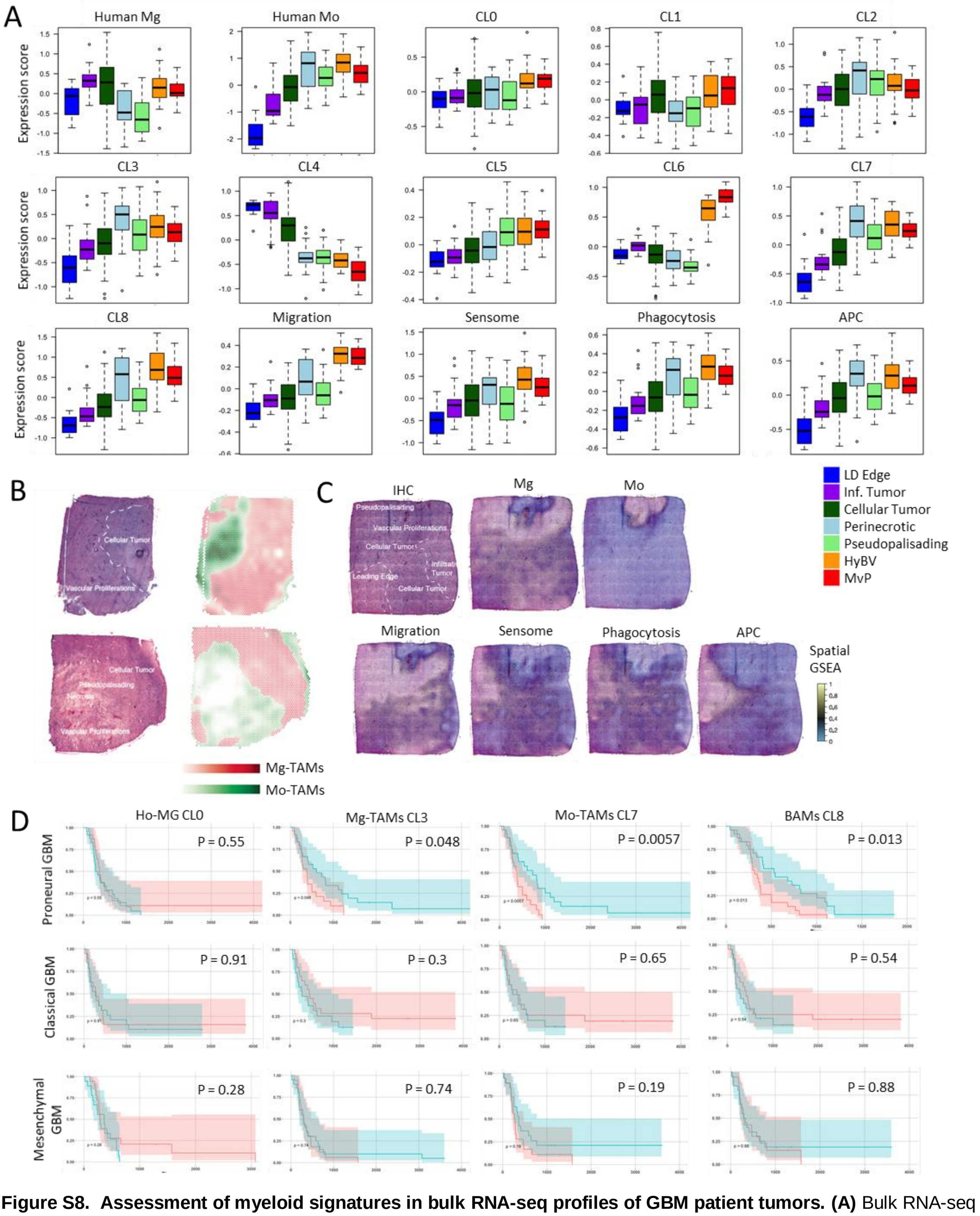


**Figure S8. Assessment of myeloid signatures in bulk RNA-seq profiles of GBM patient tumors. (A)** Bulk RNA-seq profiles of laser-micro dissected regions of GBM patient tumors. Box plots show signatures of Mg/Mo-TAMs, phenotypic states and functional signatures per tumor niche (LD edge: leading edge; Inf tumor: infiltrative tumor; HyBV: hyperplastic blood vessels in cellular tumor; MvP: microvascular proliferation). Data extracted from the Ivy Glioblastoma Atlas Project. **(B-C)** Surface plots of spatial localization of myeloid cell signatures in GBM patient tumors. **(D)** Kaplan–Meier survival analysis in GBM patients (CGGA-GBM IDHwt divided into transcriptomic subtypes: Classical, Mesenchymal and Proneural) with high- and low-enriched signatures (25 percentile top/bottom).

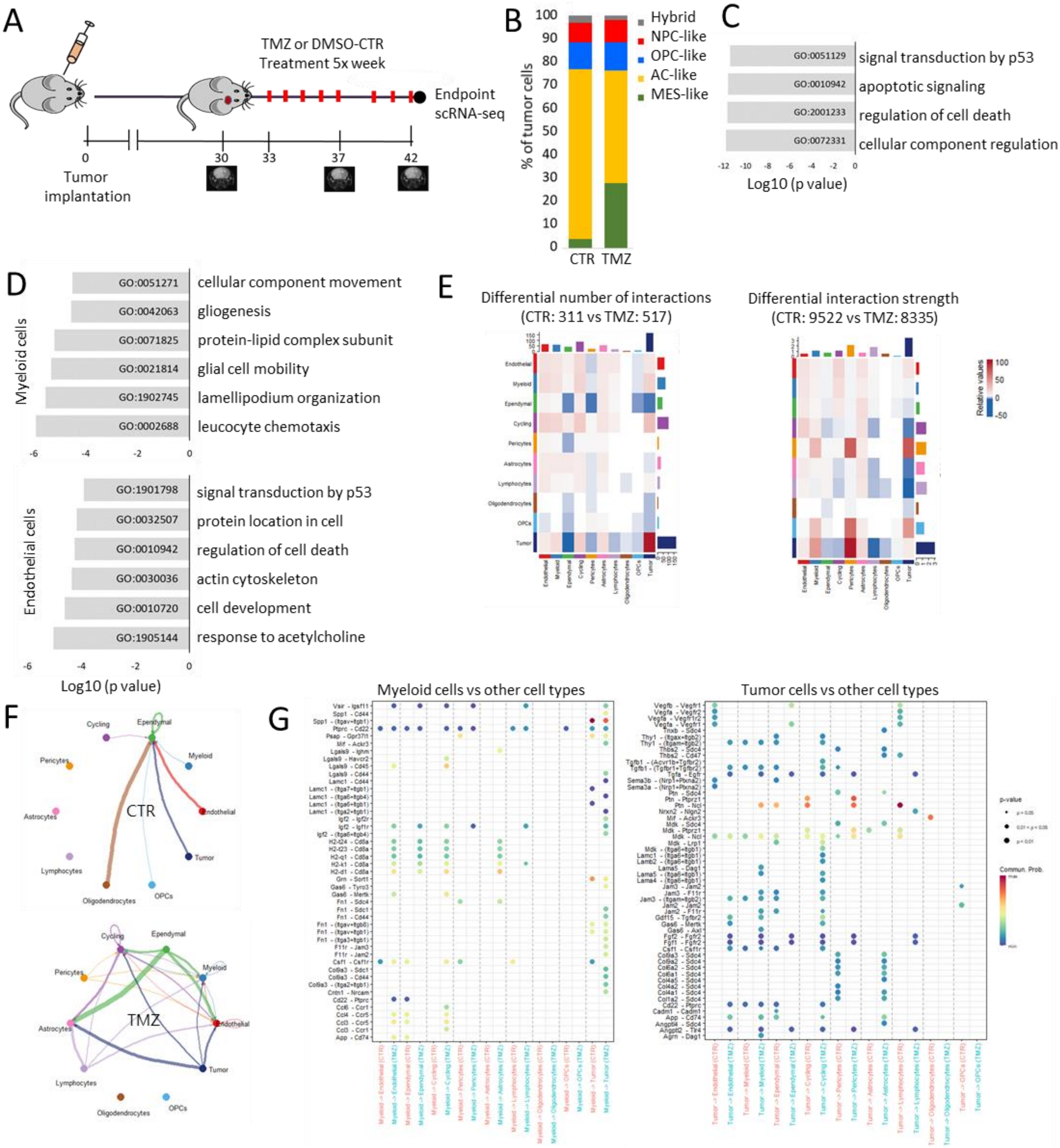


**Figure S9. GBM ecosystem upon TMZ treatment. (A)** Schematic illustration of the TMZ treatment regimen *in vivo*. Day 0 = tumor implantation, day 30 = MRI validation of tumor growth, day 33= treatment start, day 37= intermediary MRI, day 42 = endpoint. **(B)** Assessment of GBM cellular states[4] at single cell level in TMZ-treated and CTR GBM cells. **(C)** Summary of top four gene ontology terms characterizing DEGs in TMZ-treated versus CTR GBM cells (FDR ≤0.01, |logFC| ≥ 0.5 the Wilcoxon rank sum test with Benjamini-Hochberg correction). **(D)** Summary of top six gene ontology terms characterizing DEGs in TMZ-treated versus CTR myeloid and endothelial cells forming TME in P3 PDOX (FDR ≤0.01, |logFC| ≥ 0.5). **(E)** CellChat-based differential number of interactions and interaction strength, visualized as heatmaps, where red represents increased and blue decreased signaling in TMZ treated compared to CTR PDOXs. The top-colored bar plot represents the sum of a column of values displayed (incoming signaling). The right-colored bar plot represents the sum of a row of values (outgoing signaling). **(F)** Interactions related to the APP pathway in CTR and TMZ conditions. **(G)** Predicted ligand-receptor interactions based on communication probability are shown for signals outgoing from myeloid and tumor cells.

## SUPPLEMENTARY REFERENCES

1 Golebiewska, A., Hau, A. C., Oudin, A. *et al.* Patient-derived organoids and orthotopic xenografts of primary and recurrent gliomas represent relevant patient avatars for precision oncology. *Acta Neuropathol* **140**, 919-949, doi:10.1007/s00401-020-02226-7 (2020).
2 Pires-Afonso, Y., Muller, A., Grzyb, K. *et al.* Elucidating tumour-associated microglia/macrophage diversity along glioblastoma progression and under ACOD1 deficiency. *Mol Oncol* **16**, 3167-3191, doi:10.1002/1878-0261.13287 (2022).
3 Hao, Y., Hao, S., Andersen-Nissen, E. *et al.* Integrated analysis of multimodal single-cell data. *Cell* **184**, 3573-3587 e3529, doi:10.1016/j.cell.2021.04.048 (2021).
4 Neftel, C., Laffy, J., Filbin, M. G. *et al.* An Integrative Model of Cellular States, Plasticity, and Genetics for Glioblastoma. *Cell* **178**, 835-849 e821, doi:10.1016/j.cell.2019.06.024 (2019).
5 Sousa, C., Golebiewska, A., Poovathingal, S. K. *et al.* Single-cell transcriptomics reveals distinct inflammation-induced microglia signatures. *EMBO Rep*, doi:10.15252/embr.201846171 (2018).
6 Zhao, Z., Zhang, K. N., Wang, Q. *et al.* Chinese Glioma Genome Atlas (CGGA): A Comprehensive Resource with Functional Genomic Data from Chinese Glioma Patients. *Genomics Proteomics Bioinformatics* **19**, 1-12, doi:10.1016/j.gpb.2020.10.005 (2021).
7 Hanzelmann, S., Castelo, R. & Guinney, J. GSVA: gene set variation analysis for microarray and RNA-seq data. *BMC Bioinformatics* **14**, 7, doi:10.1186/1471-2105-14-7 (2013).
8 Pombo Antunes, A. R., Scheyltjens, I., Lodi, F. *et al.* Single-cell profiling of myeloid cells in glioblastoma across species and disease stage reveals macrophage competition and specialization. *Nat Neurosci* **24**, 595-610, doi:10.1038/s41593-020-00789-y (2021).
9 Ochocka, N., Segit, P., Walentynowicz, K. A. *et al.* Single-cell RNA sequencing reveals functional heterogeneity of glioma-associated brain macrophages. *Nat Commun* **12**, 1151, doi:10.1038/s41467-021-21407-w (2021).
10 Klemm, F., Maas, R. R., Bowman, R. L. *et al.* Interrogation of the Microenvironmental Landscape in Brain Tumors Reveals Disease-Specific Alterations of Immune Cells. *Cell* **181**, 1643-1660 e1617, doi:10.1016/j.cell.2020.05.007 (2020).
11 Ruiz-Moreno, C., Salas, S. M., Samuelsson, E. *et al.* Harmonized single-cell landscape, intercellular crosstalk and tumor architecture of glioblastoma. *bioRxiv*, 2022.2008.2027.505439, doi:10.1101/2022.08.27.505439 (2022).

**Table S1.** Characteristic of PDOX models applied in the study.

| PDOX model | Patient | Patient treatment | Genetic aberrations | Glioma-specific gene mutations | DNA methylation profiles | MGMT promoter methylation | PDOX phenotypic characteristics |
|---|---|---|---|---|---|---|---|
| P3 | BER0003 | na | +[Chr7, 19p, 20q], -[1p36-1p34.1, 1q21.1-q44, -5p15.33-31, Chr9, Chr10, 11p15-14, 20p] --CDKN2A/B | TP53 (C176Y), PTEN (D236fs) | GBM, IDHwt, RTK I/II | methylated | intermediate |
| P8 | BER0008 | na | ++EGFR, +[5p15.3-p12, 5q31.1-q35.3, Chr7, 8q24], -[ 6q21-q27, Chr10, 13q13.3-q34, 15q21.2-q23, 18q21.2-q22.4], --CDKN2A/B | EGFR (A289T), ATRX (S1348T), MET (A954S), PTEN (T319fs) | GBM, IDHwt, RTK II/I | methylated | invasive |
| P13 | BER0013 | na | +(Chr7, Chr19, Chr20), -(1p21.1-p31.2, 6q16.3-q21, Chr10, Chr13, 17q11-12), --CDKN2A/B | PTEN (D107V) | GBM, IDHwt, RTK II | methylated | angiogenic |
| T16 | LIH0016 | treated | ++[EGFR, MDM4], +7q, - [6q, Chr10, 13q11-q31.1], --CDKN2A/B | EGFR ex25-27del | GBM, IDHwt, RTK II | unmethylated | intermediate |
| T101 | LIH0101 | treatment-naive | ++[EGFR, MDM2] +[Chr1, Chr7, Chr9, Chr13, Chr16, Chr17, Chr19] -[3q, Chr4, Chr6, Chr10, Chr11, 14q11, Chr15], --CDKN2A/B, | | GBM, IDHwt, RTK II | methylated | invasive |
| T192 | LIH0192 | treatment-naive | ++[EGFR, 2q34], +[1q21.2-24.2, Chr7, Chr19, Chr20], - [9p24.3 - 13.3, Chr10] | EGFR complex rearrangements (ex1-6), ex14-16 | GBM, IDHwt, RTK II | unmethylated | invasive |
| T233 | LIH0192 | treated | ++[EGFR, 2q34], +[1q21.2-24.2, Chr7, Chr19, Chr20], - [9p24.3 - 13.3, Chr10] | EGFR vIII, complex rearrangements (ex1-6), ex14-17 | GBM, IDHwt, RTK II | unmethylated | invasive |
| T347 | LIH0347 | treatment-naive | ++EGFR, +[Chr7, 19p, Chr20], - [1p36.3-p34.3, 1q32.3-q44, Chr10, 13q12.11-31.1, 14q11.2-q21.3], --CDKN2A/B | EGFR ex2-15del, PTEN (C304fs) | GBM, IDHwt, RTK II | unmethylated | invasive |
| T470 | LIH0347 | treated | ++EGFR, +[Chr7, 19p, Chr20], - [ 1p36.3-p34.3, 1q32.3-q44, 1p31.1-p11.21, Chr10, 13q12.11-31.1, 14q11.2-q21.3], --CDKN2A/B | EGFR ex2-15del, PTEN (C304fs) | GBM, IDHwt, RTK II | unmethylated | invasive |

**Table S2.** List of antibodies used in the study.
*Flow cytometry test $10^6$ cells/100µl

| Antibody | Supplier | Catalog number | Concentration used |
|---|---|---|---|
| hCD90 PE-Cy7 | BD | 561558 | 5 µl/test* |
| hCD90 BV605 | BD | 562685 | 5 µl/test* |
| CD16/32 | eBioscience | 14-0161-85 | 1 µl/test* |
| mCD45 FITC | eBioscience | 11-0454-82 | 1 µl/test* |
| mCD11b Percp-Cy5.5 | BD | 550993 | 5 µl/test* |
| mLy6C PB | Biolegend | 128014 | 0.5 µl/test* |
| mLY6G BV785 | Biolegend | 127645 | 2.5 µl/test* |
| mCCR2 PE | R&D | FAB5538P | 5 µl/test* |
| iso IgG PE | R&D | IC108P | 5 µl/test* |
| mCD206 APC | Biolegend | 141708 | 2.5 µl/test* |
| mCD11c APC-Cy7 | Biolegend | 117323 | 5 µl/test* |
| mCD86 BV605 | Biolegend | 105037 | 5 µl/test* |
| iso IgG2a BV606 | Biolegend | 400540 | 5 µl/test* |
| m I-A/I-E APC-Cy7 | Biolegend | 107628 | 1 µl/test* |
| mCD45 BV605 | Biolegend | 11045182 | 1 µl/test* |
| LIVE/DEAD™ Fixable Near-IR | Invitrogen | L34975 | 0,1 µl/test* |
| Nestin | Abcam | AB6320 | IHC:1/500 |
| Vimentin | Thermo Fisher Scientific | Mab3400 | IHC:1/200 |
| CD31 | Cell Signaling | 77699 | IHC:1/100 |
| Iba1 | Biocare Medical | CP 290A | IHC:1/1000 |
| CD11c | Abcam | ab11029 | IHC:1/100 |
| GFAP | Dako | Z0334 | IHC:1/1000 |
| MHC-II | Abcam | ab25333 | IHC:1/100 |
| Pdgfra | Biolegend | 135905 | IHC:1/100 |
| Anti-Rat IgG Alexa Fluor 555 | Invitrogen | A21434 | IHC:1/1000 |
| Anti-Rabbit IgG Alexa Fluor 555 | Thermo Fisher Scientific | A-11039 | IHC:1/500 |
| Anti-Mouse IgG Alexa Fluor 647 | Thermo Fisher Scientific | A-11037 | IHC:1/500 |
| Anti-Rat IgG Alexa Fluor 488 | Thermo Fisher Scientific | A-21244 | IHC:1/500 |
| Opal 520 Flurophore | Akoya Biosciences | NEL810001KT | IHC:1/100 |
| Opal 570 Flurophore | Akoya Biosciences | NEL810001KT | IHC:1/100 |

**Table S3.** Lists of differentially expressed genes between PDOXs and nude brain per each major cell type. Differentially expressed genes were defined at threshold: FDR <=0.01 and $|log_2FC|>=1$
Genes are listed from the highest to lowest |log2FC|

| Cell type | Astrocytes | | Endothelial cells | | Ependymal cells | | Myeloid cells | | Oligodendrocytes | | OPCs | | Pericytes | |
|---|---|---|---|---|---|---|---|---|---|---|---|---|---|---|
| Fold change | upregulated | downregulated | upregulated | downregulated | upregulated | downregulated | upregulated | downregulated | upregulated | downregulated | upregulated | downregulated | upregulated | downregulated |
| **Number of genes** | 186 | 129 | 304 | 105 | 21 | 22 | 287 | 136 | 7 | 124 | 286 | 199 | 2 | 71 |
| | Gm42418 | Sfrs18 | Gm42418 | Plp1 | Tmsb4x | Gm26924 | Spp1 | Sfrs18 | Olig1 | Igfbp5 | Pdgfra | Plp1 | Gm42418 | Wdr96 |
| | Cryab | Ppap2b | Rpl14.ps1 | Gm26924 | Gm42418 | Sez6 | Gm42418 | Snord49b | Pdgfra | Mal | Gm42418 | Apod | Gm12346 | Gm26924 |
| | Vim | Gm26924 | Adgrf5 | Gpr116 | Lyz2 | Vps8 | Cxcl13 | Sepp1 | Gria2 | Plp1 | Lhfpl3 | Npy | | 1700009P17Rik |
| | Tmsb4x | Gm26339 | Selenop | Sfrs18 | Tyrobp | Rpl35 | Clec7a | Gm26924 | Cspg5 | Phldb2 | Pnisr | Sepp1 | | 1700026D08Rik |
| | Pnisr | Snord42a | Gm6977 | Ttyh1 | Hexb | Vimp | Gm6977 | 4632428N05Rik | Cacng4 | Cd200 | Gap43 | Igfbp5 | | Ptplad1 |
| | Plpp3 | Xist | Gm5905 | Sepp1 | C1qa | D3Bwg0562e | Gm5905 | Epb4.1l2 | mt.Cytb | 4922501C03Rik | Cspg5 | Slc4a4 | | Stxbp3a |
| | Rack1 | Gm3764 | Gm9794 | Fabp7 | C1qb | Gm6625 | Gm14303 | Gm26339 | Actb | Slc4a4 | 3110035E14Rik | Clca1 | | A330021E22Rik |
| | Gm6977 | 2810055G20Rik | Rpl37rt | Igfbp5 | B2m | Calml4 | Gm9794 | Entpd1 | | Ptn | Rpl14.ps1 | Gja1 | | Wdr52 |
| | Gm9625 | Slc38a3 | Gm11478 | mt.Tc | Ctss | BC023829 | Rpl14.ps1 | Abca9 | | Sash1 | Gm5905 | Ptn | | Ccdc135 |
| | Rpl13.ps5 | D4Wsu53e | Gm4617 | Fut9 | C1qc | 1110001A16Rik | Ccl4 | D4Wsu53e | | Clca1 | S100a1 | Nr2f2 | | Ccdc19 |
| | Rps3a3 | Sepp1 | Gm15427 | D4Wsu53e | Ctsd | 0610011F06Rik | Rpl37rt | P2ry12 | | Mxi1 | Pllp | Ccdc80 | | 2610015P09Rik |
| | Gm14892 | Snord49b | Gm9385 | Ppap2b | Cd63.ps | Ppia | Lpl | Gpr34 | | Ccdc157 | Slc25a5 | Vtn | | Ccdc104 |
| | Gm7558 | Ptplad1 | Rps16.ps2 | Slc4a4 | Ctsb | Ccdc41 | Ttr | Il7r | | Tlr3 | Gng3 | Gm26924 | | Spag6 |
| | Fth.ps2 | Vegfa | Gm14303 | Eltd1 | Rpl14.ps1 | 1110059M19Rik | H2.Ab1 | Ddx26b | | Sepp1 | Scg5 | Frzb | | Rps17 |
| | Rpl14.ps1 | Sc4mol | Gm5805 | Dcn | Gm9794 | Rpl29 | Gm15427 | P2ry13 | | Ttyh1 | Ramp1 | Ttyh1 | | Sfrs18 |
| | Rps5 | Slco1c1 | Rpl32 | Gm26339 | Wdr89 | 4922501C03Rik | Fth1 | Csmd3 | | Lifr | Rps5 | Apoe | | Ifltd1 |
| | Gm12346 | Adrbk2 | Gm4149 | Rps6 | Rack1 | Gm9493 | Gm11478 | Gm17087 | | Frzb | Gm9385 | Hmgcs2 | | 2510003E04Rik |
| | Gm12857 | 2610017I09Rik | Gm10080 | Snord49b | Lamp1 | Cox7b | Cd63.ps | Ccdc55 | | Wdr60 | Olig2 | Timp3 | | Gm872 |
| | Gm13215 | Slc1a2 | Gm5963 | Hnrnpa3 | Sparc | Stk39 | H2.Aa | Maf | | Timp3 | Rpl3.ps1 | Ncam1 | | Ppap2b |
| | Gm6136 | Vimp | Pnisr | Ptn | Cst3 | Selt | Rack1 | Gpr56 | | Trp53bp2 | Gm12346 | Inhba | | 2010015L04Rik |
| | Rps16.ps2 | Zfml | Rpl3.ps1 | Slc7a11 | Actb | Enpp2 | Cd74 | Hnrnpa3 | | Mybpc1 | Rpl37rt | Sash1 | | A030009H04Rik |
| | Gm13498 | Tmem66 | Adgrl4 | Vimp | | 4930546H06Rik | Ccl3 | Tmem66 | | Ralgps2 | Gm9794 | Iqub | | Pax3 |
| | Gm22567 | Syne1 | Rpl38.ps2 | Pcdhga9 | | | Gm16580 | Ppap2b | | Sfrs18 | Gm11478 | Col5a2 | | Vps28 |
| | Gm12183 | St6galnac5 | Tpt1.ps3 | Atp1a2 | | | Gm11942 | Zfhx3 | | Aspa | Tnr | Tspan15 | | D2Wsu81e |
| | Gm9385 | CT030684.1 | Gm9843 | Abca9 | | | Gm5963 | Jund | | Slc7a11 | Gm11560 | Stmn1 | | Gm6625 |
| | Gm5805 | Nim1 | Rack1 | Hes5 | | | Gm4149 | CT030684.1 | | Gja1 | Olig1 | Lpar1 | | Ccdc41 |
| | Rn7s6 | Fut9 | Rps24.ps3 | Slc1a3 | | | Rps24.ps3 | Zfml | | Plekha4 | Clu | Igf1 | | Selk |
| | Id1 | Cwc22 | Gm14586 | Cyp2j9 | | | Selenop | Nrip1 | | Rgag4 | Rack1 | Wls | | Lsmd1 |
| | Fth1 | Gnao1 | Gm6204 | Timp3 | | | Cst7 | Slco2b1 | | Gas7 | Gm15427 | Sparc | | 6720401G13Rik |
| | Gm12222 | Pcdhga9 | Gm11966 | Fam213a | | | Rps16.ps2 | Tmem119 | | Ahctf1 | Rsrp1 | Col23a1 | | B630005N14Rik |
| | Gm5370 | 2610203C20Rik | Rflnb | mt.Tp | | | Gm9385 | Vimp | | Inhba | Bcas1 | Slc7a11 | | AK129341 |
| | Gm11560 | Trim9 | Gm11560 | Nell2 | | | Gm5805 | Hpgds | | Fmo1 | Rps16.ps2 | Fam213a | | Gm26339 |
| | Gm12165 | S1pr1 | Rps13.ps2 | Scd2 | | | Rpl3.ps1 | Lrrc58 | | Gtpbp2 | Gm4617 | Sfrs18 | | Sept15 |
| | Gm13340 | Macf1 | Rpl36a.ps2 | Sash1 | | | Rps13.ps2 | Cx3cr1 | | Fbln2 | Tmem100 | Gm26339 | | Ankrd32 |
| | Rpl10l | 5830428H23Rik | Gm11942 | Ptgds | | | Pnisr | Il6ra | | Gm872 | Stmn3 | Hey2 | | Dpcd |
| | Gm11942 | Ttyh1 | Fn1 | Vegfa | | | Apoe | Cep110 | | Cldn5 | Gm12338 | Phldb2 | | CT030684.1 |
| | Rpl17.ps4 | Ank2 | Gm16580 | Gm17087 | | | Fn1 | Gm13476 | | Mdk | Calm1 | Cd59a | | Gm12942 |
| | Gm4332 | 4930546H06Rik | Wdr89 | Mllt4 | | | Ccl6 | Mertk | | Cyp4v3 | Nap1l5 | Gdpd4 | | Gm5918 |
| | Gm11810 | Ctage5 | Gm10073 | 2810055G20Rik | | | Gm6204 | Wapal | | Pla2g16 | Bex3 | Fmo1 | | Pet112 |
| | Rps15.ps2 | Dclk1 | Gm4332 | Slc1a2 | | | Gm8995 | Naalad2 | | Scd1 | Id2 | Pla2g16 | | Sc4mol |
| | Gm14450 | Malat1 | Rsrp1 | Colec12 | | | Rpl36a.ps2 | RP24.312B12.1 | | Wls | Pcdh15 | Mfap3l | | 2410018M08Rik |
| | Gm12696 | Clmn | Rps26 | Wapal | | | Cd52 | Rps23 | | Pbxip1 | Rpsa.ps10 | Atp1a2 | | I7Rn6 |
| | Gm6368 | Acss1 | Rplp0 | Mdk | | | H2.K1 | Snord42a | | Fam213a | Ranbp1 | Mybpc1 | | BC023829 |
| | Wdr89 | Itih3 | Slc38a5 | Gm14964 | | | Kctd12 | Rpl5 | | Igf1 | C1ql1 | Mal | | Ccdc37 |
| | Gm2225 | Vwa1 | Gm9800 | Mboat2 | | | Epb41l2 | Ppp1r9a | | Dab2 | Cd81 | Col27a1 | | Epb4.1l4a |
| | Gm5963 | Pitpnc1 | Rplp1 | Rpl15 | | | Rpl10a.ps1 | Smap2 | | Col5a2 | Ostf1 | Dnajc1 | | 9330101J02Rik |
| | Ttr | Fam21 | Gm6863 | Suv420h1 | | | Rps5 | Jhdm1d | | Slc43a2 | Gria3 | Pex11a | | Epb4.1l2 |
| | Gm11472 | Cep110 | Rpl31.ps8 | Fbln2 | | | Ch25h | Rps29 | | Lysmd3 | Igfbp2 | Abca8a | | 4922501C03Rik |
| | Rps12.ps19 | 4930402H24Rik | Gm12338 | Rpl13a | | | Gm4617 | Rpl21 | | Fstl1 | Cox8a | Dock7 | | 2700089E24Rik |
| | Gm17909 | Appl2 | Rpsa.ps10 | Atp13a5 | | | Gm14586 | 2810055G20Rik | | Frg1 | Dynll1 | Fbln2 | | 1700094D03Rik |
| | Rac1 | Tril | Rps10.ps1 | Rpl5 | | | Gm4332 | Tanc2 | | Hey2 | Nxph1 | Rnd3 | | Bai3 |
| | Gm12152 | Epb4.1l2 | Gm10288 | Sept15 | | | Rsrp1 | Son | | Ncam1 | Selenok | Filip1l | | Sepn1 |
| | Cd63.ps | Mycbp2 | Gm6265 | 4933426M11Rik | | | Rpsa.ps10 | Slc16a6 | | Atp8a1 | Alcam | Golgb1 | | Lphn3 |
| | Gm5835 | Atp1a2 | Scgb3a1 | Gja1 | | | Ctsd | D15Ertd621e | | Acss1 | Gm4149 | Thrsp | | 2310044G17Rik |
| | Gm9843 | Zfp949 | Fth1 | Gpm6b | | | Lilrb4a | RP24.288C12.6 | | Cd82 | Actb | 2610203C20Rik | | Rpl24 |
| | Cald1 | Acsl6 | Ctla2a | Kcnj16 | | | Ifi27l2a | Arid4a | | Glul | Rps26 | Gas7 | | Nim1 |
| | Id3 | Gja1 | Rps5 | Kmt2a | | | Rps26 | A430104N18Rik | | Mfsd6 | Gm14586 | Glul | | 2410066E13Rik |
| | Hspd1.ps3 | Timp3 | Gm10275 | Tmem66 | | | Rps25.ps1 | Chd9 | | Selk | Gm6863 | Sdc4 | | Gm10311 |
| | Gm2178 | Son | Apold1 | Spag9 | | | Tpt1.ps3 | Numb | | Lgi1 | Tpt1.ps3 | Cry2 | | Gm13225 |
| | Gm9794 | Ccdc104 | Gm12183 | Ptms | | | Mt1 | Srgap2 | | Gramd1b | Emid1 | Scd1 | | 5430417L22Rik |
| | Map1b | Btbd17 | Gm7809 | Gprasp1 | | | Gm6030 | Elmo1 | | Npy | Ndufa4 | Hsd17b11 | | Gm17388 |
| | Gm44010 | Ddhd1 | Afdn | Morf4l1 | | | Calr.ps | Pwwp2a | | Sco1 | Igfbp3 | Vimp | | Gpr125 |
| | Rps19.ps6 | Suv420h1 | Gm15500 | Rpl10 | | | Lyz2 | Ctage5 | | Slc25a13 | Car8 | Mllt4 | | Grlf1 |
| | Gm5778 | Ppia | Gm5835 | Zbtb20 | | | Gm10288 | Col27a1 | | Adam23 | B2m | Gucy1b3 | | Leprel2 |
| | Gm4204 | Slc1a3 | Rps25.ps1 | Hspa8 | | | Wdr89 | Ogfrl1 | | Tgfbr1 | Rps13.ps2 | Fam3c | | Trp53rk |
| | Selenop | Ttc14 | Gm9892 | Htra1 | | | Gm10073 | Mycbp2 | | Fgd4 | Gm14303 | Gpr56 | | Zbed6 |
| | Gm15159 | Slc7a10 | Calr.ps | S1pr3 | | | H2.D1 | Scamp2 | | Tmem66 | Gm6977 | Abca5 | | Fam101a |
| | RP23.390K13.4 | Nrcam | Rpl18.ps1 | Rpl23a | | | Gm15500 | Lpar6 | | Nr2f2 | Pcsk1n | Edil3 | | Fam115a |
| | Gm11478 | Pdcd4 | Rpl10a.ps1 | Kcnj10 | | | Rpl31.ps8 | Marcks | | BC016423 | 2900011O08Rik | Huwe1 | | 9830147E19Rik |
| | Cxxc5 | Pla2g7 | Gm8894 | Ccdc80 | | | Rpl18.ps1 | Erbb2ip | | Zmat1 | Gm5805 | Ift57 | | Rom1 |
| | Bex3 | Fam115a | Gm8292 | Rps23 | | | Apoc1 | Rbm5 | | Gm26339 | Ndufb6 | Aldh2 | | Zfp191 |
| | Gm16580 | Gyk | Rps6.ps4 | Ncam2 | | | Gm5835 | Tmem100 | | Aim1 | Cd63.ps | Ahcyl2 | | |
| | Gm15896 | Csgalnact1 | Selenom | Gnb2l1 | | | AU020206 | Rps6 | | Dock7 | Ndufa8 | Efnb2 | | |
| | Gm27046 | Nrxn1 | Ly6c1 | Inhba | | | Gm9843 | Macf1 | | Tiam1 | Rpl22l1 | Grik3 | | |
| | Gm29228 | Hlf | Eif1.ps1 | Cwc22 | | | Gm8276 | Ythdc1 | | 4931406C07Rik | Banf1 | Mgst1 | | |
| | Cnot2 | Rorb | Gm12254 | Prrc2c | | | Rplp1 | Ssh2 | | Vtn | Ddah1 | Hmgn2 | | |
| | Gm9294 | D14Abb1e | Cd34 | Ncam1 | | | Tyrobp | Qk | | Osbpl11 | Ttr | Gm12688 | | |
| | Gm11517 | Flrt1 | Rps15a.ps5 | Zfml | | | Il1b | Hmgb1 | | Phf17 | Gpm6a | Sparcl1 | | |
| | Rpl34.ps2 | Frmd4a | Rps11 | Gpx4 | | | Ccrl2 | Med12l | | Hspa8 | Scrg1 | Fut9 | | |
| | Gm9521 | Fjx1 | Ttr | S100b | | | Kcnq1ot1 | Arl4c | | Cpt1a | Wdr89 | Mest | | |
| | Gm8513 | Acin1 | Tmsb4x | Rps17 | | | AC149090.1 | Cwc22 | | 5830418K08Rik | Gm12222 | Map4k4 | | |
| | Gm4321 | Arhgap5 | Gm3531 | Xist | | | Rpl32 | Dusp6 | | Tpr | S100a16 | Ptgds | | |
| | Gm15427 | Hnrnpa3 | B2m | 4932438A13Rik | | | Rps10.ps1 | Sall1 | | Apoe | Gm12696 | Plcb4 | | |
| | Gm18014 | Mfge8 | Ly6e | Ahcyl2 | | | Axl | Dip2b | | Tmem245 | Tuba1a | Slc27a1 | | |
| | Gm12482 | C030037D09Rik | Rps9 | RP24.288C12.6 | | | Id2 | Rps27a | | Hadha | Cdo1 | Mex3a | | |
| | RP23.408J7.2 | Tnik | Rps26.ps1 | Ddit3 | | | Vim | Frmd4a | | Atp1a2 | Tceal3 | Grin3a | | |
| | Gm17511 | Selk | Selenok | Ppia | | | Ctsb | Rps28 | | Nnat | Gm9892 | Cyp2d22 | | |
| | Gm6543 | Ubn2 | Gm12346 | Rps27 | | | Itgax | P4ha1 | | Snx24 | Gm6204 | Rgag4 | | |
| | Matr3.ps2 | Slc4a4 | Cdkn1a | Mga | | | Vsir | Morf4l1 | | Dnajc1 | Gm5778 | Fabp7 | | |
| | Gm7308 | Tmed5 | Rpl8 | Ccdc104 | | | Rps20 | Olfml3 | | Plk1s1 | Nrxn1 | Cd200 | | |
| | Gm5514 | Cadm2 | Selenof | Rps12 | | | Cd300c2 | Hspa8 | | Clca2 | Plpp3 | Wapal | | |
| | Gm4366 | Nwd1 | Gm12350 | Kdm5b | | | Gm6863 | Prrc2c | | Ccdc39 | Tmem176b | Syne2 | | |
| | Rsrp1 | Dio2 | Gm15772 | Igfbp2 | | | Ccl12 | Fau | | Stmn1 | Gm5835 | Ccnd2 | | |
| | Id2 | P4ha1 | Igfbp7 | Diap2 | | | Gm11560 | Map4k4 | | Cd55 | Calr.ps | D3Bwg0562e | | |

| | | | | | | | | | | | | | | |
|---|---|---|---|---|---|---|---|---|---|---|---|---|---|---|
| | Rps3 | Acsl3 | Pomp | Rpl21 | | | Gm14681 | Safb | | 2700089E24Rik | Cox6b1 | Nid1 | | |
| | S100a6 | Tex9 | Gm6472 | Apod | | | Ints6l | Arhgap5 | | Mfap3l | Mmp15 | Suv420h1 | | |
| | Gm3531 | Myo6 | Sparc | Nupr1 | | | mt.Cytb | Acin1 | | P2ry12 | Dynlt1.ps1 | Hadha | | |
| | Gm7618 | Sparcl1 | Slc25a5 | Wdr60 | | | mt.Nd1 | Ifngr1 | | Fabp7 | Cfap20 | Tlr3 | | |
| | Gm5905 | Mfn1 | Gm7027 | Tmx4 | | | Cd83 | Bin2 | | Fut9 | Ccnd1 | Clca2 | | |
| | Gm12912 | Gm973 | Gm6368 | Son | | | Gm10275 | Srrm2 | | Fam3c | Gm5963 | Dtx4 | | |
| | Gm13815 | Slc6a11 | Gm14681 | Ednrb | | | Gm9892 | Tsc22d4 | | Trim41 | Cfap36 | Abcb1a | | |
| | Rps11 | Kcnj16 | Ppfibp1 | Stmn1 | | | Rps15a.ps5 | Selplg | | Mecp2 | Tagln2 | Gm6483 | | |
| | Gm11488 | Ythdc1 | Rab1a | Casc4 | | | Gnas | Zfp292 | | Rbms3 | Gm6265 | Gpd1 | | |
| | Pabpc1 | Mdn1 | Rpl22.ps1 | Asph | | | Rpl38.ps2 | Mtdh | | Scd2 | H2.D1 | Slc1a3 | | |
| | Dclk2 | Ntrk2 | Gm4366 | Nr2f2 | | | Flt1 | Ccr5 | | P2rx4 | Rpl36a.ps2 | Slc13a3 | | |
| | Gm12020 | Sycp2 | Rpl9.ps7 | | | | Gm7027 | Adap2 | | E130311K13Rik | Hspd1.ps3 | Gpd2 | | |
| | Gm15773 | Trpm3 | Gm8995 | | | | Selenos | Abcb4 | | Lrig1 | Grm5 | Zbtb20 | | |
| | Calm2 | Wapal | Gm15421 | | | | Ank | Vwa1 | | Rnd3 | Pcp4 | Itsn2 | | |
| | App | AI464131 | Hsp25.ps1 | | | | Gm12338 | Ivns1abp | | Paqr6 | Cacng4 | Chd9 | | |
| | Gm6204 | Mmp14 | Gm14539 | | | | Rps6.ps4 | Plcl2 | | Ltn1 | Meg3 | Sat1 | | |
| | Calr.ps | Pfkp | Plpp3 | | | | B2m | Pde3b | | Lama4 | Dnm3 | 4922501C03Rik | | |
| | Vcan | Neat1 | Gm6563 | | | | Srgn | Sox4 | | Nek9 | Rab31 | Hnrnpa3 | | |
| | Spp1 | Nebl | Anp32.ps | | | | Gm12346 | Fchsd2 | | Son | Slc38a3 | Ptms | | |
| | Marcks | Chrdl1 | Tpm3.rs7 | | | | Atf3 | Rps27 | | Slc16a1 | Pdcd5.ps | Tspan18 | | |
| | Fos | Dtna | Gm12696 | | | | Cd63 | Malat1 | | B4galt6 | Ubb | Arhgap5 | | |
| | Hacd3 | Aifm3 | Saraf | | | | Atox1 | Rock2 | | Zbtb20 | Gm10288 | Magt1 | | |
| | Gm6863 | Utp14b | Ctsd | | | | Ifi202b | Ttc14 | | Ccdc171 | Ppib | Son | | |
| | Gm21399 | Peg3 | Gm5436 | | | | Cstb | Tgfbr1 | | Nexn | Dpp6 | Gbp7 | | |
| | Atp5e | 9330159F19Rik | Gm5865 | | | | Nfkbia | Ubn2 | | Colgalt2 | Oxct1 | Nupr1 | | |
| | Ctsd | Sfxn5 | S100a11 | | | | Rps26.ps1 | March1 | | Sparcl1 | Serpine2 | Nfat5 | | |
| | Gm12892 | Slc7a11 | Cyb5a | | | | Ctsz | Pnn | | Calr | Arl2bp | 9330159F19Rik | | |
| | Golim4 | Prpf4b | RP23.123D6.12 | | | | Ms4a6c | Rbm25 | | 4930480K23Rik | Gm3531 | Cyp39a1 | | |
| | B2m | Slitrk2 | Cd63.ps | | | | Gm12254 | Rpl35 | | Kdm2a | Gm11966 | Igfbp4 | | |
| | Jun | Gprasp1 | Rpl39 | | | | Rps10.ps2 | Ppia | | | Rtn1 | Ccdc104 | | |
| | Selenos | Ankrd11 | Ybx1.ps2 | | | | Npc2 | Pmepa1 | | | Gng11 | Gm6625 | | |
| | Gfap | Cxcl14 | Shfm1 | | | | Atp5e | Serinc3 | | | Rpl10a.ps1 | Snx29 | | |
| | Gm11966 | Gapdh | AU021092 | | | | Ifi44 | Rpl10 | | | Arpc2 | Tec | | |
| | Sept7 | Kcnk1 | Gm8276 | | | | Csf1 | Nav3 | | | Rps26.ps1 | Cmtm5 | | |
| | F3 | Ccdc50 | Gm13835 | | | | Gm15772 | Gnb2l1 | | | Agpat5 | Mboat2 | | |
| | Hacd2 | | Gm5644 | | | | Tnf | Rpl18a | | | Ndufs4 | Kdm5b | | |
| | Gnas | | Gm8730 | | | | Mia2 | Rpl17 | | | Gm5865 | Abca1 | | |
| | Cct2 | | Smim10l1 | | | | Gm15421 | Ptgs1 | | | Phlda1 | RP24.288C12.6 | | |
| | Rps20 | | Gm5778 | | | | Rpl9.ps6 | Ywhah | | | Rps24.ps3 | M6pr | | |
| | Rpl32 | | Gimap4 | | | | Plpp3 | Hmha1 | | | Cox4i1 | Nfe2l2 | | |
| | Gatm | | Gm6733 | | | | Rgs1 | Basp1 | | | Tmem179 | Zfhx3 | | |
| | Rpl8 | | Vim | | | | Calm1 | Srrm1 | | | Ahi1 | Sort1 | | |
| | Sbds | | Pdcd5.ps | | | | Rpl39 | | | | Fis1 | Wnt6 | | |
| | Skp1a | | Cfap36 | | | | Rpl22.ps1 | | | | Selenof | Tmem132b | | |
| | Msmo1 | | Rps3 | | | | Gm11966 | | | | Eif3i | Frmd4a | | |
| | Snx3 | | Tspan13 | | | | Gm7266 | | | | Ly6h | Phf17 | | |
| | Ybx1 | | Rpl18.ps2 | | | | Gm12183 | | | | Rplp1 | Entpd1 | | |
| | H3f3b | | Nsrp1 | | | | Cox4i1 | | | | Ndufa7 | Agt | | |
| | Eif3k | | Rps19.ps6 | | | | Ctsl | | | | Gm16580 | Cldn5 | | |
| | Sox4 | | Wfdc1 | | | | Gm9800 | | | | Gm15500 | Crip2 | | |
| | Hint1 | | Ifitm3 | | | | Cox6a1 | | | | Gm4204 | Plcd4 | | |
| | Calm1 | | Selenow | | | | Rps15 | | | | Omg | Lix1 | | |
| | Psma7 | | mt.Cytb | | | | Dbi | | | | Ednrb | Ctbp2 | | |
| | Fez1 | | Tcea1.ps1 | | | | Rps11.ps1 | | | | Gria2 | Cyp2j6 | | |
| | Rheb | | Higd2a | | | | Cxcl2 | | | | Rpl36al | Baz2b | | |
| | Nucks1 | | Ier2 | | | | Saraf | | | | Tspan13 | Socs2 | | |
| | Eef2 | | H2.K1 | | | | Rps19.ps6 | | | | Gm6368 | Atp8a1 | | |
| | Cbr1 | | Gm7266 | | | | Cox6b1 | | | | Cox7b | Cyp4v3 | | |
| | H2.D1 | | Selenos | | | | Hacd2 | | | | Il1rap | Snord49b | | |
| | Tcf12 | | Gm12481 | | | | Wfdc17 | | | | Commd6 | Stx5a | | |
| | Eef1a1 | | Gm15920 | | | | mt.Nd4 | | | | Gm14681 | Cep85l | | |
| | Matr3 | | Cox7a2 | | | | Capg | | | | Mpc2 | Nell2 | | |
| | Rps9 | | Gpx1 | | | | Prdx1 | | | | Atp5g3 | Fxyd1 | | |
| | Anapc11 | | Rps10.ps2 | | | | Pld3 | | | | Ndufa1 | Parp4 | | |
| | Rps14 | | mt.Nd1 | | | | Lgals3bp | | | | Cct7 | Zfp191 | | |
| | Gtf2i | | Cox4i1 | | | | Postn | | | | Nsg1 | 2310022B05Rik | | |
| | Bcan | | Actb | | | | Gm6136 | | | | Psmb1 | Bmp7 | | |
| | Rps4x | | Igf1r | | | | Gm4366 | | | | Cfl1 | Hspa8 | | |
| | Ubb | | Ostf1 | | | | Gm8730 | | | | Prdx2 | Foxo1 | | |
| | Hnrnpd | | Mia2 | | | | Grn | | | | Dbi | Eps15 | | |
| | Dync1li2 | | Fkbp1a | | | | Slc25a5 | | | | Sulf2 | Cdh8 | | |
| | Rps21 | | Eif3j2 | | | | Rplp0 | | | | Dtd1 | Paqr6 | | |
| | Rps15 | | Rps15.ps2 | | | | Gm4604 | | | | Cspg4 | Slc43a3 | | |
| | Tpm3.rs7 | | Ube2d3 | | | | Fcer1g | | | | Pcyt1b | Chd2 | | |
| | Rpl4 | | Arpc2 | | | | Cd9 | | | | Dscam | Fnbp1l | | |
| | Rplp1 | | Gm4604 | | | | Zfp638 | | | | Mlf2 | Notch2 | | |
| | Dstn | | Tonsl | | | | Cox8a | | | | Gm1673 | Sipa1l1 | | |
| | Sec11c | | Ramp2 | | | | Rps3 | | | | Nop10 | Tmtc2 | | |
| | Cnn3 | | Pabpc1 | | | | Pabpc1 | | | | Ugcrq | Lipg | | |
| | Nasp | | Gm14253 | | | | Eif1.ps1 | | | | Atxn10 | Prrg3 | | |
| | Ppp1cb | | Hint1 | | | | Gusb | | | | Ckb | Scd2 | | |
| | Vapa | | Id3 | | | | Cntrl | | | | A730017C20Rik | Megf10 | | |
| | Ilf3 | | Gm9840 | | | | Gm6265 | | | | Serpina3n | Hivep3 | | |
| | Pcbp2 | | Slc16a4 | | | | Ier3 | | | | Aplp1 | Slc25a13 | | |
| | Cct6a | | Rps15 | | | | Lgals1 | | | | Higd2a | Ncoa7 | | |
| | Ywhab | | Rpl9.ps6 | | | | Tmsb10 | | | | Cxcl14 | Synm | | |
| | Pcbp1 | | Pglyrp1 | | | | Gpx1 | | | | Rps9 | Leprel2 | | |
| | Ctss | | Gstp.ps | | | | Csf2ra | | | | Dner | RP23.32A8.1 | | |
| | Zhx1 | | BC002163 | | | | Gm43712 | | | | Pcbp2 | Prss56 | | |
| | Ywhae | | Tpm4 | | | | Hpf1 | | | | Olfm1 | Fam107a | | |
| | Arpc2 | | Epb41l2 | | | | Gm6368 | | | | Cryab | Tiam1 | | |
| | Rpl23 | | Naa38 | | | | Rps15.ps2 | | | | Kcnd2 | Gm4076 | | |
| | | | Gm12020 | | | | Rps9 | | | | Znhit6 | CT030684.1 | | |
| | | | Ndufa11 | | | | Anxa5 | | | | Gtf2h5 | Tmem47 | | |
| | | | Calm1 | | | | E230029C05Rik | | | | Ndufc2 | Cpne8 | | |
| | | | S100a16 | | | | Gm5644 | | | | Opcml | Ccdc173 | | |
| | | | Gm6542 | | | | Ugcrq | | | | 1810037I17Rik | Erbb3 | | |
| | | | AC149090.1 | | | | Rpl18.ps2 | | | | Uchl3 | Vwa1 | | |
| | | | Ssr2 | | | | Gm6472 | | | | Cdh13 | Cgnl1 | | |
| | | | Rpl37 | | | | Wapl | | | | Sfr1 | Tnfrsf19 | | |

| | | | | | | | | | | | | | | |
|---|---|---|---|---|---|---|---|---|---|---|---|---|---|---|
| | | | Gm13436 | | | | Rps19 | | | | Gm2a | Itga6 | | |
| | | | Gm10039 | | | | Nsrp1 | | | | Cp | Zfp609 | | |
| | | | Grrp1 | | | | Mir703 | | | | Tubb2a | mt.Rnr1 | | |
| | | | S100a13 | | | | Sh3bgrl3 | | | | 3632451O06Rik | Alx4 | | |
| | | | Tpm3 | | | | Rpl9.ps7 | | | | Gjc3 | Prrc2c | | |
| | | | Gm7407 | | | | Gm10080 | | | | Atp6v1g1 | | | |
| | | | Fam204a | | | | Rps11 | | | | Lnx1 | | | |
| | | | Myl12a | | | | Cotl1 | | | | Adam9 | | | |
| | | | Gm10031 | | | | Gm8606 | | | | Pea15a | | | |
| | | | Txn.ps1 | | | | Lyzl4 | | | | Sfxn1 | | | |
| | | | Atp5j | | | | H2.DMa | | | | Pfn1 | | | |
| | | | Psma5 | | | | Plau | | | | Uqcrc1 | | | |
| | | | Ccdc186 | | | | Akr1a1 | | | | Srp14 | | | |
| | | | Gm6136 | | | | Gm12020 | | | | Cdk4 | | | |
| | | | Ifi27l2a | | | | Cybb | | | | Itgb8 | | | |
| | | | Rplp2 | | | | Psme2b | | | | Deb1 | | | |
| | | | Dynlt1.ps1 | | | | Ssr4 | | | | Megf11 | | | |
| | | | Sf3b6 | | | | Cd72 | | | | Cox6c | | | |
| | | | Zfp638 | | | | Selenof | | | | Myo5a | | | |
| | | | Rps11.ps1 | | | | Gm15148 | | | | Pou3f1 | | | |
| | | | Wapl | | | | Zfas1 | | | | Tacc2 | | | |
| | | | Icam2 | | | | Ndufa2 | | | | Atp5j | | | |
| | | | Rac1 | | | | Gm9294 | | | | Gnb4 | | | |
| | | | Gm14513 | | | | Shfm1 | | | | Cadm2 | | | |
| | | | Bag1 | | | | Il4i1 | | | | Atp6v1a | | | |
| | | | Gm43712 | | | | Gm13436 | | | | Gria4 | | | |
| | | | Gm21399 | | | | Gm12481 | | | | Sept7 | | | |
| | | | Tmem252 | | | | Erbin | | | | Brinp1 | | | |
| | | | Rps15a | | | | Ppp1r15a | | | | Eif5a | | | |
| | | | P4hb | | | | Selenok | | | | S100a13 | | | |
| | | | Lgals1 | | | | Rpl22l1 | | | | Ndufb8 | | | |
| | | | Tmsb10 | | | | Eif3f | | | | Pgp | | | |
| | | | Pcbp1 | | | | Ifitm3 | | | | Psmb3 | | | |
| | | | Uqcrh.ps2 | | | | Rps21 | | | | Bcan | | | |
| | | | Gm11964 | | | | Zeb2os | | | | Gm10250 | | | |
| | | | H2.D1 | | | | Gm1966 | | | | Ptprn | | | |
| | | | RP23.269H21.1 | | | | Gm14513 | | | | Krtcap2 | | | |
| | | | Ilk | | | | Ctsh | | | | Atp5b | | | |
| | | | Cox6b1 | | | | Mt2 | | | | Sri | | | |
| | | | Lmo2 | | | | Glmp | | | | Cask | | | |
| | | | Rasip1 | | | | Gm10076 | | | | Tceb2 | | | |
| | | | Psme2b | | | | Pdcd5.ps | | | | Mt3 | | | |
| | | | Erbin | | | | Pfdn5 | | | | Ppp1r14c | | | |
| | | | Zmat2 | | | | Cxcl16 | | | | Park7 | | | |
| | | | Gm9294 | | | | Crip1 | | | | C4b | | | |
| | | | Gata2 | | | | Gm6394 | | | | Sox6 | | | |
| | | | Oaz1.ps | | | | Cspg4 | | | | Pcdh9 | | | |
| | | | Anp32b.ps1 | | | | Ier2 | | | | Uqcrb | | | |
| | | | Atox1 | | | | Myeov2 | | | | Rgs20 | | | |
| | | | Rras | | | | Cd68 | | | | Hint1 | | | |
| | | | Rhoj | | | | Gm8692 | | | | Ugp2 | | | |
| | | | Ly6a | | | | RP24.389J11.1 | | | | Mdh2 | | | |
| | | | Mydgf | | | | Gm4204 | | | | 6330403K07Rik | | | |
| | | | Rps19 | | | | Gm3531 | | | | Ndufab1 | | | |
| | | | Uqcr11 | | | | Gm14539 | | | | Mt1 | | | |
| | | | Esam | | | | Lgals3 | | | | Atp6v0e | | | |
| | | | Rps20 | | | | Nfkbiz | | | | Rpl32 | | | |
| | | | Gm8129 | | | | Gm8292 | | | | Pomp | | | |
| | | | Gm8566 | | | | Gm11361 | | | | Rps3 | | | |
| | | | Psmb2 | | | | Snhg8 | | | | Rpl39 | | | |
| | | | Cldn5 | | | | Ifi207 | | | | Psma7 | | | |
| | | | Bvht | | | | Rps14 | | | | Isoc1 | | | |
| | | | Snhg8 | | | | Gm12696 | | | | Zcchc24 | | | |
| | | | Gm12231 | | | | Ybx1.ps2 | | | | Bzw1 | | | |
| | | | Junb | | | | 2010107E04Rik | | | | Cdc42 | | | |
| | | | Hpf1 | | | | Diaph2 | | | | Cox6a1 | | | |
| | | | Chchd2 | | | | Mydgf | | | | Lrrtm3 | | | |
| | | | Ndufa8 | | | | Bcl2a1b | | | | Pafah1b1 | | | |
| | | | Pea15a | | | | Mpeg1 | | | | Phyhipl | | | |
| | | | Alas1 | | | | Cox7a2 | | | | Ptpre | | | |
| | | | Anxa2 | | | | Rpl8 | | | | Ran | | | |
| | | | Hspd1.ps3 | | | | Spcs2.ps | | | | Gm10260 | | | |
| | | | Csnk2b | | | | Cox6c | | | | Cox7a2 | | | |
| | | | Suclg1 | | | | Gadd45b | | | | Wbp5 | | | |
| | | | Gm16399 | | | | Hspd1.ps3 | | | | Acat1 | | | |
| | | | Id1 | | | | Atp6v1f | | | | Cd9 | | | |
| | | | Ost4 | | | | Rab1a | | | | Rps14 | | | |
| | | | Eea1 | | | | Ccl9 | | | | Atpif1 | | | |
| | | | Gabarapl1 | | | | RP23.123D6.12 | | | | Atp5a1 | | | |
| | | | Dpm3 | | | | Arpc1b | | | | Tmem176a | | | |
| | | | Spcs2.ps | | | | Tspo | | | | mt.Cytb | | | |
| | | | Gng5 | | | | Txn1 | | | | Phactr3 | | | |
| | | | Txndc17 | | | | Rpl39.ps | | | | Atp5e | | | |
| | | | Sec61b | | | | Ndufa1 | | | | Sdhb | | | |
| | | | Gm6394 | | | | Psmb8 | | | | Ndufa2 | | | |
| | | | Atp5o | | | | Eef2 | | | | Rps20 | | | |
| | | | Rbx1 | | | | Gm15920 | | | | Ywhab | | | |
| | | | Snx3 | | | | Ifi211 | | | | mt.Nd1 | | | |
| | | | Tcaf1 | | | | Cd14 | | | | Gnas | | | |
| | | | Cstb | | | | Hcar2 | | | | Tpm1 | | | |
| | | | BC028528 | | | | H2.T23 | | | | Atp5c1 | | | |
| | | | Gm2000 | | | | Bcl2a1a | | | | Fermt2 | | | |
| | | | Gm9392 | | | | Mif | | | | | | | |
| | | | Mrps5 | | | | | | | | | | | |
| | | | Uri1 | | | | | | | | | | | |
| | | | Gm8692 | | | | | | | | | | | |
| | | | Rpl7.ps7 | | | | | | | | | | | |
| | | | Dynll1 | | | | | | | | | | | |
| | | | Snrpb | | | | | | | | | | | |
| | | | Gm8606 | | | | | | | | | | | |
| | | | Srgn | | | | | | | | | | | |
| | | | Lrp8 | | | | | | | | | | | |
| | | | Gm5881 | | | | | | | | | | | |
| | | | Col4a2 | | | | | | | | | | | |
| | | | Anapc11 | | | | | | | | | | | |
| | | | Gm11361 | | | | | | | | | | | |
| | | | RP24.389J11.1 | | | | | | | | | | | |
| | | | Cox8a | | | | | | | | | | | |
| | | | Myh10 | | | | | | | | | | | |
| | | | Gm12966 | | | | | | | | | | | |

**Table S4.** Characteristics of myeloid clusters identified in DROP-seq data. Top 4 gene ontology (GO) terms were listed. Marker genes for each cluster were defined as differentially expressed genes between clusters at threshold: FDR <=0.01 and lo$_{g2}$FC>=0.05
Genes are listed from the highest to lowest log$_2$FC

| Cluster number | CL0 | CL1 | CL2 | CL3 | CL4 | CL5 | CL6 | CL7 | CL8 |
|---|---|---|---|---|---|---|---|---|---|
| Ontogeny | Ho-Mg | Ho-Mg | Mg-TAMs | Mg-TAMs | Mg-TAMs astrocytic-like | Mg-TAMs transcriptionally active | Mg-TAMs EC-like | Mo-TAMs | BAMs |
| Top 4 GO terms | GO:0071277 cellular response to calcium ion | GO:0008380 RNA splicing | GO:0055094 response to lipoprotein particle | GO:0030593 neutrophil chemotaxis | GO:0042063 gliogenesis | GO:0042273 ribosomal large subunit biogenesis | GO:0001944 vasculature development | GO:0031347 regulation of defense response | GO:0006954 inflammatory response |
| | GO:0070997 neuron death | GO:0034110 regulation of homotypic cell-cell adhesion | GO:0050866 negative regulation of cell activation | GO:0006954 inflammatory response | GO:0034330 cell junction organization | GO:1904667 negative regulation of ubiquitin protein ligase activity | GO:2000147 positive regulation of cell motility | GO:0009615 response to virus | GO:0032103 positive regulation of response to external stimulus |
| | GO:0002521 leukocyte differentiation | GO:0010563 negative regulation of phosphorus metabolic process | GO:0097529 myeloid leukocyte migration | GO:0055094 response to lipoprotein particle | GO:0120035 regulation of plasma membrane bounded cell projection organization | GO:2000343 positive regulation of chemokine (C-X-C motif) ligand 2 production | GO:0032990 cell part morphogenesis | GO:0034341 response to interferon-gamma | GO:1904646 cellular response to amyloid-beta |
| | GO:0150146 cell junction disassembly | GO:0071824 protein-DNA complex subunit organization | GO:0032102 negative regulation of response to external stimulus | GO:0032760 positive regulation of tumor necrosis factor production | GO:0007610 behavior | GO:0000470 maturation of LSU-rRNA | GO:0034330 cell junction organization | GO:0019221 cytokine-mediated signaling pathway | GO:0043410 positive regulation of MAPK cascade |
| Marker genes | Jun | Sfrs18 | Cxcl13 | Cxcl13 | Sparcl1 | Rps12 | Ly6c1 | Ly6c2 | Mrc1 |
| | Egr1 | Frmd4a | Cst7 | Ccl4 | Clu | Rps27a | Cldn5 | Plac8 | F13a1 |
| | P2ry12 | Malat1 | Spp1 | Cst7 | Slc1a2 | Rps29 | Itm2a | H2.Eb1 | Dab2 |
| | Tmem119 | Pnn | Cd63.ps | Cd63.ps | Ptn | Rps27 | Gkn3 | AA467197 | Pf4 |
| | Fos | Prpf4b | Ccl6 | Spp1 | Ttr | Rpl17 | Abcb1a | Lyz2 | Ms4a7 |
| | Hspa1a | Akap9 | Cd63 | Ccl3 | Ttyh1 | Rpl23a | Igfbp7 | Il1b | Cd163 |
| | Ier5 | Jmjd1c | Lpl | Gm11942 | Mt2 | Gnb2l1 | Flt1 | Thbs1 | Wfdc17 |
| | Gpr34 | Dock10 | Tyrobp | Ccl12 | Atp1a2 | Rps28 | Ptprb | Tgfbi | Cp |
| | Rhob | Atrx | Apoe | Tyrobp | Plp1 | Rps23 | Spock2 | Arg1 | Ccl8 |
| | Zfp36 | Ssh2 | Creg1 | Ctsd | Aldoc | Rpl21 | Bsg | Ifitm3 | Wwp1 |
| | Btg2 | Smap2 | Rpl32 | Gm14303 | Cpe | Rpl11 | Adgrf5 | Ms4a4c | Clec10a |
| | Jund | Ythdc1 | Cd52 | Cd63 | Plpp3 | Fau | Slco1a4 | Ly6a | Cbr2 |
| | Selplg | Blnk | Cd9 | Cd52 | Scg3 | Rpl13a | Slco1c1 | Lgals3 | Ccl7 |
| | Cx3cr1 | Cx3cr1 | Rps5 | Ctsz | Gfap | Rpl18a | Cxcl12 | H2.Ab1 | Maf |
| | Siglech | Rdx | mt.Nd1 | Rps25.ps1 | Ptprz1 | Rpl12 | Pltp | Ccr2 | Stab1 |
| | Junb | Fnbp4 | Ctsb | Apoc1 | Gja1 | Rps6 | Ctla2a | Ly6i | Lyve1 |
| | Ptgs1 | Myo9a | Ctsd | Ctsl | Fabp7 | Rps17 | Ly6a | AW112010 | Gas6 |
| | Vsir | Golgb1 | Npc2 | Rpl37rt | Apod | Epb4.1l2 | Sptbn1 | Crip1 | Ccl24 |
| | Cst3 | Il6ra | Ctsl | Gm4149 | Fxyd1 | Sepp1 | Slc2a1 | S100a11 | Ccl2 |
| | Abca9 | Smarca2 | Gnas | Ch25h | Mt1 | Rpl10 | Pcp4l1 | Cxcl9 | Selenop |
| | P2ry13 | Nav3 | Tmsb4x | Cd9 | Dbi | Rpl9 | Gng11 | Srgn | Mgl2 |
| | Olfml3 | Ncor1 | mt.Cytb | Ctsb | Slc4a4 | Rps13 | Ablim1 | Cxcl2 | Itsn1 |
| | Txnip | Ivns1abp | Gm6977 | Clec7a | Slc1a3 | Rpl19 | Adgrl4 | Fxyd5 | Cmah |
| | Nav3 | Ogfrl1 | Trem2 | Grn | Gpm6b | D4Wsu53e | Egfl7 | Cdkn1a | Fcgr2b |
| | Ltc4s | Tcf4 | | Syngr1 | Cspg5 | RP24.288C12.6 | Esam | Mndal | Cd209a |
| | Tgfbr1 | Rsrc2 | | Gm15421 | Aqp4 | Rpl5 | Sdpr | Ifi204 | Pltp |
| | Srgap2 | Pip4k2a | | Gm10269 | Ednrb | Rpl31 | Tm4sf1 | Isg15 | Folr2 |
| | Ivns1abp | Nipbl | | Rps16.ps2 | Mt3 | Ptplb | Kitl | Plbd1 | Igfbp4 |
| | Ubc | Phf14 | | Gm9794 | Cald1 | Rpl3 | Id3 | Gbp2 | Stard8 |
| | Golm1 | Wnk1 | | Gm10076 | Prnp | AF251705 | Tsc22d1 | Slfn4 | Igf1 |
| | Il6ra | Dnajc7 | | B2m | Enpp2 | Rpl36a | Slc39a10 | Pim1 | Ifi203 |
| | Ccr5 | Bptf | | C1qc | Acsl3 | 4632428N05Rik | Ramp2 | H2.Aa | Gpx3 |
| | Hpgds | Gtf2h2 | | Gm10288 | Ndrg2 | Cep110 | Car4 | Rsad2 | Ehd4 |
| | Elmo1 | Acin1 | | Glmp | Atp1b2 | Rpl27 | Ccdc141 | Cfp | Vcam1 |
| | Pde3b | Ubash3b | | Gm6977 | Syt11 | Lilrb4 | Mal | Tnfaip2 | Iqgap2 |
| | Nrip1 | Git2 | | Gm15427 | Gria2 | Rps7 | 9430020K01Rik | Ccl5 | Pla2g2d |
| | Slco2b1 | Bod1l | | Gm6030 | Gpr37l1 | Sfrs18 | Palmd | S100a6 | Pla2g7 |
| | Crybb1 | Rps6ka1 | | Rpl36a.ps2 | Scd2 | Ddx26b | Pecam1 | Clec4n | Clec4a3 |
| | Hpgd | Arglu1 | | Cd300c2 | Luzp2 | Rpl35 | Slc16a1 | Ifi209 | Blvrb |
| | Zfhx3 | Tardbp | | Rps26 | Bcan | Rpl7a | Golim4 | Ifi203 | Cd36 |
| | Lpcat2 | Ddx17 | | Ccl2 | Ntrk2 | Apoc1 | Slc38a5 | Msrb1 | Cd209b |
| | Csmd3 | Pnisr | | Fcer1g | Htra1 | Ctage5 | Ppfibp1 | Slfn2 | Sdc4 |
| | Tanc2 | Gcnt1 | | Lgals3bp | Dclk1 | Tmem66 | Fam13c | Vegfa | Myo5a |
| | Mertk | | | Gm14586 | Asrgl1 | Ppia | Sgms1 | Vim | Nrp1 |
| | Rnase4 | | | Rpl32 | Gstm1 | Rps25 | Cd93 | Il1rn | Cfp |
| | Serinc3 | | | Pld3 | Nnat | Erbb2ip | Edn1 | Ifi211 | Snx6 |
| | Csf1r | | | Tmsb4x | S100b | Gm26924 | Sema3c | Cebpb | Hpgd |
| | Arhgap5 | | | Rps24.ps3 | Nrxn1 | Postn | Ets1 | Ms4a7 | Lgals1 |
| | Maf | | | Cd68 | Id3 | Rpl13 | Utrn | Cd14 | Fcna |
| | Mef2a | | | Gm9843 | Lsamp | 2700029M09Rik | C130074G19Rik | Ptgs2 | Ifi207 |
| | Lgmn | | | C1qa | Rgs5 | Ccdc55 | Ptn | Iqgap1 | Snx2 |
| | Mef2c | | | Rpl3.ps1 | Tuba1a | Tpt1 | Cd59a | Mxd1 | Clec4n |
| | Srrm2 | | | Gm10073 | Timp3 | Rps18 | Epas1 | S100a4 | Fosb |
| | Itgam | | | Rpl39 | Mgp | Spp1 | Col4a1 | Prdx5 | Ms4a6b |
| | Son | | | Ier3 | Gpm6a | Rpl30 | Podxl | Clec4e | Ctsc |
| | Marcks | | | Gm11478 | Igfbp5 | Rps8 | Sox17 | Samhd1 | Dse |
| | Malat1 | | | Gm5805 | Gabrb1 | Rps16 | Fermt2 | Txn1 | Apod |
| | Apbb1ip | | | Rpl10a.ps1 | Sox9 | Rps2 | Slc6a6 | S100a10 | Ifitm3 |
| | Hexb | | | Gm15772 | Car2 | BC016423 | Crip2 | Ifi205 | Mndal |
| | | | | Rps13.ps2 | Cadm2 | Diap2 | Erg | Ifi27l2a | Fcrls |
| | | | | Gm4332 | Prdx6 | Cd74 | Itih5 | Ms4a6c | Ifitm2 |
| | | | | Calr.ps | Ncam1 | Lrrc58 | Pglyrp1 | Fosl2 | Lyz2 |
| | | | | Rps19 | Igfbp2 | Rpl7 | Arhgap29 | Slfn5 | Fos |
| | | | | Ccl6 | Pdgfra | Zfml | Nes | Cytip | Lilra5 |
| | | | | Rack1 | Tmem47 | Rpl37a | Cp | Tmsb10 | Fcgrt |
| | | | | Ssr4 | Olig1 | A430104N18Rik | Ptprg | Ms4a6b | |
| | | | | H2.D1 | S1pr1 | 0610031J06Rik | App | Cxcl10 | |
| | | | | Itgb2 | Ntsr2 | Rpl27a | Foxq1 | Ahnak | |
| | | | | Cox6a2 | Itih3 | 1500012F01Rik | Id1 | Calm1 | |
| | | | | C1qb | Ckb | Vimp | Kdr | Cd74 | |
| | | | | Gm7266 | Nedd4 | 4933426M11Rik | Vwa1 | Msr1 | |
| | | | | Rpl31.ps8 | Atp1b1 | Cwc22 | Rflnb | Nr4a2 | |
| | | | | Ndufa1 | Fam213a | D17Wsu104e | Fn1 | Psmb8 | |
| | | | | Rpl22l1 | Vim | Rpl15 | Ltbp4 | Il2rg | |
| | | | | Gm5963 | Fermt2 | Rpl10a | Tek | Cybb | |
| | | | | Hexa | Kcnj10 | Fam105b | Selenom | Ifitm2 | |
| | | | | Rpl22.ps1 | S100a1 | Wapal | Arl4a | Ifi202b | |
| | | | | Npc2 | Acsl6 | Rab1 | Abcg2 | Cstb | |
| | | | | Lpl | Cryab | Snord49b | Maoa | Lilrb4a | |
| | | | | Rps26.ps1 | Tspan7 | Gm13476 | Grrp1 | Ifit2 | |
| | | | | Gm9385 | Id4 | Sept15 | Ece1 | Irf7 | |
| | | | | Rpl41 | Sox2 | Selk | Timp3 | Clec7a | |
| | | | | Rpl14.ps1 | Tpm1 | Erdr1 | Stmn2 | Lgals1 | |
| | | | | Gnas | Scrg1 | Ccl6 | Ptprr | Fgl2 | |
| | | | | Ifi27l2a | Tril | Asph | Slc22a8 | Emb | |
| | | | | Gm11361 | S100a6 | Lpl | Apod | Fth1 | |
| | | | | Gm10260 | Agt | B630005N14Rik | Col4a2 | Cd44 | |
| | | | | Gm6204 | Zbtb20 | RP24.312B12.1 | Ptrf | Ifi207 | |
| | | | | Rpl39.ps | Cnn3 | A630007B06Rik | Lef1 | Iigp1 | |
| | | | | Ank | Meg3 | Rpl38 | Slc16a2 | Ccr1 | |
| | | | | Ctsh | S100a16 | Hnrnpa3 | Uaca | Gm6977 | |
| | | | | Rnaset2a | Fam107a | Rps4x | AU021092 | Samd9l | |
| | | | | Cd83 | Pou3f3 | Rpsa | Rgs5 | Nr4a3 | |
| | | | | Rps15a | Cldn10 | Ptms | Nostrin | Slamf7 | |
| | | | | Rps15a.ps5 | Pla2g7 | Ccdc41 | Akap12 | Bst2 | |
| | | | | Lyzl4 | Slco1c1 | Gm26339 | Slc9a3r2 | H2.Q7 | |
| | | | | Rpl38.ps2 | Map2 | Snord42a | Dynll1 | Bcl2a1b | |
| | | | | Rps20 | Igfbp7 | Clcn4.2 | Aplp2 | Txn.ps1 | |
| | | | | Gm5905 | Ntm | Rplp2 | Slc7a1 | Sp140 | |
| | | | | Gm6472 | Cacng4 | Jhdm1d | Ushbp1 | Nampt | |
| | | | | Selenof | Gm42418 | Rpl37 | Ifitm3 | F10 | |
| | | | | Gusb | Hepacam | Siglec5 | Paqr5 | Ifrd1 | |
| | | | | Gm2000 | Ncan | Rpl18 | Apold1 | Gbp3 | |
| | | | | Gm8730 | Gm12222 | Smap2 | Edn3 | Tagln2 | |
| | | | | | Mlc1 | Tnfsf8 | Tspan13 | Fcgr2b | |
| | | | | | Serpina3n | 2700089E24Rik | Hspb1 | Amica1 | |
| | | | | | Chchd10 | D14Abb1e | Tpm1 | Tpt1.ps3 | |
| | | | | | Mfge8 | Suv420h1 | Ifitm2 | Emp3 | |
| | | | | | Mmd2 | Abcd2 | Vcam1 | Tiparp | |
| | | | | | F3 | Ing4 | Cgnl1 | Pla2g7 | |
| | | | | | Ddah1 | 1110001A16Rik | Adcy4 | Clec12a | |
| | | | | | Syne1 | Leprot | Eng | Gda | |
| | | | | | Limch1 | Ccdc104 | Anxa2 | Ifi30 | |
| | | | | | Ldhb | Slc11a1 | Mpzl1 | Cyp4f18 | |
| | | | | | Slc6a1 | Rps24 | Ebf1 | Rpl34 | |
| | | | | | Tpm2 | Myo1e | Ctnnb1 | Slfn1 | |
| | | | | | Lhfpl3 | D15Ertd621e | Crip1 | Gm7676 | |
| | | | | | Nrcam | Apoe | Wfdc1 | Zbp1 | |
| | | | | | Fut9 | Tec | Heg1 | C3 | |
| | | | | | Nrxn2 | Niacr1 | Tfrc | Emilin2 | |
| | | | | | Kif1b | Colec12 | Col4a3bp | Phf11d | |
| | | | | | Dmd | Plgrkt | Clec14a | Lst1 | |
| | | | | | Aplp1 | Rpl36 | Tceb2 | Cxcl16 | |
| | | | | | Sdc4 | 2610305D13Rik | Cd200 | Crem | |
| | | | | | Myo6 | D19Bwg1357e | Ndrg1 | Rps2 | |
| | | | | | Rgcc | Cox7c | Nedd4 | Shfm1 | |
| | | | | | Tspan3 | Rin2 | Sema7a | Tgm2 | |
| | | | | | Gm5514 | 2510009E07Rik | Scgb3a1 | Stat1 | |
| | | | | | Prex2 | Phyhd1 | Net1 | Phf11b | |
| | | | | | Fez1 | Gtl3 | Actn4 | Lsp1 | |
| | | | | | Slc38a3 | Arl6ip5 | Luzp1 | Klra2 | |
| | | | | | Dtna | Sc4mol | Cyyr1 | Myof | |
| | | | | | Fjx1 | Apobec1 | Fnbp1l | H2.K1 | |
| | | | | | Chpt1 | Sfi1 | Prnp | Dab2 | |
| | | | | | Sash1 | Creg1 | Tshz2 | Acp5 | |
| | | | | | Map1b | Il4i1 | Tmsb10 | Anxa1 | |
| | | | | | Hmgcs1 | Uba52 | Car2 | Psme2b | |
| | | | | | Nap1l5 | Hmgb1 | Bmx | Sp100 | |
| | | | | | Dner | Cd84 | Plcb1 | AI662270 | |
| | | | | | Cbx5 | Tial1 | Igfbp4 | Gpnmb | |

| | | | | | | | | | |
|---|---|---|---|---|---|---|---|---|---|
| | | | | | Slc6a11 | Mnda | BC002163 | Wfdc17 | |
| | | | | | 2900052N01Rik | Cfh | Prex2 | Mmp14 | |
| | | | | | sept.07 | Ftl1 | Sparcl1 | Ccrl2 | |
| | | | | | mt.Rnr1 | Calr | S100a13 | Itga4 | |
| | | | | | Gpld1 | 4632419I22Rik | Igf1r | Nfil3 | |
| | | | | | Vcan | Rpl23 | Afap1l1 | Sh3bgrl | |
| | | | | | Epn2 | Dnajb14 | Afdn | Pde4b | |
| | | | | | Tnik | Ccdc66 | Kank3 | H2.DMa | |
| | | | | | RP23.4P23.1 | Gm10263 | Foxc1 | Hspa8 | |
| | | | | | Gnao1 | Rpl6 | Gm3531 | Osm | |
| | | | | | Gng11 | Gapdh | Fzd6 | Runx3 | |
| | | | | | Klf9 | Mpeg1 | Snx3 | Epsti1 | |
| | | | | | Sptbn1 | Cd63 | Hip1 | Gm12854 | |
| | | | | | Itgb8 | 5830428H23Rik | Gm5436 | Anxa2 | |
| | | | | | Glud1 | Myl6 | Wwtr1 | H2.T23 | |
| | | | | | Psip1 | 2810055G20Rik | Uqcc2 | Anxa5 | |
| | | | | | Hbegf | D19Ertd737e | Hes1 | Stk17b | |
| | | | | | Agpat5 | Cyth4 | Lsr | Taldo1 | |
| | | | | | Rarres2 | Cnpy2 | Nos3 | Gbp4 | |
| | | | | | Slc25a4 | Ptplad2 | Ap2m1 | Clec4d | |
| | | | | | Kif21a | RP23.32A8.1 | Ctnna1 | Sh3bgrl3 | |
| | | | | | Tubb2b | St8sia6 | Prom1 | Mgst1 | |
| | | | | | Ezr | Slc15a3 | Plcb4 | Psmb9 | |
| | | | | | Col16a1 | Tmem179b | Rpl38.ps2 | Gbp5 | |
| | | | | | C4b | 4922501C03Rik | Rgs12 | Tspo | |
| | | | | | Pcsk1n | Gm11206 | Tmem252 | Bcl2a1d | |
| | | | | | Acsbg1 | Galnt7 | Wbp5 | AB124611 | |
| | | | | | Gstm5 | Ccdc50 | Atox1 | Ass1 | |
| | | | | | Sdc2 | Akap8l | Gm7809 | Rpl35a | |
| | | | | | Gap43 | Ctss | Serpinb6a | Btg1 | |
| | | | | | Gjb6 | mt.Tm | Arhgef12 | Ms4a6d | |
| | | | | | Pcdh10 | Abcg1 | Dab2ip | Bhlhe40 | |
| | | | | | Cyp2j9 | Ptplad1 | Calm1 | Txnrd1 | |
| | | | | | Skp1a | Agpat6 | Mfsd2a | Pygl | |
| | | | | | Fgfr3 | Hiat1 | Myl12b | Oasl1 | |
| | | | | | Arhgef12 | Aim2 | Fgd5 | Ifit3 | |
| | | | | | Nfia | Smad4 | Mt2 | Rnf149 | |
| | | | | | Bex3 | Apbb1ip | Vat1 | Mcl1 | |
| | | | | | Ccnd2 | Hspa4 | Itga1 | Lyz1 | |
| | | | | | Msi2 | Rp2h | Ttyh2 | Tpm4 | |
| | | | | | Add3 | Fmn1 | Lama4 | H2.DMb1 | |
| | | | | | Gm9625 | Rpl24 | Serpinb6b | Parp14 | |
| | | | | | Fam184a | Cacna1a | Alas1 | Gm6377 | |
| | | | | | Myo10 | Ank | Icam2 | Fabp5 | |
| | | | | | Pcdh17 | Pros1 | Myl12a | Alox5ap | |
| | | | | | Tcf12 | Naalad2 | Dynlt3 | Ifih1 | |
| | | | | | Fam171b | Brwd1 | Osbpl1a | Mcemp1 | |
| | | | | | Rcn2 | AI607873 | Fkbp1a | Oasl2 | |
| | | | | | Pcdh9 | Ptprc | Thra | Trim30a | |
| | | | | | Tmod2 | Usp12 | Iqgap1 | Rps27l | |
| | | | | | Msmo1 | Cldn25 | Cript | Ldha | |
| | | | | | Pcdh7 | Rpl14 | Tmem256 | Lrrfip1 | |
| | | | | | Mapt | Morf4l1 | Ahnak | Pirb | |
| | | | | | Plcd4 | Actg1 | Tonsl | Dusp5 | |
| | | | | | Cdh2 | Rpl28 | Dkk2 | Malt1 | |
| | | | | | Phyhipl | Myo5a | Eci1 | Gpr35 | |
| | | | | | Gm12346 | Atp2b1 | Gm4617 | Rps18 | |
| | | | | | Gm3764 | Selt | Sema3g | Ctsc | |
| | | | | | Mdk | Cd9 | Limch1 | Cd274 | |
| | | | | | 2900011O08Rik | Csf2ra | Pir | Prdx6 | |
| | | | | | Dlgap1 | Zbed6 | Lrp8 | Tpi1 | |
| | | | | | Ccdc88a | Osbpl8 | Sorbs2 | Gadd45b | |
| | | | | | Phactr3 | Prdm2 | Tes | Pfn1 | |
| | | | | | Chl1 | Naa35 | St6galnac2 | Mif | |
| | | | | | Gm12892 | Gm17087 | Cald1 | Nfkbie | |
| | | | | | 4930402H24Rik | Col27a1 | Slc30a1 | Csrnp1 | |
| | | | | | Dkk3 | Fgd3 | Mecom | B2m | |
| | | | | | Nkain4 | Tgfbr1 | Gm8894 | Sod2 | |
| | | | | | Serpine2 | Fam134b | Arpc1a | Actr3 | |
| | | | | | Cyp2j6 | Nptn | Dnajc10 | Myl12a | |
| | | | | | Kcnj16 | Ramp1 | Esyt2 | Psma7 | |
| | | | | | Rev3l | Acer3 | Nfib | Gbp7 | |
| | | | | | Hmgn5 | C230081A13Rik | Clec2d | Bcl2a1a | |
| | | | | | Cfap36 | Pdia3 | Bcl2l1 | RP23.354J5.3 | |
| | | | | | Phlda1 | Tm9sf3 | Ddit4 | Pkm | |
| | | | | | Pantr1 | Plxdc2 | Ccser2 | Fcgr4 | |
| | | | | | Npas3 | Zfp949 | Tinagl1 | Neurl3 | |
| | | | | | Cnp | Timm21 | S100a16 | Igsf6 | |
| | | | | | Cxcl14 | Golga4 | Aes | Plaur | |
| | | | | | Atp5g3 | Gm21092 | Fam63a | Psme2 | |
| | | | | | Syne2 | 5430435G22Rik | Ctdnep1 | Sdcbp | |
| | | | | | Ctnnd2 | Cxcl14 | Syne1 | Rps24.ps3 | |
| | | | | | Pmm1 | Arrdc4 | Gm8186 | Ets2 | |
| | | | | | Pea15a | Tmem57 | Grb14 | Arhgap15 | |
| | | | | | Cmtm5 | Ptpra | Hsp25.ps1 | Prdx1 | |
| | | | | | | Pgrmc2 | Ifnar1 | Gm15427 | |
| | | | | | | 1200014J11Rik | Zic3 | Gm9794 | |
| | | | | | | Tmed3 | Gm11223 | Rps13.ps2 | |
| | | | | | | Cux1 | Sec61b | Abracl | |
| | | | | | | Zmiz1 | Arhgap31 | Wdr89 | |
| | | | | | | Nr2c2 | Igf1 | Cd52 | |
| | | | | | | Eml4 | Clic5 | Psma2 | |
| | | | | | | Nsa2 | Robo4 | Cfb | |
| | | | | | | ZEB2-AS1-2 | Polk | Slc16a3 | |
| | | | | | | Nufip2 | Usp46 | Gbp8 | |
| | | | | | | | Rabac1 | H2.T22 | |
| | | | | | | | Use1 | Ptprc | |
| | | | | | | | Plscr2 | Chmp4b | |
| | | | | | | | Kdelr1 | Plin2 | |
| | | | | | | | Capns1 | Coro2a | |
| | | | | | | | Sntb2 | C130026I21Rik | |
| | | | | | | | Rnpepl1 | Cyba | |
| | | | | | | | Bola3 | Oas1a | |
| | | | | | | | Rell1 | Spty2d1 | |
| | | | | | | | Map1b | Rpl22 | |
| | | | | | | | Cfdp1 | Rps15a | |
| | | | | | | | Atp5j | H3f3b | |
| | | | | | | | Myo1b | Rel | |
| | | | | | | | Myh10 | Rbms1 | |
| | | | | | | | Pinx1 | Klf4 | |
| | | | | | | | Ndufa3 | Rps3 | |
| | | | | | | | Agfg1 | Gm15753 | |
| | | | | | | | Vim | Nr4a1 | |
| | | | | | | | Gm11942 | H2.D1 | |
| | | | | | | | | Clic4 | |
| | | | | | | | | Diaph1 | |
| | | | | | | | | Atp11b | |
| | | | | | | | | Rps10.ps2 | |
| | | | | | | | | Cd47 | |
| | | | | | | | | Rnf213 | |
| | | | | | | | | Gm4332 | |
| | | | | | | | | Cd300lf | |
| | | | | | | | | Cdk2ap2 | |
| | | | | | | | | Ccnd2 | |
| | | | | | | | | Rtp4 | |
| | | | | | | | | Metrnl | |
| | | | | | | | | Snx10 | |
| | | | | | | | | Arpc5 | |
| | | | | | | | | Cox17 | |
| | | | | | | | | Gm9843 | |
| | | | | | | | | Ly6e | |
| | | | | | | | | Il18bp | |
| | | | | | | | | Nfkb2 | |
| | | | | | | | | Gm1966 | |
| | | | | | | | | Fosb | |
| | | | | | | | | Capg | |
| | | | | | | | | Rps9 | |
| | | | | | | | | Gm5905 | |
| | | | | | | | | Gm6472 | |
| | | | | | | | | Vasp | |
| | | | | | | | | Rps16.ps2 | |
| | | | | | | | | Tuba1c | |
| | | | | | | | | Lcp1 | |
| | | | | | | | | Rplp0 | |
| | | | | | | | | Psmb10 | |
| | | | | | | | | Ezr | |
| | | | | | | | | Irf1 | |
| | | | | | | | | Rpl29 | |
| | | | | | | | | Ell2 | |
| | | | | | | | | Psap | |

| | | | | | | | | | |
|---|---|---|---|---|---|---|---|---|---|
| | | | | | | | | Rbpsuh.rs3 | |
| | | | | | | | | Rpl39 | |
| | | | | | | | | Gm9385 | |
| | | | | | | | | Sec61b | |
| | | | | | | | | Gm12254 | |
| | | | | | | | | Trim25 | |
| | | | | | | | | H2.Q6 | |
| | | | | | | | | Stat2 | |
| | | | | | | | | Nfe2l2 | |
| | | | | | | | | Nfkbia | |
| | | | | | | | | Rps15a.ps5 | |
| | | | | | | | | Slk | |
| | | | | | | | | Gm8995 | |
| | | | | | | | | Gm9835 | |
| | | | | | | | | Myl12b | |
| | | | | | | | | Ccl2 | |
| | | | | | | | | Cast | |
| | | | | | | | | Gpr65 | |
| | | | | | | | | Gpx1 | |
| | | | | | | | | Clec4a3 | |
| | | | | | | | | Gm6030 | |
| | | | | | | | | Gm10169 | |
| | | | | | | | | Aldoa | |
| | | | | | | | | Rps15a.ps7 | |
| | | | | | | | | Cnn2 | |
| | | | | | | | | Neat1 | |
| | | | | | | | | Rpl36al | |
| | | | | | | | | Gm5963 | |
| | | | | | | | | Gm15500 | |
| | | | | | | | | Rpl18.ps1 | |
| | | | | | | | | H2.Q4 | |
| | | | | | | | | Rps24.ps2 | |
| | | | | | | | | Sub1 | |
| | | | | | | | | Gm8822 | |
| | | | | | | | | Tmem123 | |
| | | | | | | | | Esyt1 | |
| | | | | | | | | Tpt1 | |
| | | | | | | | | Arpc2 | |
| | | | | | | | | Rps20 | |
| | | | | | | | | 1810037I17Rik | |
| | | | | | | | | Glrx | |
| | | | | | | | | Prelid1 | |
| | | | | | | | | Sp110 | |
| | | | | | | | | Syngr2 | |
| | | | | | | | | Rps14 | |
| | | | | | | | | Xaf1 | |
| | | | | | | | | Gm4204 | |
| | | | | | | | | Zfas1 | |
| | | | | | | | | Arpc3 | |
| | | | | | | | | F13a1 | |
| | | | | | | | | Psme1 | |
| | | | | | | | | Pid1 | |
| | | | | | | | | Rpl9.ps7 | |
| | | | | | | | | Hif1a | |
| | | | | | | | | Mdfic | |
| | | | | | | | | Lmnb1 | |
| | | | | | | | | Selenow | |
| | | | | | | | | Trps1 | |
| | | | | | | | | Ncf4 | |
| | | | | | | | | Card19 | |
| | | | | | | | | Gm5835 | |
| | | | | | | | | Shisa5 | |
| | | | | | | | | Tap1 | |
| | | | | | | | | Irgm1 | |
| | | | | | | | | Spop | |
| | | | | | | | | Tpm3.rs7 | |
| | | | | | | | | Pmaip1 | |
| | | | | | | | | Arl5c | |
| | | | | | | | | Cyth1 | |
| | | | | | | | | Daxx | |
| | | | | | | | | Ccl9 | |
| | | | | | | | | Atox1 | |
| | | | | | | | | Baz1a | |
| | | | | | | | | Tes | |
| | | | | | | | | Gm5805 | |
| | | | | | | | | Ninj1 | |
| | | | | | | | | Mrpl33 | |
| | | | | | | | | Gm6204 | |
| | | | | | | | | Rps26 | |
| | | | | | | | | Herc6 | |
| | | | | | | | | Gsn | |
| | | | | | | | | Arpc1b | |
| | | | | | | | | Gm4617 | |
| | | | | | | | | Gm10250 | |
| | | | | | | | | Fth.ps3 | |
| | | | | | | | | Gm14586 | |
| | | | | | | | | Snx20 | |
| | | | | | | | | AI504432 | |
| | | | | | | | | Litaf | |
| | | | | | | | | Rps5 | |
| | | | | | | | | Gm13092 | |
| | | | | | | | | Rps3a1 | |
| | | | | | | | | Got1 | |
| | | | | | | | | Pttg1 | |
| | | | | | | | | Gm11478 | |
| | | | | | | | | Rpl14.ps1 | |
| | | | | | | | | Atp5h | |
| | | | | | | | | Rps19 | |
| | | | | | | | | Sri | |
| | | | | | | | | Tax1bp1 | |
| | | | | | | | | Lsm4 | |
| | | | | | | | | Gm7809 | |
| | | | | | | | | Gm10443 | |
| | | | | | | | | Dnaja2 | |
| | | | | | | | | Nlrp3 | |
| | | | | | | | | Cmpk1 | |
| | | | | | | | | Rps10.ps1 | |
| | | | | | | | | Gm9493 | |
| | | | | | | | | Gm14539 | |
| | | | | | | | | Apobec1 | |
| | | | | | | | | Trafd1 | |
| | | | | | | | | Nmi | |
| | | | | | | | | Cox5a | |
| | | | | | | | | Etf1 | |
| | | | | | | | | Rpl37rt | |
| | | | | | | | | Sap18b | |
| | | | | | | | | Ddx58 | |
| | | | | | | | | Slc7a2 | |
| | | | | | | | | Rpl38.ps2 | |
| | | | | | | | | Sdc4 | |
| | | | | | | | | Tgif1 | |
| | | | | | | | | Npc2 | |
| | | | | | | | | Trim30d | |
| | | | | | | | | Gmfg | |
| | | | | | | | | H3f3a | |
| | | | | | | | | Gm4149 | |
| | | | | | | | | Rack1 | |
| | | | | | | | | Rpsa.ps10 | |
| | | | | | | | | Anxa4 | |
| | | | | | | | | Gm10275 | |
| | | | | | | | | Pilra | |
| | | | | | | | | Eif4ebp1 | |
| | | | | | | | | Kdm7a | |
| | | | | | | | | Fos | |
| | | | | | | | | Sf3b6 | |
| | | | | | | | | Rps11 | |
| | | | | | | | | Scand1 | |
| | | | | | | | | Pitpna | |
| | | | | | | | | 2010107E04Rik | |
| | | | | | | | | Gabarap | |
| | | | | | | | | Arhgdib | |
| | | | | | | | | Rpl8 | |
| | | | | | | | | Nufip1 | |

**Table S5.** Lists of differentially expressed genes between PDOXs and GL261 per each myeloid cell type.
Differentially expressed genes were defined at threshold: FDR <=0.01 and |log2FC|>=1
Genes are listed from the highest to lowest |log2FC|

| Cell type | Microglia | | Monocytes | | BAMs | |
|---|---|---|---|---|---|---|
| Fold change | upregulated | downregulated | upregulated | downregulated | upregulated | downregulated |
| Number of genes | 55 | 63 | 44 | 77 | 0 | 56 |
| | Cxcl13 | H2.Eb1 | Ifi202b | Thbs1 | | Rsad2 |
| | Spp1 | Plp1 | Ftl1 | H2.Eb1 | | Cxcl10 |
| | Ttr | Rpl34 | Ifi44 | Cd14 | | Iigp1 |
| | Lpl | Hspa8 | Cxcl11 | Cxcl2 | | Ptgs2 |
| | Ifi202b | Ccl2 | Cwc22 | Hspa8 | | Il10 |
| | Ank | Rps2 | Gm15772 | Rps2 | | Ifit3 |
| | Cwc22 | H2.Q7 | Gm11942 | Bst2 | | Rnf19b |
| | Gm11942 | Rps18 | Gm10260 | Rpl34 | | Mx1 |
| | Ch25h | Fosb | Igkc | Arg1 | | Rps18 |
| | Fn1 | Cebpb | Ifi214 | Fosb | | Rpl34 |
| | Apoc1 | Hspa1a | Cxcl13 | Gpnmb | | Oasl2 |
| | Ccl6 | Bst2 | Hopx | C1qb | | Hspa8 |
| | Apoe | Ccl5 | H2.Ea.ps | Nr4a2 | | Sdhc |
| | Ftl1 | Ly6a | Rps4x | Rps18 | | Ifi205 |
| | Itgax | Cxcl9 | Ifi211 | C1qc | | Ccl7 |
| | Colec12 | Nr4a1 | Ttr | Fosl2 | | Mndal |
| | Sparcl1 | Gbp2 | H2.T.ps | Saa3 | | Hist1h2bc |
| | Flt1 | Rpl35a | Ighm | Plp1 | | Tmcc3 |
| | Gm10260 | Gbp7 | Ccdc88a | Ptgs2 | | Pnp |
| | Postn | Lect1 | Rps27a | Mrc1 | | Ifi209 |
| | Arsb | Fos | Rpl13a | Pf4 | | Rab11fip5 |
| | Ifi44 | AW112010 | Klrk1 | C1qa | | Timp2 |
| | Scg3 | Mbp | Siglech | Ccl7 | | Numa1 |
| | Ptprz1 | Hpgd | Gbp2b | Rpl29 | | Ssbp4 |
| | Axl | Atf3 | Tpt1 | Il1r2 | | Mx2 |
| | Clu | Rpl29 | Gm4759 | Il1b | | Lrp6 |
| | Gm15772 | Ldhb | Rps7 | Cebpb | | Srsf3 |
| | Ptn | Fosl2 | Cx3cr1 | Cxcl1 | | Msi2 |
| | mt.Co1 | Il1a | Cst3 | Vegfa | | Hpse |
| | Lyzl4 | Crybb1 | Gm7407 | Ccl2 | | Xdh |
| | mt.Cytb | Gm9493 | mt.Co1 | Lyz2 | | A930011G23Rik |
| | Cd34 | H2.Q6 | Gm6472 | Dab2 | | Spg11 |
| | Cacna1a | H2.Ab1 | Igfbp7 | Mt1 | | H6pd |
| | Ctsd | Cd14 | Itpr2 | Sash1 | | Mrps22 |
| | Ttyh1 | Cd83 | Colec12 | Ccr5 | | Cenpb |
| | Myo1e | Cdkn1a | 9930111J21Rik2 | Apoe | | Rpp30 |
| | Il4i1 | Cxcl10 | Rps29 | Rpl30 | | Prkacb |
| | Aqp4 | Il1b | S100a4 | Nlrp3 | | Oasl1 |
| | Scd2 | Tnfaip3 | Rgs1 | Rpl3 | | Tap2 |
| | Gnas | Rpl3 | Sept11 | Fabp5 | | Mrpl34 |
| | mt.Nd2 | Ifrd1 | Igbp1 | Tnfaip3 | | Samd9l |
| | St8sia6 | Fcrls | Gpr183 | Plaur | | Dyrk2 |
| | Cspg4 | Txnip | Rpl10 | Ly86 | | Lap3 |
| | Cadm1 | Dusp1 | Hal | Nfkbiz | | Col3a1 |
| | Tnfsf8 | Ccl12 | | Ccl6 | | Gspt1 |
| | Rgs1 | Rpl30 | | Ifi213 | | Ifi206 |
| | mt.Nd1 | Tnfaip2 | | Clec4n | | Csf2rb |
| | mt.Nd4 | Ifi204 | | Ccr1 | | Nub1 |
| | Atp1a2 | Trim12a | | Trim25 | | Ifi213 |
| | Ramp1 | H2.DMa | | Gm9493 | | Anxa3 |
| | Alcam | Pim1 | | Cd63 | | Iqgap2 |
| | Gatm | Nlrp3 | | Zfp622 | | C1qb |
| | Ccdc80 | Ccl4 | | Ets2 | | Rpl3 |
| | Cst7 | C1qb | | Acp5 | | Ddb1 |
| | Rpl13a | Mcl1 | | Dusp1 | | Col9a1 |
| | | Rnf19b | | Crem | | Plekha2 |
| | | H2.T22 | | Il1a | | |
| | | Nfkbia | | Ctsc | | |
| | | Psmb8 | | Got1 | | |
| | | Ubc | | Gm10443 | | |
| | | Gbp3 | | Ccl9 | | |
| | | Socs3 | | Cd63.ps | | |
| | | Klf4 | | H2.Q7 | | |
| | | | | Fgl2 | | |
| | | | | Metrnl | | |
| | | | | Osm | | |
| | | | | Tiparp | | |
| | | | | Cd93 | | |
| | | | | Prdx2 | | |
| | | | | Mir692.1 | | |
| | | | | Klf4 | | |
| | | | | Sgk1 | | |
| | | | | Spint1 | | |
| | | | | Gm5614 | | |
| | | | | Rpl31.ps8 | | |
| | | | | Nr4a1 | | |
| | | | | Nfkbia | | |

**Table S6.** List of gene signatures applied for scRNA-seq analysis.

| Signature | Human Mg | Human Mo | Enzymatic activation | Homeostatic Mg | Sensome | Sensome w/o homeostatic Mg | Migration | APC | Phagocytosis |
|---|---|---|---|---|---|---|---|---|---|
| Genes | 12 | 13 | 25 | 35 | 140 | 127 | 464 | 106 | 219 |
| | HEXB | CCR2 | Rgs1 | Bhlhe41 | 4632428N05Rik | Abcc3 | Abl1 | Abcb9 | 4932438A13Rik |
| | P2RY12 | PLAC8 | Hist2h2aa1 | Crybb1 | Abcc3 | Adora3 | Acvrl1 | Ap3b1 | 4933434E20Rik |
| | P2RY13 | CLEC12A | Hist1h4i | Csf1r | Adora3 | Adrb2 | Adam17 | Ap3d1 | Abca1 |
| | TMEM119 | FCN1 | Nfkbiz | Ctsd | Adrb2 | Bin2 | Adam8 | Arl8b | Abca7 |
| | GPR34 | VCAN | Klf2 | Ctsf | AF251705 | C3ar1 | Adam9 | Atg5 | Abl1 |
| | CXCR1 | LYZ | Junb | Cttnbp2 | Bin2 | C5ar1 | Adamts9 | B2m | Abl2 |
| | TGFBR1 | LGALS3 | Dusp1 | Cttnbp2nl | C3ar1 | Ccr5 | Adgra2 | Bag6 | Abr |
| | CST3 | CRIP1 | Ccl3 | Cx3cr1 | C5ar1 | Ccrl2 | Adgrb1 | Calr | Adgrb1 |
| | OLFML3 | S100A4 | Hspa1a | Fcrls | C5ar2 | Cd14 | Adgrg1 | Ccr7 | Aif1 |
| | SALL1 | S100A6 | Hsp90aa1 | Golm1 | Ccr5 | Cd180 | Adipor1 | Cd1d1 | Ano6 |
| | SELPLG | S100A8 | Fos | Gpr34 | Ccrl2 | Cd22 | Adora2b | Cd68 | Anxa1 |
| | TREM2 | S100A9 | Hspa1b | Gpr84 | Cd101 | Cd300a | Agt | Cd74 | Anxa3 |
| | | CD44 | Jun | Hexb | Cd14 | Cd33 | Aif1 | Clec4a1 | Appl1 |
| | | | Jund | Hpgds | Cd180 | Cd37 | Akirin1 | Clec4a2 | Appl2 |
| | | | Nfkbid | Hspa1a | Cd22 | Cd48 | Akt1 | Clec4a3 | Arhgap12 |
| | | | Gem | Lgmn | Cd300a | Cd52 | Akt3 | Ctsl | Arhgap25 |
| | | | Ccl4 | Lrrc3 | Cd33 | Cd53 | Amot | Ctss | Arl8b |
| | | | Ier5 | Ltc4s | Cd37 | Cd68 | Amotl1 | Erap1 | Atg3 |
| | | | Txnip | Mafb | Cd48 | Cd74 | Angpt1 | Ext1 | Atg5 |
| | | | Hist1h2bc | Nuak1 | Cd52 | Cd83 | Angpt2 | Fcer1g | Atg7 |
| | | | Zfp36 | Olfml3 | Cd53 | Cd84 | Anln | Fcgr1 | Axl |
| | | | Hist1h1c | P2ry12 | Cd68 | Cd86 | Ano6 | Fcgr2b | Bcr |
| | | | Egr1 | P2ry13 | Cd74 | Clec4a2 | Anxa1 | Fcgr3 | Becn1 |
| | | | Atf3 | Plxdc2 | Cd79b | Clec4a3 | Anxa3 | Fcgr4 | Bin2 |
| | | | Rhob | Rab3il1 | Cd83 | Clec5a | Apoe | Fgl2 | C2 |
| | | | | Sall1 | Cd84 | Clec7a | App | Gba | C3 |
| | | | | Scoc | Cd86 | Cmklr1 | Aqp1 | Gm8909 | C4b |
| | | | | Serpine2 | Clec4a2 | Cmtm6 | Arf6 | H2.Aa | Calr |
| | | | | Siglech | Clec4a3 | Cmtm7 | Arl13b | H2.Ab1 | Camk1d |
| | | | | Slc2a5 | Clec4b1 | Csf2rb2 | Arsb | H2.D1 | Ccl2 |
| | | | | Tgfbr1 | Clec5a | Csf3r | Atoh8 | H2.DMa | Ccr2 |
| | | | | Tmem119 | Clec7a | Cxcl16 | Atp2b4 | H2.DMb1 | Ccr7 |
| | | | | Atp8a2 | Cmklr1 | Cysltr1 | Atp5a1 | H2.DMb2 | Cd300a |
| | | | | Lag3 | Cmtm6 | Daglb | Atp5b | H2.Eb1 | Cd300lf |
| | | | | Slco4a1 | Cmtm7 | Ecscr | Axl | H2.K1 | Cd302 |
| | | | | | Csf1r | Entpd1 | Bcar1 | H2.M3 | Cd36 |
| | | | | | Csf2rb2 | Fcer1g | Bcas3 | H2.Oa | Cd47 |
| | | | | | Csf3r | Fcgr1 | Bmpr2 | H2.Q10 | Cdc42 |
| | | | | | Cx3cr1 | Fcgr2b | Bsg | H2.Q4 | Cdc42se1 |
| | | | | | Cxcl16 | Fcgr3 | Bst1 | H2.O6 | Cdc42se2 |
| | | | | | Cysltr1 | Fcgr4 | C1qbp | H2.Q7 | Cfp |
| | | | | | Daglb | Gpr160 | C3ar1 | H2.T22 | Clcn3 |
| | | | | | Ecscr | Gpr183 | C5ar1 | H2.T23 | Clec7a |
| | | | | | Emr1 | Gsdmd | Calr | H2.T24 | Cln3 |
| | | | | | Entpd1 | H2.Oa | Camk1d | H2.T3 | Cnn2 |
| | | | | | Fcer1g | H2.Ob | Capn7 | Hfe | Colec12 |
| | | | | | Fcgr1 | H2.T23 | Card10 | Icam1 | Coro1a |
| | | | | | Fcgr2b | Havcr2 | Ccdc141 | Ide | Coro1c |
| | | | | | Fcgr3 | Hfe | Ccl12 | Ifi30 | Csk |
| | | | | | Fcgr4 | I830077J02Rik | Ccl17 | Ighm | Cyba |
| | | | | | Fcrl1 | Icam1 | Ccl2 | Mfsd6 | Dnm2 |
| | | | | | Fcrls | Icosl | Ccl24 | Mr1 | Dock1 |
| | | | | | Gpr160 | Ifitm6 | Ccl25 | Nod1 | Dock2 |
| | | | | | Gpr183 | Ifngr1 | Ccl3 | Nod2 | Eif2ak1 |
| | | | | | Gpr34 | Igsf6 | Ccl4 | Pdia3 | Elmo1 |
| | | | | | Gpr56 | Il10ra | Ccl5 | Pikfyve | Elmo2 |
| | | | | | Gpr84 | Il21r | Ccl6 | Psap | Elmo3 |
| | | | | | Gsdmd | Il4ra | Ccl7 | Psmb8 | Fcer1g |
| | | | | | H2-Oa | Il6ra | Ccl8 | Psmb9 | Fcgr1 |
| | | | | | H2-Ob | Itgam | Ccl9 | Psme1 | Fcgr2b |
| | | | | | H2-T23 | Itgb2 | Ccr1 | Psme2 | Fcgr3 |
| | | | | | Havcr2 | Itgb5 | Ccr2 | Ptpn22 | Fgr |
| | | | | | Hfe | Kctd12 | Ccr7 | Pycard | Gas6 |
| | | | | | I830077J02Rik | Lair1 | Cd200 | Rab10 | Gata2 |
| | | | | | Icam1 | Laptm5 | Cd200r1 | Rab27a | Gsn |
| | | | | | Icam4 | Lat2 | Cd40 | Rab32 | Gulp1 |
| | | | | | Icosl | Lgals9 | Cd63 | Rab33a | Hck |
| | | | | | Ifitm6 | Lilra5 | Cd74 | Rab34 | Hmgb1 |
| | | | | | Ifngr1 | Ly86 | Cd81 | Rab35 | Hspa8 |
| | | | | | Igsf6 | Nckap1l | Cd9 | Rab3b | Ighm |
| | | | | | Il10ra | Nfam1 | Cdh13 | Rab3c | Il15 |
| | | | | | Il21r | Ninj1 | Cdh5 | Rab4a | Il15ra |
| | | | | | Il4ra | Numb | Cdk5 | Rab5b | Il1b |
| | | | | | Il6ra | P2ry6 | Cdk5r1 | Rab6a | Il2rg |
| | | | | | Itgam | Pilra | Ceacam1 | Rab8b | Irf8 |
| | | | | | Itgb2 | Plekho1 | Cib1 | Relb | Itga2 |
| | | | | | Itgb5 | Pmepa1 | Cklf | Rftn1 | Itgal |
| | | | | | Kctd12 | Pmp22 | Clasp1 | Slc11a1 | Itgam |
| | | | | | Lag3 | Ptafr | Clasp2 | Tap1 | Itgav |
| | | | | | Lair1 | Ptprc | Clec14a | Tap2 | Itgb1 |
| | | | | | Laptm5 | Selplg | Cmklr1 | Tapbp | Itgb2 |
| | | | | | Lat2 | Sirpa | Cntn2 | Tapbpl | Itgb3 |
| | | | | | Lgals9 | Slamf9 | Col3a1 | Thbs1 | Jmjd6 |
| | | | | | Lilra5 | Slc11a1 | Coro1b | Traf6 | Lbp |
| | | | | | Liph | Slc16a3 | Coro1c | Trem2 | Ldlr |
| | | | | | Lpar5 | Slc44a2 | Creb3 | Unc93b1 | Lepr |
| | | | | | Ltf | Slc46a1 | Csf1 | Was | Letmd1 |
| | | | | | Ly86 | Slc7a7 | Csf3r | Wdfy4 | Lman2 |
| | | | | | Nckap1l | Slc7a8 | Csnk2b | Ythdf1 | Lrp1 |
| | | | | | Nfam1 | Slco2b1 | Ctnnb1 | H2.M5 | Lyar |
| | | | | | Ninj1 | Stab1 | Ctsh | Kdm5d | Lyst |
| | | | | | Numb | Tcirg1 | Cul5 | Flt3 | Masp2 |
| | | | | | P2ry12 | Tgfbr2 | Cx3cl1 | Gm11127 | Megf10 |
| | | | | | P2ry13 | Tlr13 | Cxadr | H2.Ob | Mertk |
| | | | | | P2ry6 | Tlr2 | Cxcl1 | H2.Eb2 | Met |
| | | | | | Pilra | Tlr4 | Cxcl10 | H2.M2 | Mex3b |
| | | | | | Plekho1 | Tlr6 | Cxcl12 | H2.Q1 | Mfge8 |
| | | | | | Plxdc2 | Tlr7 | Cxcl13 | H2.Q2 | Msr1 |
| | | | | | Pmepa1 | Tmem173 | Cxcl2 | Treml4 | Myd88 |
| | | | | | Pmp22 | Tmem206 | Cxcl9 | Ifng | Myh9 |
| | | | | | Ptafr | Tmem37 | Cxcr4 | Clec4b1 | Myo18a |
| | | | | | Ptprc | Tnfrsf13b | Cyp1b1 | Ctse | Myo1g |
| | | | | | Selplg | Tnfrsf1a | Daam2 | H2.M9 | Myo7a |
| | | | | | Siglec5 | Tnfrsf1b | Dab1 | Azgp1 | Ncf2 |
| | | | | | Siglece | Trem2 | Dab2ip | Gm7030 | Ncf4 |
| | | | | | Siglech | Trpv2 | Dcn | Ccl21a | Nckap1l |
| | | | | | Sirpa | Tyrobp | Dicer1 | | Nod2 |
| | | | | | Slamf9 | Icam4 | Disc1 | | Nr1h3 |
| | | | | | Slc11a1 | Cd79b | Dixdc1 | | P2rx7 |
| | | | | | Slc16a3 | Siglece | Dnaja4 | | P2ry6 |
| | | | | | Slc2a5 | Tlr1 | Dnm1l | | Pear1 |
| | | | | | Slc44a2 | C5ar2 | Dock1 | | Pecam1 |
| | | | | | Slc46a1 | Cd101 | Dock5 | | Pik3ca |
| | | | | | Slc7a7 | Liph | Dpp4 | | Pikfyve |
| | | | | | Slc7a8 | Tlr12 | Dusp1 | | Pip5k1c |
| | | | | | Slco2b1 | Tnfrsf17 | Dusp10 | | Pla2g5 |
| | | | | | Stab1 | Upk1b | Edn1 | | Plcg2 |
| | | | | | Tcirg1 | Fcrl1 | Edn3 | | Pld4 |
| | | | | | Tgfbr1 | Tmem8c | Ednra | | Pot1b |
| | | | | | Tgfbr2 | Clec4b1 | Ednrb | | Prkcg |
| | | | | | Tlr1 | Lpar5 | Efhc1 | | Pros1 |
| | | | | | Tlr12 | 4632428N05Rik | Efna1 | | Pten |
| | | | | | Tlr13 | AF251705 | Efnb2 | | Ptk2 |
| | | | | | Tlr2 | Emr1 | Egf | | Ptprc |
| | | | | | Tlr4 | Gpr56 | Egfr | | Ptprj |
| | | | | | Tlr6 | Siglec5 | Emc10 | | Ptx3 |
| | | | | | Tlr7 | Ltf | Emilin1 | | Pycard |
| | | | | | Tmem119 | | Emp2 | | Rab11fip2 |
| | | | | | Tmem173 | | Emx2 | | Rab14 |
| | | | | | Tmem206 | | Enpp1 | | Rab20 |
| | | | | | Tmem37 | | Enpp2 | | Rab27a |
| | | | | | Tmem8c | | Epb41l4b | | Rab31 |
| | | | | | Tnfrsf13b | | Epb41l5 | | Rab34 |
| | | | | | Tnfrsf17 | | Epha2 | | Rab5a |
| | | | | | Tnfrsf1a | | Ephb4 | | Rab7 |
| | | | | | Tnfrsf1b | | Ets1 | | Rab7b |
| | | | | | Trem2 | | Evl | | Rac1 |
| | | | | | Trpv2 | | Fap | | Rac2 |
| | | | | | Tyrobp | | Fbxo45 | | Rack1 |
| | | | | | Upk1b | | Fbxw7 | | Rap1a |
| | | | | | | | Fcer1g | | Rap1gap |
| | | | | | | | Fcgr3 | | Rapgef1 |
| | | | | | | | Fgf1 | | Rara |
| | | | | | | | Fgf2 | | Rhobtb1 |

| | | | | | | | | | |
|---|---|---|---|---|---|---|---|---|---|
| | | | | | | | Fgf7 | | Rhobtb2 |
| | | | | | | | Fgfr1 | | Rhog |
| | | | | | | | Flt1 | | Rhoh |
| | | | | | | | Flt4 | | Scarb1 |
| | | | | | | | Foxc2 | | Sh3bp1 |
| | | | | | | | Foxg1 | | Sirpa |
| | | | | | | | Foxp1 | | Slc11a1 |
| | | | | | | | Fstl1 | | Snx3 |
| | | | | | | | Fut10 | | Sod1 |
| | | | | | | | Gab2 | | Spg11 |
| | | | | | | | Gadd45a | | Sphk1 |
| | | | | | | | Gata2 | | Stap1 |
| | | | | | | | Gbf1 | | Syk |
| | | | | | | | Gipc1 | | Syt11 |
| | | | | | | | Gli3 | | Syt7 |
| | | | | | | | Glipr2 | | Tgfb1 |
| | | | | | | | Glul | | Tgm2 |
| | | | | | | | Gpi1 | | Thbs1 |
| | | | | | | | Gpld1 | | Tlr2 |
| | | | | | | | Gpr35 | | Tlr4 |
| | | | | | | | Gpx1 | | Tm9sf4 |
| | | | | | | | Grn | | Tmem175 |
| | | | | | | | Has2 | | Tnf |
| | | | | | | | Hbegf | | Trbc2 |
| | | | | | | | Hc | | Trem2 |
| | | | | | | | Hdac5 | | Tub |
| | | | | | | | Hdac6 | | Tusc2 |
| | | | | | | | Hdac7 | | Tyro3 |
| | | | | | | | Hdac9 | | Tyrobp |
| | | | | | | | Hif1a | | Unc13d |
| | | | | | | | Hmgb1 | | Vamp7 |
| | | | | | | | Hmox1 | | Vav1 |
| | | | | | | | Hspb1 | | Xkr5 |
| | | | | | | | Hyal1 | | Xkr6 |
| | | | | | | | Igf1 | | Xkr8 |
| | | | | | | | Igf2 | | Fer1l5 |
| | | | | | | | Il1b | | Icam5 |
| | | | | | | | Il34 | | Trbc1 |
| | | | | | | | Iqsec1 | | Apoa2 |
| | | | | | | | Itga1 | | Il2rb |
| | | | | | | | Itga2 | | Mbl2 |
| | | | | | | | Itga3 | | Cfb |
| | | | | | | | Itga9 | | Siglece |
| | | | | | | | Itgam | | Sirpb1b |
| | | | | | | | Itgav | | Sirpb1c |
| | | | | | | | Itgb1 | | Ticam2 |
| | | | | | | | Itgb1bp1 | | Xkr4 |
| | | | | | | | Itgb2 | | Gm9733 |
| | | | | | | | Itgb3 | | Dysf |
| | | | | | | | Jam3 | | Gm5150 |
| | | | | | | | Jun | | Ighd |
| | | | | | | | Jup | | Igkc |
| | | | | | | | Kank1 | | Iglc3 |
| | | | | | | | Kank2 | | Rab39 |
| | | | | | | | Kdr | | Sirpb1a |
| | | | | | | | Kit | | Slamf1 |
| | | | | | | | Klf4 | | Treml4 |
| | | | | | | | Krit1 | | F2rl1 |
| | | | | | | | Lamb1 | | Ifng |
| | | | | | | | Lbp | | Marco |
| | | | | | | | Lemd3 | | Prtn3 |
| | | | | | | | Lgals3 | | Ager |
| | | | | | | | Lgals8 | | Apoa1 |
| | | | | | | | Loxl2 | | Cd209b |
| | | | | | | | Lpxn | | Iglc1 |
| | | | | | | | Lrp6 | | Iglc2 |
| | | | | | | | Lrp8 | | Masp1 |
| | | | | | | | Lrrk2 | | Spon2 |
| | | | | | | | Lyn | | Srpx |
| | | | | | | | Macf1 | | Tulp1 |
| | | | | | | | Map2k3 | | Alox15 |
| | | | | | | | Map2k5 | | Havcr1 |
| | | | | | | | Map3k3 | | Timd4 |
| | | | | | | | Map4k4 | | Trdc |
| | | | | | | | Mapk1 | | Colec11 |
| | | | | | | | Mapk3 | | |
| | | | | | | | Mapre2 | | |
| | | | | | | | Mboat7 | | |
| | | | | | | | Mcc | | |
| | | | | | | | Mcu | | |
| | | | | | | | Mdk | | |
| | | | | | | | Mecp2 | | |
| | | | | | | | Mef2c | | |
| | | | | | | | Met | | |
| | | | | | | | Mia3 | | |
| | | | | | | | Mif | | |
| | | | | | | | Mmp14 | | |
| | | | | | | | Mmp28 | | |
| | | | | | | | Mmrn2 | | |
| | | | | | | | Mospd2 | | |
| | | | | | | | Mpp1 | | |
| | | | | | | | Mtor | | |
| | | | | | | | Mtus1 | | |
| | | | | | | | Myh9 | | |
| | | | | | | | Myo9b | | |
| | | | | | | | Nanos1 | | |
| | | | | | | | Nbl1 | | |
| | | | | | | | Nckap1l | | |
| | | | | | | | Ndel1 | | |
| | | | | | | | Nf1 | | |
| | | | | | | | Nfe2l2 | | |
| | | | | | | | Ninj1 | | |
| | | | | | | | Nod2 | | |
| | | | | | | | Nos3 | | |
| | | | | | | | Notch1 | | |
| | | | | | | | Nr2e1 | | |
| | | | | | | | Nr2f2 | | |
| | | | | | | | Nr4a1 | | |
| | | | | | | | Nrp1 | | |
| | | | | | | | Nup85 | | |
| | | | | | | | Nus1 | | |
| | | | | | | | P2rx4 | | |
| | | | | | | | Pafah1b1 | | |
| | | | | | | | Patz1 | | |
| | | | | | | | Paxip1 | | |
| | | | | | | | Pdcd10 | | |
| | | | | | | | Pdcd6 | | |
| | | | | | | | Pde4b | | |
| | | | | | | | Pde4d | | |
| | | | | | | | Pdgfb | | |
| | | | | | | | Pdpk1 | | |
| | | | | | | | Pecam1 | | |
| | | | | | | | Pex13 | | |
| | | | | | | | Pex5 | | |
| | | | | | | | Pf4 | | |
| | | | | | | | Pfn1 | | |
| | | | | | | | Pfn2 | | |
| | | | | | | | Pik3c2a | | |
| | | | | | | | Pik3cb | | |
| | | | | | | | Pik3cd | | |
| | | | | | | | Pik3cg | | |
| | | | | | | | Pik3r2 | | |
| | | | | | | | Pikfyve | | |
| | | | | | | | Pkn1 | | |
| | | | | | | | Pkn2 | | |
| | | | | | | | Pkn3 | | |
| | | | | | | | Pla2g7 | | |
| | | | | | | | Plcg1 | | |
| | | | | | | | Plcg2 | | |
| | | | | | | | Plekhg5 | | |
| | | | | | | | Plk2 | | |
| | | | | | | | Plpp3 | | |
| | | | | | | | Plxnd1 | | |
| | | | | | | | Pou3f2 | | |
| | | | | | | | Pou3f3 | | |
| | | | | | | | Ppard | | |
| | | | | | | | Ppia | | |
| | | | | | | | Ppib | | |
| | | | | | | | Ppm1f | | |
| | | | | | | | Prcp | | |

| | | | | | | | | | |
|---|---|---|---|---|---|---|---|---|---|
| | | | | | | | Prex1 | | |
| | | | | | | | Prkca | | |
| | | | | | | | Prkce | | |
| | | | | | | | Prkd1 | | |
| | | | | | | | Prkd2 | | |
| | | | | | | | Prox1 | | |
| | | | | | | | Psen1 | | |
| | | | | | | | Pten | | |
| | | | | | | | Ptgs2 | | |
| | | | | | | | Ptk2 | | |
| | | | | | | | Ptk2b | | |
| | | | | | | | Ptp4a3 | | |
| | | | | | | | Ptpn11 | | |
| | | | | | | | Ptpn23 | | |
| | | | | | | | Ptprg | | |
| | | | | | | | Ptprj | | |
| | | | | | | | Ptprm | | |
| | | | | | | | Ptpro | | |
| | | | | | | | Pxn | | |
| | | | | | | | Rab11a | | |
| | | | | | | | Rab13 | | |
| | | | | | | | Rac1 | | |
| | | | | | | | Rac2 | | |
| | | | | | | | Rarres2 | | |
| | | | | | | | Reln | | |
| | | | | | | | Rgcc | | |
| | | | | | | | Rhoa | | |
| | | | | | | | Rhob | | |
| | | | | | | | Rhoj | | |
| | | | | | | | Rin2 | | |
| | | | | | | | Rnf7 | | |
| | | | | | | | Robo1 | | |
| | | | | | | | Rock1 | | |
| | | | | | | | Rock2 | | |
| | | | | | | | Rpl13a | | |
| | | | | | | | Rps19 | | |
| | | | | | | | Rras | | |
| | | | | | | | Rreb1 | | |
| | | | | | | | Rtn4 | | |
| | | | | | | | Sash1 | | |
| | | | | | | | Scarb1 | | |
| | | | | | | | Sell | | |
| | | | | | | | Sema3a | | |
| | | | | | | | Sema4a | | |
| | | | | | | | Sema5a | | |
| | | | | | | | Serpine1 | | |
| | | | | | | | Serpinf1 | | |
| | | | | | | | Sh3bp1 | | |
| | | | | | | | Sirt1 | | |
| | | | | | | | Slc37a4 | | |
| | | | | | | | Slit2 | | |
| | | | | | | | Smoc2 | | |
| | | | | | | | Snai2 | | |
| | | | | | | | Socs7 | | |
| | | | | | | | Sox9 | | |
| | | | | | | | Sp1 | | |
| | | | | | | | Sp100 | | |
| | | | | | | | Sparc | | |
| | | | | | | | Spp1 | | |
| | | | | | | | Spred1 | | |
| | | | | | | | Src | | |
| | | | | | | | Srf | | |
| | | | | | | | Srgap2 | | |
| | | | | | | | Srpx2 | | |
| | | | | | | | Stap1 | | |
| | | | | | | | Stard13 | | |
| | | | | | | | Stat5a | | |
| | | | | | | | Sun1 | | |
| | | | | | | | Sun2 | | |
| | | | | | | | Svbp | | |
| | | | | | | | Syk | | |
| | | | | | | | Syne2 | | |
| | | | | | | | Synj2bp | | |
| | | | | | | | Tbxa2r | | |
| | | | | | | | Tek | | |
| | | | | | | | Tesk1 | | |
| | | | | | | | Tgfb1 | | |
| | | | | | | | Tgfb2 | | |
| | | | | | | | Tgfbr2 | | |
| | | | | | | | Tgfbr3 | | |
| | | | | | | | Thbs1 | | |
| | | | | | | | Tirap | | |
| | | | | | | | Tnf | | |
| | | | | | | | Tnfaip6 | | |
| | | | | | | | Tnr | | |
| | | | | | | | Trem1 | | |
| | | | | | | | Trem2 | | |
| | | | | | | | Trpv4 | | |
| | | | | | | | Tyro3 | | |
| | | | | | | | Vash1 | | |
| | | | | | | | Vav1 | | |
| | | | | | | | Vav3 | | |
| | | | | | | | Vegfa | | |
| | | | | | | | Vhl | | |
| | | | | | | | Vil1 | | |
| | | | | | | | Wdpcp | | |
| | | | | | | | Wnt5a | | |
| | | | | | | | Zc3h12a | | |
| | | | | | | | Zeb2 | | |
| | | | | | | | Zfp580 | | |
| | | | | | | | Zmiz1 | | |
| | | | | | | | Il23a | | |
| | | | | | | | Mst1 | | |
| | | | | | | | Ccl22 | | |
| | | | | | | | Xcl1 | | |
| | | | | | | | Apoh | | |
| | | | | | | | Cxcl3 | | |
| | | | | | | | Gata3 | | |
| | | | | | | | Gdf2 | | |
| | | | | | | | S100a14 | | |
| | | | | | | | Arx | | |
| | | | | | | | Bmper | | |
| | | | | | | | Ccl11 | | |
| | | | | | | | Dcx | | |
| | | | | | | | Dll4 | | |
| | | | | | | | Fezf2 | | |
| | | | | | | | Fgf13 | | |
| | | | | | | | Lcn2 | | |
| | | | | | | | Ptprr | | |
| | | | | | | | Stc1 | | |
| | | | | | | | Thbs4 | | |
| | | | | | | | Wnt7a | | |
| | | | | | | | Cdk5r2 | | |
| | | | | | | | Bmp4 | | |
| | | | | | | | C5ar2 | | |
| | | | | | | | Ccr6 | | |
| | | | | | | | Dysf | | |
| | | | | | | | Nrg1 | | |
| | | | | | | | S100a8 | | |
| | | | | | | | Slamf1 | | |
| | | | | | | | Slamf8 | | |
| | | | | | | | Trem3 | | |
| | | | | | | | Vegfc | | |
| | | | | | | | Vstm4 | | |
| | | | | | | | Mcoln2 | | |
| | | | | | | | Dapk2 | | |
| | | | | | | | Ccl1 | | |
| | | | | | | | Ifng | | |
| | | | | | | | Tac1 | | |
| | | | | | | | Tnfsf11 | | |
| | | | | | | | Ager | | |
| | | | | | | | Alox12 | | |
| | | | | | | | Apoa1 | | |
| | | | | | | | Cxcl5 | | |
| | | | | | | | Defb25 | | |
| | | | | | | | Fgf18 | | |
| | | | | | | | Fgfbp1 | | |
| | | | | | | | Foxb1 | | |
| | | | | | | | Lgals12 | | |
| | | | | | | | Mdga1 | | |
| | | | | | | | Slit1 | | |
| | | | | | | | Adtrp | | |
| | | | | | | | Ccbe1 | | |
| | | | | | | | Drd1 | | |
| | | | | | | | Fgf10 | | |
| | | | | | | | Lhx6 | | |
| | | | | | | | Lrg1 | | |
| | | | | | | | Meox2 | | |
| | | | | | | | Mmp9 | | |
| | | | | | | | Msmp | | |
| | | | | | | | Mstn | | |
| | | | | | | | Ror2 | | |
| | | | | | | | S100a9 | | |
| | | | | | | | Tacr1 | | |
| | | | | | | | Cxcr2 | | |
| | | | | | | | Egr3 | | |
| | | | | | | | Trim55 | | |
| | | | | | | | Nrg3 | | |
| | | | | | | | Fermt1 | | |
| | | | | | | | Ccl21a | | |

**Table S7.** Lists of differentially expressed genes between TMZ treated and control P3 PDOXs per each major cell type analysed.
Differentially expressed genes were defined at threshold: FDR <=0.01 and |log2FC|>=0.5
Genes are listed from the highest to lowest $|log_2FC|$

| Cell origin | mouse TME | | | | | | human GBM | |
|---|---|---|---|---|---|---|---|---|
| Cell type | Astrocytes | | Endothelial cells | | Myeloid cells | | Tumor cells | |
| Fold change | upregulated | downregulated | upregulated | downregulated | upregulated | downregulated | upregulated | downregulated |
| Number of genes | 2 | 1 | 41 | 47 | 127 | 28 | 90 | 78 |
| | Scg2 | Gm12222 | RP23.269H21.1 | Ttr | Rnaset2b | Rpl26 | GDF15 | BCAN |
| | Aldoc | | Rnaset2b | Rpl26 | Gm14513 | Rpl35a | CDKN1A | ID3 |
| | | | mt.Tc | Rpl35a | Cxcl13 | Igfbp7 | SCG2 | C1orf61 |
| | | | Gm7638 | Hexb | Gm7638 | Ttr | CHI3L1 | HES5 |
| | | | Abcb1a | Apln | Pde3b | Ptn | NEAT1 | CSPG5 |
| | | | Cxcl12 | Rpl39.ps | Ptgs1 | Gng5 | GADD45A | FABP7 |
| | | | Pcp4l1 | Ctsd | Jund | Rps15a.ps5 | RPS27L | HMGCS1 |
| | | | Paqr5 | Cwc22 | Slco2b1 | Gm10076 | FTL | CST3 |
| | | | Timp3 | Lrp8 | Spp1 | Rpl22.ps1 | MDM2 | HSPA1A |
| | | | Slc6a6 | Myh10 | Zfhx3 | Enpp2 | DDB2 | HSPA1B |
| | | | H2.D1 | Ctss | Rpsa | Gm12338 | GAP43 | NCAN |
| | | | Hsp25.ps1 | Tyrobp | Apoe | Rps15a.ps7 | OCIAD2 | MSMO1 |
| | | | Degs2 | Ndufs5 | P2ry12 | Gm5644 | NRP2 | TTYH1 |
| | | | Fam32a | Rpl36a.ps2 | Tmem119 | Gas5 | S100A6 | ACAT2 |
| | | | Ly6a | Dok4 | Cfl1 | Gm10269 | BTG1 | HEY1 |
| | | | Ppil4 | Hopx | Cnpy2 | Cd52 | PHLDA1 | FDPS |
| | | | Ucp2 | Hspa5 | Cx3cr1 | Rpl36a.ps2 | SPP1 | DBI |
| | | | Hspb1 | Slc7a1 | Rpl9.ps6 | Ndufa4 | SQSTM1 | IDH1 |
| | | | Ly6c1 | Lyz2 | Cited2 | Rpl37rt | TIMP1 | METRN |
| | | | Serinc3 | Gm7266 | Tubgcp5 | Gm4332 | SLC3A2 | LMO4 |
| | | | Sparcl1 | Wwtr1 | Mpc1 | Rplp2 | ZMAT3 | PEA15 |
| | | | Lrg1 | Ccny | Fscn1 | Rpl23 | C6orf141 | MT3 |
| | | | Maoa | Rpl38.ps2 | Ltc4s | Gm4149 | TUBA1C | EDNRB |
| | | | Aldh2 | Rps15a.ps5 | Gm10263 | Rps16.ps2 | IGFBP3 | SCRG1 |
| | | | mt.Rnr1 | Slc38a2 | Mylip | Rps21 | PHPT1 | NDRG2 |
| | | | Tspo | Rps11.ps3 | Ftl1 | Ndufs5 | PHLDA3 | CYP51A1 |
| | | | U2af1 | Gas5 | Aimp1 | Rpl32 | IDS | CNN3 |
| | | | Pltp | Gm10076 | Dtnbp1 | Rps15 | GAS5 | HIST1H4C |
| | | | mt.Rnr2 | Gm15148 | C1qa | | VGF | RPS10 |
| | | | Cd59a | RP23.288C18.3 | Ucp2 | | DDIT3 | YWHAE |
| | | | Gm694 | Cst3 | Pwwp2a | | ARL4C | FDFT1 |
| | | | Mal | Pmepa1 | Rhob | | MALAT1 | FAM181B |
| | | | Gm14513 | Gm10269 | Coro1a | | FDXR | ARC |
| | | | Ly6e | Rflnb | Pik3cg | | RPS19 | ATP1A2 |
| | | | Bsg | Rpl37rt | Zfp90 | | BAX | IDI1 |
| | | | Malat1 | Nostrin | Rpl13 | | LMNA | MARCKS |
| | | | Ifitm2 | Trf | Gpr34 | | GABPB1-AS1 | ASCL1 |
| | | | Utrn | Calr | Frmd4a | | CAMK2D | FOS |
| | | | Ubb | Rps11.ps2 | Ifngr1 | | RCAN1 | GNAI2 |
| | | | Gm11560 | Gm4149 | Scamp2 | | PMEPA1 | SAMD1 |
| | | | Cldn5 | Sparc | Rpl18.ps1 | | IGFBP5 | HES4 |
| | | | | Tmsb4x | Lrp1 | | LONRF2 | SDC3 |
| | | | | Hsp90b1 | Sox4 | | MDK | STMN1 |
| | | | | Gm9794 | Selplg | | ENC1 | IGFBP2 |
| | | | | Tmsb10 | Qpct | | AEN | LRRC17 |
| | | | | Rpl32 | Sltm | | PGM2L1 | TUBB2B |
| | | | | Rplp1 | Rpl10.ps3 | | TNFRSF12A | TMSB4X |
| | | | | | Gpsm3 | | CEBPB | TUBA1B |
| | | | | | Lamtor1 | | ZFAS1 | ITM2C |
| | | | | | Ier3 | | SRPX | NRARP |
| | | | | | Tmem100 | | NUPR1 | MGST3 |
| | | | | | Sdhd | | HBEGF | SQLE |
| | | | | | Commd2 | | YBX3 | SLC1A3 |
| | | | | | Eif4g2 | | ANXA2 | CHMP4B |
| | | | | | Arl4c | | PMAIP1 | ID1 |
| | | | | | Mknk1 | | VMP1 | GFAP |
| | | | | | Dnm2 | | ASCC3 | TMSB15A |
| | | | | | Kctd12 | | RBP1 | PLPP3 |
| | | | | | Scd2 | | PLK3 | CAMTA1 |
| | | | | | Fam76b | | TRIAP1 | GATM |
| | | | | | Ankle2 | | TMSB10 | MDFI |
| | | | | | Scarb2 | | APLP1 | HSPA8 |
| | | | | | Ly6e | | PLXNB2 | HNRNPA2B1 |
| | | | | | Gm15536 | | SLC25A37 | QKI |
| | | | | | Rab10 | | AC010198.2 | SLC6A11 |
| | | | | | Gm10443 | | TMEM158 | ARL6IP6 |
| | | | | | Hpgds | | KCNF1 | TUBB2A |
| | | | | | Mxi1 | | SESN2 | OLIG1 |
| | | | | | Zfand6 | | AL353138.1 | MT1M |
| | | | | | Prdx5 | | CMBL | APCDD1 |
| | | | | | H2.T.ps | | CCND1 | TSPAN3 |
| | | | | | Naa50 | | CIRBP | FGFBP3 |
| | | | | | Tmem86a | | FBXO22 | AC004540.2 |
| | | | | | Slc29a3 | | S100A16 | MT2A |
| | | | | | Abca1 | | SMIM3 | ID2 |
| | | | | | Atp6v0a1 | | JAG1 | MARCKSL1 |
| | | | | | Ninj1 | | LGALS3 | PAFAH1B3 |
| | | | | | Limd2 | | ARHGEF2 | SAPCD2 |
| | | | | | Olfml3 | | PDLIM4 | |
| | | | | | Elf1 | | XPC | |
| | | | | | Prune2 | | TIGAR | |
| | | | | | Arpc4 | | BLOC1S2 | |
| | | | | | Zfp36l1 | | TXNIP | |
| | | | | | Timp2 | | CEBPG | |
| | | | | | Msr1 | | METTL7B | |
| | | | | | Bin1 | | PRKCQ-AS1 | |
| | | | | | Parvg | | QDPR | |
| | | | | | Marcks | | SCG5 | |
| | | | | | Gm8979 | | SH3BGRL3 | |
| | | | | | Ywhae | | PDGFC | |
| | | | | | Phyh | | | |
| | | | | | Rgs19 | | | |
| | | | | | Sp3 | | | |
| | | | | | Arl6ip4 | | | |
| | | | | | Tuba1b | | | |
| | | | | | Ndufs7 | | | |
| | | | | | Cd37 | | | |
| | | | | | Comt | | | |
| | | | | | Brk1 | | | |
| | | | | | Rpl10a | | | |
| | | | | | Sun2 | | | |
| | | | | | Scoc | | | |
| | | | | | Rtn4rl1 | | | |
| | | | | | Colgalt1 | | | |
| | | | | | Crebrf | | | |
| | | | | | Pla2g7 | | | |
| | | | | | H2.D1 | | | |
| | | | | | Abcg1 | | | |
| | | | | | Srgap2 | | | |
| | | | | | Gla | | | |
| | | | | | Gm8399 | | | |
| | | | | | Ube2d3 | | | |
| | | | | | Lgmn | | | |
| | | | | | mt.Co1 | | | |
| | | | | | Bmyc | | | |
| | | | | | Csf1r | | | |
| | | | | | Gm8606 | | | |
| | | | | | Id2 | | | |
| | | | | | Cndp2 | | | |
| | | | | | Zfp62 | | | |
| | | | | | Lyl1 | | | |
| | | | | | Iah1 | | | |
| | | | | | Dock10 | | | |
| | | | | | Fam49b | | | |
| | | | | | Kdm7a | | | |
| | | | | | Psmc4 | | | |
| | | | | | Selenop | | | |
